# Heterologous cAd3-Ebola and MVA-EbolaZ vaccines are safe and immunogenic in US and Uganda phase 1/1b trials

**DOI:** 10.1038/s41541-024-00833-z

**Published:** 2024-03-29

**Authors:** Myra Happe, Amelia R. Hofstetter, Jing Wang, Galina V. Yamshchikov, LaSonji A. Holman, Laura Novik, Larisa Strom, Francis Kiweewa, Salim Wakabi, Monica Millard, Colleen F. Kelley, Sarah Kabbani, Srilatha Edupuganti, Allison Beck, Florence Kaltovich, Tamar Murray, Susanna Tsukerman, Derick Carr, Carl Ashman, Daphne A. Stanley, Aurélie Ploquin, Robert T. Bailer, Richard Schwartz, Fatim Cham, Allan Tindikahwa, Zonghui Hu, Ingelise J. Gordon, Nadine Rouphael, Katherine V. Houser, Emily E. Coates, Barney S. Graham, Richard A. Koup, John R. Mascola, Nancy J. Sullivan, Merlin L. Robb, Julie A. Ake, Kirsten E. Lyke, Mark J. Mulligan, Julie E. Ledgerwood, Hannah Kibuuka, the VRC 208 and RV 422 study team, Joseph P. Casazza, Grace L. Chen, Mary E. Enama, Martin R. Gaudinski, Cynthia Starr Hendel, Pamela J. M. Costner, Brenda Larkin, Floreliz Mendoza, Jamie Sanders, William R. Whalen, Kathryn L. Zephir, Judith Straling, Hope DeCederfelt, Michelle Conan-Cibotti, Judy Stein, Iris R. Pittman, Olga Vasilenko, Adam DeZure, Sandra Sitar, Lesia K. Dropulic, Sarah H. Plummer, Thuy A. Nguyen, Nina M. Berkowitz, Nancy Greenberg, Lisa Chrisley, Melissa Billington, Xiaolin Wang, JoAnna Becker, James D. Campbell, Wilbur H. Chen, Alyson Kwon, Brenda Dorsey, Jennifer Courneya, Panagiota Komninou, Myounghee Lee, Mary Bower, Charles A. Bailey, Wendy Nesheim, Tigisty Girmay, Jianguo Xu, Melinda Ogilvie, Joann Sadowski, Eileen Osinski, Lilin Lai, Vicki Grimes, Moses R. Kamya, Nelson L. Michael, Francis Kajumba, Jinantat Ananworanich, Betty Mwesigwa, Geofrey Kimbugne, Kenneth Luzinda, Immaculate Nakabuye, Maureen G. Mukyala, Mable Kabahubya, Lydia Nakibuuka, Robinah Matovu

**Affiliations:** 1 Vaccine Research Center, National Institute of Allergy and Infectious Diseases, National Institutes of Health, Bethesda, MD USA; 2 Clinical Monitoring Research Program Directorate, Frederick National Laboratory for Cancer Research, Frederick, MD USA; 3 Makerere University-Walter Reed Project, Kampala, Uganda; 4 Walter Reed Army Institute of Research, Silver Spring, MD USA; 5 Department of Medicine, Division of Infectious Diseases, The Hope Clinic of the Emory Vaccine Center, Emory University, Atlanta, GA USA; 6 Biostatistics Research Branch, Division of Clinical Research, National Institute of Allergy and Infectious Diseases, National Institutes of Health, Bethesda, MD USA; 7 Henry M. Jackson Foundation for the Advancement of Military Medicine, Bethesda, MD USA; 8 University of Maryland School of Medicine, Center for Vaccine Development and Global Health, Baltimore, MD USA

**Keywords:** Vaccines, Viral infection

## Abstract

Ebola virus disease (EVD) is a filoviral infection caused by virus species of the *Ebolavirus* genus including *Zaire ebolavirus* (EBOV) and *Sudan ebolavirus* (SUDV). We investigated the safety and immunogenicity of a heterologous prime-boost regimen involving a chimpanzee adenovirus 3 vectored Ebola vaccine [either monovalent (cAd3-EBOZ) or bivalent (cAd3-EBO)] prime followed by a recombinant modified vaccinia virus Ankara EBOV vaccine (MVA-EbolaZ) boost in two phase 1/1b randomized open-label clinical trials in healthy adults in the United States (US) and Uganda (UG). Trial US (NCT02408913) enrolled 140 participants, including 26 EVD vaccine-naïve and 114 cAd3-Ebola-experienced participants (April-November 2015). Trial UG (NCT02354404) enrolled 90 participants, including 60 EVD vaccine-naïve and 30 DNA Ebola vaccine-experienced participants (February-April 2015). All tested vaccines and regimens were safe and well tolerated with no serious adverse events reported related to study products. Solicited local and systemic reactogenicity was mostly mild to moderate in severity. The heterologous prime-boost regimen was immunogenic, including induction of durable antibody responses which peaked as early as two weeks and persisted up to one year after each vaccination. Different prime-boost intervals impacted the magnitude of humoral and cellular immune responses. The results from these studies demonstrate promising implications for use of these vaccines in both prophylactic and outbreak settings.

## Introduction

Ebola virus disease (EVD) is one of the World Health Organization’s (WHO) priority diseases posing the greatest risk to global health^1^. Most viruses of the six known species of *Ebolavirus* are known to cause hemorrhagic fever in humans^2,3^. *Zaire ebolavirus* (EBOV) and *Sudan ebolavirus* (SUDV) have been responsible for over thirty human outbreaks since the discovery of EVD in 1976 with average case fatality rates (CFRs) of 67% and 48%, respectively^4^. Outbreaks of EVD have occurred with increased frequency in the 21^st^ century^5–7^. The 2014–2016 West African epidemic of EBOV was the largest EVD outbreak in history; it spread widely from Guinea into Liberia and Sierra Leone, infected nearly 30,000 individuals and resulted in 11,310 deaths^8^. WHO declared this epidemic a Public Health Emergency of International Concern (PHEIC)^8^. This, along with other recent outbreaks of EBOV and SUDV, accelerated the development of EVD vaccines^4,9,10^.

A key antigenic target for EVD vaccines is the surface viral glycoprotein (GP) that mediates host cell attachment and fusion of the viral membrane and host endosomal membrane^11,12^. Dozens of EVD vaccines targeting GP progressed into clinical testing between 2014 and 2016^13^, resulting in two vaccines receiving regulatory approval^14^. A single dose vesicular stomatitis virus-vectored EBOV vaccine rVSV-ZEBOV (Ervebo®) was approved in the United States (US), European Union (EU), and several African countries. Later, a heterologous prime-boost regimen was approved in the EU: adenovirus serotype 26-vectored EBOV vaccine Ad26.ZEBOV (Zabdeno®) boosted eight weeks later by a modified vaccinia virus Ankara quadrivalent filovirus MVA-BN-Filo vaccine (Mvabea®)^14,15^. Both vaccine regimens were used during the 2018-2020 Democratic Republic of the Congo (DRC) outbreak, the second largest EBOV outbreak with 3,323 confirmed cases of EVD^16–18^. While rVSV-ZEBOV has been utilized for pre-exposure prophylaxis for frontline workers during Ebola outbreaks^19–22^, neither vaccine has been widely utilized for routine vaccinations in endemic regions for EVD^23^.

In response to the 2014–2016 outbreak, we conducted two parallel clinical trials of a monovalent or bivalent chimpanzee adenovirus vector 3 (cAd3) Ebola GP vaccine prime followed by an MVA-vectored EBOV GP boost in healthy adults in the United States (Trial US) and Uganda (Trial UG). The primary objectives of both trials were determination of the safety and tolerability profile of the vaccines. An ideal EVD vaccine would induce broad, rapid, and durable immunity after a single injection, with flexible and optional boosting. Vaccine multivalency could be advantageous for an EVD vaccine due to the multiple *Ebolavirus* species but might also reduce the immune response to each individual viral antigen. Therefore, we explored the relative immunogenicity of a monovalent (cAd3-EBOZ) versus a bivalent (cAd3-EBO) vaccine in both clinical trials. Furthermore, sporadic and unpredictable EVD outbreaks emphasize the need for a vaccine with flexible deployment options. Vaccine prime-boost intervals can impact immune responses, including a positive correlation between the interval length and maximum post-boost antibody titers^24^, although the long-term impact on humoral immunity is less clear^25,26^. Therefore, as an exploratory objective of Trial US, we investigated the impact of different prime-boost intervals on immunogenicity. Due to the similarities between Trial US and Trial UG, they have been combined in this report.

## Results

### Study population

**Fig. 1:**
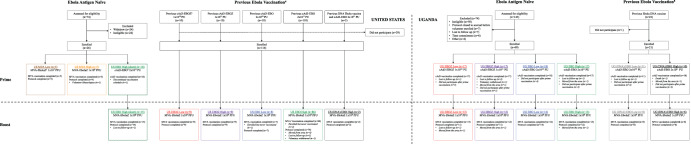
Consort diagram. ^1^MVA-EbolaZ (MVA): recombinant modified vaccinia virus Ankara Zaire ebolavirus (EBOV) vaccine^2^; cAd3-EBO: bivalent Zaire ebolavirus (EBOV) and Sudan ebolavirus (SUDV) chimpanzee adenovirus 3 (cAd3) vectored vaccine^3^, cAd3-EBOZ: monovalent Zaire ebolavirus (EBOV) chimpanzee adenovirus 3 (cAd3) vectored vaccine^4^; Previous *Ebolavirus* Vaccination: study participants previously participated in NCT02231866^5^; Previous *Ebolavirus* Vaccination: study participants previously participated in NCT00997607.

Between April 27 and November 20, 2015, 140 participants were enrolled into Trial US (Fig. 1), including 70 (50%) males and 70 (50%) females with the mean age of 38 years (range: 20–66). Among the participants, 26 were EVD vaccine-naïve who received either MVA-EbolaZ alone (*n* = 10) or a cAd3-Ebola vaccine (*n* = 16) followed by MVA-EbolaZ (*n* = 15). The remaining 114 participants were previously vaccinated with a cAd3-Ebola vaccine at least 12 weeks prior to enrollment and received only MVA-EbolaZ in Trial US. Prior vaccinees included nine EBOZ Low, nine EBOZ High, eight EBO Low, and 88 EBO High recipients (NCT02231866). Two of the latter had received a DNA Ebola vaccine (NCT00605514) five years prior to EBO High vaccination. A total of 12 participants (9%) discontinued the study after receipt of MVA-EbolaZ due to moving away from the area (*n* = 7, 5%), being lost to follow-up (*n* = 3, 2%), withdrawal (*n* = 1, 1%), or due to an unrelated illness/injury (*n* = 1, 1%). One participant (1%) received cAd3-EBO but not MVA-EbolaZ boost after the PI determined it was in the best interest of the participant. Complete Trial US demographic information is contained in Supplementary Table 2.

Between February 16, 2015, and April 10, 2015, 90 participants were enrolled into Trial UG (Fig. 1), including 75 (83%) males and 15 (17%) females, with the mean age of 30 years (range: 19-48). All study participants received a single cAd3-Ebola vaccine followed by MVA-EbolaZ. Among the participants, 69 were EVD vaccine-naïve. The remaining 21 had previously received a DNA Ebola vaccine (NCT00997607). 66 participants who completed at least 36 weeks of follow-up after cAd3-Ebola vaccination were enrolled to receive the MVA-EbolaZ boost. After cAd3-Ebola vaccination, a total of ten participants (11%) were discontinued from the study due to being lost to follow up (*n* = 5, 6%), moving from the area (*n* = 3, 3%), voluntary withdrawal (*n* = 1, 1%) and unrelated death (*n* = 1, 1%). Fourteen participants (16%) elected not to receive a boost. After MVA-EbolaZ vaccination, a total of four participants (4%) were discontinued from the study due to moving from the area (*n* = 3, 5%) or being lost to follow-up (*n* = 1, 2%). Complete Trial UG demographic information is listed in Supplementary Table 3.

### Safety

In both trials, the cAd3-Ebola and MVA-EbolaZ vaccines were safe when administered alone or in a prime-boost regimen. No SAEs related to study products were recorded. In Trial US, the most frequent study-product related AEs were neutropenia (*n* = 13, 9.5%), lymphopenia (*n* = 12, 8.8%), and leukopenia (*n* = 10, 7.3%). A full list of Trial US AEs and their duration is in Supplementary Table 4. In Trial UG, the most frequently reported AEs assessed as related to the product were mild leukopenia (n = 10, 11.1%) or mild to moderate neutropenia (*n* = 7, 7.8%). A full list of Trial UG AEs and their duration is in Supplementary Table 5. All abovementioned AEs resolved without sequelae.

**Fig. 2:**
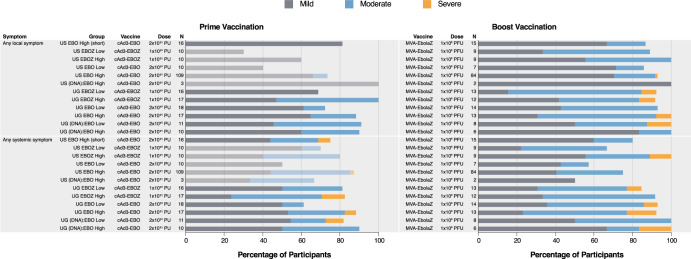
Solicited reactogenicity. Percent of participants (*x* axis) reporting solicited local or systemic symptoms by vaccine group (*y* axis) in the seven days following each vaccination. For symptoms persisting more than one day, a single count per person at the maximum severity of the symptom was used for the figure. Saturated bars are from the herein reported trials, faded bars are results from a previous trial (NCT02231866) and have been partially reported^28,32^. Detailed reactogenicity data is presented in Supplementary Fig. 1 (Trial US) and 2 (Trial UG).

Solicited local and systemic reactogenicity was mostly mild to moderate in severity (Fig. 2, Supplementary Figs. S1, S2). Most solicited symptoms exhibited dose-dependency: reports occurred with greater incidence in the higher dose groups than comparable lower dose groups. In Trial US, two participants developed severe transient fever after receiving either a cAd3-Ebola vaccine (*n* = 1, 6.3%) or MVA-EbolaZ (*n* = 1, 0.7%), and one participant experienced transient severe injection site pain after MVA-EbolaZ (*n* = 1, 0.7%; Supplementary Fig. S1). In Trial UG, five participants developed severe fever after cAd3-Ebola (*n* = 3, 3.4%) or MVA-EbolaZ (*n* = 2, 3.0%) and three participants developed transient severe injection site pain after MVA-EbolaZ (*n* = 3, 4.5%; Supplementary Fig. S2). Additional solicited severe systemic events in Trial UG included isolated cases of severe malaise or myalgia after receiving cAd3-Ebola vaccines (1.1% each). After MVA-EbolaZ boost, two severe headaches (3.0%) and single reports (1.5%) of severe injection site swelling, chills, malaise, and myalgia were reported. All symptoms were transient and resolved without sequelae.

### Antibody Response to cAd3-Ebola vaccines

**Fig. 3:**
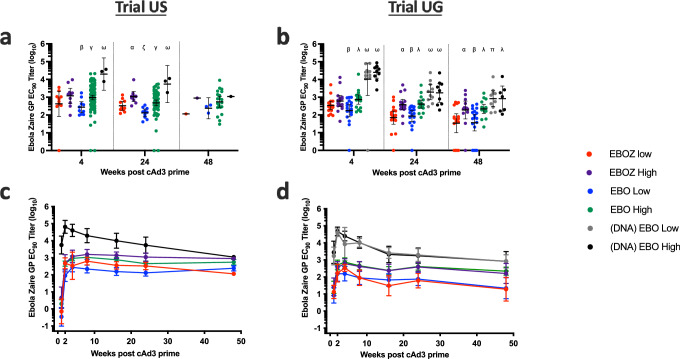
Antibody responses to the cAd3-Ebola vaccines followed similar dose-dependence and kinetics in the Trial US and Trial UG participants. Baseline-subtracted antibody titers following cAd3-EBOZ or cAd3-EBO vaccination in the US and UG trial participants as assessed by Ebola Zaire GP ELISA. Data expressed as EC_90_ titers. In (**a**, **b**) titers from weeks 4, 24 and 48 post-cAd3 Ebola vaccination are shown as dot plots overlaid with a line at the geometric mean titer (GMT). Titers were compared between groups from the same country and at the same time using Wilcoxon rank-sum test. Statistical significance is noted as follows: α: significant difference from EBOZ Low, β: Significant difference from EBOZ High, γ: Significant difference from EBO Low, λ: Significant difference from all Low, ζ: Significant difference from all EBOZ, π: significant difference from all Low and EBO High, ω: Significant difference from all non-DNA-primed. The *n* participants for each comparison and the *p* values are listed in Supplementary Tables 6 and 7. Results from (**a**, **c**) are from a previous clinical trial (NCT02231866), and the results have been partially reported^28,32^. Low *n* participants in (**a**) at week 48 are due to recruitment of participants into the subsequent Trial US to receive MVA vaccine (NCT02408913). In (**c**, **d**), durability of the vaccine-induced antibody titers are expressed as GMTs. Throughout figure, error bars indicate 95% CIs.

To evaluate immune responses to the cAd3-Ebola vaccines, we measured EBOZ GP-specific IgG antibodies by ELISA. Average baseline-subtracted antibody titers increased above a titer of 100 EC_90_ in all groups in both US and UG trial participants by the four-week secondary endpoint (Fig. 3a, b). Additional time points after vaccination were evaluated in exploratory analyses. A rapid and robust recall response to the cAd3-EBO vaccine was observed in (DNA): EBO participants (*n* = 24: 3 in US, 21 in UG), peaking at two weeks regardless of the dose (Fig. 3c, d). The titers in (DNA): EBO participants remained significantly greater at four weeks and 24 weeks than in the cAd3-Ebola primed groups (Fig. 3a, b). In participants without prior DNA Ebola vaccination, titers peaked by four to eight weeks after cAd3-Ebola vaccination. Titers remained significantly increased over baseline for all dose groups of *n* > 1 (group details in Supplementary Table 8) at 48 weeks after cAd3 vaccination (For Trial US, EBO low: *p* = 0.0079; EBO high: *p* < 0.0001. For Trial UG, EBOZ low: *p* = 0.0152; EBOZ high: p = 0.0003; EBO low: *p* = 0.0017; EBO high: *p* < 0.0001; (DNA) EBO low: *p* = 0.0014; (DNA) EBO high: *p* = 0.1136).

Among previously naïve recipients who received a cAd3-Ebola vaccine, the titer in EBO High was greater than the titer in EBO Low in both Trial US and Trial UG participants (Fig. 3a, b). Similarly, EBOZ High outperformed EBOZ Low, a difference that was statistically significant by 24 weeks post vaccination (Supplementary Table 6, 7). In UG participants, there was no difference in antibody titers between EBO and EBOZ at equivalent doses (Fig. 3b). However, in the US participants, titers elicited by EBO Low were significantly lower than those elicited by EBOZ Low at 24 weeks after vaccination (Supplementary Table 6). The impact of vaccine valency on antibody titers is apparent when viewed longitudinally, as the titers elicited by vaccines of equivalent dose overlapped in UG, but in the US the graphs were interleaved, with EBO titers lower than EBOZ titers (Fig. 3c). When we compared vaccine valency in an ad-hoc analysis by two-sample *t* test, recipients of either dose of cAd3-EBO had significantly lower antibody titers at week 24 compared to recipients of the equivalent doses of EBOZ in Trial US (*p* = 0.013 between low doses *p* = 0.022 between high doses) but not in Trial UG.

In a subset of participants from Trial US, the antibody response to SUDV GP was assessed by ELISA (Supplementary Fig. 3a). As expected, the SUDV GP-specific antibody titers were significantly higher in participants receiving the bivalent vaccine than the monovalent vaccine, and the titers among the bivalent vaccine recipients were dose-dependent.

### T cell responses to cAd3-Ebola vaccines

**Fig. 4:**
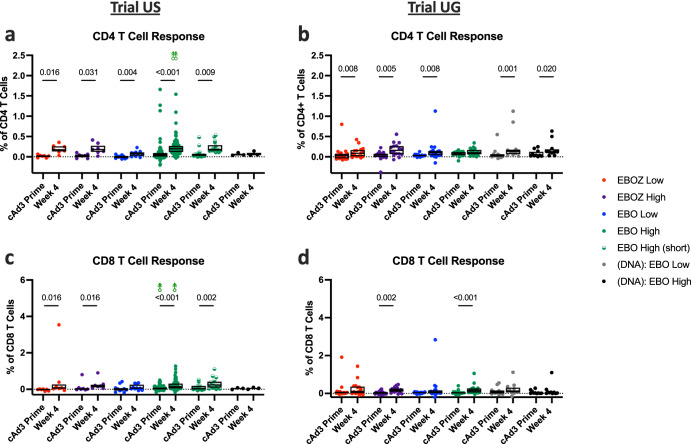
cAd3-Ebola vaccination increased the proportion of CD4+ and CD8 + T cells responding to Zaire epitopes by week 4. Percentage of memory background-subtracted CD4 (**a**, **b**) and CD8 (**c**, **d**) T cells responding to ex vivo stimulation with production of any tested cytokine at baseline and four weeks post cAd3-Ebola vaccination in Trial US (**a**, **c**) and Trial UG (**b**, **d**) participants. Box plots denote median with upper and lower quartiles of each group. Dotted line indicates background cytokine production in the absence of stimulation. Each ⥉ in (**a**, **c**) indicates a single data point greater than the extent of the *y* axis : one participant displayed high baseline frequencies between 20 and 25% of GP-reactive memory CD4 and CD8 T cells, that diminished to less than 1% after vaccination; four weeks after vaccination, GP reactivity reached 2.7% of memory CD4 T cells in one individual and 6.9% of memory CD8 T cells in a separate individual. For each group, titers were compared between baseline and week 4 using the Wilcoxon rank-sum test, and statistical significance is reported above each group for which *p* < 0.05. Results from (**a**, **c**) are from a previous clinical trial (NCT02231866), and the results have been partially reported^28,32^.

To evaluate cell-mediated immune responses, we quantified CD4 and CD8 memory T cell responses to peptides derived from the EBOZ GP antigen by flow cytometry. CD4 and CD8 memory T cell responses were detected at four weeks after vaccination with a cAd3-Ebola vaccine (Fig. 4). The most pronounced increase in the percentage of CD4 T cells from baseline to four weeks post prime was observed in Trial US study participants who received EBO High (*p* < 0.001, Fig. 4a). Interestingly, no differences were observed in the corresponding group in Trial UG (Fig. 4b). Significant increases in CD4 T cells were also found in EBOZ Low (US: p = 0.016; UG: *p* = 0.008) and EBOZ High dose groups (US: *p* = 0.031; UG: *p* = 0.005), in the EBO Low recipients (US: *p* = 0.004; UG: *p* = 0.008), and in Trial UG (DNA):EBO groups ((DNA):EBO Low: *p* = 0.001; (DNA):EBO High: *p* = 0.020).

Similar trends were observed for EBOZ GP-specific CD8 memory T cell responses in participants from both trials (Fig. 4 c, d). Significant increases were observed in EBO High (*p* < 0·001 for both US and UG) and in EBOZ High dose groups (US: *p* = 0.0016, UG: *p* = 0.002). However, an increase in the percentage of CD8 T cells in EBOZ Low groups (*p* = 0·016) was seen only in Trial US. No statistically significant differences were observed for the DNA Ebola groups.

We evaluated the subset of participants from Trial US for SUDV GP-specific T cell responses to an overlapping peptide pool from the SUDV GP vaccine insert by flow cytometry. SUDV GP-specific CD4 T cells were significantly increased at week 4 over baseline (*p* ≤ 0.005) by vaccination with cAd3-EBO, but not cAd3-EBOZ (Supplementary Fig. 3b). Only the higher dose of the cAd3-EBO vaccine elicited a significant week 4 increase (*p* ≤ 0.005) in SUDV GP-specific CD8 T cells.

### Immune response to MVA alone in Ebola-naïve participants

MVA-EbolaZ was initially evaluated alone in Trial US because safety of this product in humans was yet to be established when the trial began. Unlike the cAd3-Ebola vaccine-primed participants, and consistent with previous trials of MVA-vectored vaccines^27^, modest immunogenicity was elicited by MVA-EbolaZ alone (Supplementary Fig. 4).

### Antibody responses to MVA-EbolaZ administered as a boost to cAd3-Ebola

**Fig. 5:**
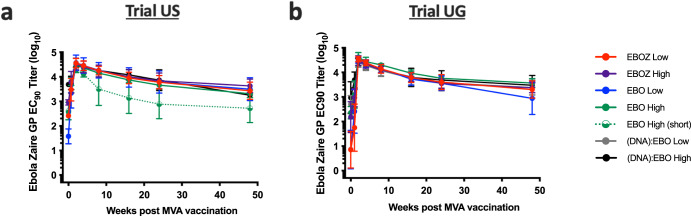
MVA-EbolaZ boosted EBOZ GP-specific antibody titers in participants with previous cAd3-Ebola vaccine exposure to similar magnitudes in Trial US and Trial UG. Baseline (pre-cAd3)-subtracted antibody titers (*y* axis) in the weeks (*x* axis) following MVA-EbolaZ vaccination in the US (**a**) and UG (**b**) participants as assessed by Ebola Zaire GP ELISA. Data expressed as EC_90_ titers with 95% confidence intervals (CIs) indicated by error bars. The EBO High (short) group is the Trial US group with a prime-boost interval of 6–11 weeks.

In both Trial US and Trial UG, when administered after cAd3-Ebola, MVA-EbolaZ boosted the EBOZ GP-specific antibody responses to a uniformly high titer, irrespective of the cAd3-Ebola priming vaccine or whether they were previously exposed to a DNA Ebola vaccine (Fig. 5). Baseline-subtracted (pre-cAd3) responses peaked at two weeks after the boost and were maintained at an average titer of over 1000 EC_90_ out to 48 weeks post MVA-EbolaZ. The similar peak titers across all groups indicate that MVA-EbolaZ is an effective boost for cAd3-exposed participants regardless of the dose, valency, or number of previous Ebola vaccines.

### T cell responses to MVA-EbolaZ administered as a boost to cAd3-Ebola

**Fig. 6:**
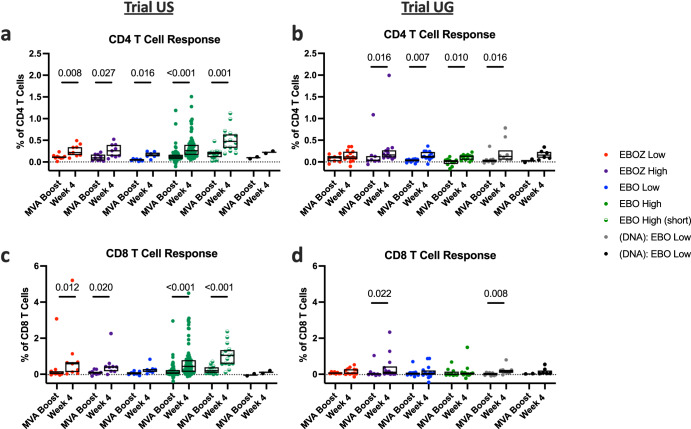
MVA-EbolaZ boost increased proportion of CD4+ and CD8 + T cells responding to Zaire epitopes by week 4. Percentage of memory background-subtracted CD4 (**a**, **b**) and CD8 (**c**, **d**) T cells responding to ex vivo stimulation with production of any tested cytokine at baseline and four weeks post-MVA vaccination in US (**a**, **c**) and UG (**b**, **d**) participants. Box plots denote median with upper and lower quartiles of each group. Dotted line indicates background cytokine production in the absence of stimulation. For each group, titers were compared between baseline and week 4 using the Wilcoxon rank-sum test, and statistical significance is reported above each group for which *p* < 0.05.

We also measured cell-mediated immune responses following boost vaccination with MVA-EbolaZ by intracellular cytokine staining after stimulation with a pool of overlapping peptides from the EBOV GP vaccine insert. Most groups in both Trial US and Trial UG displayed significant increases in antigen specific CD4 memory T cells from baseline to four weeks (*p* < 0.05) after the boost (Fig. 6a, b). Significant increases in CD8 T cell antigen specific immune responses were observed in most Trial US groups (*p* < 0.05) following MVA-EbolaZ boost (Fig. 6c). In Trial UG, differences at four weeks after the boost were seen only in two groups: EBOZ High (*p* = 0.022) and (DNA): EBO Low (*p* = 0.008) (Fig. 6d).

### Effect of prime-boost interval on antibody titers and T cell responses

**Fig. 7:**
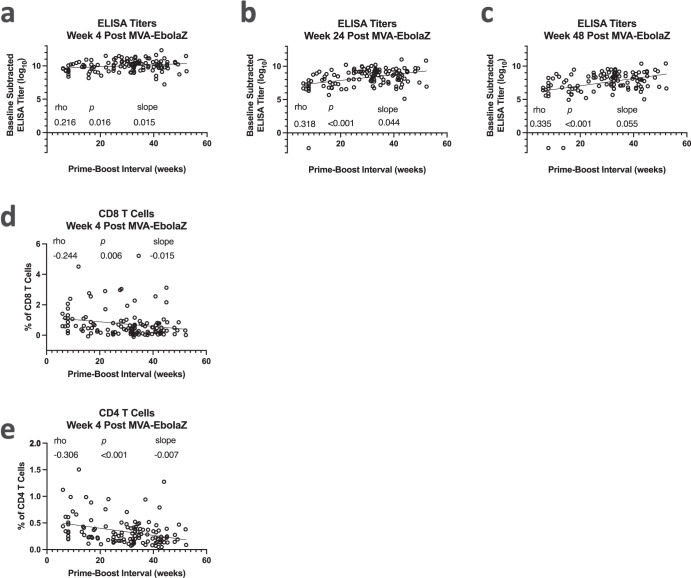
Memory T cell and antibody responses are differentially dependent on the prime-boost interval in Trial US. Log_10_ transformed EC_90_ titers (**a**–**c**) or percent of memory T cells responding to stimulation with any tested cytokine production (**d**, **e**) of samples from US participants (*y* axis) are plotted against the interval in weeks between the participant’s cAd3-Ebola prime and MVA-EbolaZ boost vaccinations (*x* axis). Spearman correlations *p*, and rho are included for each comparison. A simple linear regression line and its slope is included for reference. For antibody titers, correlations were performed comparing the prime-boost interval against week four (**a**), 24, (**b**) and 48 (**c**) EBOZ GP-specific antibody titers after the boost, whereas for T cells the comparison used CD4 (**d**) and CD8 (**e**) T cell percentages from week 4 only.

An exploratory objective of Trial US was to evaluate the immunogenicity elicited by different intervals between the cAd3-Ebola vaccine prime and the MVA-EbolaZ boost. Participants who received EBO High after a 6–11-week prime boost interval (US EBO High (short)) had a steeper decline in titers than the US EBO High population that had prime-boost intervals from 12 to 52 weeks, (Fig. 5a). These results suggest that a short prime-boost interval was suboptimal for antibody durability with these vaccines. To expand upon this observation, we assessed the correlation of antibody titers or T cell responses versus the prime-boost intervals (Fig. 7). Indeed, there was a positive correlation of antibody titers with increasing prime-boost intervals. The correlation at week 4 post-MVA-EbolaZ was negligible (Fig. 7a), consistent with the similar peak antibody recall responses (Fig. 5). However, with increasing time after boost, the effect of prime-boost interval increased, such that the correlation was observed at 24 weeks (Fig. 7b) and was the strongest at 48 weeks post MVA-EbolaZ boost (Fig. 7c). In contrast, the percentage of CD4 and CD8 memory T cells at four weeks post MVA-EbolaZ boost was negatively associated with the prime-boost interval. The effect was negligible for CD8 T cells (Fig. 7d) but stronger for CD4 T cells (Fig. 7e).

### Adenoviral vector immunogenicity

As a secondary objective of Trial UG and exploratory objective of Trial US, we assayed the neutralizing antibody responses to the cAd3 vector. This GP antigen-agnostic assay was performed to determine the impact of pre-existing cAd3-specific antibodies on the immunogenicity of the virus-vectored vaccines. cAd3-neutralizing titers after cAd3-Ebola vaccination have been previously published for Trial US participants^28^, except for the EBO High (short) cohort, which is presented alongside the results from the UG cohort (Supplementary Fig. 5). As observed in previous cAd3 vector trials^28^, the cAd3 vector elicited neutralizing antibody responses against all cAd3-Ebola vaccine formulations.

## Discussion

This report summarizes the findings of two phase 1/1b clinical trials to determine the safety and immunogenicity of cAd3-Ebola vaccine primes followed by an MVA-EbolaZ boost. The trials were conducted in the US and Uganda during the 2014–2016 PHEIC Ebola epidemic, when no licensed Ebola vaccines were available. The three vaccines (cAd3-EBO, cAd3-EBOZ, and MVA-EbolaZ) tested in these trials were safe and tolerable alone and in combination. The prime-boost regimen induced EBOV and SUDV GP-specific antibody responses and cell-mediated responses, which are associated with protection against viral infection and durability of protection, respectively^29,30^. Humoral responses were sustained for at least 48 weeks after both cAd3-Ebola prime and MVA-EbolaZ boost, with the boost increasing antibody titers in all groups to a similar absolute level by two weeks. Both the prime-boost interval and the valency of the priming vaccine had impacts on humoral and cellular immune responses. These studies support and expand the body of evidence indicating safety and immunogenicity of the cAd3-Ebola prime, MVA-EbolaZ boost regimen, while revealing insights about vaccine valency and prime-boost interval which will inform future EVD vaccine development.

All three vaccines evaluated were safe and tolerable. Most vaccine-elicited symptoms were mild to moderate in severity, and most unsolicited AEs related to the vaccine were mild and involved perturbations to blood counts with no clinical sequelae. In naïve participants, the cAd3-Ebola vaccines were more reactogenic than MVA-EbolaZ, in a dose-dependent manner. Higher rates of MVA-EbolaZ reactogenicity were observed after MVA-EbolaZ was administered as a boost. These results are consistent with the relative immunogenicity of the vaccines: MVA-EbolaZ generated lower antibody titers than the cAd3-Ebola vaccines in naïve participants, and the humoral response to cAd3-Ebola vaccines alone was dose-dependent. Importantly, no arthralgia was reported in Trial US, and cases of arthralgia in Trial UG had a median duration of 1 day (interquartile range, 0-2) and were never severe, compared to the 8 or 18 day median durations reported in early phase rVSV-ZEBOV trials which included grade 3 arthralgias^20,31^. Also, no virus-filled skin vesicles occurred after either cAd3-Ebola or MVA-EbolaZ vaccination; this was a safety concern reported in early phase rVSV-ZEBOV clinical trials^20,31^. The data presented add to the body of evidence^19,24,27,28,32,33^ that the cAd3-Ebola and MVA-EbolaZ vaccines are safe and tolerable in humans.

The exact correlates of protection against EVD in humans have yet to be established; however, the presence of anti-GP antibodies is associated with protection against viral infection^29,30^. Both cellular and humoral responses are important for protection of nonhuman primates from EBOV^29,30^. Studies performed in macaques demonstrated that protective antibody responses to EBOV predominantly target GP^34^, and that induction of antigen-specific effector and memory CD8 T cells might also be required to achieve durable protection^29^. T-cell dependent B cell activation is critical for Ebola GP-specific IgG production^30^. These results suggest that both humoral and cellular immunity are integral to the response to EBOV.

In the presented studies, cAd3-Ebola vaccines elicited robust and durable humoral immune responses, which were enhanced by the MVA-EbolaZ boost. Consistent with previous trials^28,32^, the cAd3-Ebola vaccines can elicit long-lasting humoral immunity against EBOV with one vaccination dose^28^, which would be advantageous to rapidly produce antibody titers in the face of an outbreak. The stability of EBOZ GP-specific antibody titers in both US and UG cohorts for up to 48 weeks prior to boosting suggests a cAd3-Ebola vaccine could be potentially administered far in advance of an outbreak without significant loss of protection. In general, both populations had similar immune responses to cAd3-Ebola vaccines. The approved Ebola vaccines have demonstrated antibody titers lasting at least two (Ad26.ZEBOV + MVA-BN-Filo) or three (rVSV-ZEBOV) years post vaccination^14,35^. Interestingly, participants with prior receipt of a DNA Ebola vaccine displayed a rapid recall of the humoral response following cAd3-Ebola vaccination, despite the minimum of five years’ interval between vaccines, indicating a possible use for DNA priming of front-line workers that could be boosted quickly in outbreak responses. MVA-EbolaZ administered at intervals ranging from 6 to 52 weeks post-vaccination successfully boosted the cAd3-Ebola vaccine-primed response. The MVA-EbolaZ boost uniformly increased EBOZ GP-specific antibody titers approximately five-5,000-fold, and the boosted titers were durable for at least 48 weeks, irrespective of prime dose or valency. Given the dose-dependent reactogenicity of the cAd3-Ebola vaccines, the results suggest that the prime dose could be strategically lowered in the interest of dose-sparing if a high rate of booster vaccine compliance is expected.

Prime-boost interval flexibility would benefit Ebola-endemic areas, allowing effective boosting of a primed population upon detection of an outbreak’s index cases. Due to the 56-day delay between recommended doses of Ad26.ZEBOV and MVA-BN-Filo, that regimen is not recommended for those at high risk of Ebola infection^35^. That the vast majority of participants in both Trial US and UG had titers well above baseline levels as early as week 2 suggests that the cAd3-Ebola vaccines may be valuable in a rapid response scenario, without necessitating the MVA-EbolaZ boost before potential infectious exposure. In situations where prime-boost flexibility is less essential, an interval favoring the most protective immune outcome might be utilized. The cAd3-Ebola, MVA-Ebola Z prime-boost interval was observed to differentially affect the magnitude of the humoral and cellular immune response. Longer prime-boost intervals favored a robust humoral boost and shorter intervals favored increased cellular memory responses, a pattern that has been reported previously^24,25,27^, lending greater weight to these findings^27^. Whether, and in what way, these differences are biologically meaningful remains to be determined.

Limitations of these trials include a higher percentage of men as compared to women in Trial UG, and the small number of study participants who completed all study visits post cAd3-Ebola prime in the US participants resulting from their enrollment to receive MVA-EbolaZ boost. These small phase 1 trials were designed to establish safety of the vaccines evaluated and were not powered to determine vaccine efficacy or correlates of protection. Finally, while the magnitude of the immune responses against SUDV GP were in a similar range to those against EBOV, they were only captured for a subset of participants who received the cAd3-Ebola vaccines in the US trial and should be evaluated further in future studies.

These parallel phase 1/1b clinical trials in the US and Uganda demonstrated safety and immunogenicity of the cAd3-EBO, cAd3-EBOZ, and MVA-EbolaZ vaccines alone and in combination. Importantly, the elicited antibody responses were durable up to 48 weeks following each vaccination. The MVA-EbolaZ vaccine generated a robust boosting effect to the Ebola-specific immune response, irrespective of prime dose or valency. While still robust, differences in the magnitude of the humoral and cellular immune responses depended on the MVA-EbolaZ prime-boost interval. The cAd3-based filovirus platform has been licensed for further development by the Sabin Vaccine Institute. The results of these clinical trials will inform future Ebola vaccine development and optimization.

## Methods

### Study design and participants

Two phase 1/1b open-label, randomized, dose-escalation clinical trials were conducted in parallel to evaluate the safety, tolerability, and immunogenicity of a prime-boost regimen in healthy adults with or without prior EVD vaccination in the US and Uganda. This regimen included priming with one of two cAd3-vectored vaccines (collectively referred to as cAd3-Ebola) followed by an MVA vector expressing EBOV GP (MVA-EbolaZ). The cAd3-Ebola prime was either monovalent, expressing the GP of EBOV (cAd3-EBOZ), or a bivalent cocktail of two cAd3 vectors (cAd3-EBO), each encoding either the GP of EBOV or SUDV. Trial US was reviewed and approved by the NIAID Institutional Review Board (IRB). Trial UG was approved by the infectious diseases IRB of the Uniformed Services of the Health Sciences (Bethesda, MD, USA), the research and ethics committee of Makerere University School of Public Health (Kampala, Uganda), and the Uganda National Council of Science and Technology (Kampala, Uganda). Both trials were conducted in compliance with International Council for Harmonisation (ICH) Good Clinical Practice (GCP) guidelines; all participants provided written informed consent prior to enrollment.

A phase 1/1b trial (NCT02408913) was conducted at three sites in the US (herein designated Trial US): Vaccine Research Center (VRC), National Institutes of Allergy and Infectious Diseases (NIAID), National Institutes of Health (NIH) Clinical Center, Bethesda, MD; Center for Vaccine Development and Global Health, University of Maryland School of Medicine, Baltimore, MD and The Hope Clinic of the Emory Vaccine Center, Emory University, Atlanta, GA. Eligible EVD vaccine-naïve study participants between the ages of 18–50 years old and with no clinically significant medical history were recruited from the Washington, D.C. metropolitan area (VRC site). Additionally, eligible study participants between the ages of 18–66 who had already received either the cAd3-EBOZ or cAd3-EBO vaccine in a previous clinical trial (NCT02231866) and completed at least 12 weeks of follow-up were offered to enroll into Trial US to receive MVA-EbolaZ vaccine (all three US sites). Two of the latter participants had also received a DNA Ebola vaccine in an earlier clinical trial (NCT00605514) before receipt of the cAd3-EBO vaccine in the previous trial (NCT02231866). A full list of inclusion/exclusion criteria can be found in the study protocol (Supplementary Materials).

A phase 1b trial (NCT02354404) was conducted at Makerere University-Walter Reed Project, Kampala, Uganda area (herein designated Trial UG). EVD vaccine-naïve study participants were healthy adults 18–65 years of age without clinically significant medical history. Exclusion criteria included history of severe adverse reactions to vaccines and receipt of investigational products or live vaccines 28 days prior to enrollment. Individuals who had received Ebola monovalent DNA or bivalent Ebola and Marburg DNA vaccines (DNA Ebola, collectively) in a previous clinical trial (NCT00997607) and completed at least 36 weeks of follow-up were also provisionally eligible for enrollment- additional details, including full inclusion/exclusion criteria, can be found in the study protocol (Supplementary Materials).

### Randomization

In Trial US, EVD vaccine-naïve participants were randomized 1:2 to receive 1 × 10^7^ PFU dose of MVA-EbolaZ or 2 × 10^11^ PU dose of cAd3-EBO (Fig. 1). The randomization obtained via computer-generated random numbers were provided to the study pharmacist by the protocol statistician. The 1 × 10^8^ PFU dose of MVA-EbolaZ was enrolled thereafter. All cAd3-Ebola-experienced participants received MVA-EbolaZ at a dose of 1 × 10^8^ PFU and were grouped based on their previous clinical trial history (NCT02231866). In Trial UG, EVD vaccine-naïve study participants were randomized by unblinded staff 1:1:1:1 to receive a single injection of cAd3-EBOZ at a dose of 1 × 10^10^ PU or 1 × 10^11^ PU or cAd3-EBO at a dose of 2 × 10^10^ PU or 2 × 10^11^ PU. Volunteers who previously received a DNA Ebola vaccine (NCT00997607) were randomized 1:1 to receive cAd3-EBO at 2 × 10^10^ PU or 2 × 10^11^ PU. Both Trial US and Trial UG were open-label and medical personnel were aware of treatment assignments.

### Vaccines

A recombinant replication-deficient cAd3 virus was used as a vector for two EVD vaccines manufactured at the VRC Vaccine Pilot Plant operated under contract by the Vaccine Clinical Materials Program (VCMP), Leidos Biomedical Research, Inc. in Frederick, MD. The monovalent cAd3-EBOZ vaccine encoded the wildtype GP from *Zaire ebolavirus* and was manufactured at 1 × 10^11^ PU/mL in formulation buffer. It was administered intramuscularly in a 1 mL volume at either 1 × 10^10^ PU (EBOZ Low) or 1 × 10^11^ PU (EBOZ High) doses. The bivalent cAd3-EBO vaccine consisted of 1:1 ratio of cAd3 vectors encoding the GPs from *Zaire ebolavirus* (EBOZ) Mayinga^28^ and *Sudan ebolavirus* (SUDV) Gulu strains and was manufactured at 2 × 10^11^ PU/mL in formulation buffer. It was administered intramuscularly in a 1 mL volume at 2 × 10^10^ PU (EBO Low) or 2 × 10^11^ PU (EBO High) doses. The shorthand term “cAd3-Ebola” is used to indicate administration of either cAd3-EBO or cAd3-EBOZ.

MVA-EbolaZ was manufactured at IDT Biologika GmbH, Dessau, Germany under contract to Advent S.r.l, Pomezia, Italy (a subsidiary of Okairos S.r.l.) and managed by VCMP. MVA-EbolaZ consisted of the attenuated Modified Vaccinia Ankara (MVA) vector modified to express GP from *Zaire ebolavirus* (EBOV). The vaccine was formulated at 3.2 × 10^8^ PFU/mL and was administered in a 0.3 mL volume for a final dose of 1 × 10^8^ PFU unless noted otherwise. All study vaccines were manufactured under Good Manufacturing Practices.

### Study procedures

In Trial US, to test the safety of MVA-EbolaZ alone or as a boost, EVD vaccine-naïve participants received MVA-EbolaZ at either 1 × 10^7^ PFU (MVA Low) or 1 × 10^8^ PFU (MVA High) (Fig. 1, Supplementary Table 1). A third group of naïve participants received a bivalent vaccine prime of cAd3-EBO at 2 × 10^11^ PU, followed by a boost of MVA-EbolaZ 6–11 weeks later (EBO High (short)). All participants with prior cAd3-Ebola vaccination (EBOZ Low/High or bivalent EBO Low/High (NCT02231866)) received MVA-EbolaZ 12-52 weeks following their cAd3-Ebola prime dose. A subset of three participants who received cAd3-EBO High in NCT02231866 had previously participated in a trial in 2008-2009, in which they were exposed to a DNA Ebola vaccine (NCT00605514). Two of these participants were also boosted with MVA-EbolaZ in Trial US and are designated (DNA): EBO High (Fig. 1).

In Trial UG, participants were primed with cAd3-EBO or cAd3-EBOZ at one of two doses (Fig. 1, Supplementary Table 1). Naïve participants were randomized into four groups to receive EBOZ Low, EBOZ High, EBO Low or EBO High. Participants previously vaccinated with DNA Ebola in 2009-2010 (NCT00997607) were randomized into two groups and given either EBO Low ((DNA): EBO Low) or EBO High ((DNA): EBO High). In total, 60–78% of cAd3-Ebola-primed participants per group who had completed at least 36 weeks of follow-up were successfully retained to receive the MVA-EbolaZ boost.

All study injections were administered intramuscularly into the deltoid muscle by needle and syringe. Safety monitoring included a 30-minute post-vaccination monitoring period and clinical and laboratory evaluations at protocol-specified follow-up visits. Participants reported solicited reactogenicity for the first week following each vaccination. Adverse events (AEs) were collected for the first 28 days after each vaccination, while serious adverse events (SAEs) and new chronic medical conditions were recorded throughout the trial.

Serum and peripheral blood mononuclear cell samples were collected at protocol-specified timepoints for immunogenicity analysis of vaccine-induced antibody and T cell responses.

### Outcomes

The primary objectives were to evaluate the safety and tolerability of cAd3-Ebola and MVA-EbolaZ vaccines when administered alone or as a prime-boost regimen. The secondary objectives were to investigate vaccine-induced antibody responses and T cell responses for each vaccine/dose combination, cAd3 neutralizing antibody titers, and the priming effect of a prior Ebola DNA vaccine exposure.

### Assessment of EBOLA EBOZ GP-Specific response by ELISA

EBOZ and SUDV GP-specific serum IgG antibody titers were assessed by enzyme-linked immunosorbent assay (ELISA) as previously described^36^, with the following modification: prepared lectin plates were incubated at 4 °C overnight with a transmembrane deleted form of the EBOZ GP Mayinga strain (Mayinga 1976, GenBank - U31033) or SUDV GP Gulu strain (Uganda 2000, GenBank – AAP88031). Serum samples were run in triplicate. Results were expressed as 90% effective concentration (EC_90_) titers, reciprocal serum dilution values that represent the dilution at which there is a 90% decrease in antigen binding activity. All post-vaccination titers have been baseline-subtracted from the matched pre-vaccination titer. Titers below the assay limit of detection were imputed to 0.1 for statistical analysis, but where individual data points are shown, below the limit of detection samples are graphed at log(0).

### Assessment of EBOZ GP-specific T cell responses by ICS assay

Vaccine-induced T cell responses were evaluated by a qualified intracellular cytokine staining (ICS) as previously described^37^. In this assay, cryopreserved peripheral-blood mononuclear cells (PBMCs) were stimulated in an antigen-specific manner with overlapping 15-mer peptide pools, overlapping by 11 amino acids, that matched either vaccine inserts for the EBOZ GP or SUDV Gulu GP to assess memory T cell responses at week 4 after immunization. Responding T cells were identified by production of IFN-γ, TNF-α, and/or IL-2 after peptide stimulation, corrected for background cytokine production in the absence of peptide stimulation. Memory CD4 and CD8 T cells were identified based on the expression of CD45RA and CD28. Percentages of background-subtracted memory CD4 and CD8 T cells were reported for each sample, assayed without technical replicates.

### cAd3 serologic assessment

Adenovirus serum neutralization assays were performed to assess neutralizing antibody titers at baseline and at 4 weeks post-vaccination against the cAd3 vector as previously described^38^. Briefly, threefold serially diluted participant serum were added to plates without technical replicates before addition of A549 cells and E1-deleted replication-incompetent recombinant cAd3-luciferase reporter viruses for a final in-well dilution range from 1:12 to 1:8748. Reciprocal antibody titers are reported as the 90% inhibitory concentration (IC_90_; the titer at which 90% of virus infectivity is inhibited).

### Statistical analysis

All participants were monitored for safety and reactogenicity. Participants were included in analyses for vaccine-induced antibody and T cell responses after each vaccination received; participants were not included in post-boost immunogenicity analyses unless boosted. Primary sample size calculations for safety were expressed in terms of the ability to detect SAEs. For Trial US, within each group of 10 participants, the probability of observing at least one SAE is at least 90% if the true rate of at least one SAE is 0·206 and over 90% probability to observe no SAE if the true rate is no more than 0·01; for participants who received MVA-EbolaZ, there is over 90% chance to observe at least one SAE if the true rate is no less than 0·001 and over 90% chance of observing no SAE if the true rate is no more than 0·023 given the number of vaccinees is 100. For Trial UG, Clopper-Pearson 95% confidence intervals (CIs) for the true rate of at least one event for a sample size of 15 were determined; if one safety event occurred, then the upper limit of 95% CI would be 32·0 and the lower limit 0·2.

For humoral immune responses, comparisons between the groups at weeks 4, 24, and 48 post-prime vaccination were performed by Wilcoxon rank-sum test. Vaccine-induced antibody responses were reported as geometric mean of baseline subtracted EBOZ or SUDV GP-specific EC_90_ titers with 95% CIs at each study visit post-prime and post-boost. Paired *t* test was performed to compare log-transformed baseline titers with log-transformed titers at week 48 post-infection.

For cell-mediated immune responses, background-subtracted percentages of EBOZ- or SUDV-specific CD4 and CD8 memory T cells were compared between baseline and four weeks after prime and between baseline (at the time of the boost) and four weeks post-boost using Wilcoxon signed rank test; median and interquartile ranges were also reported. Correlations in the exploratory analyses were computed using Spearman’s rank order correlations test to understand the impact of the prime-boost interval on memory T cell and antibody responses in the US cohort. To assess if the antibody responses against cAd3-Ebola vaccines differed by vaccine valency, a two-sample *t* test was applied within each dose group. Only groups EBO Low and EBO High from Trial US were included in the latter analysis due to paucity of data from the other Trial US groups.

Geometric Mean titers and 95% CIs were calculated for neutralizing antibody responses to the cAd3 vector. All analyses were two-sided and performed using R software (version 4.1.3).

### Reporting summary

Further information on research design is available in the Nature Research Reporting Summary linked to this article.

## Supplementary information

Supplementary Material

REPORTING SUMMARY

## Data Availability

The datasets and any unique materials used and/or analyzed during the current study available from the corresponding author on a reasonable request. The study protocols, statistical analysis plans, and informed consent forms are available in the Supplementary Appendices.
