# Supplementary material for: Heterologous cAd3-Ebola and MVA-EbolaZ vaccines are safe and immunogenic in US and Uganda phase 1/1b trials
Source: NPJ Vaccines. 2024 Mar 29;9:67. doi: 10.1038/s41541-024-00833-z (PMC10980745; doi:10.1038/s41541-024-00833-z)
Supplement: Supplementary file 1 — Supplementary Material [file 41541_2024_833_MOESM1_ESM.pdf]

Supplementary Materials

Supplementary Figures **2**
Supplementary Figure 1: Solicited reactogenicity detailed from the US trial. 2
Supplementary Figure 2: Solicited reactogenicity detailed from the UG trial. 4
Supplementary Figure 3: cAd3-Ebola vaccines elicit SUDV GP-specific immune responses by four weeks after vaccination........ 5
Supplementary Figure 4: MVA-EbolaZ alone elicited increased antibody titers but not T cell responses in the US trial. 7
Supplementary Figure 5: cAd3 vector-specific responses were observed after all cAd3 vaccinations. 7

Supplementary Tables **8**
Supplementary Table 1: Group Designations 8
Supplementary Table 2: US Demographics 9
Supplementary Table 3: UG Demographics........ 10
Supplementary Table 4: Trial US Adverse Events (AEs) assessed as related to study product. 11
Supplementary Table 5: Trial UG Adverse Events (AEs) assessed as related to study product........ 12
Supplementary Table 6: Results of Wilcoxon rank-sum test comparing EBOZ-specific IgG titers elicited by the different vaccination regimens in Trial US. 13
Supplementary Table 7: Results of Wilcoxon rank-sum test comparing EBOZ-specific IgG titers elicited by the different vaccination regimens in Trial UG. 14
Supplementary Table 8: Sample numbers for Ebola Zaire-specific immunogenicity assays........ 15

Supplementary References **16**

Trial US Protocol **17**

Trial UG Protocol **90**

Supplementary Figures

Supplementary Figure 1: Solicited reactogenicity detailed from the US trial.

Percent of participants (x axis) reporting solicited local or systemic symptoms by vaccine group (y axis) in the seven days following (A) MVA-EbolaZ only or (B, *see next page*) cAd3-Ebola and MVA-EbolaZ in a prime-boost regimen. For symptoms persisting more than one day, a single count per person at the maximum severity of the symptom was used for the figure. Saturated bars are from the herein reported trials, faded bars are results from a previous trial (NCT02231866) and have been partially reported previously.[1,2]

a

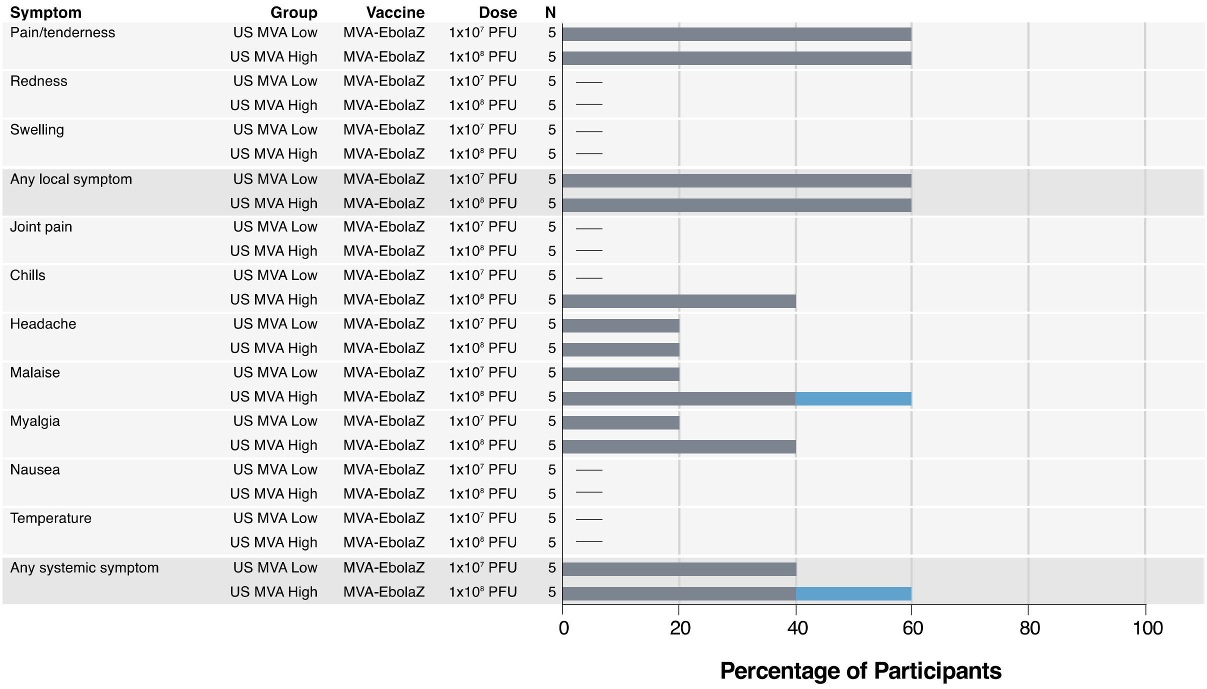

b

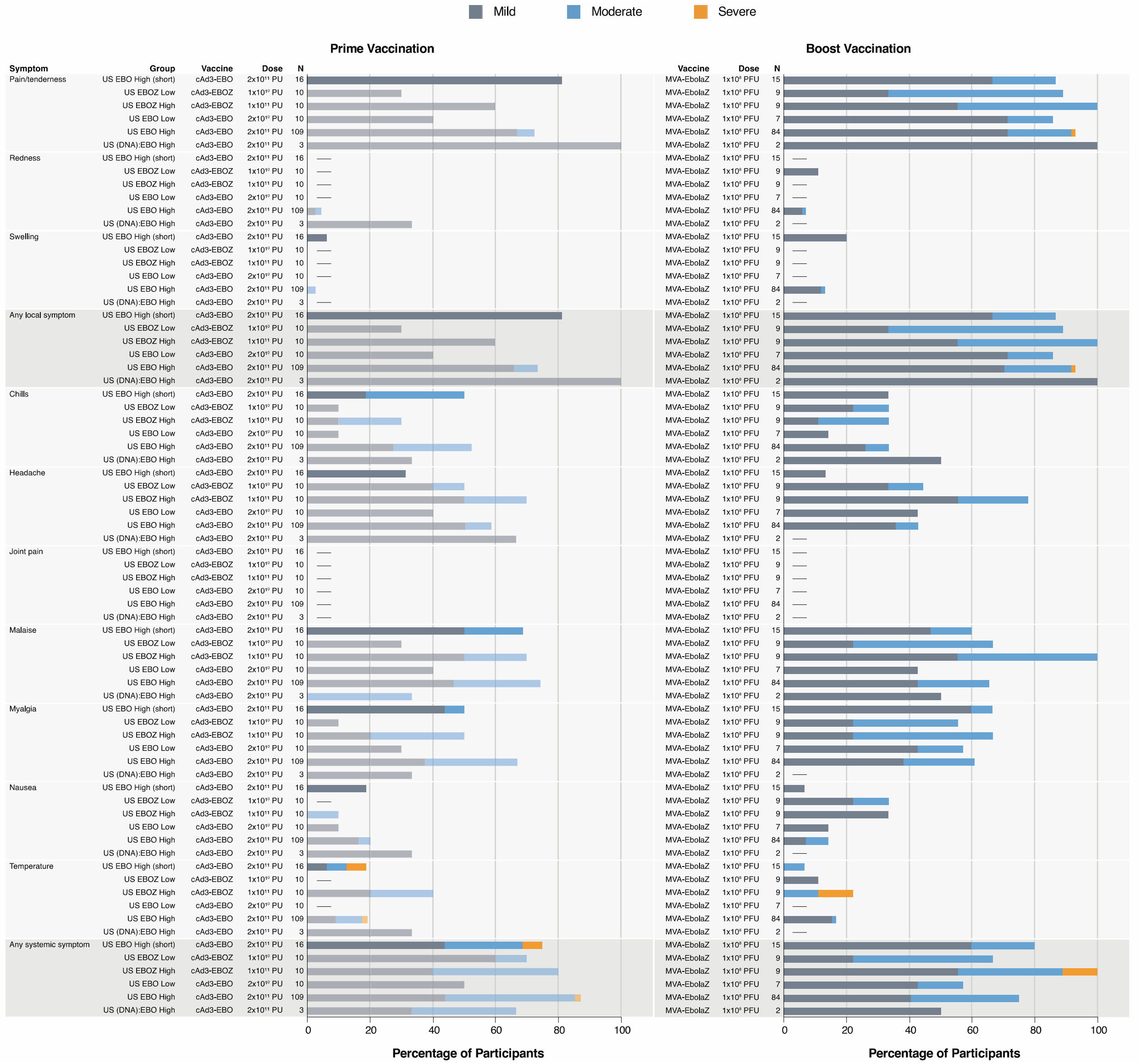

Mild
Moderate
Severe
Prime Vaccination
Boost Vaccination
Symptom
Group
Vaccine
Dose
N
Pain/tenderness
Redness
Swelling
Any local symptom
Chills
Headache
Joint pain
Malaise
Myalgia
Nausea
Temperature
Any systemic symptom
US EBO High (short)
US EBOZ Low
US EBOZ High
US EBO Low
US EBO High
US (DNA):EBO High
cAd3-EBO
cAd3-EBOZ
MVA-EbolaZ
Percentage of Participants

Supplementary Figure 2: Solicited reactogenicity detailed from the UG trial.

Percent of participants (*x* axis) reporting solicited local or systemic symptoms by vaccine group (*y* axis) in the seven days following each vaccination. For symptoms persisting more than one day, a single count per person at the maximum severity of the symptom was used for the figure.

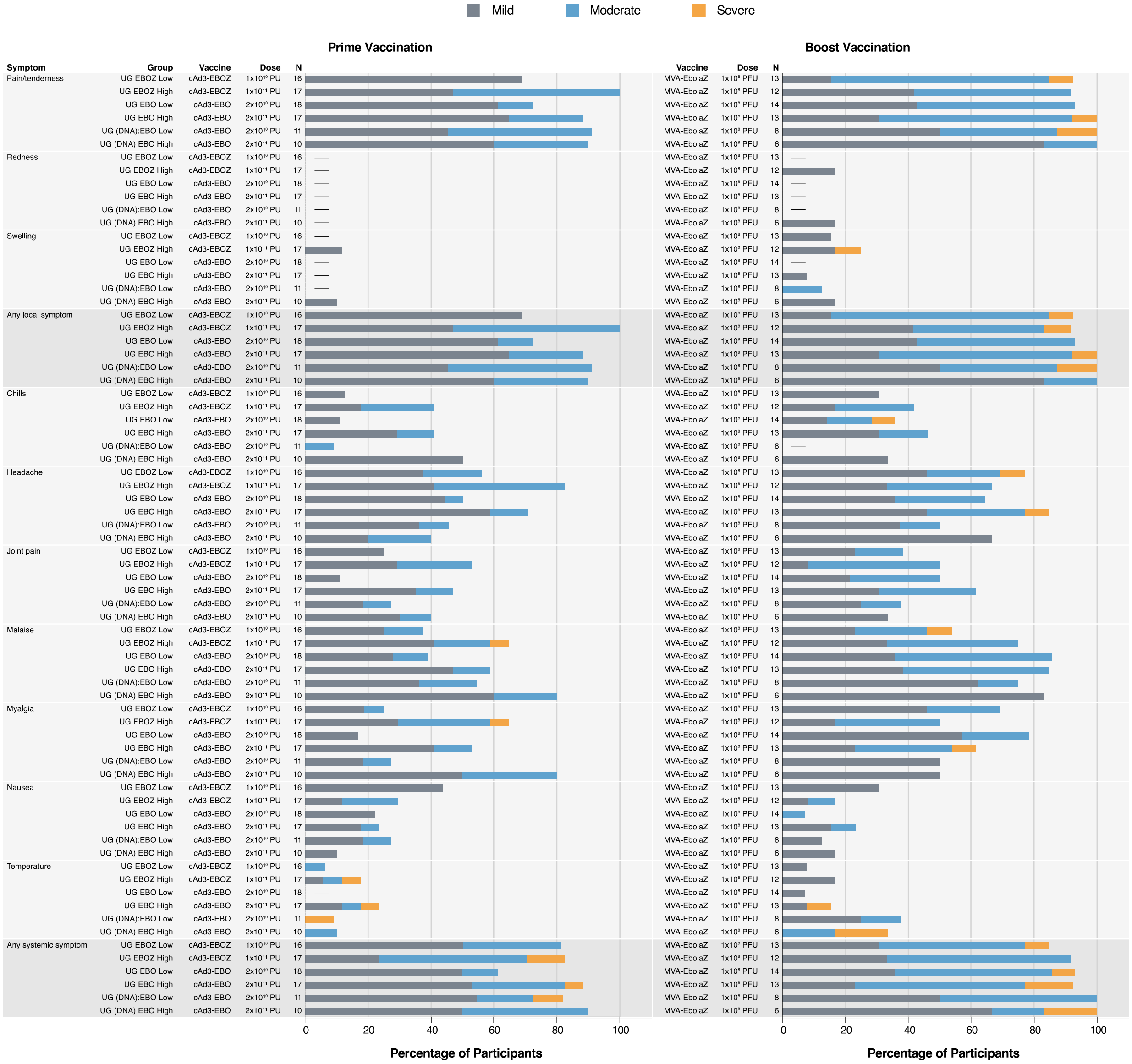

Supplementary Figure 3: cAd3-Ebola vaccines elicit SUDV GP-specific immune responses by four weeks after vaccination.

In (a), baseline-subtracted antibody titers following cAd3-EBOZ or cAd3-EBO vaccination in a subset of Trial US participants at four weeks after vaccination as assessed by SUDV GP ELISA. Data are expressed as EC_{90} titers, with individual dots noting each participants' titers and the lines noting the group geometric mean titer with error bars showing the 95% confidence interval. Titers were compared between groups using the Wilcoxon rank-sum test, and statistical significance is reported as denoted below (a). (b) and (c) display the percentage of memory background-subtracted CD4 (b) and CD8 (c) T cells responding to *ex vivo* SUDV GP stimulation with production of any tested cytokine at baseline and four weeks after cAd3-Ebola vaccination in Trial US participants. Box plots denote median with upper and lower quartiles of each group. The dotted line indicates background cytokine production in the absence of stimulation. For each group, titers were compared between baseline and week 4 using the Wilcoxon rank-sum test, and statistical significance is reported above each group for which $p<0.05$. The table in panel (d) supplies *n* and *p* values for figure (A) and *n* values for figures (b) and (c). These results have been partially reported (NCT02231866).[1,2] *See next page for figure*.

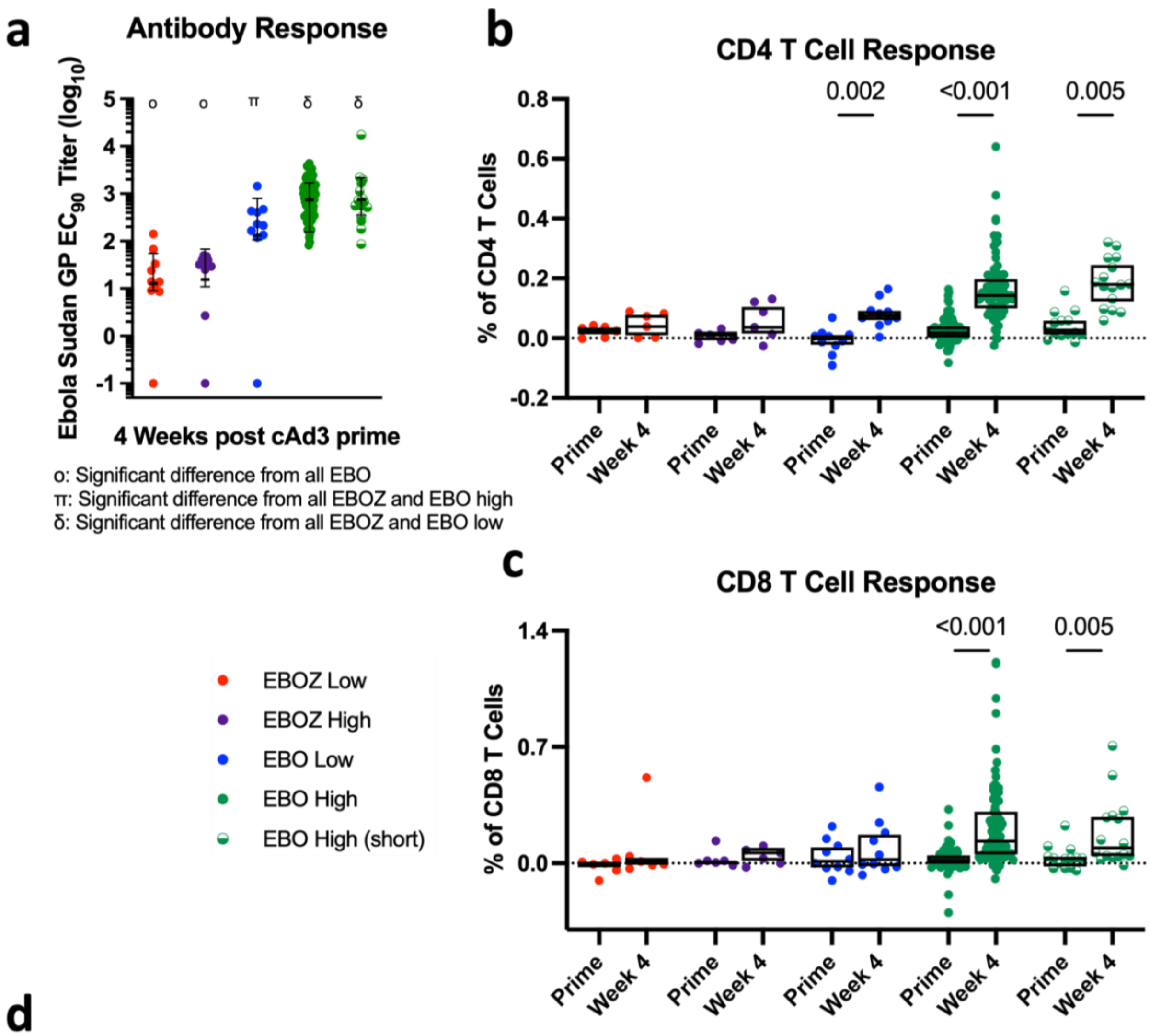

d

	ELISA					T cells	
Group		EBOZ Low	EBOZ High	EBO Low	EBO High	Prime	Wk4
	n	***p*-value**				***n***	
EBO High (short)	15	**<0.001**	**<0.001**	**0.0115**	0.7405	14	16
EBO High	64	**<0.001**	**<0.001**	**0.0026**		85	82
EBO Low	10	**0.0025**	**0.0041**			10	10
EBOZ High	10	0.3642				6	6
EBOZ Low	10					6	6

Supplementary Figure 4: MVA-EbolaZ alone elicited increased antibody titers but not T cell responses in the US trial.

(A) Antibody titers following MVA-EbolaZ vaccination of *Ebolavirus*-vaccine naïve participants in the US trial as assessed by EBOZ GP ELISA. Data expressed as geometric mean EC_{90} titers with 95% CI shown by error bars. (B,C) Percentage of memory background-subtracted CD4 (B) and CD8 (C) T cells responding to ex vivo stimulation with production of any tested cytokine at baseline and four weeks post MVA-EbolaZ vaccination. Box plots denote median with upper and lower quartiles of each group. Dotted line indicates zero cytokine production over levels detected without stimulation. PV: post-vaccination

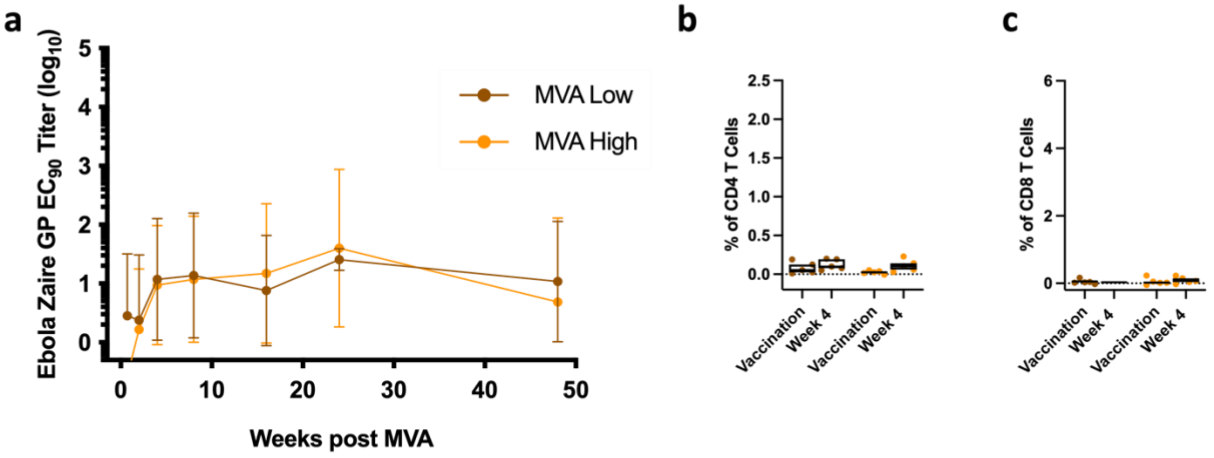

Supplementary Figure 5: cAd3 vector-specific responses were observed after all cAd3 vaccinations.

(A) Geometric means and 95% CIs of cAd3-neutralizing antibody titers following cAd3-Ebola vaccination in UG groups and the US EBO High (short) group as assessed by cAd3 neutralization assay. Data expressed as geometric mean IC_{90} titers with 95% CI shown by error bars. Remaining US results are available in previous reports.[1,2]

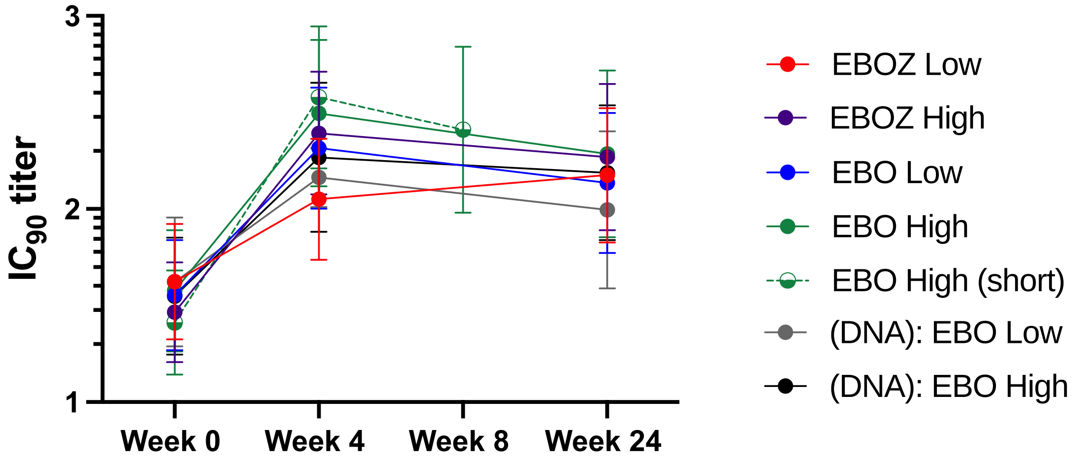

Supplementary Tables

Supplementary Table 1: Group Designations

Study site	Group Designation	Previous Ebola Vaccination	Prime		Boost	
			Vaccine	Dose	Vaccine	Dose
United States	US MVA Low	-	MVA-EbolaZ	$1x10^7$ PFU	-	-
	US MVA High	-	MVA-EbolaZ	$1x10^8$ PFU	-	-
	US EBO High (short)†	-	cAd3-EBO	$2x10^{11}$ PU	MVA-EbolaZ	$1x10^8$ PFU
	US EBOZ Low	-	cAd3-EBOZ*	$1x10^{10}$ PU	MVA-EbolaZ	$1x10^8$ PFU
	US EBOZ High	-	cAd3-EBOZ*	$1x10^{11}$ PU	MVA-EbolaZ	$1x10^8$ PFU
	US EBO Low	-	cAd3-EBO*	$2x10^{10}$ PU	MVA-EbolaZ	$1x10^8$ PFU
	US EBO High	-	cAd3-EBO*	$2x10^{11}$ PU	MVA-EbolaZ	$1x10^8$ PFU
	US (DNA): EBO High	Ebola DNA	cAd3-EBO*	$2x10^{11}$ PU	MVA-EbolaZ	$1x10^8$ PFU
Uganda	UG EBOZ Low	-	cAd3-EBOZ	$1x10^{10}$ PU	MVA-EbolaZ	$1x10^8$ PFU
	UG EBOZ High	-	cAd3-EBOZ	$1x10^{11}$ PU	MVA-EbolaZ	$1x10^8$ PFU
	UG EBO Low	-	cAd3-EBO	$2x10^{10}$ PU	MVA-EbolaZ	$1x10^8$ PFU
	UG EBO High	-	cAd3-EBO	$2x10^{11}$ PU	MVA-EbolaZ	$1x10^8$ PFU
	UG (DNA): EBO Low	Ebola DNA	cAd3-EBO	$2x10^{10}$ PU	MVA-EbolaZ	$1x10^8$ PFU
	UG (DNA): EBO High	Ebola DNA	cAd3-EBO	$2x10^{11}$ PU	MVA-EbolaZ	$1x10^8$ PFU

†The US EBO High (short) designation is reserved for those who received prime and boost vaccinations during Trial US. The prime-boost interval for this group was 6-11 weeks. US EBO High participants who received only MVA-EbolaZ as a boost during Trial US had a prime-boost interval of 12-52 weeks. *cAd3-Ebola vaccines administered during a prior clinical trial, for which results have been previously published.[1,2]

Supplementary Table 2: US Demographics

	Ebolavirus-vaccine Antigen Naïve			Previous cAd3-Ebola Vaccination					
**Category**	**MVA Low (N=5)**	**MVA High (N=5)**	**EBO High (short) (N=16)**	**EBOZ Low (N=9)**	**EBOZ High (N=9)**	**EBO Low (N=8)**	**EBO High (N=86)**	**(DNA): EBO High (N=2)**	**Overall (N=140)**
**Gender — no. (%)**									
**Male**	3 (60)	3 (60)	10 (63)	4 (44)	5 (56)	3 (38)	41 (48)	1 (50)	70 (50)
**Female**	2 (40)	2 (40)	6 (38)	5 (56)	4 (44)	5 (63)	45 (52)	1 (50)	70 (50)
**Age**									
**Mean (S.D.)**	33 (3.4)	34 (11)	31 (7.7)	35 (13)	39 (11)	35 (7.5)	40 (12)	56 (13)	38 (12)
**Range**	[28, 36]	[22, 47]	[20, 48]	[23, 59]	[24, 61]	[27, 50]	[22, 66]	[47, 65]	[20, 66]
**Race† — no. (%)**									
**Asian**	0 (0)	1 (20)	2 (13)	0 (0)	2 (22)	0 (0)	10 (11)	0 (0)	15 (11)
**Black or African American**	0 (0)	1 (20)	2 (13)	2 (22)	2 (22)	2 (25)	2 (2)	0 (0)	11 (8)
**White**	5 (100)	3 (60)	12 (75)	7 (78)	5 (56)	6 (75)	68 (79)	2 (100)	108 (77)
**Multiracial**	0 (0)	0 (0)	0 (0)	0 (0)	0 (0)	0 (0)	6 (7)	0 (0)	6 (4)
**Hispanic or Latino ethnic group† — no. (%)**									
**Non-Hispanic/Latino**	4 (80)	4 (80)	15 (94)	8 (89)	9 (100)	8 (100)	82 (95)	2 (100)	132 (94)
**Hispanic/Latino**	1 (20)	1 (20)	1 (6)	1 (11)	0 (0)	0 (0)	3 (3)	0 (0)	7 (5)
**Unknown/Not Reported**	0 (0)	0 (0)	0 (0)	0 (0)	0 (0)	0 (0)	1 (1)	0 (0)	1 (1)
**BMI‡**									
**Mean (S.D.)**	24 (4.4)	26 (3.8)	26 (2.8)	28 (5.4)	25 (3.5)	27 (5.0)	26 (4.7)	36 (4.7)	26 (4.6)
**Range**	[19-31]	[23-32]	[22-34]	[19-35]	[21-32]	[22-36]	[18-40]	[32-39]	[18-40]
**Educational level — no. (%)**									
**High school graduate/GED**	1 (20)	1 (20)	1 (6)	1 (11)	1 (11)	0 (0)	2 (2)	0 (0)	7 (5)
**College/University**	2 (40)	2 (40)	6 (38)	4 (44)	1 (11)	3 (38)	21 (24)	0 (0)	39 (28)
**Advanced degree**	2 (40)	2 (40)	9 (56)	4 (44)	7 (78)	5 (63)	63 (73)	2 (100)	94 (67)

†Race and ethnic group were reported by the participants. ‡ The body-mass index (BMI) is the weight in kilograms divided by the square of the height in meters.
This calculation was performed on the basis of weight and height measured at the time of enrollment.

Supplementary Table 3: UG Demographics

Category	*Ebolavirus*-Vaccine Naïve				Previous DNA Ebola Vaccination		
	**EBOZ Low (N=17)**	**EBOZ High (N=17)**	**EBO Low (N=18)**	**EBO High (N=17)**	**(DNA): EBO Low (N=11)**	**(DNA): EBO High (N=10)**	**Overall (N=90)**
**Gender- no. (%)**							
**Male**	16 (94)	15 (88)	15 (83)	12 (71)	8 (73)	9 (90)	75 (83)
**Female**	1 (6)	2 (12)	3 (17)	5 (29)	3 (27)	1 (10)	15 (17)
**Age**							
**Mean (S.D.)**	27 (5)	29 (6)	31 (7)	29 (6)	33 (7)	34 (5)	30 (7)
**Range**	[20, 38]	[20, 43]	[19, 45]	[20, 41]	[23, 48]	[28, 42]	[19, 48]
**Race - no. (%)**							
**African Ugandan**	17 (100)	17 (100)	18 (100)	17 (100)	11 (100)	10 (100)	90 (100)
**BMI†**							
**Mean (S.D.)**	22 (4)	22 (2)	23 (4)	22 (5)	22 (5)	22 (6)	22 (4)
**Range**	[18, 33]	[18, 28]	[18, 30]	[17, 36]	[16, 36]	[17, 37]	[16, 37]
**Education - no. (%)**							
**None**	0 (0)	0 (0)	1 (6)	0 (0)	0 (0)	0 (0)	1 (1)
**Primary**	0 (0)	2 (12)	2 (11)	2 (12)	2 (18)	1 (10)	9 (10)
**Secondary**	8 (47)	10 (59)	7 (39)	6 (35)	3 (27)	6 (60)	40 (44)
**College/University**	9 (53)	5 (29)	8 (44)	9 (53)	6 (55)	3 (30)	40 (44)

†The body-mass index (BMI) is the weight in kilograms divided by the square of the height in meters. This calculation was performed on the basis of weight and height measured at the time of enrollment.

Supplementary Table 4: Trial US Adverse Events (AEs) assessed as related to study product.

Term	Severity	N (%)	D. after Vaccination	D. to Resolution
**cAd3-EBO High**		**8/16 (50.0)**		
Prolonged aPTT	*Total*	*3 (18.8)*		
	Mild	1(6.3)	15	41
	Moderate	2 (12.5)	14	39-54
Axillary Pain	Mild	1 (6.3)	2	3
Leukopenia	Mild	3 (18.8)	2	6-12
Lymphopenia	Mild	2 (12.5)	2	12-13
Neutropenia	Moderate	1 (6.3)	2	6
**MVA-EbolaZ**		**38/136 (27.9)**		
Prolonged aPTT	*Total*	*4 (2.9)*		
	Mild	3 (2.2)	2	1-14
	Moderate	1 (0.7)	2	12
ALT increased	Mild	2 (1.5)	2-14	12-14
Diarrhea	Moderate	2 (1.5)	2	2-3
Hyperesthesia	Mild	1 (0.7)	0	2
Injection site nodule	Mild	1 (0.7)	15	2
Injection site pruritus	Mild	1 (0.7)	6	0
Leukopenia	Mild	7 (5.1)	2-28	10-28
Lymphadenopathy	Mild	2 (1.5)	0-4	1-3
Lymphopenia	*Total*	*10 (7.4)*		
	Mild	9 (6.6)	2	6-15
	Moderate	1 (0.7)	2	12
Neutropenia	*Total*	*12 (8.8)*		
	Mild	4 (2.9)	2-3	12-25
	Moderate	6 (4.4)	2	4-12
	Severe	2 (1.5)	2-3	2-5
Paresthesia	Mild	1 (0.7)	1	1
Syncope	Moderate	1 (0.7)	0	0
Thrombocytopenia	Mild	1 (0.7)	2	12
Vomiting	Mild	1 (0.7)	1	0
**VRC 208 Related AE Total**		**44/137 (32.1)**		

N: number of participants with AE out of number vaccinated, aPTT: activated partial thromboplastin time, ALT: alanine aminotransferase, D: Days

Supplementary Table 5: Trial UG Adverse Events (AEs) assessed as related to study product.

Term	Severity	N (%)	D. after Vaccination	D. to Resolution
**cAd3-EBO**		**10/56 (17.9)**		
**cAd3-EBO Low**		2/29 (6.9)		
Hb decreased	Mild	1 (3.4)	3	20
Leukopenia	Mild	1 (3.4)	14	14
**cAd3-EBO High**		8/27 (29.6)		
ALT increased	Mild	2 (7.4)	3-14	11-14
Hb decreased	Mild	1 (3.7)	14	14
Headache	Mild	1 (3.7)	16	5
Leukopenia	Mild	3 (11.1)	3	11-110
Lymphopenia	Mild	1 (3.7)	3	11
Neutropenia	Mild	2 (7.4)	3	11
**cAd3-EBOZ**		**12/34 (35.3)**		
**cAd3-EBOZ Low**		4/17 (23.5)		
Epistaxis	Mild	1 (5.9)	1	0
Leukopenia	Mild	3 (17.6)	3-14	11-98
Neutropenia	Mild	1 (5.9)	3	11
**cAd3-EBOZ High**		8/17 (47.1)		
Hb decreased	Moderate	1 (5.9)	28	83
Hypersensitivity	Mild	1 (5.9)	1	1
Laryngitis	Mild	1 (5.9)	1	11
Leukopenia	Mild	3 (17.6)	3	11
Neutropenia	*Total*	*4 (23.5)*		
	Mild	2 (11.8)	3	11
	Moderate	2 (11.8)	3	11
**MVA-EbolaZ**		**9/66 (13.6)**		
Abdominal discomfort	Mild	1 (1.5)	0	3
Decreased appetite	Mild	2 (3.0)	0-1	1-2
Dizziness	Mild	1 (1.5)	1	0
Lymph node pain	Mild	1 (1.5)	26	30
Lymphadenopathy	Mild	1 (1.5)	3	1
Neutropenia	Mild	2 (3.0)	2	12
Palpitations	Mild	1 (1.5)	1	0
Tachycardia	Mild	1 (1.5)	2	4
Tachypnoea	Severe	1 (1.5)	2	110
**RV 422 Related AE Total**		**31/90 (34.4)**		

N: number of participants with AE out of number vaccinated, Hb: Hemoglobin, ALT: alanine aminotransferase, D: Days

Supplementary Table 6: Results of Wilcoxon rank-sum test comparing EBOZ-specific IgG titers elicited by the different vaccination regimens in Trial US.

Group	N	Week	EBOZ Low	EBOZ High	EBO Low	EBO High
				p-value		
(DNA) EBO High	3	4	**0.0070**	**0.0070**	**0.0070**	**0.0037**
EBO High	105	4	0.2562	0.7099	**0.0018**	
EBO Low	10	4	0.0892	**0.0288**		
EBOZ High	10	4	0.2799			
EBOZ Low	10	4				
(DNA) EBO High	3	24	**0.0070**	**0.0280**	**0.0070**	**0.0092**
EBO High	71	24	0.1411	0.1573	**0.0004**	
EBO Low	10	24	**0.0288**	**<0.0001**		
EBOZ High	10	24	**0.0068**			
EBOZ Low	10	24				

Bolded values indicated p-values < 0.05.

Supplementary Table 7: Results of Wilcoxon rank-sum test comparing EBOZ-specific IgG titers elicited by the different vaccination regimens in Trial UG.

Group	N	Week	EBOZ Low	EBOZ High	EBO Low	EBO High	(DNA) EBO Low
				**p-value**			
(DNA) EBO High	10	4	**<0.0001**	**<0.0001**	**<0.0001**	**<0.0001**	0.5116
(DNA) EBO Low	11	4	**0.0001**	**0.0002**	**0.0003**	**0.0003**	
EBO High	17	4	**0.0314**	0.5572	**0.0048**		
EBO Low	18	4	0.4235	**0.0326**			
EBOZ High	16	4	0.0743				
EBOZ Low	16	4					
(DNA) EBO High	9	24	**<0.0001**	**0.0231**	**<0.0001**	**0.0250**	0.9048
(DNA) EBO Low	10	24	**<0.0001**	**0.0041**	**<0.0001**	**0.0032**	
EBO High	17	24	**0.0003**	0.6312	**0.0003**		
EBO Low	18	24	0.4677	0.0009			
EBOZ High	16	24	**0.0012**				
EBOZ Low	17	24					
(DNA) EBO High	7	48	**0.0036**	0.3114	**0.0040**	0.1750	0.9623
(DNA) EBO Low	10	48	**0.0007**	**0.0769**	**0.0002**	**0.0356**	
EBO High	16	48	**0.0166**	0.8460	**0.0091**		
EBO Low	18	48	0.9585	0.0152			
EBOZ High	13	48	**0.0330**				
EBOZ Low	16	48					

Supplementary Table 8: Sample numbers for Ebola Zaire-specific immunogenicity assays.

cAd3 Prime Trial US	ELISA							CD4 and CD8 T Cells	
**Week**	**1**	**2**	**4**	**8**	**16**	**24**	**48**	**Prime**	**Wk4**
**Group**	n							n	
EBOZ Low	10	10	10	10	10	10	1	7	7
EBOZ High	10	10	10	10	9	10	1	7	7
EBO Low	10	10	10	10	10	10	3	10	10
EBO High	109	107	108	105	92	71	20	86	82
DNA EBO High	3	3	3	3	3	3	1	3	3
EBO High (short)	16	16	16	16	n/a	n/a	n/a	16	16
**cAd3 Prime Trial UG**	**ELISA**							**CD4 and CD8 T Cells**	
**Week**	**1**	**2**	**4**	**8**	**16**	**24**	**48**	**Prime**	**Wk4**
**Group**	n							n	
EBOZ Low	17	16	17	16	17	17	16	17	15
EBOZ High	18	16	16	16	16	16	13	17	16
EBO Low	18	18	18	18	18	18	18	16	18
EBO High	17	17	17	17	17	17	16	17	16
DNA EBO Low	11	11	11	10	11	10	10	11	11
DNA EBO High	10	10	10	10	9	9	7	10	10
**MVA Boost Trial US**	**ELISA**							**CD4 and CD8 T Cells**	
**Week**	**1**	**2**	**4**	**8**	**16**	**24**	**48**	**Boost**	**Wk4**
**Group**	n							n	
EBO High (short)	15	15	15	14	14	14	14	16	16
EBOZ Low	9	9	9	9	9	9	9	9	9
EBOZ High	9	9	9	9	9	9	9	9	9
EBO Low	7	7	7	7	7	7	7	7	7
EBO High	83	81	84	81	83	83	73	84	84
DNA EBO High	2	2	2	2	2	2	1	2	2
**MVA Boost Trial UG**	**ELISA**							**CD4 and CD8 T Cells**	
**Week**	**1**	**2**	**4**	**8**	**16**	**24**	**48**	**Boost**	**Wk4**
**Group**	n							n	
EBOZ Low	13	13	13	12	11	12	11	8	13
EBOZ High	12	12	12	11	11	11	11	8	12
EBO Low	14	14	14	14	14	14	14	12	14
EBO High	13	13	13	13	12	13	12	10	13
DNA EBO Low	8	8	8	8	8	8	8	8	8
DNA EBO High	6	6	6	6	6	6	6	2	6

Supplementary References

1. Ledgerwood JE, DeZure AD, Stanley DA, et al. Chimpanzee Adenovirus Vector Ebola Vaccine. *N Engl J Med* 2017; **376**(10): 928-38.
2. Tapia MD, Sow SO, Lyke KE, et al. Use of ChAd3-EBO-Z Ebola virus vaccine in Malian and US adults, and boosting of Malian adults with MVA-BN-Filo: a phase 1, single-blind, randomised trial, a phase 1b, open-label and double-blind, dose-escalation trial, and a nested, randomised, double-blind, placebo-controlled trial. *Lancet Infect Dis* 2016; **16**(1): 31-42.

Version 3.0
January 4, 2017

VACCINE RESEARCH CENTER

Protocol VRC 208
(NIH 15-I-0107)

A Phase 1/1b Open-Label Clinical Trial to Evaluate Dose, Safety and Immunogenicity of a Recombinant Modified Vaccinia Virus Ankara Ebola Vaccine, VRC-EBOMVA079-00-VP (MVA-EbolaZ), Administered Alone or as a Boost to cAd3-Ebola Vaccines in Healthy Adults

Vaccine Provided by
National Institute of Allergy and Infectious Diseases (NIAID)
Vaccine Research Center (VRC)
Bethesda, Maryland

Clinical Trial Sponsored by:
National Institute of Allergy and Infectious Diseases (NIAID)
Vaccine Research Center (VRC)
Bethesda, Maryland

BB-IND 16343 – held by VRC, NIAID, NIH

VRC Site Principal Investigator and Protocol Chair:
Julie E. Ledgerwood, D.O.
Vaccine Research Center,
National Institute of Allergy and Infectious Diseases (NIAID)
National Institutes of Health (NIH)
Bethesda, MD 20892

IRB Initial Review Date: February 9, 2015

TABLE OF CONTENTS

Page

TABLE OF CONTENTS 2
ABBREVIATIONS 5
PRÉCIS 8
1. INTRODUCTION AND RATIONALE 10
1.1. Ebola Infection: Background 10
1.2. Rationale for Development of VRC-EBOMVA079-00-VP Vaccine 11
1.2.1. Previous Human Experience with VRC Filovirus Vaccines 12
1.2.2. Previous Human Experience with Modified Vaccinia Virus Ankara (MVA) and MVA-vectored Vaccines 14
1.3. Assessment of Immunogenicity 15
2. BACKGROUND ON VACCINE 15
2.1. Description of the Study Agents 15
2.2. Preclinical Studies Supporting Safety of cAd3 and MVA Constructs 16
2.3. Nonclinical Immunogenicity and Protection Studies of cAd3 and MVA Constructs 16
3. STUDY OBJECTIVES 17
3.1. Primary Objectives 17
3.2. Secondary Objectives 18
3.3. Exploratory Objectives 18
4. STUDY DESIGN AND METHODS 18
4.1. Study Population 20
4.1.1. Inclusion Criteria for Groups 1, 2, and 3. 20
4.1.2. Exclusion Criteria for Groups 1, 2, and 3 21
4.1.3. Rollover Criteria for Groups 4, 5, 6, and 7. 22
4.2. Clinical Procedures and Schedule of Evaluations 23
4.2.1. Screening 23
4.2.2. Study Schedule and Enrollment 24
4.2.3. Administration of the Study Injection 24
4.2.4. 7-Day Solicited Reactogenicity and Follow-up 24
4.2.5. Concomitant Medications 25
4.3. Criteria for Dose Escalation and Dose Continuation in Part 1 of the Study 26
4.4. Criteria for Discontinuing Study Injections or Protocol Participation 26
4.4.1. Discontinuation of Study Injections 26
4.4.2. Criteria for Discontinuing Study Protocol Participation 27
4.5. Criteria for Pausing the Study 27
5. SAFETY AND ADVERSE EVENT REPORTING 28
5.1. Adverse Events 28
5.2. Serious Adverse Events 28

5.3. Adverse Event Reporting to the IND Sponsor 29
5.3.1. IND Sponsor Reporting to the FDA 29
5.4. Reporting to the Institutional Review Board 29
5.4.1. Unanticipated Problem (UP) Definition 29
5.4.2. Protocol Deviation Definition 30
5.4.3. Non-Compliance Definition 30
5.4.4. Expedited Reporting to the NIAID IRB 30
5.4.5. Annual Reporting to the NIAID IRB 31
5.5. Serious Adverse Event Reporting to the Institutional Biosafety Committee 31
6. STATISTICAL CONSIDERATIONS 31
6.1. Overview 31
6.2. Objectives 31
6.3. Endpoints 31
6.3.1. Primary Endpoints: Safety 31
6.3.2. Secondary Endpoints: Immunogenicity 32
6.3.3. Exploratory Endpoints 32
6.4. Sample Size and Accrual 32
6.4.1. Power Calculations for Safety 32
6.4.2. Sample Size Calculations for Immunogenicity 34
6.5. Statistical Analysis 34
6.5.1. Analysis Variables 34
6.5.2. Baseline Demographics 34
6.5.3. Safety Analysis 35
6.5.4. Immunogenicity Analysis 35
6.5.5. Interim analyses 35
6.6. Randomization of Treatment Assignments 36
7. PHARMACY AND VACCINE ADMINISTRATION PROCEDURES 36
7.1. Study Agents 36
7.1.1. VRC-EBOMVA079-00-VP, MVA-EbolaZ vaccine 36
7.1.2. VRC-EBOADC069-00-VP, cAd3-EBO vaccine 37
7.1.3. Study Agent Labels 37
7.1.4. Study Agent Storage: 37
7.2. Preparation of Study Agent for Injection 37
7.2.1. Preparation of VRC-EBOMVA079-00-VP at $1x10^7$ PFU dose 38
7.2.2. Preparation of VRC-EBOMVA079-00-VP at $1x10^8$ PFU dose 38
7.2.3. Preparation of VRC-EBOADC069-00-VP at $2x10^{11}$ PU dose 38
7.3. Study Agent Accountability 38
7.3.1. Documentation 38
7.3.2. Disposition 38
8. HUMAN SUBJECT PROTECTIONS AND ETHICAL OBLIGATIONS 39
8.1. Institutional Review Board 39
8.2. Subject Recruitment and Enrollment 39

8.3. Informed Consent 39
8.4. Subject Confidentiality 39
8.5. Risks and Benefits 40
8.5.1. Risks of the MVA-EbolaZ Vaccine 40
8.5.2. Risks of the cAd3-EBO Vaccine 40
8.5.3. Other Risks 41
8.5.4. Study Benefits 41
8.6. Plan for Use and Storage of Biological Samples 41
8.6.1. Use of Samples, Specimens and Data 41
8.6.2. Storage and Tracking of Blood Samples and Other Specimens 42
8.6.3. Disposition of Samples, Specimens and Data at Completion of the Protocol 42
8.6.4. Loss or Destruction of Samples, Specimens or Data 42
8.7. Subject Identification and Enrollment of Study Participants 42
8.7.1. Participation of Children 43
8.7.2. Participation of NIH Employees and Employees of Study Sites 43
8.8. Compensation 43
8.9. Safety Monitoring, Protocol Safety Review Team 43
9. ADMINISTRATIVE AND LEGAL OBLIGATIONS 44
9.1. Protocol Amendments and Study Termination 44
9.2. Study Documentation and Storage 44
9.3. Data Collection and Protocol Monitoring 45
9.3.1. Data Collection 45
9.3.2. Source Documents 45
9.3.3. Protocol Monitoring Plan 45
9.4. Language 45
9.5. Policy Regarding Research-Related Injuries 45
9.6. Multi-site Management 46
10. REFERENCES 47
APPENDIX 1. INFORMED CONSENT FORM 50
APPENDIX 2. CONTACT INFORMATION 60
APPENDIX 3. SCHEDULE OF EVALUATIONS 63
APPENDIX 4. TABLE FOR GRADING SEVERITY OF ADVERSE EVENTS 68

ABBREVIATIONS

Abbreviation	Term
AAE	acquired angioedema
Ad5	human adenovirus serotype 5
ADL	activities of daily living
AE	adverse event
ALT	alanine aminotransferase
AoU	assessment of understanding
APA	Anti-phospholipid antibody
BDBV	Species *Bundibugyo Ebolavirus*
BMI	body mass index
cAd	recombinant chimpanzee adenovirus
cAd3	recombinant chimpanzee adenovirus serotype 3
cAd63	recombinant chimpanzee adenovirus serotype 63
cAd3-EBO	recombinant chimpanzee adenovirus type 3-vectored Ebola virus vaccine encoding GP from Zaire and Sudan species of Ebola virus
cAd3-EBOZ	recombinant chimpanzee adenovirus type 3-vectored Ebola virus vaccine encoding GP from Zaire species of Ebola virus
CBC	complete blood count
CDMS	clinical data management system
cGMP	current Good Manufacturing Practices
EBOV	Species *Zaire Ebolavirus*
ECG	electrocardiogram
EHF	Ebola hemorrhagic fever
ELISA	enzyme-linked immunosorbent assay
ELISPOT	enzyme-linked immunospot
EVD	Ebola virus disease
FDA	Food and Drug Administration
GCP	Good Clinical Practices
GLP	Good Laboratory Practices
GP	glycoprotein
GP (S)	glycoprotein from *Sudan Ebolavirus*
GP (Z)	glycoprotein from *Zaire Ebolavirus*
HAE	hereditary angioedema
HIV	human immunodeficiency virus
HLA	human leukocyte antigen
HRPP	Human Research Protections Program
IB	Investigator’s Brochure
IBC	Institutional Biosafety Committee
ICH	International Conference on Harmonisation

Abbreviation	Term
ICS	intracellular cytokine staining
ICTV	International Committee on the Taxonomy of Viruses
IgG	Immunoglobulin G
IgM	Immunoglobulin M
IM	Intramuscular
IND	investigational new drug application
IRB	Institutional Review Board
LIMS	Laboratory Information Management System
MedDRA	Medical Dictionary for Regulatory Activities
MVA	modified vaccinia virus Ankara
NIAID	National Institute of Allergy and Infectious Diseases
NIH	National Institutes of Health
NHP	non-human primate
NP	nucleoprotein
NSAID	nonsteroidal anti-inflammatory drug
NVITAL	NIAID Vaccine Immune T-Cell and Antibody Laboratory
PBMC	peripheral blood mononuclear cells
PBS	phosphate buffered saline
PI	Principal Investigator
PM	point mutation
PSRT	Protocol Safety Review Team
PT	prothrombin time
PTT / aPTT	partial thromboplastin time; used interchangeably with aPTT, activated partial thromboplastin time
PFU	plaque forming units
PU	particle units
rAd	recombinant human adenovirus
rAd5	recombinant human adenovirus serotype 5
RESTV	Species *Reston Ebolavirus*
RNA	ribonucleic acid
SAE	serious adverse event
SAS	Statistical Analysis System
SC	Study Coordinator
SUDV	Species *Sudan Ebolavirus*
SUSAR	suspected, unexpected and serious adverse reaction
TAFV	Species *Taï Forest Ebolavirus*
ULN	upper limit of normal
UP	unanticipated problem
VRC	Vaccine Research Center

Abbreviation	Term
VP	virus particle
VPP	VRC/NIAID/Vaccine Pilot Plant
WBC	white blood cell
WHO	World Health Organization
WT	wild type

PRÉCIS

VRC 208 Study: A Phase 1/1b Open-Label Clinical Trial to Evaluate Dose, Safety and Immunogenicity of a Recombinant Modified Vaccinia Virus Ankara Ebola Vaccine, VRC-EBOMVA079-00-VP (MVA-EbolaZ), Administered Alone or as a Boost to cAd3-Ebola Vaccines in Healthy Adults

Study Design: This Phase 1/1b study will examine dose, safety, tolerability and immunogenicity of an investigational modified vaccinia virus Ankara (MVA) -vectored Ebola vaccine (MVA-EbolaZ) in healthy adults. The vaccine encodes wild type (WT) glycoprotein (GP) from the Zaire strain of Ebola and will be administered intramuscularly (IM) with needle and syringe. The safety and tolerability of the MVA-EbolaZ will be evaluated at escalating doses of $1x10^7$ and $1x10^8$ plaque-forming units (PFU). Part 1 includes enrollment of Ebola vaccine-naive subjects to conduct a dose escalation of the MVA-EbolaZ vaccine and to evaluate the vaccine as a boost for the cAd3-EBO vaccine. In Part 2 of the study, about 140 subjects who received the cAd3-EBO or cAd3-EBOZ vaccine in the VRC 207 study will be boosted with MVA-EbolaZ. The hypothesis is that the study vaccines will be safe and will elicit immune responses to Ebola GP. In addition, we hypothesize that the prime-boost regimens will be safe and will result in a polyfunctional response to Ebola GP that is of greater magnitude and duration than the response to either of the vaccines alone.

Objectives: The primary objectives are: 1) to evaluate the safety and tolerability of MVA-EbolaZ vaccine administered at $1x10^7$ and $1x10^8$ PFU and 2) to evaluate the safety and tolerability of the prime-boost regimens. The secondary objectives are related to immunogenicity of MVA-EbolaZ administered alone and as a booster vaccination for the cAd3-EBO or cAd3-EBOZ vaccines.

Product Description: VRC-EBOMVA079-00-VP (MVA-EbolaZ) is composed of a recombinant MVA vector that expresses WT Ebola GP from Zaire strain. It is formulated at $3.2x10^8$ PFU/mL. Injections will be administered IM in a 0.3 mL volume by needle and syringe. The diluent for MVA-EbolaZ is phosphate buffered saline (PBS, VRC-PBSPLA043-00-VP).

VRC-EBOADC069-00-VP (cAd3-EBO) is composed of two recombinant cAd3 vectors in a 1:1 ratio that express Ebola WT GPs from Zaire and Sudan strains. It is formulated at $2x10^{11}$ PU/mL. Injections will be administered IM in a 1 mL volume by needle and syringe.

Subjects: Part 1: Healthy adult volunteers, 18 to 50 years old who are Ebola vaccine-naive;
Part 2: Healthy adult volunteers, 18 to 66 years old who received cAd3-vectored Ebola vaccines in the VRC 207 study.

Study Plan: **Part 1:** 15 Ebola vaccine-naive subjects will be randomized at the VRC NIH Clinical Center site to either Group 1 or Group 3 in a 1:2 ratio. Group 1 subjects will receive MVA-EbolaZ at $1x10^7$ PFU and Group 3 subjects will receive the cAd3-EBO vaccine at $2x10^{11}$ PU. When criteria for dose escalation are met for Group 1, 5 vaccine-naive subjects will be enrolled into Group 2 to receive MVA-EbolaZ at $1x10^8$ PFU. In Groups 1 and 2, no more than one vaccination per day will occur for the first 3 subjects. When there is at least 1 week of follow-up safety data for the first 3 vaccinated subjects in Group 1, an interim safety review will occur before proceeding with dose escalation and enrollment of Group 2. Similarly, an interim safety review will occur when there is at least 1 week of follow-up safety data for the first 3 vaccinated subjects in Group 2. If MVA-EbolaZ at $1x10^8$ PFU is assessed as safe for

further evaluation, then 3 subjects on a prime-boost schedule will receive a booster injection with MVA-EbolaZ at $1x10^8$ PFU; these 3 pilot subjects may be from either Group 3 or Group 7. An interim safety review will occur once the first 3 subjects boosted with MVA-EbolaZ have completed 7 days of follow-up. This interim safety review will occur before additional subjects are boosted in Groups 3 through 7.

Part 2: Subjects who previously received either the cAd3-EBO or cAd3-EBOZ vaccine in VRC 207 and completed at least 12 weeks of follow-up, will be offered to enroll into VRC 208 to receive an MVA-EbolaZ booster injection. Part 2 of the study will be conducted at all three VRC 207 sites: the VRC NIH Clinical Center (CC), The Hope Clinic of the Emory Vaccine Center, and the University of Maryland Center for Vaccine Development. Enrollment of all Part 2 groups may occur simultaneously.

**VRC 208 Schema**				
**Group**	**Subjects**	**Day 0**	**Week 8 Boost**	**Study site**
1	5	MVA-EbolaZ ($1x10^7$ PFU)		VRC
2	5	MVA-EbolaZ ($1x10^8$ PFU)		VRC
3	10*	cAd3-EBO ($2x10^{11}$ PU)	MVA-EbolaZ ($1x10^8$ PFU)	VRC
**Part 2: Evaluation of MVA-EBOLAZ boost for VRC 207 subjects**				
**Group**	**Subjects**	**Prime received in VRC 207**	**Day 0 Boost**	
4	up to 10	cAd3-EBOZ ($1x10^{10}$ PU)	MVA-EbolaZ ($1x10^8$ PFU)	UMD
5	up to 10	cAd3-EBOZ ($1x10^{11}$ PU)	MVA-EbolaZ ($1x10^8$ PFU)	UMD
6	up to 10	cAd3-EBO ($2x10^{10}$ PU)	MVA-EbolaZ ($1x10^8$ PFU)	VRC
7	up to 110	cAd3-EBO ($2x10^{11}$ PU)	MVA-EbolaZ ($1x10^8$ PFU)	VRC, Emory
Total	up to 160	All injections are IM in deltoid muscle with needle and syringe. For Groups 4-7, the interval of time between the prime received in VRC 207 and the boost in VRC 208 will vary but will be at least 12 weeks. To the degree possible, within Group 7, the enrolling site will attempt to target the timing of the rollover into VRC 208 to fit within the subgroups with 12, 16, and 24 week intervals between the prime and the booster.<br>*After the randomization of 10 subjects into Group 3, up to 10 additional subjects may be enrolled. The total accrual to the study will not exceed 160.		

Study Duration: Subjects will be evaluated by 9 clinic visits over 48 weeks for Groups 1, 2, and 4-7; and by 15 visits over 56 weeks for Group 3.

1. INTRODUCTION AND RATIONALE

1.1. Ebola Infection: Background

In 2013, the International Committee on the Taxonomy of Viruses (ICTV) Filoviridae Study Group and other experts published an updated taxonomy for filoviruses. Ebolavirus is one of three genera in the family Filoviridae, which along with Marburgvirus and Cuevavirus, are known to induce viral hemorrhagic fever. Five distinct species included in the genus Ebolavirus are Bundibugyo (BDBV), Reston (RESTV), Sudan (SUDV), Taï Forest (TAFV), and Zaire (EBOV) [1].

Ebolavirus is a large, negative-strand RNA virus composed of 7 genes encoding viral proteins, including a single glycoprotein (GP) [2-4]. The virus is responsible for causing Ebola virus disease (EVD), formerly known as Ebola hemorrhagic fever (EHF), in humans. In particular, BDBV, EBOV, and SUDV have been associated with large outbreaks of EVD in Africa and reported case fatality rates of up to 90% [5]. Transmission of Ebola virus to humans is not yet fully understood, but is likely due to incidental exposure to infected animals [6-8]. EVD spreads through human-to-human transmission, with infection resulting from direct contact with blood, secretions, organs or other bodily fluids of infected people, and indirect contact with environments contaminated by such fluids [5].

EVD has an incubation period of 2 to 21 days (7 days on average, depending on the strain) followed by a rapid onset of non-specific symptoms such as fever, extreme fatigue, gastrointestinal complaints, abdominal pain, anorexia, headache, myalgias and/or arthralgias. These initial symptoms last for about 2 to 7 days after which more severe symptoms related to hemorrhagic fever occur, which may include hemorrhagic rash, epistaxis, hematuria, hemoptysis, hematemesis, melena, conjunctival hemorrhage, tachypnea, confusion, somnolence, hearing loss, and in some cases, both internal and external bleeding. Laboratory findings include low white blood cell and platelet counts and elevated liver enzymes [5]. In general, the symptoms last for about 7 to14 days after which recovery may occur. Death can occur 6 to 16 days after the onset of symptoms, with vomiting and diarrhea resulting in dehydration, shock and multi-organ failure being the more common causes of death [6, 9]. People are infectious as long as their blood and secretions contain the virus; men who have recovered from the disease can still transmit the virus through their semen for up to 7 weeks after recovery from illness [5, 10, 11].

Immunoglobulin M (IgM) antibodies to the virus appear 2 to 9 days after infection whereas immunoglobulin G (IgG) antibodies appear approximately 17 to 25 days after infection, which coincides with the recovery phase. In survivors of EVD, both humoral and cellular immunity are detected, however, their relative contribution to protection is unknown [12].

While prior outbreaks of EVD have been localized to regions of Africa, there is a potential threat of spread to other countries given the frequency of international travel. The 2014 outbreak in West Africa was first recognized in March 2014 and by June 2014 the known case rate had exceeded the largest prior EVD outbreak, which occurred in Uganda in 2000-2001 with 425 cases and 224 deaths (case-fatality rate=53%) [13]. As of January 14, 2015, the reported number of EVD cases has been 21,296 and 8,429 deaths have been reported [14].

Viruses in the Filoviridae family are also categorized as potential threats for use as biological weapons due to ease of dissemination and transmission, and high levels of mortality. Currently,

no specific antiviral therapies or FDA-licensed vaccines exist for any member of Filoviridae family of viruses.

1.2. Rationale for Development of VRC-EBOMVA079-00-VP Vaccine

The Vaccine Research Center (VRC), National Institute of Allergy and Infectious Diseases (NIAID), National Institutes of Health (NIH) has developed the recombinant live modified vaccinia virus Ankara (MVA)-vectored Ebola vaccine, VRC-EBOMVA079-00-VP (MVA-EbolaZ). The vaccine is composed of a recombinant MVA virus that expresses wild type (WT) GP from Zaire [GP (Z)] species of Ebola virus.

For the MVA-EbolaZ vaccine, an attenuated poxvirus strain, modified vaccinia virus Ankara (MVA), was used as a vector. The MVA was derived from a smallpox vaccine strain that after more than 570 passages in primary chicken embryo fibroblast (CEF) cells lost about 12% of the genome and became defective for replication in most mammalian cells [15-17]. In human cells, MVA produces both early and late viral proteins, but the viral assembly and thus production of infectious progeny and cell-to-cell viral transmission are impaired [16, 18]. The first successful attempt to use MVA as a vector for expression of foreign proteins in human cells was reported in 1992 [18]. Since then, MVA vector is being used in investigational vaccines against infectious diseases and cancer. Many of these investigational vaccines have been tested in Phase 1 clinical trials. As a result of these studies, the safety of MVA vector is well documented [16, 17].

The rationale for development of the MVA-EbolaZ vaccine is based on previous human experience with other investigational Filovirus vaccines (Section 1.2.1), previous human experience with the MVA vaccines (Section 1.2.2), and preclinical studies of vaccine candidates against Filoviruses (Section 2.3).

A vaccination strategy to achieve protective immunity in most recipients with a single vaccination would be desirable in an outbreak setting. Vaccination strategies that achieve durable protective immunity would be desirable for populations in areas of the world where outbreaks occur sporadically. Optimally, one approach should serve both needs, but a different approach may be needed for rapid immunity than is needed for durable immunity. The ongoing VRC 207 Phase 1 study is primarily directed at assessing safety and immunogenicity of the cAd3-vectored Ebola vaccines (cAd3-EBO and cAd3-EBOZ) that may provide rapid immunity with a single injection. Responding to the current Ebola outbreak in Western Africa, evaluation of the cAd3-EBO and cAd3-EBOZ vaccines in phase 1 studies has been greatly accelerated.

In animal testing, the cAd3-EBO vaccine demonstrated 100% efficacy in a non-human primate (NHP) challenge model at 5 weeks after a single injection, and partial efficacy at 10 months. In further NHP studies, boosting cAd3-EBO-primed animals at 8 months with recombinant MVA encoding WT GP increased the longevity of immune responses and resulted in durable 100% protection from challenge at 10 months [19].

Therefore, the VRC 208 study will assess safety and immunogenicity of the MVA-EbolaZ vaccine alone or administered as a booster to the cAd3-vectored Ebola vaccines. The prime-boost regimen may provide rapid immunity with a prime injection and more durable protection after the boost.

There is no prior human experience with the MVA-EbolaZ vaccine.

1.2.1. Previous Human Experience with VRC Filovirus Vaccines

Prior to 2014, VRC/NIAID had evaluated three different investigational Ebola vaccines and one Marburg virus vaccine, which were evaluated as safe, well tolerated and immunogenic in a series of four Phase 1 human clinical trials that are listed in the first 4 rows of Table 1.

The next generation of the VRC Ebola vaccines, cAd3-vectored VRC-EBOADC069-00-VP (cAd3-EBO) and VRC-EBOADC076-00-VP (cAd3-EBOZ), began evaluation in 2014 in the VRC 207 study as indicated in the final row of Table 1. The cAd3-vectored vaccines are also being evaluated in a series of studies being conducted by others in European and African countries.

Table 1. Summary of the VRC Studies for Evaluation of Ebola Vaccines

Study Identifier (Clinicaltrials.gov)	Study Design	Vaccine Product(s)	Dosage, route, x N administrations	Accrual* Product/ Placebo
VRC 204 [20] (NCT00072605), completed	Phase I, randomized, placebo-controlled, dose escalation	VRC-EBODNA012-00-VP (Ebola DNA, ΔTM GP)	2 mg IM x 3 doses<br>4 mg IM x 3 doses<br>8 mg IM x 3 doses	**5**/2<br>**8**/2<br>**8**/2
VRC 205 [21] (NCT00374309), completed	Phase I, randomized, placebo-controlled, dose escalation	VRC-EBOADV018-00-VP (Ebola-rAd5, PM GP)	$2x10^9$ VP† IM x 1 dose<br>$2x10^{10}$ VP IM x 1 dose	**12**/4<br>**12**/4
VRC 206 [22] (NCT00605514), completed	Phase I, open label	VRC-EBODNA023-00-VP (Ebola DNA, WT GP)<br>VRC-MARDNA025-00-VP (Marburg DNA, WT GP)	4 mg IM x 3 or 4 doses<br>4 mg IM x 3 or 4 doses	**10**/0<br>**10**/0
RV 247 [23] (NCT00997607), completed	Phase Ib, randomized, placebo-controlled	VRC-EBODNA023-00-VP<br>VRC-MARDNA025-00-VP<br>VRC-EBODNA023-00-VP and VRC-MARDNA025-00-VP	4 mg IM x 3 doses<br>4 mg IM x 3 doses<br>4 mg IM x 3 doses; both vaccines	**30**/6<br>**30**/6<br>**30**/6
VRC 207 [24] (NCT02231866), in progress	Phase I/Ib, open label	VRC-EBOADC069-00-VP (cAd3-EBO, WT GP)<br>VRC-EBOADC076-00-VP (cAd3-EBOZ, WT GP)	$2x10^{10}$ PU^ IM x 1dose<br>$2x10^{11}$ PU IM x 1 dose<br>$1x10^{10}$ PU IM x 1 dose<br>$1x10^{11}$ PU IM x 1 dose	**10**/0<br>**ongoing (>60)**/0<br>**10**/0<br>**10**/0

* number of subjects who received Ebola vaccine is shown in bold
† VP - viral particles
^ PU - particle units

Each completed clinical trial has contributed to product development and a better understanding of human immune responses to investigational vaccines. The first investigational vaccine study was initiated in 2003. This was a 3-plasmid recombinant DNA vaccine, VRC-EBODNA012-00-

VP, that encoded nucleoprotein (NP) from the Zaire strain of Ebola and transmembrane deleted (ΔTM) Ebola GP sequences from Zaire and Sudan strains. At that time, deletion of the TM region of the GP was included in the vaccine construct design to address theoretical concerns related to cellular toxicity that had been observed during *in vitro* experiments when plasmids expressing full length, WT GP proteins were transfected into cell culture [25].

The second investigational vaccine evaluated was a replication-defective, recombinant adenovirus serotype 5 vaccine, VRC-EBOADV018-00-VP (Ebola-rAd5). The constructs in this vaccine encoded GP genes with a point-mutation [PM, aspartic acid substituted for glutamic acid at position 71 (E71D)], which had been found to be safe and immunogenic in animal studies without *in vitro* or *in vivo* toxicity. The design of this vaccine was based on observation of complete protection in macaques vaccinated with a rAd vaccine encoding for E71D GP (Z) combined with E71D GP (S) and then challenged with Ebola Zaire species [9]. The Ebola-rAd5 vaccine was immunogenic and induced humoral and T cell responses to the point mutant GP inserts with a single vaccination, but pre-existing immunity to Ad5 partially blunted antibody responses to the vaccine antigen [21].

Non-human primate (NHP) studies, ongoing in a similar timeframe to the conduct of these human clinical trials, showed that ΔTM GP and PM GP antigens were partially protective against Ebola virus infection, but WT GP constructs provided the highest level of protection [26]. Therefore, the WT GP antigen became the subsequent focus of Ebola vaccine research and development at VRC/NIAID. The third investigational Ebola vaccine, VRC-EBODNA023-00-VP (Ebola DNA WT), was a 2-plasmid recombinant DNA vaccine encoding for the WT GP of the Zaire and Sudan species of Ebola virus. At the same time, a DNA vaccine encoding for GP of the Angola strain of Marburg virus, VRC-MARDNA025-00-VP (Marburg DNA), was also evaluated. The plasmid DNA vaccines encoding for Ebola WT GP and Marburg WT GP that were evaluated in both the VRC 206 and RV 247 studies were safe and immunogenic but repetitive vaccination with 3 or 4 doses was needed to achieve high response rates to some of the antigens. Importantly, the evaluation of WT GP constructs has not been associated with coagulopathy or serious adverse events.

The investigational vaccines listed in Table 1 were assessed as safe and well tolerated in the completed Phase 1 clinical trials.

The use of recombinant non-replicating cAd3 as a vector for the next generation of Ebola vaccines is based on the observation that this vector is highly potent, and there is low pre-existing immunity to this vector in human populations.

Human clinical trials with VRC-EBOADC069-00-VP (cAd3-EBO) and VRC-EBOADC076-00-VP (cAd3-EBOZ) vaccines began on September 2, 2014 in the VRC 207 study. As of January 2015, 10 subjects have received $1x10^{10}$ PU and 10 subjects have received $1x10^{11}$ PU of cAd3-EBOZ, while 10 subjects have received $2x10^{10}$ PU and administration of $2x10^{11}$ PU cAd3-EBO is ongoing in the VRC 207 study. The safety and immunogenicity assessments for 20 subjects who received cAd3-EBO in dosages up to $2x10^{11}$ PU in this study have been described in a preliminary report [24]. Greater than 200 subjects have received the cAd3-EBOZ vaccine in ongoing international studies.

There have been no serious adverse events or clinical evidence of coagulopathy. Of note, prolonged PTT is an expected adverse event for adenoviral vector vaccines and is described in

the Investigator's Brochure as an *in vitro* effect on the lab assay for PTT. Nine (12.9%) of the first 70 subjects in the VRC 207 study had asymptomatic prolonged PTT laboratory results at 2 weeks after vaccination. Evaluation of these prolonged PTT adverse events (AEs) showed these to be consistent with the induction of an antiphospholipid antibody (APA) and not of a coagulopathy. We have observed comparable cases in other adenoviral vector vaccine studies and note that this typically occurs about 2 weeks after vaccination in a subset of vaccine recipients. The APA effect on the PTT test resolves over several weeks without any clinical side effects.

The $2x10^{11}$ PU dose of cAd3-EBO vaccine is associated with more reactogenicity than the $2x10^{10}$ PU dose and this pattern is similar to other adenoviral vector vaccines. The early data suggest that about 60% of subjects will have at least one systemic and/or local reactogenicity symptom. Based on data as of 12/1/2014, the systemic reactogenicity typically occurs within a day after vaccination and may include headache, malaise, myalgia, chills, and fever; about 30% of subjects in the higher dose group experienced mostly mild or moderate fever. When present, fever onset was within one day of vaccination and resolved within 24 hours of onset. A pattern of fever, occurring later than one day after vaccination or lasting longer than a day, may require evaluation for additional etiology [24].

Evaluation of cAd3-vectored Ebola vaccines is ongoing in VRC 207 as well as in international studies being conducted by others. To date, both the VRC-EBOADC069-00-VP (cAd3-EBO) and VRC-EBOADC076-00-VP (cAd3-EBOZ) are evaluated as well tolerated and safe for further evaluation in humans at dosages up to $2x10^{11}$ PU and $1x10^{11}$ PU, respectively.

1.2.2. Previous Human Experience with Modified Vaccinia Virus Ankara (MVA) and MVA-vectored Vaccines

The highly attenuated poxvirus strain modified vaccinia virus Ankara (MVA) has been used as a vector delivery system and a vaccine candidate for multiple pathogens in phase I/II clinical trials [16]. The MVA and recombinant MVA-vectored vaccines have been shown to be safe and immunogenic in human studies evaluating candidate vaccines for Variola major (smallpox) [27-30], HIV [31, 32], and influenza [33, 34] in adults, and a tuberculosis vaccine candidate in infants [35].

A new smallpox vaccine based on the MVA (Imvanex, Bavarian Nordic, Denmark) was granted marketing authorization in the European Union in 2013 [36]. Imvanex is the first smallpox vaccine licensed for active immunization of the general adult population, including those with atopic dermatitis and weakened immune systems. The product is also licensed in Canada where it is marketed as Imvamune. Imvamune is undergoing additional evaluation in studies conducted in the US (ClinicalTrials.gov, NCT01827371).

The MVA-vectored vaccines and chimpanzee adenovirus-vectored vaccines have been administered in investigational prime-boost regimens targeted against malaria [37-39]. In the clinical assessment of cAd63-vectored malaria vaccine expressing multiple epitope thrombospondin-related adhesion protein (ME-TRAP) in Phase I study, 54 healthy volunteers had vaccine administered alone intradermal (ID) or IM with the doses escalated up to $2x10^{11}$ VP (n=28), or followed with a MVA booster in 8 weeks at the $2x10^{8}$ plaque-forming units (PFU) dose (n=26). The cAd63-vectored vaccine prime followed by MVA boost appeared to be safe and highly immunogenic [40].

Based on human clinical trial experience with MVA-vectored vaccines, human clinical trial experience with cAd3-vectored vaccines, and the pre-clinical assessment of cAd3-vectored vaccine prime with MVA-vectored vaccine boost [19], the prime-boost approach to delivering encoded Ebola WT GP offers a promising approach for achieving more durable immunity and will be evaluated in the VRC 208 study.

1.3. Assessment of Immunogenicity

Immunogenicity of the study vaccines will be evaluated as humoral and cellular immune responses assessed by ELISA and intracellular cytokine staining (ICS) assays. Other measures of immunogenicity may be also applied as exploratory evaluations.

The ELISA and ICS assays will be performed at the VRC or at the NIAID Vaccine Immune T-Cell and Antibody Laboratory (NVITAL), and the exploratory evaluation of Ebola-specific neutralizing antibodies will be conducted at the Laboratory of Immunology, Biodefense Research Section, VRC/NIAID, using previously published methods [21].

The ICS assay quantitates the frequency of CD4+ and CD8+ cells that produce interleukin-2, interferon-gamma and/or tumor necrosis factor in response to pools of overlapping peptides representing Ebola GP antigens and is based upon previously published methods [41].

As an exploratory evaluation, specific peptides may also be used to detect T and B cell responsiveness by an enzyme-linked immunospot (ELISpot) assay, modified from a previously published method [42]. The pre-existing and post-vaccination presence of cAd3-specific antibodies also will be evaluated as an exploratory objective [43]. Feasibility for the evaluation of MVA-specific antibodies will be considered pending the identification, availability and/or development of a suitably sensitive assay.

Stored samples from vaccine study subjects may also be used in the future for evaluation of immune response to the vaccine as measured by other assays. The experimental laboratory procedures for the exploratory objectives may be conducted using fresh or stored samples at the NIH, contract laboratories, or at the laboratories of NIH affiliates and/or collaborators.

2. BACKGROUND ON VACCINE

2.1. Description of the Study Agents

VRC-EBOMVA079-00-VP: Recombinant live modified vaccinia virus Ankara (MVA) Ebola vaccine, VRC-EBOMVA079-00-VP (MVA-EbolaZ), consists of the attenuated replication-defective orthopoxvirus vector, MVA, that expresses Ebola Zaire WT GP. The vaccine was manufactured per VRC directive at IDT Biologika GmbH, Dessau, Germany under contract to Advent s.r.l, Pomezia, Italy and managed by the Vaccine Clinical Materials Program (VCMP), Leidos Biomedical Research, Inc., Frederick, MD. The vaccine is a homogeneous suspension composed of MVA-EbolaZ drug substance filled into single dose vials at $3.2x10^8$ PFU/mL.

VRC-EBOADC069-00-VP: Recombinant chimpanzee adenovirus Type 3-vectored Ebola vaccine, VRC-EBOADC069-00-VP (cAd3-EBO), consists of a 1:1 ratio of two drug substances: recombinant replication-deficient adenovirus chimpanzee serotype 3 (cAd3) vectors expressing WT Ebola GPs from Zaire (EBO Z) and Sudan (EBO S) strains. The vaccine was manufactured

at the VRC Vaccine Pilot Plant (VPP) operated under contract by Vaccine Clinical Materials Program (VCMP), Leidos Biomedical Research, Inc., Frederick, MD. The vaccine is a sterile, aqueous, buffered solution composed of 1:1 ratio of cAd3 EBO GP Zaire and cAd3 EBO GP Sudan drug substances filled into single dose vials at $1x10^{11}$ PU/mL each ($2x10^{11}$ PU/mL total).

VRC-EBOADC076-00-VP (cAd3-EBOZ) product will not be administered in VRC 208; the rollover subjects in Groups 4 and 5 will have already received cAd3-EBOZ in VRC 207. Additional information on this product can be found in the Investigator's Brochure (IB).

2.2. Preclinical Studies Supporting Safety of cAd3 and MVA Constructs

The March 2014 Ebola outbreak in West Africa [13] prompted the VRC/NIAID to accelerate initiation of the first clinical study of VRC-EBOADC069-00-VP. The vaccine vector, cAd3, was used in an investigational HCV vaccine product, AdCh3-HCV (BB-IND 14818, Okairos, Inc.), for which both nonclinical and clinical evaluations were performed by others and a nonclinical toxicology study with VRC cAd3-HIV Vaccine (VRC-HIVADC064-00-VP) was performed by VRC/NIAID. In addition, VRC/NIAID evaluated in both a GLP toxicity study and two Phase 1 clinical trials, a plasmid DNA vaccine, VRC-EBODNA023-00-VP (BB-IND 13609, RCHSPB), which encodes the same Ebola Zaire and Sudan WT glycoproteins used in the cAd3-EBO vaccine.

In April 2014, the VRC/NIAID proposed to the FDA that these prior nonclinical and clinical studies support evaluation of cAd3-EBO through a staged, dose escalation Phase 1 study design without the conduct of a GLP toxicity study. The FDA had previously reviewed product information and testing at a pre-IND meeting conducted in August 2013. In May 2014, the FDA concurred with the VRC proposal to proceed with evaluation of the cAd3-EBO vaccine in a Phase 1 clinical trial and no preclinical GLP toxicology study was conducted. Prior relevant nonclinical and clinical studies are summarized in the Investigator's Brochure.

In August 2014 the IND to begin evaluation of cAd3-EBO was reviewed and assessed as safe to proceed by the FDA. On the basis of consultation with collaborating researchers the VRC/NIAID also sought and received approval to evaluate the cAd3 EBO GP Zaire drug substance alone as a vaccine under the same IND. This is designated VRC-EBOADC076-00-VP (cAd3-EBOZ).

Since significant experience has been accumulated in humans with the MVA vector (Section 1.2.2), and with the Ebola GP inserts (Section 1.2.1), no additional preclinical GLP toxicology studies were conducted.

2.3. Nonclinical Immunogenicity and Protection Studies of cAd3 and MVA Constructs

Several non-GLP studies were performed in NHP, Cynomolgus macaques, to select the cAd3-based and MVA-based constructs for further development and to provide animal proof-of-concept data necessary to enter Phase 1 clinical studies. Research-grade materials, made with the same constructs as clinical material, of cAd3 EBO Z (containing GP from Ebolavirus Zaire), cAd3 EBO S (containing GP from Ebolavirus Sudan), cAd3-EBO (composed of a 1:1 ratio of cAd3 EBO Z and cAd3 EBO S) and/or MVA encoding GPs from Zaire and Sudan species of Ebola were used in all studies summarized in Table 2.

Evaluation of the prime-boost regiment of MVA-vectored Ebola vaccine administered as a boost to the cAd3-vectored Ebola vaccine prime demonstrated that durable protection has been achieved against lethal EBOV challenge in Cynomolgus macaques. Animals that received a single IM dose of MVA-based vaccine had detectable viremia by day 6 after challenge and none survived infection [19].

Table 2. Preclinical Proof-of-Concept Studies in Cynomolgus Macaques

Study Purpose	Study Outcome
Demonstrate protection against lethal challenge with Ebolavirus Zaire in Cynomolgous macaques after single IM dose of cAd3 EBOZ	100% protection after a single IM dose of cAd3 EBO Z at 10^{10} and 10^{11} VP*
Demonstrate protection against lethal challenge with Ebolavirus Zaire in Cynomolgous macaques after single IM dose of cAd3-EBO	100% protection after a single IM dose of cAd3-EBO at $2x10^{10}$ VP<br>50% protection after cAd3-EBO at $2x10^{9}$ VP
Demonstrate protection against lethal challenge with Ebolavirus Sudan in Cynomolgous macaques after single IM dose of cAd3-EBO	100% protection against Ebolavirus Sudan after a single IM dose of cAd3-EBO at $2x10^{10}$ VP
Demonstrate generation of humoral and cellular immune responses after single IM dose of cAd3-EBO at $2x10^{9}$ or $2x10^{10}$ VP.	Single IM dose of cAd3-EBO at $2x10^{9}$ VP or $2x10^{10}$ VP elicited antibody and antigen-specific CD4+ and CD8+ T cell responses. The lower cellular immune responses correlated with the lower level of protection (50%) observed after the $2x10^{9}$ VP dose.
Demonstrate durability of the immune responses after single IM dose of cAd3 EBO Z at 10^{11} VP or cAd3-EBO at $2x10^{10}$ VP administered 10 months before the lethal challenge with Ebolavirus Zaire.	Protective immune responses measured during the acute phase of infection declined over time. When challenged 10 months after vaccination, 2/4 macaques were protected after a single IM dose of cAd3 EBO Z at 10^{11}VP while 0/4 were protected after a single IM dose of cAd3-EBO at $2x10^{10}$VP.
Demonstrate protection against lethal challenge with Ebolavirus Zaire in Cynomolgous macaques after prime with single IM dose of cAd3-EBO and week 8 boost with single IM dose of MVA (expressing GPs from EBOV and SUDV).	100% protection after a prime with a single IM dose of cAd3-EBO at 10^{10} VP and week 8 boost with a single IM dose of MVA (expressing GPs from EBOV and SUDV) at 10^{8} PFU**.
*VP – Viral Particles<br>**PFU – Plaque-Forming Unit	

3. STUDY OBJECTIVES

3.1. Primary Objectives

- To evaluate the safety and tolerability of VRC-EBOMVA079-00-VP when administered IM at a dose of 1×10^{7} plaque forming units (PFU) to healthy adults;

- If a dose of 1 x 10^7 PFU administered IM is safe and well tolerated, to evaluate the safety and tolerability of VRC-EBOMVA079-00-VP administered IM at a dose of 1 x 10^8 PFU to healthy adults.
- If VRC-EBOMVA079-00-VP administered IM at a dose of 1 x 10^8 PFU is safe and well tolerated, to evaluate the safety and tolerability of the prime-boost regimens.

3.2. Secondary Objectives

- To evaluate the antibody response to VRC-EBOMVA079-00-VP at 4 weeks after vaccination as assessed by Ebola GP-specific ELISA and neutralization assays.
- To evaluate the Ebola GP-specific T cell responses to VRC-EBOMVA079-00-VP at 4 weeks after vaccination as assessed by ICS.
- To evaluate the antibody response to the cAd3-EBO/EBOZ prime - MVA-EbolaZ boost regimen at 4 weeks after the boost as assessed by Ebola GP-specific ELISA and neutralization assays.
- To evaluate the Ebola GP-specific T cell responses to the cAd3-EBO/EBOZ prime - MVA-EbolaZ boost regimen at 4 weeks after the boost as assessed by ICS.

3.3. Exploratory Objectives

- To evaluate the immunogenicity of VRC-EBOMVA079-00-VP administered alone and in the cAd3-EBO/EBOZ prime - MVA-EbolaZ boost regimen by various assay methods at some or all of the research sample collection timepoints indicated in the Schedule of Evaluations; genetic factors associated with immune response may also be evaluated.
- To evaluate if different prime-boost intervals would influence the immunogenicity in subjects previously primed by vaccination with the cAd3-EBO and cAd3-EBOZ vaccines in VRC 207 study.
- To evaluate vaccine-induced mRNA expression profiles through 1 week after each vaccination.
- To evaluate the cAd3- and MVA-specific antibodies prior to vaccination and at 4 weeks post vaccination.
- To evaluate the time course and durability of cAd3 and MVA neutralizing antibody titers, mediators of inflammation following vaccination, and time course and durability of immune response by a variety of exploratory assays using samples collected throughout the study.

4. STUDY DESIGN AND METHODS

This is a two-part Phase 1/1b, open-label, dose escalation clinical trial to evaluate safety, tolerability, and immunogenicity of the Ebola modified vaccinia virus Ankara vaccine (MVA-EbolaZ) administered alone or as a boost to the cAd3-EBO or cAd3-EBOZ vaccines in healthy adults. The hypotheses are that the study vaccine, MVA-EbolaZ, will be safe and well-tolerated, will elicit immune responses to Ebola GP, and that the prime-boost regimens will be safe and

will result in a polyfunctional response to Ebola GP that is of greater magnitude and duration than response to either of the vaccines alone.

The study will be initiated with Part 1 at VRC NIH CC where Groups 1, 2 and 3 will be enrolled. If needed, another study site may participate in the completion of enrollments into these groups. Part 2 will be conducted as a multicenter clinical trial as shown on the schema in Table 3. Part 2 will be conducted at all three VRC 207 sites: the VRC NIH CC, The Hope Clinic of the Emory Vaccine Center, and the University of Maryland Center for Vaccine Development, where the VRC 207 study participants were enrolled.

Table 3. VRC 208 Study Schema

VRC 208 Schema				
**Group**	**Subjects**	**Day 0**	**Week 8 Boost**	**Study site**
1	5	MVA-EbolaZ (1x10^7 PFU)		VRC
2	5	MVA-EbolaZ (1x10^8 PFU)		VRC
3	10*	cAd3-EBO (2x10^{11} PU)	MVA-EbolaZ (1x10^8 PFU)	VRC
**Part 2: Evaluation of MVA-EBOLAZ boost for VRC 207 subjects**				
**Group**	**Subjects**	**Prime received in VRC 207**	**Day 0 Boost**	
4	up to 10	cAd3-EBOZ (1x10^{10} PU)	MVA-EbolaZ (1x10^8 PFU)	UMD
5	up to 10	cAd3-EBOZ (1x10^{11} PU)	MVA-EbolaZ (1x10^8 PFU)	UMD
6	up to 10	cAd3-EBO (2x10^{10} PU)	MVA-EbolaZ (1x10^8 PFU)	VRC
7	up to 110	cAd3-EBO (2x10^{11} PU)	MVA-EbolaZ (1x10^8 PFU)	VRC, Emory
Total	up to 160	All injections are IM in deltoid muscle with needle and syringe. For Groups 4-7, the interval of time between the prime received in VRC 207 and the boost in VRC 208 will vary, but will be at least 12 weeks.<br>*After the randomization of 10 subjects into Group 3, up to 10 additional subjects may be enrolled. The total accrual to the study will not exceed 160.		

Part 1 will begin with 15 Ebola vaccine-naive subjects randomized at the VRC NIH CC site to either Group 1 or Group 3 in a 1:2 ratio. Group 1 subjects will receive MVA-EbolaZ at 1x10^7 PFU and Group 3 subjects will begin a cAd3-EBO prime regimen. When criteria for dose escalation are met in Group 1, enrollment of vaccine-naive subjects into Group 2 may begin. If the randomization of all 15 subjects needed to fully enroll both Group 1 and Group 3 is not yet completed, the remaining randomizations may be completed after Group 2 is fully enrolled in order to facilitate the prompt safety evaluation of the higher dose level.

Group 2 subjects will receive MVA-EbolaZ at 1x10^8 PFU. If MVA-EbolaZ at 1x10^8 PFU is assessed as safe for further evaluation, then 3 subjects on a prime-boost schedule will receive a booster injection with MVA-EbolaZ at 1x10^8 PFU; these 3 pilot subjects may be from either Group 3 or Group 7. An interim safety review will occur once the first 3 subjects boosted with MVA-EbolaZ have completed 7 days of follow-up. This interim safety review will occur before additional subjects are boosted in Groups 3 through 7. Group 3 subjects will receive the MVA-EbolaZ boost 8 weeks after the cAd3-EBO prime. **Part 1** includes a staged enrollment plan with required interim safety reviews before proceeding to the next step as detailed in Section 4.3. The

interim safety reviews will be conducted by the Protocol Safety Review Team (PSRT, Section 8.9).

Enrollment of **Part 2** groups may occur concurrently as VRC 207 study schedule permits for rollover subjects who will be required to complete at least 12 weeks of follow-up on VRC 207. To control and unify the prime-boost intervals for data analysis, to the degree possible within Group 7, the enrolling site will attempt to target the timing of the rollover into VRC 208 to fit within the subgroups with 12 (+2) week, 16 (+2) week, and 24 (+4/-1) week intervals between the prime and the boost.

4.1. Study Population

Healthy, vaccine-naive adults for Groups 1, 2 and 3 will be recruited through Institutional Review Board (IRB)-approved advertising and screened through the VRC 500 (11-I-0164) screening protocol. The rollover subjects in Groups 4-7 will be invited to participate by site personnel at the site where they are enrolled in VRC 207.

4.1.1. Inclusion Criteria for Groups 1, 2, and 3.

A volunteer must meet all of the following criteria to be eligible:

1. 18 to 50 years old.
2. Available for clinical follow-up through the last study visit.
3. Able to provide proof of identity to the satisfaction of the study clinician completing the enrollment process.
4. Able and willing to complete the informed consent process.
5. Willing to donate blood for sample storage to be used for future research.
6. In good general health without clinically significant medical history.
7. Physical examination and laboratory results without clinically significant findings and a body mass index (BMI) ≤ 40 within the 56 days prior to enrollment.

   Laboratory Criteria within 56 days prior to enrollment:

8. Hemoglobin within institutional normal range or accompanied by the Principal Investigator (PI) or designee approval.
9. White blood cells (WBC) = 3,300-12,000 cells/mm^3.
10. WBC differential either within institutional normal range or accompanied by the PI or designee approval.
11. Total lymphocyte count ≥ 800 cells/mm^3.
12. Platelets = 125,000 – 400,000/mm^3.
13. Alanine aminotransferase (ALT) ≤ 1.25 x upper limit of normal.
14. Serum creatinine ≤ 1.1 x upper limit of normal.
15. Partial thromboplastin time (PTT) ≤ 1.1 x upper limit of normal or accompanied by the Principal Investigator (PI) or designee approval.

16. Prothrombin time (PT) ≤ 1.1 x upper limit of normal or accompanied by the Principal Investigator (PI) or designee approval.
17. HIV-uninfected as evidenced by a negative FDA-approved HIV diagnostic blood test.

Female-Specific Criteria:

18. Negative β-HCG (human chorionic gonadotropin) pregnancy test (urine or serum) on day of enrollment if woman is presumed to be of reproductive potential.
19. Agrees to use an effective means of birth control from at least 21 days prior to enrollment through 24 weeks after last study vaccination if presumed to be of reproductive potential.

4.1.2. Exclusion Criteria for Groups 1, 2, and 3

A volunteer will be excluded if one or more of the following conditions apply:

Volunteer has received any of the following substances:

1. Investigational Marburg vaccine in a prior clinical trial.
2. Investigational Ebola vaccine in a prior clinical trial.
3. Investigational cAd3 or MVA vaccines in a prior clinical trial.
4. Evidence of increased cardiovascular disease risk defined as >10% five year risk by the non-laboratory method [44].
5. Electrocardiogram (ECG) with clinically significant abnormalities (examples may include: pathologic Q waves, significant ST-T wave changes, left ventricular hypertrophy, any non-sinus rhythm excluding isolated premature atrial contractions, right or left bundle branch block, advanced A-V heart block). ECG abnormalities determined by a cardiologist to be clinically insignificant as related to study participation do not preclude study enrollment.
6. Type 1 hypersensitivity reaction to aminoglycoside antibiotics.
7. More than 10 days of systemic immunosuppressive medications except for short-term treatments of minor ailments in otherwise healthy volunteers, or cytotoxic medications within the 4 weeks prior to enrollment, or any within the 14 days prior to enrollment.
8. Blood products within 112 days (16 weeks) prior to enrollment.
9. Investigational research agents within 28 days (4 weeks) prior to enrollment.
10. Live attenuated vaccines within 28 days (4 weeks) prior to enrollment.
11. Medically indicated subunit or killed vaccines, e.g. influenza, pneumococcal within 2 weeks of initial study vaccine administration unless approved by the study Principal Investigator (PI) or designee
12. Current anti-tuberculosis prophylaxis or therapy.

Female-specific criteria:

13. Woman who is breast-feeding or planning to become pregnant during the 24 weeks of

study participation.

Volunteer has a history of any of the following clinically significant conditions:

14. Serious adverse reactions to vaccines such as anaphylaxis, urticaria (hives), respiratory difficulty, angioedema, or abdominal pain.
15. Clinically significant autoimmune disease or immunodeficiency.
16. Asthma that is not well controlled.
17. Diabetes mellitus (type I or II), with the exception of gestational diabetes.
18. Thyroid disease that is not well controlled.
19. A history of hereditary angioedema (HAE), acquired angioedema (AAE), or idiopathic forms of angioedema.
20. Idiopathic urticaria within the last 1 year.
21. Hypertension that is not well controlled.
22. Bleeding disorder diagnosed by a doctor (e.g. factor deficiency, coagulopathy, or platelet disorder requiring special precautions) or significant bruising or bleeding difficulties with IM injections or blood draws.
23. Malignancy that is active or history of a malignancy that is likely to recur during the period of the study.
24. Seizure in the past 3 years or treatment for seizure disorder in the past 3 years.
25. Asplenia or functional asplenia.
26. Psychiatric condition that precludes compliance with the protocol; past or present psychoses; or within five years prior to enrollment, history of a suicide plan or attempt.
27. Any medical, psychiatric, social condition, occupational reason or other responsibility that, in the judgment of the investigator, is a contraindication to protocol participation or impairs a volunteer’s ability to give informed consent.

4.1.3. Rollover Criteria for Groups 4, 5, 6, and 7.

Each rollover subject must have received the VRC-EBOADC069-00-VP (cAd3-EBO) or the VRC-EBOADC076-00-VP (cAd3-EBOZ) vaccine in the VRC 207 study, must have completed at least 12 weeks of follow-up, and must be assessed as eligible. Eligibility assessments are based on knowledge of subject health and medical history from VRC 207.

The following inclusion criteria apply to rollover subjects:

1. The subject is willing to participate in VRC 208.
2. By clinical judgment of the site PI or designee, the subject is in good general health without clinically significant medical history that precludes study participation.

The following exclusion criteria apply to rollover subjects:

1. Type 1 hypersensitivity reaction to aminoglycoside antibiotics.

An ECG will be performed after enrollment into VRC 208 at Visit 01R. The subject may be

excluded from receiving the MVA-EbolaZ vaccine for ECG with clinically significant abnormalities (examples may include: pathologic Q waves, significant ST-T wave changes, left ventricular hypertrophy, any non-sinus rhythm excluding isolated premature atrial contractions, right or left bundle branch block, advanced A-V heart block). ECG abnormalities determined by a cardiologist to be clinically insignificant as related to study participation do not preclude receipt of MVA-EbolaZ.

If a subject is unable to receive the MVA-EbolaZ vaccine in VRC 208, the subject will be marked as off-study for VRC 208 and may continue with an on-study status for VRC 207 to complete any remaining VRC 207 visits.

4.2. Clinical Procedures and Schedule of Evaluations

This section describes the clinical procedures for evaluating study participants and follow-up after administration of study vaccine.

4.2.1. Screening

Part 1 Subjects: Eligibility screening for the vaccine-naive subjects in this study (Part 1: Groups 1 through 3) will be completed through the VRC screening protocol, VRC 500 (NIH 11-I-0164).

Testing will be done according to eligibility criteria and clinical assessment at screening as per Appendix 3 of the protocol. Screening evaluations for specific eligibility criteria (see Sections 4.1.1 and 4.1.2) must be completed within the time interval specified prior to enrollment for the given parameter, but may be repeated as needed to confirm eligibility. Research blood samples will also be collected and stored during screening; a particular interval of time prior to enrollment for collection of these samples is not required. Informed consent documents for vaccine trials will be reviewed, counseling related to the potential risks of becoming pregnant during study participation will be provided and an Assessment of Understanding (AoU) will be completed prior to enrollment into VRC 208. Any study volunteer who has not yet received the current year influenza vaccination may receive this, if available, during participation in the screening protocol.

Part 2 Subjects: Assessment of eligibility for rollover subjects who enroll into Part 2 of the study will be performed through evaluations conducted during participation on VRC 207. Clinical judgment will be used for eligibility evaluation of these subjects, and each rollover case will be approved by the site PI or designee. An Assessment of Understanding (AoU) will be completed prior to enrollment into VRC 208. A baseline ECG will be performed at Visit 01R to assess for eligibility to receive the VRC 208 study vaccine. If the subject receives the VRC 208 study vaccine, the subject will be off-study for VRC 207. If the subject does not receive the VRC 208 study vaccine for any reason, the subject may continue with on-study status for VRC 207 to complete any remaining VRC 207 visits.

For both Part 1 and Part 2 subjects, information on previous exposure to smallpox and MVA vaccines will be collected at screening or at Visit 01R.

4.2.2. Study Schedule and Enrollment

The schedule of study visits, permitted windows for completing the visits, and evaluations performed at each visit are shown in Appendix 3. For all groups, the enrollment day is designated Visit 01R on the Schedule of Evaluations (Appendix 3). Day 0 (Visit 02) is defined as the day of study injection. The Schedule of Evaluations allows an interval of time between Visit 01R and Visit 02 but these two visits may occur on the same date.

Since the first 15 vaccine-naive subject enrolled will be randomized into Groups 1 and Group 3, randomization may occur rapidly but the subjects may be scheduled to receive their study vaccination at a later date in order to adhere to the protocol rule that no more than one vaccination per day may occur for the first 3 subjects in Group 1. Protocol-specific eligibility is reviewed as part of the enrollment process. Eligibility evaluations conducted during a screening visit (VRC 500) are routinely used for eligibility if the screening occurred within the specified window prior to the enrollment visit. If one or more subjects are enrolled that do not receive the study vaccination, additional subjects may be enrolled in the same group in order to meet the study objectives.

Day 0 evaluations prior to study vaccination are the baseline for subsequent safety assessments.

Deviations from the visit windows in completing study visits are discouraged, but are permitted at the discretion of the PI (or designee) in the interest of completing the study schedule and obtaining subject safety and immunogenicity data needed for evaluating research results. Deviations will be recorded.

4.2.3. Administration of the Study Injection

On the injection day, before the injection, study subjects will be evaluated by clinical evaluation and samples will be collected for laboratory tests as per the Schedule of Evaluations. Pregnancy test results for women of reproductive potential must be obtained prior to the study injection on Day 0. A subject who arrives at the clinic with fever or evidence of an acute illness, which precludes administration of the vaccine, may be rescheduled to a different date.

Injections will be administered into the deltoid muscle by needle and syringe. It is recommended, but not required, that the injection be administered into the non-dominant arm. When choosing an arm for the injection, clinicians should consider whether there is an arm injury, local skin problem or significant tattoo that precludes administering the injection or will interfere with evaluating the arm after injection. In keeping with the NIH Clinical Center policy and good medical practice, acute medical care will be provided to subjects for any immediate allergic reactions or other injury resulting from participation in this research study. Procedures for follow-up in the clinic after vaccination are shown in Appendix 3.

4.2.4. 7-Day Solicited Reactogenicity and Follow-up

Subjects will be given a “Diary Card” to use as a memory aid, on which to record temperature, local and systemic symptoms and concomitant medications daily for 7 days after the injection. Subjects will be trained to use the secure database or complete the paper diary card depending on their preference. When the diary card parameters are recorded directly by the subject through a password-protected secure database, the subject’s electronic record will be the source for these data. The written (paper) diary card may be used as a source document. When neither a written

nor electronic diary card is available from the subject, the study clinician will note the source of reactogenicity information recorded in the study database.

The solicited signs and symptoms on the diary card will include the following parameters: unusually tired/feeling unwell, muscles aches (other than at injection site), headache, chills, nausea, and pain/tenderness at injection site. Subjects will also record the day's highest measured temperature and measurement of largest diameter for redness and swelling at the injection site.

Follow-up on subject well-being will be performed by telephone on the first or second day following all vaccinations, and by clinic visit on Day 7 following the injection. Diary cards will be reviewed with the clinician at any visits from day of vaccination through the first study visit following completion of the diary card.

Events following injection that may require clinical evaluation include rash, urticaria, fever of 38.5˚C (Grade 2) or higher lasting greater than 24 hours, or significant impairment in the activities of daily living (ADL). Any condition which in the judgment of the clinician should be evaluated would require a clinical visit.

Management of laboratory abnormalities in 4 weeks following vaccination:

The following apply to all Groups: If the PT, aPTT, ALT, creatinine, hemoglobin and/or platelet count are Grade 3 or Grade 4, then repeat testing of the abnormal test will be performed promptly (typically within 48 hours). The specific timing of repeat testing for a Grade 1 or Grade 2 laboratory test change will be determined by the study clinicians as medically appropriate.

Follow-up through the last study visit:

Follow-up will occur via clinical visits through 48 weeks after last vaccination as shown on the Schedule of Evaluation in Appendix 3. At intervals throughout the study, blood will be drawn for safety and immunologic assays. Blood will be drawn from the arm veins of subjects by standard phlebotomy procedures. Total blood volume drawn from each subject will not exceed the NIH Clinical Center Guidelines or local guidelines at the participating sites. Research blood samples will be processed and stored at NVITAL or a collaborating research laboratory. Stored samples may be used later to further evaluate immune responses and to elucidate genetic factors associated with immune response.

4.2.5. Concomitant Medications

Current concomitant medications are recorded in the study database at enrollment. Medications taken during the 7-day period following vaccination will be documented. Subsequently, concomitant medications will be updated in the study database if there is an occurrence of an adverse event that requires expedited reporting or development of a new chronic medical condition that requires ongoing medical management.

Clinicians should work with study subjects with regard to the timing of administration of FDA-approved vaccines. Receipt of a licensed vaccine during study participation will be recorded in the study database. Otherwise, a record of concomitant medication changes throughout the study will not be recorded in the study database.

4.3. Criteria for Dose Escalation and Dose Continuation in Part 1 of the Study

The Protocol Safety Review Team (PSRT) and the IND Sponsor Medical Officer (MO) will conduct the following three interim safety data reviews to assess whether or not the safety data meet criteria for moving on to the next stage of the study:

Review for Dose Escalation to 1 x 10^8 PFU Dose of VRC-EBOMVA079-00-VP: No more than 1 subject per day will be vaccinated for the first 3 subjects in Group 1 at the 1 x 10^7 PFU dose. The two remaining subjects may be vaccinated on the same or different days. When the first 3 subjects who received the 1 x 10^7 PFU dose have completed post vaccination follow up through at least the "Study Week 1 (02D)" visit, an interim safety data review will be conducted by the PSRT. If the safety data support proceeding to the 1 x 10^8 dose, then enrollment of Group 2 may begin.

Review for Continuation of 1 x 10^8 PFU Dose of VRC-EBOMVA079-00-VP and Starting Boost Injections: No more than 1 subject per day will be vaccinated for the first 3 subjects in Group 2 at the 1 x 10^8 PFU dose. The two remaining subjects may be vaccinated on the same or different days. After 3 subjects have completed post vaccination follow up through at least the "Study Week 1 (02D)" visit, an interim safety data review will be conducted by the PSRT. If PSRT assesses the 1 x 10^8 PFU dose as safe for further evaluation, then boost injections may begin. Depending on the study progress, the 3 pilot subjects for the first 3 boost injections may be either Group 3 or Group 7 subjects who received the cAd3-EBO (2 x 10^{11} PU) prime.

Review for Safety of Further Evaluation of 1 x 10^8 PFU of VRC-EBOMVA079-00-VP as a Boost to the cAd3-vectored Ebola vaccines: No more than 1 subject per day will receive a boost at the 1 x 10^8 PFU dose for the first 3 subjects to be boosted. When the 3 subjects who receive the 1 x 10^8 PFU boost have completed post vaccination follow up through at least one week post boost, an interim safety data review will be conducted by the PSRT. The PSRT will conduct an assessment of whether the 1 x 10^8 PFU dose of MVA-EbolaZ vaccine is safe for further evaluation as a boost to the cAd3-vectored Ebola vaccines.

After each of these interim safety reviews, the IND Sponsor, IRB and FDA will be provided with documentation of the safety review and notification of the plan.

4.4. Criteria for Discontinuing Study Injections or Protocol Participation

In general, subjects who receive the Day 0 study injection will continue to be followed for at least 12 weeks of safety follow-up, whenever possible. Decisions on discontinuation of study injections or protocol participation for a subject will be made by the study PI or designee.

4.4.1. Discontinuation of Study Injections

Under certain circumstances, a subject will be terminated from participating in further injections. Participants who are discontinued from additional study injections will continue to be followed according to the schedule of safety and immunogenicity evaluations. Specific events that will require withdrawal of a subject from the injection schedule include:

1. Pregnancy;
2. SAE that is assessed as possibly, probably or definitely related to study injection;

3. Immediate hypersensitivity reaction associated with a study injection;
4. Intercurrent illness that is not expected to resolve prior to the next scheduled study injection assessed by study clinician to require withdrawal from the injection schedule;
5. Treatment with systemic glucocorticoids (e.g., prednisone or other glucocorticoid) or other immunomodulators (other than nonsteroidal anti-inflammatory drugs [NSAIDs]), with the exception that, study vaccination may continue per investigator discretion if the next one occurs at least 2 weeks following completion of glucocorticoid treatment.
6. The study PI assesses that it is not in the best interest of the subject to continue on the vaccination schedule.

4.4.2. Criteria for Discontinuing Study Protocol Participation

A subject may be discontinued from protocol participation for the following reasons:

1. Subject decides to discontinue participation.
2. Subject develops a medical condition that is a contraindication to continuing study participation.
3. The IND sponsor or regulatory authority stops the protocol.
4. The PI assesses that it is not in the best interest of the subject to continue participation in the study or that the subject's compliance with the study is not sufficient.

4.5. Criteria for Pausing the Study

The Principal Investigator will closely monitor and analyze study data as they become available and will make determinations regarding the presence and severity of adverse events. The administration of study injections and new enrollments will be paused and the IND Sponsor will be promptly notified according to the following criteria:

One (or more) subject experiences a **Serious Adverse Event (SAE)** that is assessed as possibly, probably or definitely related to study agent, or

Two (or more) subjects experience the same **Grade 3 or higher** adverse event assessed as possibly, probably or definitely related to study agent, except that pause is not required for self-limited solicited reactogenicity following either vaccine or for asymptomatic prolonged PTT observations following the cAd3-EBO vaccine.

Plan for Review of Pauses and Resuming Rules:

The study injections and enrollments would resume only if review of the adverse events that caused the pause resulted in a recommendation to permit further study injections and study enrollments. The reviews to make this decision will occur as follows:

Pauses for SAEs: The IND Sponsor, with participation by the Principal Investigator, will consult with the FDA to conduct the review and make the decision to resume or close the study for any SAEs that meet the criteria for pausing the study.

Pauses for Grade 3 or higher Events: The IND Sponsor, in consultation with the Principal Investigator, will conduct the review and make the decision to resume or close the study for the

Grade 3 or higher events that meet the criteria for pausing the study. As part of the pause review, the reviewers will also advise on whether the study needs to be paused again for any subsequent Grade 3 or higher events of the same type. The FDA will be notified of pause reviews.

When indicated, safety data reports and changes in study status are submitted to the IRB in accordance with Section 5.4 and institutional policy.

5. SAFETY AND ADVERSE EVENT REPORTING

5.1. Adverse Events

An adverse event (AE) is any untoward or unfavorable medical occurrence in a human subject, including any abnormal sign (e.g., abnormal physical exam or laboratory finding), symptom, or disease temporally associated with the use of study treatment, whether or not considered related to the study treatment.

Each adverse event will be graded according to the table for grading severity of adverse events (see Appendix 4). The following guidelines will be used to determine whether or not an adverse event is recorded in the study database:

Solicited adverse event (*i.e*, reactogenicity parameters) will be recorded in the study database for 7 days after injection without the collection of attribution assessments. Unsolicited AEs of all severities will be recorded in the study database from receipt of each study injection through 28 days after each study injection. At other time periods between injections for Group 3 or when greater than 28 days after the MVA-EbolaZ injection for any group, only SAEs (as defined in Section 5.2) or new chronic medical conditions that require ongoing medical management will be recorded through the last study visit. Because this is an Ebola vaccine study, in the unlikely circumstance of EVD diagnosis in a study subject at any time throughout the study, this will be recorded on an “EVD case report form”, rather than an AE form, without requiring an investigator attribution (“relatedness to study agent”) or severity grade.

5.2. Serious Adverse Events

As defined in 21 CFR 312.32, an adverse event or suspected adverse reaction is considered "serious" if, in the view of either the investigator or sponsor, it results in any of the following outcomes: “Death, a life-threatening adverse drug experience, inpatient hospitalization or prolongation of existing hospitalization, a persistent or significant disability/incapacity, or a congenital anomaly/birth defect. Important medical events that may not result in death, be life-threatening, or require hospitalization may be considered a serious adverse drug experience when, based upon appropriate medical judgment, they may jeopardize the subject or require medical or surgical intervention to prevent one of the outcomes listed in this definition. Examples of such medical events include allergic bronchospasm requiring intensive treatment in an emergency room or at home, blood dyscrasias or convulsions that do not result in inpatient hospitalization, or the development of drug dependency or drug abuse.”

“Life threatening” refers to an adverse event that at occurrence represented an immediate risk of death to the subject; it does not refer to an event that hypothetically might have caused death if it were more severe. Similarly, a hospital admission for an elective procedure is not considered an SAE.

5.3. Adverse Event Reporting to the IND Sponsor

Adverse events that meet SAE Reporting Requirements must be reported and submitted by the clinical site on an expedited basis to the IND sponsor, VRC/NIAID/NIH, according to sponsor guidelines as follows:

- results in death
- is life threatening
- results in persistent or significant disability/incapacity
- requires unplanned inpatient hospitalization or prolongation of existing hospitalization
- is a congenital anomaly/birth defect in the offspring of a study subject
- is an important medical event that may jeopardize the subject or may require intervention to prevent one of the other outcomes listed above.

In addition, any event, regardless of severity, which in the judgment of an investigator represents a serious adverse event, may be reported on an expedited basis.

An investigator will communicate the initial SAE report within 24 hours of site awareness of occurrence to the IND sponsor (see Appendix 2).

A written report by investigator should be submitted to the IND Sponsor within 3 working days. In order for the IND Sponsor to comply with regulations mandating sponsor notification of specified SAEs to the FDA within 7 or 15 calendar days, the investigator must submit additional information as soon as it is available.

5.3.1. IND Sponsor Reporting to the FDA

The IND Sponsor is responsible for making the determination of which SAEs are suspected unexpected serious adverse reactions (SUSARs) that meet criteria for expedited reporting as defined in 21 CFR 312.32.

- *Suspected adverse reaction* means any adverse event for which there is a reasonable possibility that the drug caused the adverse event.
- *Unexpected Adverse Event* means an AE that is not listed in the Investigator's Brochure or is not listed at the specificity or severity that has been observed.

The IND Sponsor will also submit an IND Annual Report of the progress of the investigation to the FDA as defined in 21 CFR 312.33.

The IND Sponsor is responsible for providing copies of IND Safety Reports to the Clinical Site for submission to the IRB.

5.4. Reporting to the Institutional Review Board

5.4.1. Unanticipated Problem (UP) Definition

A serious "Unanticipated Problem (UP)" is defined as any incident, experience, or outcome that meets all three of the following criteria:

- unexpected in nature, severity, or frequency in relation to the research risks that are described in the protocol, informed consent, Investigator's Brochure, other study

documents or in consideration of the characteristics of the subject population being studied; **and**

- related to participation in the research; **and**
- suggests that the research places subjects or others at a greater risk of harm (including physical, psychological, economic, or social harm) than was previously known or recognized.

Non-serious UP: An unanticipated problem that is not an Adverse Event (UPnonAE) is an unanticipated problem that does not fit the definition of an adverse event, but which may, in the opinion of the investigator, involve risk to the subject, affect others in the research study, or significantly impact the integrity of research data. Such events would be considered a non-serious UP. For example, we will report occurrences of breaches of confidentiality, accidental destruction of study records, or unaccounted-for study drug.

5.4.2. Protocol Deviation Definition

A Protocol Deviation is defined as any change, divergence, or departure from the IRB-approved study procedures in a research protocol. Protocol deviations are designated as serious or non-serious and further characterized as:

- Those that occur because a member of the research team deviates from the protocol.
- Those that are identified before they occur, but cannot be prevented.
- Those that are discovered after they occur.

Serious Protocol Deviation: A deviation that resulted in a Serious Adverse Event or compromises the safety, welfare or rights of subjects or others.

5.4.3. Non-Compliance Definition

Non-compliance is the failure to comply with applicable NIH Human Research Protections Program (HRPP) policies, IRB requirements, or regulatory requirements for the protection of human subjects. Non-compliance is further characterized as serious, continuing or minor.

"Serious non-compliance" is defined as non-compliance that:

- Increases risks, or causes harm, to participants
- Decreases potential benefits to participants
- Compromises the integrity of the NIH-HRPP
- Invalidates the study data

"Continuing non-compliance" is non-compliance that is recurring.

"Minor non-compliance" is non-compliance that is neither serious nor continuing.

5.4.4. Expedited Reporting to the NIAID IRB

The following will be reported within 7 calendar days of investigator awareness:

- Serious and non-serious UP
- Deaths
- Serious protocol deviations
- Serious or continuing non-compliance

- SAEs that are possibly, probably, or definitely related to the research regardless of expectedness

The following waiver applies to reporting anticipated protocol deviations and expected UPnonAEs: Anticipated deviations in the conduct of the protocol will not be reported to the IRB unless they occur at a rate greater than anticipated by the study team. Expected adverse events will not be reported to the IRB unless they occur at a rate greater than that known to occur in healthy adults. If the rate of these events exceeds the rate expected by the study team, the events will be classified and reported as though they are unanticipated problems.

5.4.5. Annual Reporting to the NIAID IRB

The following will be reported to the NIAID IRB in summary at the time of Continuing Review:

- Serious and non-serious UP
- Expected SAEs that are possibly, probably, or definitely related to the research
- SAEs that are not related to the research
- All adverse events, except expected AEs granted a waiver of reporting
- Serious and Non-Serious Protocol Deviations
- Serious, continuing, and minor non-compliance
- Any trends or events which in the opinion of the investigator should be reported

5.5. Serious Adverse Event Reporting to the Institutional Biosafety Committee

The NIH Institutional Biosafety Committee (IBC) (Bethesda, MD) reviews research using recombinant DNA for compliance with NIH Guidelines. In keeping with IBC requirements, any SAE reports sent to the IRB will be subsequently provided to the IBC by the investigators.

6. STATISTICAL CONSIDERATIONS

6.1. Overview

This study is a multicenter trial to assess the safety and tolerability of MVA-EbolaZ administered alone at 2 dose levels or administered as a boost to cAd3-vectored Ebola vaccines as per study schema (Table 3). An assessment of immunogenicity will also be performed.

6.2. Objectives

The primary objective of this trial concerns safety. The secondary and exploratory objectives concern immunogenicity.

6.3. Endpoints

6.3.1. Primary Endpoints: Safety

Assessment of product safety will include clinical observation and monitoring of clinical chemistry and hematology parameters. Safety will be closely monitored after injection and

evaluated through the last study visit. See Section 4 and Appendix 3 for details and specified time points. The following parameters will be assessed for all study groups:

- Occurrence of solicited local reactogenicity signs and symptoms for 7 days following the vaccination
- Occurrence of solicited systemic reactogenicity signs and symptoms for 7 days following the vaccination
- Change from baseline for safety laboratory measures
- Occurrence of adverse events of all severities through 28 days after each injection
- Occurrence of serious adverse events or new chronic medical conditions that require ongoing medical management through the last study visit

6.3.2. Secondary Endpoints: Immunogenicity

The primary immunogenicity endpoints are ELISA and neutralization antigen-specific assays for antibody responses and intracellular cytokine staining (ICS) assay for T cell responses. The principal time point for antibody and T cell responses is 4 weeks after each vaccination.

6.3.3. Exploratory Endpoints

ELISA, neutralization assay, ICS and ELISPOT performed with research samples collected at study timepoints shown in the Schedule of Evaluations (Appendix 3), as well as other immunogenicity assays throughout the study and evaluation of genetic factors associated with immune responses, may be completed as exploratory evaluations. Vaccine-induced mRNA expression profiles through 1 week after each vaccination may also be performed as an exploratory evaluation.

6.4. Sample Size and Accrual

For Part 1, the study design is Phase 1 dose escalation based on a target accrual of 5 healthy adult participants who will receive MVA-EbolaZ at $1x10^7$ PFU (Group 1) and of 5 participants who will receive MVA-EbolaZ at $1x10^8$ PFU (Group 2). Participants in Group 3 will receive cAd3-EBO ($2x10^{11}$ PU) prime followed by MVA-EbolaZ boost at the highest dose determined to be safe for future evaluation. By the study design, the first 15 subjects enrolled in the study will be randomized between Group 1 and Group 3 in the ratio of 1:2.

After the initial safety review, in Part 2, a Phase 1b evaluation of safety and immunogenicity will continue by evaluation of the MVA-EbolaZ boost in up to 140 subjects who received cAd3-EBOZ or cAd3-EBO vaccine in VRC 207.

6.4.1. Power Calculations for Safety

The goal of the safety evaluation for this study is to identify safety concerns associated with vaccination. Primary sample size calculations for safety are expressed in terms of the ability to detect serious adverse experiences.

The ability of the study to identify SAEs will be expressed in terms of the probability of observing a certain number of serious adverse events. Useful values are the minimum true rate such that the probability of observing at least one event is at least 90%, and the maximum true rate such that the probability of not observing any event is at least 90%. Within each group of 10

participants (n=10), there is over 90% chance to observe at least 1 SAE if the true rate is at least 0.206 and over 90% chance to observe no SAE if the true rate is no more than 0.01. Over the vaccinees receiving MVA-EbolaZ vaccine, there is over 90% chance to observe at least 1 SAE if the true rate is no less than 0.002, 0.001 and 0.001 given the number of vaccinees of 50, 75 and 100, respectively. There is over 90% chance of observing no SAE if the true rate is no more than 0.046, 0.031 and 0.023 at the three respective enrollment sizes.

Probabilities of observing 0 or more than 1 serious adverse event within different size groups are presented in Table 4 for a range of possible true event rates. These calculations provide a more complete picture of the sensitivity of this study design to identify potential safety problems with the vaccine.

Table 4. Probability of Events for Different Safety and Immunogenicity Scenarios Within a Group (n=5, 10, 50, 100)

True Event Rate	n=5		n=10		n=50		n=100	
	Pr(0)	Pr(>=2)	Pr(0)	Pr(>=2)	Pr(0)	Pr(>=2)	Pr(0)	Pr(>=2)
0.005	0.975	0	0.951	0.001	0.778	0.026	0.606	0.090
0.01	0.951	0.001	0.904	0.004	0.605	0.089	0.366	0.264
0.03	0.859	0.008	0.737	0.035	0.218	0.445	0.048	0.805
0.05	0.774	0.023	0.599	0.086	0.077	0.721	0.006	0.963
0.1	0.59	0.081	0.349	0.264	0.005	0.966	0	1
0.2	0.328	0.263	0.107	0.624	0	1	0	1

Table 5 and Table 6 give the upper and lower bounds for 95% exact binomial confidence intervals of the true SAE rate at all possible numbers of events within each group. For a group with n=10 vaccinees, if none experience SAE, the 95% exact confidence interval has upper bound 0.308. Due to symmetry of estimation accuracy around 0.5, if 6 out of the 10 vaccinees experience SAE, the exact 95% confidence interval has lower bound 0.262 and upper bound 0.878.

Table 5. 95% Confidence Intervals for the True Rate at Possible Observed Number of Events Within a Group (n=5 or 10)

Observed number of events	95% confidence interval (n=5)		95% confidence interval (n=10)	
	Lower bound	Lower bound	Lower bound	Upper bound
0	0	0.522	0	0.308
1	0.005	0.716	0.003	0.445
2	0.053	0.853	0.025	0.556
3	0.147	0.947	0.067	0.652
4	0.284	0.995	0.122	0.738
5	0.478	1	0.187	0.813

Table 6. 95% Confidence Intervals for the True Rate at Possible Observed Number of Events Within a Group (n=50 or 100)

Observed number of events	95% confidence interval (n=50)		95% confidence interval (n=100)	
	Lower bound	Lower bound	Lower bound	Upper bound
0	0	0.071	0	0.036
5	0.033	0.218	0.016	0.113
10	0.1	0.337	0.049	0.176
15	0.179	0.446	0.086	0.235
25	0.355	0.645	0.169	0.347
50	0.929	1	0.398	0.602

6.4.2. Sample Size Calculations for Immunogenicity

Table 5 and Table 6 are applicable to the immunogenic response rates, and give the exact 95% confidence interval of the true response rate over possible number of responses out of the 10 subjects. For example, if we observe 5 responses within a group of 10 vaccinees, the 95% exact binomial confidence interval of the true response rate will range from 0.187 to 0.813.

Table 4 gives the probabilities of observing 0 or at least 2 responses over a range of underlying response rates. For example, if the true response rate at a particular time point is 0.20, then there is a probability of 0.893 to observe at least one response and a probability of 0.624 to observe at least two responses within a group of size n=10.

6.5. Statistical Analysis

Since enrollment is concurrent with receiving the study vaccination, the expectation is that all participants will provide some safety data.

All statistical analyses will be performed using Statistical Analysis System (SAS), R, or S-Plus statistical software.

No formal multiple comparison adjustments will be employed for safety endpoints or secondary endpoints. Missing data will be assumed to be missing at random; that is, conditional on the available observations, missingness in the observation of a variable is independent of the potential value of that variable.

6.5.1. Analysis Variables

The analysis variables consist of baseline variables, safety variables, and immunogenicity variables for primary and secondary objective analyses.

6.5.2. Baseline Demographics

Baseline characteristics including demographics and laboratory measurements will be summarized using descriptive statistics.

6.5.3. Safety Analysis

Reactogenicity

The number and percentage of participants experiencing each type of reactogenicity sign or symptom will be tabulated by severity. For a given sign or symptom, each participant's reactogenicity will be counted once under the maximum severity for all assessments.

Adverse Experiences

Adverse experiences are coded into Medical Dictionary for Regulatory Activities (MedDRA) preferred terms. The number and percentages of participants experiencing each specific adverse event will be tabulated by severity and relationship to treatment. For the calculations in these tables, each participant's adverse experience will be counted once under the maximum severity or strongest recorded causal relationship to treatment.

A complete listing of adverse experiences for each participant will provide details including severity, relationship to treatment, onset, duration and outcome.

Local Laboratory Values

Boxplots of local laboratory values will be generated for baseline values and for values measured during the course of the study. Each boxplot will show the 1st quartile, the median, and the 3rd quartile. Outliers, or values outside the boxplot, will also be plotted. If appropriate, horizontal lines representing boundaries for abnormal values will be plotted.

6.5.4. Immunogenicity Analysis

The statistical analysis for immunogenicity will employ the intent-to-treat principle, i.e., all data from enrolled participants will be used. In the final analysis of immunogenicity, if there are cases of a subject receiving a schedule different from the assignment, then an as-treated analysis will be performed.

If assay data are qualitative (i.e., positive or negative) then analyses will be performed by tabulating the frequency of positive response for each assay at each timepoint that an assessment is performed. Binomial response rates will be presented with their corresponding exact 95% confidence interval estimates. Fisher's exact tests will be used to compare two vaccine groups to each other. Missing responses will be assumed to be missing at random, i.e., conditional on the observed data the missingness is independent of the unobserved responses. Graphical descriptions of the longitudinal immune responses will also be given.

Some immunologic assays have underlying continuous or count-type readout that is often dichotomized into responder/non-responder categories. For these assays, graphical and tabular summaries of the underlying distributions will be made. These summaries may be performed on transformed data (*e.g.,* log transformation) to better satisfy assumptions of symmetry and homoscedasticity.

6.5.5. Interim analyses

Safety Reviews: The Protocol Safety Review Team (PSRT) will review safety data routinely throughout the study. The study will utilize both electronic database features and reviews by

designated safety review personnel to identify in a timely manner if any of the safety pause rules of the study are met.

Immunogenicity Review: Periodic analyses of immunogenicity for each group may be performed during the study for the purpose of informing future vaccine-related decisions in a timely manner once there are at least 3 subjects in the group with the 4 weeks post vaccination follow-up completed. The results will not influence the conduct of the trial in terms of early termination or later safety or immunogenicity endpoint assessments.

Interim Endpoint Analysis: The primary and secondary objectives may be analyzed and reported for Groups 1, 2 and Groups 4-7 after subjects reach Week 4 (visit 03). Week 4 is the principal immunogenicity time point, and data through Week 4 will be evaluated to assess the safety and immunogenicity of the MVA-EbolaZ and of the prime-boost regimen to make determinations regarding future clinical trials and accelerated advanced development.

Additional study groups, time points, and assessments may be evaluated to address additional objectives.

6.6. Randomization of Treatment Assignments

The randomization sequence will be obtained by computer-generated random numbers and provided to the study pharmacist by the protocol statistician.

The subject and the study clinicians will be informed on the subject's group assignment upon completing of enrollment in Advantage Electronic Data Collection (AdEDC) system.

7. PHARMACY AND VACCINE ADMINISTRATION PROCEDURES

The study groups are shown in Section 4. Refer to the Investigator's Brochure for further information about the study agent.

7.1. Study Agents

7.1.1. VRC-EBOMVA079-00-VP, MVA-EbolaZ vaccine

The drug product, VRC-EBOMVA079-00-VP, was prepared at the IDT Biologika GmbH, Dessau, Germany. The vaccine is a homogeneous suspension composed of MVA-EbolaZ drug substance filled into single dose vials at 3.2 x 10^8 PFU/mL. Vials are aseptically filled under cGMP to a volume of 0.7 mL. The vaccine is intended to be used for IM administration. The different dose levels are achieved by administering the appropriate volume directly from the vial or dilution to the appropriate dosage.

The Phosphate Buffered Saline (PBS, VRC-PBSPLA043-00-VP) will be used for dilution of the MVA-EbolaZ vaccine. It is supplied in 3mL glass vials containing 1.2ml of a clear colorless isotonic sterile solution.

7.1.2. VRC-EBOADC069-00-VP, cAd3-EBO vaccine

The drug product, VRC-EBOADC069-00-VP, was prepared at the Vaccine Pilot Plant (VPP). It is formulated as a 1:1 ratio of cAd3 Ebola GP Zaire and cAd3 Ebola GP Sudan drug substances. Final product meeting all test specifications will be released for use in the clinical study.

The vaccine is manufactured at a $2x10^{11}$ PU/mL dose in a formulation buffer. Vials are aseptically filled under cGMP to a volume of >1 mL to allow withdrawal of 1 mL.

7.1.3. Study Agent Labels

The labels for the VRC-EBOADC069-00-VP (cAd3-EBO) and VRC-EBOMVA079-00-VP (MVA-EbolaZ) will have specific product information (e.g., part number, lot number, fill volume, storage temperature) included on the product vial labels. The label will contain an Investigational Use Statement ("Caution: New Drug – Limited by Federal Law to Investigational Use"), and manufacturer information.

7.1.4. Study Agent Storage:

VRC-EBOADC069-00-VP (cAd3-EBO) and VRC-EBOMVA079-00-VP (MVA-EbolaZ) will be stored until use at ≤ -60°C in a qualified, continuously monitored, temperature-controlled freezer.

The diluent (PBS, VRC-PBSPLA043-00-VP) will be stored until use at -45°C to -10ºC in a qualified, continuously monitored, temperature-controlled freezer.

If deviations in storage temperature occur from the normal allowance for the pharmacy freezer, the site pharmacist must report the storage temperature excursion promptly to the PI and the IND sponsor. The excursion must be evaluated and investigated and action must be taken to restore and maintain the desired temperature limits. Pending the outcome of the investigation, the IND sponsor will notify the pharmacist if continued clinical use of the product is acceptable.

7.2. Preparation of Study Agent for Injection

This section describes how the site pharmacist will prepare study injections. Clinician instructions on how to select an arm and administer the injection are in Section 4.2.3.

To prepare the vaccine for injection, thaw the vial containing MVA-EbolaZ or cAd3-EBO at ambient temperature. Material should be kept at ambient temperature (15-25ºC) during the preparation procedure until injection. Prepared injections should be kept out of direct sunlight lying horizontal for not longer than 4 hours after removing the vial from the freezer.

Preparation should be done in a temperature controlled preparation unit on a clean table with limited access using aseptic technique. Assure that only the required vials are present in the preparation unit during reconstitution, and medication labels are strictly segregated to avoid mix-ups.

All injections will be administered IM into the deltoid muscle by needle and syringe and must be administered within 4 hours after removing the vaccine vial from the freezer.

7.2.1. Preparation of VRC-EBOMVA079-00-VP at $1x10^7$ PFU dose

Preparation of the $1x10^7$ PFU dosage of VRC-EBOMVA079-00-VP (MVA-EbolaZ) requires one serial dilution. Remove one vial of MVA-EbolaZ vaccine and two vials of diluent (PBS, VRC-PBSPLA043-00-VP) from the freezer and allow them to equilibrate to room temperature. Using 1 mL sterile syringes, draw up 1 mL of diluent from one diluent vial and 0.8 mL from the other diluent vial and inject these volumes into a 10 mL sterile vial. Then, using a 1 mL sterile syringe draw 0.2 mL of the $3.2x10^8$ PFU/mL vaccine and inject this into the 10 mL vial with the diluent to achieve a total volume of 2.0 mL. Vortex the vial at half speed for 3-5 seconds. This vial now contains $6.4x10^7$ PFU VRC-EBOMVA079-00-VP in 2.0 mL and has a concentration of $3.2x10^7$ PFU/mL. One 0.3 mL injection of this preparation will be administered for each $1x10^7$ PFU dose within 4 hours after removing the vaccine vial from the freezer.

7.2.2. Preparation of VRC-EBOMVA079-00-VP at $1x10^8$ PFU dose

No dilution is needed for preparation of a $1x10^8$ PU dose of VRC-EBOMVA079-00-VP (MVA-EbolaZ). Remove a vial of vaccine from the freezer and allow it to equilibrate to room temperature. Withdraw at least 0.3 mL from the vial to prepare a syringe for administration of a $1x10^8$ PFU dose in 0.3 mL volume. One 0.3 mL injection of the preparation will be administered IM into the deltoid muscle for each $1x10^8$ PFU dose within 4 hours after removing the vaccine vials from the freezer.

7.2.3. Preparation of VRC-EBOADC069-00-VP at $2x10^{11}$ PU dose

No dilution is needed for preparation of a $2x10^{11}$ PU dose of VRC-EBOADC069-00-VP (cAd3-EBO). Remove a vial of vaccine from the freezer and allow it to equilibrate to room temperature. One 1 mL injection of the preparation will be administered for each dose. All injections will be administered IM into the deltoid muscle by needle and syringe and must be administered within 4 hours after removing the vaccine vial from the freezer.

7.3. Study Agent Accountability

7.3.1. Documentation

The study pharmacist will be responsible for maintaining an accurate record of the codes, inventory, and an accountability record of vaccine supplies for this study. Electronic documentation as well as paper copies will be used. Final pharmacy records from non-VRC sites will be sent to the IND sponsor.

7.3.2. Disposition

The empty vials and the unused portion of a vial will be discarded in a biohazard containment bag and incinerated or autoclaved following the injection. Any unopened vials that remain at the end of the study will be discarded or transferred at the discretion of the VRC in accordance with policies that apply to investigational agents. Partially used vials or expired prepared doses cannot be administered to other subjects nor used for *in vitro* experimental studies and will be discarded as indicated above.

8. HUMAN SUBJECT PROTECTIONS AND ETHICAL OBLIGATIONS

This research study will be conducted in compliance with the protocol, Good Clinical Practices (GCP) guidelines, and all applicable regulatory requirements.

8.1. Institutional Review Board

A copy of the protocol, proposed informed consent form, other written subject information, and any proposed advertising material will be submitted to the NIAID IRB for written approval prior to implementation.

The investigator must submit and, where necessary, obtain approval from the IRB for all subsequent protocol amendments and changes to the informed consent document. The investigator will notify the IRB of deviations from the protocol and serious adverse events as per IRB policy.

The investigator will be responsible for obtaining IRB approval of the annual Continuing Review throughout the duration of the study.

8.2. Subject Recruitment and Enrollment

Subjects for this study will be recruited in accordance with the IRB standard for recruitment practices. Effort will be made to include women and minorities in proportions similar to that of the community from which they are recruited.

8.3. Informed Consent

The study informed consent template is provided in Appendix 1. The study consent describes the investigational product to be used and all aspects involved in protocol participation.

Before a subject may participate in the study, it is the investigator's responsibility to obtain written informed consent from the subject, after adequate explanation of the aims, methods, anticipated benefits, and potential hazards of the study and before any protocol-specific procedures or study medications are administered.

The acquisition of informed consent will be documented, as required by 21 CFR 312.62, and the informed consent form will be signed and personally dated by the subject and by the person who conducted the informed consent discussion. The original signed informed consent form will be retained at the site and a copy of the informed consent form will be provided to the subject.

8.4. Subject Confidentiality

The investigator must ensure that the subject's anonymity is maintained. Subjects will not be identified in any reports on this study. All records will be kept confidential to the extent provided by federal, state and local law. Medical records are made available for review when required by the FDA or other authorized users, such as the vaccine manufacturer, only under the guidelines set by the Federal Privacy Act. Direct access includes examining, analyzing, verifying, and reproducing any records and reports that are important to the evaluation of the study. The investigator is obligated to inform the subjects that the above named representatives will review their study-related records without violating the confidentiality of the subjects.

Stored study research samples are labeled by a code (such as a number) that only the VRC Clinic team can link to the subject. The requirement to maintain subject confidentiality is included in the study informed consent document.

8.5. Risks and Benefits

8.5.1. Risks of the MVA-EbolaZ Vaccine

The first and the second generation smallpox vaccines have been associated with increased incidence of developing myoperiocarditis [45], however, no cases of myopericarditis have been reported with the highly attenuated modified vaccinia Ankara (MVA) based vaccines in healthy or immunocompromised volunteers [27-35]. In the VRC 208 study, subjects will be screened for cardiovascular risks using the non-laboratory method [44]. A baseline electrocardiogram (ECG) will be performed to serve as a baseline if further evaluations are required later.

Overall, MVA-vectored vaccines targeted to different pathogens have been evaluated as safe in Phase I/II studies in healthy and immunocompromised adults and infants [27-35].

The most often observed local reactogenicity include pain, redness and swelling at injection site. Some subjects may experience pruritus at the site of injection.

Subjects may exhibit general signs and symptoms associated with administration of a vaccine, including fatigue, headache, myalgia, nausea, chills and fever.

The side effects will be monitored, but these are generally short term and do not require treatment. As with all vaccines, an allergic reaction is possible.

Study subjects can receive medications such as acetaminophen, NSAIDs, or antihistamines as required.

There may be other unknown side effects.

8.5.2. Risks of the cAd3-EBO Vaccine

Potential side effects resulting from intramuscular injection include stinging, arm discomfort, redness of the skin or mild bruising at vaccine injection sites. Study subjects can receive medications such as acetaminophen, NSAIDs, or antihistamines as required.

Subjects may exhibit general signs and symptoms associated with administration of a vaccine, including fever, chills, rash, aches and pains, nausea, headache, dizziness and fatigue. These side effects will be monitored, but are generally short term and do not require treatment. As with all vaccines, an allergic reaction is possible.

Chimpanzee adenoviral vector vaccines have been generally safe in healthy adults at dosages up to 10^{11} PU per injection in completed [40, 46] and ongoing [24] clinical trials.

An Ebola-rAd5 vaccine, previously evaluated by the VRC, was associated with asymptomatic prolongations in the activated PTT (aPTT) in the 2 weeks following vaccination in two subjects, which after further investigation were assessed as due to the induction of a non-specific anti-phospholipid antibody (APA) and not due to coagulopathy. This effect is an artifact of the aPTT test as this test measure the clotting cascade and the assay requires the presence of phospholipid as a reagent. The presence of any APA can result in an elevated aPTT *in vitro*. In these 2

subjects, the aPTT returned to normal within 4 to 8 weeks as the APA waned, consistent with the half-life of immunoglobulin. Other laboratory tests, including coagulation factors, thrombin time and prothrombin time were normal and there were no clinical abnormalities associated with the in-vitro aPTT findings [21]. These APA were further evaluated and determined to be part of the acute phase response and were not consistent with anti-phospholipids that are known to be associated with disease. The basis for this *in vitro* phenomenon has been reported by others and observed following administration of other adenovirus vectored products [47-51]. Observation of prolonged PTT consistent with this above noted etiology has also been observed with the cAd3-EBO vaccine [24]. This is an expected adverse event. The protocol includes a plan for assessing the basis for abnormal aPTT findings.

There may be other unknown side effects.

8.5.3. Other Risks

Blood drawing may cause pain, bruising, fainting, and, rarely, infection at the site where the blood is taken.

Women of reproductive potential will be required to agree to use birth control for sexual intercourse beginning 21 days prior to enrollment and continue through 24 weeks after the study injection. Because this is a research study, women of reproductive potential will be asked to notify the site immediately upon learning of a pregnancy during this study and will be tested for pregnancy prior to administration of the study injection. The amount of blood drawn will be reduced. The subject will be contacted to ask about the outcome of a pregnancy that begins during the study.

It is possible that the standard medical tests performed as part of this research protocol will result in new diagnoses. Depending upon the medical findings and consequences of being provided with the new medical information about health status, the study subject may view this aspect of study participation as either a risk or a benefit. Any such information will be shared and discussed with the subject and, if requested by the subject, will be forwarded to the subject’s primary health care provider for further workup and management.

8.5.4. Study Benefits

Study subjects will receive no direct benefit from participation as this is the first study in humans. Others may benefit from knowledge gained in this study that may aid in the development of an Ebolavirus vaccine.

8.6. Plan for Use and Storage of Biological Samples

The plan for use and storage of biological samples from this protocol is as outlined in the following sections.

8.6.1. Use of Samples, Specimens and Data

Samples, specimens and data collected under this protocol may be used to conduct protocol-related safety and immune response evaluations, exploratory laboratory evaluations related to the type of infection the study agent was designed to prevent, exploratory laboratory evaluations related to vaccine or infectious disease research in general and for research assay validation.

Genetic testing may be performed in accordance with the genetic testing information that was included in the study informed consent.

8.6.2. Storage and Tracking of Blood Samples and Other Specimens

All of the stored study research samples are labeled by a code (such as a number) that only the site of enrollment can link to the subject. Samples are stored at the NIAID Vaccine Immune T-Cell and Antibody Laboratory (NVITAL), Gaithersburg, MD or at VRC Laboratories in Building 40 or at a collaborating research laboratory; all are secure facilities with limited access. Data will be kept in password-protected computers. Only investigators or their designees will have access to the samples and data. Samples will be tracked in the Laboratory Information Management System (LIMS) database or using another software designed for this purpose (e.g., Freezerworks).

8.6.3. Disposition of Samples, Specimens and Data at Completion of the Protocol

In the future, other investigators (both at NIH and outside) may wish to study these samples and/or data. IRB approval must be sought prior to any sharing of samples. Any clinical information shared about those samples would similarly require prior IRB approval. The research use of stored, unlinked or unidentified samples may be exempt from the need for prospective IRB review and approval. Exemption requests will be submitted in writing to the NIH Office of Human Subjects Research, which is authorized to determine whether a research activity is exempt.

At the time of protocol termination, samples will remain at NVITAL, VRC and other site laboratories or, after IRB approval, transferred to another repository. Regulatory oversight of the stored samples and data may be transferred to a stored samples protocol as part of the IRB-approved termination plan. Data will be archived by the VRC in compliance with requirements for retention of research records, or, after the IRB and the IND sponsor approval, may be either destroyed or transferred to another repository.

8.6.4. Loss or Destruction of Samples, Specimens or Data

The NIH Intramural Protocol Deviation definition related to loss of or destruction of samples or data will be followed. Any loss or unanticipated destruction of samples (for example, due to freezer malfunction) or data (for example, misplacing a printout of data with identifiers) that compromises the scientific integrity of the study will be reported to the IRB in accordance with institutional policies. The PI will also notify the IRB if the decision is made to destroy the remaining samples.

8.7. Subject Identification and Enrollment of Study Participants

All study activities will be carried out at approved study sites, beginning first at the NIH Clinical Center. The vaccine-naive study subjects (Groups 1, 2 and 3) will be recruited through on-site and off-site advertising done for the screening protocol, VRC 500 (11-I-0164). The rollover subjects from VRC 207 will be provided with an IRB approved information letter about the study. This phase 1/1b study is designed to establish safety of the vaccines in healthy adults. Effort will be made to include women and minorities in proportions similar to that of the community from which they are recruited.

A subject who enrolled into VRC 207 (14-I-0183) at one site may participate in the rollover booster vaccination with the MVA-EbolaZ vaccine at another site. Similarly, a subject who enrolls at one site may transfer to another site for completion of the study.

The subject will be consented at each site where study visits have been conducted so that each site will have a consent in the site files for the subject.

In reports accounting for VRC 208 (15-I-0107) enrollments, the subject have a study identification code based on the site of enrollment for VRC 208 and will be counted in the VRC 208 accrual under the site of enrollment for VRC 208.

8.7.1. Participation of Children

Children are not eligible to participate in this clinical trial because the investigational vaccine has not been previously evaluated in adults. If the product is assessed as safe and immunogenic, other protocols designed for children may be conducted in the future.

8.7.2. Participation of NIH Employees and Employees of Study Sites

Each study site will follow any institutional policies related to participation of employees. At the NIH Clinical Center/VRC site, employees and members of their immediate families may participate in this protocol. The NIH Clinical Center/VRC site will follow the Guidelines for the Inclusion of Employees in NIH Research Studies and will give each employee a copy of the "NIH Information Sheet on Employee Research Participation."

Neither participation nor refusal to participate will have an effect, either beneficial or adverse, on the participant's employment or work situation. The NIH information sheet regarding NIH employee research participation will be distributed to all potential subjects who are NIH employees. The employee subject's privacy and confidentiality will be preserved in accordance with NIH Clinical Center and NIAID policies. For NIH employee subjects, consent will be obtained by an individual who is independent of the employee's team. If the individual obtaining consent is a co-worker to the subject, independent monitoring of the consent process will be included through the Bioethics Consultation Service. Protocol study staff will be trained on obtaining potentially sensitive and private information from co-workers or subordinates.

8.8. Compensation

Subjects will be compensated for time and inconvenience in accordance with the standards for compensation at each site. The total compensation for the subject is based on the number of study clinic visits, injections completed and if optional research blood collections are performed. The approximate total compensation is included in each site informed consent document.

8.9. Safety Monitoring, Protocol Safety Review Team

Close cooperation between the designated members of the Protocol Team will occur to evaluate and respond to individual adverse events in a timely manner. Site clinicians will conduct a daily safety review of any new clinical data. The Protocol Safety Review Team (PSRT) includes designated team members (Principal Investigators, Associate Investigators, Study Coordinators, Protocol Specialist, and other Study Clinicians). The PSRT will routinely review the summary

study safety data reports on a weekly basis through 4 weeks after the last subject receives the final study injection in order to be certain that the vaccine has an acceptable safety profile and will continue to monitor the study safety data reports on at least a monthly basis through completion of the study. The PSRT will also perform the dose continuation and dose escalation reviews specified in Section 4.3.

9. ADMINISTRATIVE AND LEGAL OBLIGATIONS

9.1. Protocol Amendments and Study Termination

Protocol amendments must be made only with the prior approval of the IND sponsor, VRC/ NIAID. Agreement from the investigator must be obtained for all protocol amendments and amendments to the informed consent document. All study amendments will be submitted to the IRB for approval.

The VRC Study Chair, the NIAID IRB, NIAID and the FDA reserve the right to terminate the study. The Study Chair will notify the IRB in writing of the study's completion or early termination.

9.2. Study Documentation and Storage

Each site Principal Investigator will maintain a list of appropriately qualified persons to whom trial duties have been delegated.

Source documents are original documents, data, and records from which the subject's data are obtained. These include but are not limited to hospital records, clinical and office charts, laboratory and pharmacy records, diaries, microfiches, radiographs, and correspondence.

The investigator and staff are responsible for maintaining a comprehensive and centralized filing system of all study-related (essential) documentation, suitable for inspection at any time by representatives from the VRC, IRB, FDA, and/or applicable regulatory authorities. Elements include:

- Subject files containing completed informed consent forms, and supporting copies of source documentation
- Study files containing the protocol with all amendments, Investigator Brochures, and copies of all correspondence with the IRB and VRC

In addition, all original source documentation must be maintained and be readily available.

All essential documentation should be retained by the institution for the same period of time required for medical records retention. The FDA requires study records to be retained for two years after marketing approval or refusal (21 CFR 312.62). No study document should be destroyed without prior written agreement between the VRC and the Site Principal Investigator. Should the investigator wish to assign the study records to another party or move them to another location, VRC must be notified in writing of the new responsible person and/or the new location.

9.3. Data Collection and Protocol Monitoring

9.3.1. Data Collection

Clinical research data will be collected in a secure electronic web-based clinical data management system (CDMS) through a contract research organization, EMMES (Rockville, MD). Extracted data without patient identifiers will be sent to the Protocol Statistician for statistical analysis.

9.3.2. Source Documents

The site will maintain appropriate medical and research records for this trial, in compliance with ICH-GCP, regulatory and institutional requirements for the protection of confidentiality of subjects.

Source data are all information, original records of clinical findings, observations, or other activities in a clinical trial necessary for the reconstruction and evaluation of the trial. Examples of these original documents and data records include, but are not limited to, medical records, laboratory reports, pharmacy records and other research records maintained for the clinical trial.

9.3.3. Protocol Monitoring Plan

The NIAID and VRC or their authorized representatives are responsible for contacting and visiting the site investigators for the purpose of inspecting the facilities and, upon request, inspecting the various records of the trial, provided that subject confidentiality is respected.

Site investigators will allow the study monitors, the NIAID IRB, and the FDA to inspect study documents (*e.g.*, consent forms, drug distribution forms, and case report forms) and pertinent hospital or clinic records for confirmation of the study data.

Site visits by study monitors will be made in accordance with the study monitoring plan to monitor the following: study operations, the quality of data collected in the research records, the accuracy and timeliness of data entered in the database, and to determine that all process and regulatory requirements are met. Study monitoring visits will occur as defined by the IND Sponsor approved monitoring plan.

9.4. Language

All written information and other material to be used by subjects and investigative staff must use vocabulary and language that are clearly understood.

9.5. Policy Regarding Research-Related Injuries

The study site will provide short-term medical care for any injury resulting from participation in this research. In general, the Study Sites, the NIH, the Clinical Center, or the Federal Government will provide no long-term medical care or financial compensation for research-related injuries.

9.6. Multi-site Management

The Vaccine Research Center, NIAID, NIH is the coordinating center as well as a site for this protocol. Each site that will be participating in enrolling and vaccinating study subjects will have a site Principal Investigator and Associate Investigators (see Appendix 2) who have parallel roles at their respective institutions in managing the conduct of the study at their sites in compliance with all applicable regulations and good clinical practices. The protocol plan is to establish a Reliance Agreement with each collaborating study site such that the NIAID IRB is the IRB of Record for the conduct of the VRC 208 protocol. If a reliance agreement is not established, the site will be required to obtain a local IRB review.

10. REFERENCES

1. Kuhn, J.H., et al., *Virus nomenclature below the species level: a standardized nomenclature for natural variants of viruses assigned to the family Filoviridae.* Arch Virol, 2013. **158**(1): p. 301-11.
2. Sanchez, A., et al., *Sequence analysis of the Ebola virus genome: organization, genetic elements, and comparison with the genome of Marburg virus.* Virus Res, 1993. **29**(3): p. 215-40.
3. Sanchez, A., et al., *Sequence analysis of the Marburg virus nucleoprotein gene: comparison to Ebola virus and other non-segmented negative-strand RNA viruses.* J Gen Virol, 1992. **73 (Pt 2)**: p. 347-57.
4. Hart, M.K., *Vaccine research efforts for filoviruses.* Int J Parasitol, 2003. **33**(5-6): p. 583-95.
5. WHO. *Ebola virus disease; Fact Sheet*. 2014 September 2014 [cited 2014; Available from: http://www.who.int/mediacentre/factsheets/fs103/en/.
6. Geisbert, T.W. and P.B. Jahrling, *Exotic emerging viral diseases: progress and challenges.* Nat Med, 2004. **10**(12 Suppl): p. S110-21.
7. Meslin, F.X., *Global aspects of emerging and potential zoonoses: a WHO perspective.* Emerg Infect Dis, 1997. **3**(2): p. 223-8.
8. Okware, S.I., et al., *An outbreak of Ebola in Uganda.* Trop Med Int Health, 2002. **7**(12): p. 1068-75.
9. Hensley, L.E., et al., *Ebola and Marburg viruses: pathogenesis and development of countermeasures.* Curr Mol Med, 2005. **5**(8): p. 761-72.
10. Bausch, D.G., et al., *Assessment of the risk of Ebola virus transmission from bodily fluids and fomites.* J Infect Dis, 2007. **196 Suppl 2**: p. S142-7.
11. Kreuels, B., et al., *A Case of Severe Ebola Virus Infection Complicated by Gram-Negative Septicemia.* New England Journal of Medicine, 2014. **371**(25): p. 2394-2401.
12. Sullivan, N., Z.Y. Yang, and G.J. Nabel, *Ebola virus pathogenesis: implications for vaccines and therapies.* J Virol, 2003. **77**(18): p. 9733-7.
13. Dixon, M.G. and I.J. Schafer, *Ebola Virus Disease Outbreak - West Africa, 2014.* Morbidity and mortality weekly report, 2014. **63**: p. 1-4.
14. WHO. *Ebola Response Roadmap: Situation Report*. 2015 14 January 2015; Available from: http://apps.who.int/iris/bitstream/10665/148237/2/roadmapsitrep_14Jan2015_eng.pdf?ua=1.
15. Verheust, C., et al., *Biosafety aspects of modified vaccinia virus Ankara (MVA)-based vectors used for gene therapy or vaccination.* Vaccine, 2012. **30**(16): p. 2623-32.
16. Gomez, C.E., et al., *Clinical applications of attenuated MVA poxvirus strain.* Expert Rev Vaccines, 2013. **12**(12): p. 1395-416.
17. Gilbert, S.C., *Clinical development of Modified Vaccinia virus Ankara vaccines.* Vaccine, 2013. **31**(39): p. 4241-6.
18. Sutter, G. and B. Moss, *Nonreplicating vaccinia vector efficiently expresses recombinant genes.* Proc Natl Acad Sci U S A, 1992. **89**(22): p. 10847-51.
19. Stanley, D.A., et al., *Chimpanzee adenovirus vaccine generates acute and durable protective immunity against ebolavirus challenge.* Nat Med, 2014. **20**(10): p. 1126-9.

20. Martin, J.E., et al., *A DNA vaccine for Ebola virus is safe and immunogenic in a phase I clinical trial.* Clin Vaccine Immunol, 2006. **13**(11): p. 1267-77.
21. Ledgerwood, J.E., et al., *A replication defective recombinant Ad5 vaccine expressing Ebola virus GP is safe and immunogenic in healthy adults.* Vaccine, 2010. **29**(2): p. 304-13.
22. Sarwar, U.N., et al., *Safety and Immunogenicity of DNA Vaccines Encoding Ebolavirus and Marburgvirus Wild-Type Glycoproteins in a Phase I Clinical Trial.* J Infect Dis, 2014.
23. Kibuuka, H., et al., *Safety and immunogenicity of Ebola virus and Marburg virus glycoprotein DNA vaccines assessed separately and concomitantly in healthy Ugandan adults: a phase 1b, randomised, double-blind, placebo-controlled clinical trial.* Lancet, 2014.
24. Ledgerwood, J.E., et al., *Chimpanzee Adenovirus Vector Ebola Vaccine - Preliminary Report.* N Engl J Med, http://www.nejm.org/doi/full/10.1056/NEJMoa1410863, 2014.
25. Yang, Z.Y., et al., *Identification of the Ebola virus glycoprotein as the main viral determinant of vascular cell cytotoxicity and injury.* Nat Med, 2000. **6**(8): p. 886-9.
26. Sullivan, N.J., et al., *Immune protection of nonhuman primates against Ebola virus with single low-dose adenovirus vectors encoding modified GPs.* PLoS Med, 2006. **3**(6): p. e177.
27. Vollmar, J., et al., *Safety and immunogenicity of IMVAMUNE, a promising candidate as a third generation smallpox vaccine.* Vaccine, 2006. **24**(12): p. 2065-70.
28. von Krempelhuber, A., et al., *A randomized, double-blind, dose-finding Phase II study to evaluate immunogenicity and safety of the third generation smallpox vaccine candidate IMVAMUNE.* Vaccine, 2010. **28**(5): p. 1209-16.
29. Frey, S.E., et al., *Phase II randomized, double-blinded comparison of a single high dose (5x10(8) TCID50) of modified vaccinia Ankara compared to a standard dose (1x10(8) TCID50) in healthy vaccinia-naive individuals.* Vaccine, 2014. **32**(23): p. 2732-9.
30. Frey, S.E., et al., *Safety and immunogenicity of IMVAMUNE(R) smallpox vaccine using different strategies for a post event scenario.* Vaccine, 2013. **31**(29): p. 3025-33.
31. Peters, B.S., et al., *Studies of a prophylactic HIV-1 vaccine candidate based on modified vaccinia virus Ankara (MVA) with and without DNA priming: effects of dosage and route on safety and immunogenicity.* Vaccine, 2007. **25**(11): p. 2120-7.
32. Gorse, G.J., et al., *DNA and modified vaccinia virus Ankara vaccines encoding multiple cytotoxic and helper T-lymphocyte epitopes of human immunodeficiency virus type 1 (HIV-1) are safe but weakly immunogenic in HIV-1-uninfected, vaccinia virus-naive adults.* Clin Vaccine Immunol, 2012. **19**(5): p. 649-58.
33. Antrobus, R.D., et al., *A T cell-inducing influenza vaccine for the elderly: safety and immunogenicity of MVA-NP+M1 in adults aged over 50 years.* PLoS One, 2012. **7**(10): p. e48322.
34. Lillie, P.J., et al., *Preliminary assessment of the efficacy of a T-cell-based influenza vaccine, MVA-NP+M1, in humans.* Clin Infect Dis, 2012. **55**(1): p. 19-25.
35. Tameris, M.D., et al., *Safety and efficacy of MVA85A, a new tuberculosis vaccine, in infants previously vaccinated with BCG: a randomised, placebo-controlled phase 2b trial.* Lancet, 2013. **381**(9871): p. 1021-8.
36. EMA. *Imvanex, Marketing Authorization from the European Medicines Agency (EMA).* 2013 [cited 2014 15 December]; Available from:

http://www.ema.europa.eu/ema/index.jsp?curl=pages/medicines/human/medicines/002596/human_med_001666.jsp&mid=WC0b01ac058001d124.

37. Sheehy, S.H., et al., *Phase Ia clinical evaluation of the Plasmodium falciparum blood-stage antigen MSP1 in ChAd63 and MVA vaccine vectors.* Mol Ther, 2011. **19**(12): p. 2269-76.
38. Ewer, K.J., et al., *Protective CD8+ T-cell immunity to human malaria induced by chimpanzee adenovirus-MVA immunisation.* Nat Commun, 2013. **4**: p. 2836.
39. Ogwang, C., et al., *Safety and immunogenicity of heterologous prime-boost immunisation with Plasmodium falciparum malaria candidate vaccines, ChAd63 ME-TRAP and MVA ME-TRAP, in healthy Gambian and Kenyan adults.* PLoS One, 2013. **8**(3): p. e57726.
40. O'Hara, G.A., et al., *Clinical assessment of a recombinant simian adenovirus ChAd63: a potent new vaccine vector.* J Infect Dis, 2012. **205**(5): p. 772-81.
41. Betts, M.R., J.P. Casazza, and R.A. Koup, *Monitoring HIV-specific CD8+ T cell responses by intracellular cytokine production.* Immunol Lett, 2001. **79**(1-2): p. 117-25.
42. Helms, T., et al., *Direct visualization of cytokine-producing recall antigen-specific CD4 memory T cells in healthy individuals and HIV patients.* J Immunol, 2000. **164**(7): p. 3723-32.
43. Quinn, K.M., et al., *Comparative analysis of the magnitude, quality, phenotype, and protective capacity of simian immunodeficiency virus gag-specific CD8+ T cells following human-, simian-, and chimpanzee-derived recombinant adenoviral vector immunization.* J Immunol, 2013. **190**(6): p. 2720-35.
44. Gaziano, T.A., et al., *Laboratory-based versus non-laboratory-based method for assessment of cardiovascular disease risk: the NHANES I Follow-up Study cohort.* Lancet, 2008. **371**(9616): p. 923-31.
45. *ACAM2000 Vaccines and Related Biological Products Advisory Committee (VRBPAC) Briefing Document* 2007 [cited 2014, December 8]; Available from: http://www.fda.gov/ohrms/dockets/ac/07/briefing/2007-4292b2-02.pdf.
46. Barnes, E., et al., *Novel adenovirus-based vaccines induce broad and sustained T cell responses to HCV in man.* Sci Transl Med, 2012. **4**(115): p. 115ra1.
47. Shoenfeld, Y., M. Blank, and I. Krause, *The relationship of antiphospholipid antibodies to infections--do they bind to infecting agents or may they even be induced by them?* Clin Exp Rheumatol, 2000. **18**(4): p. 431-2.
48. Jaeger, U., et al., *Transient lupus anticoagulant associated with hypoprothrombinemia and factor XII deficiency following adenovirus infection.* Ann Hematol, 1993. **67**(2): p. 95-9.
49. Malaeb, B.S., et al., *Elevated activated partial thromboplastin time during administration of first-generation adenoviral vectors for gene therapy for prostate cancer: identification of lupus anticoagulants.* Urology, 2005. **66**(4): p. 830-4.
50. Cervera, R., et al., *Antiphospholipid syndrome associated with infections: clinical and microbiological characteristics of 100 patients.* Ann Rheum Dis, 2004. **63**(10): p. 1312-7.
51. Enama, M.E., et al. *Induction of false-positive PTT elevations by investigational adenoviral vector vaccines*. in *14th Annual Conference on Vaccine Research*. 2011. Baltimore, MD.

APPENDIX 1. INFORMED CONSENT FORM

Informed Consent Template

TITLE: VRC 208: A Phase 1/1b Open-Label Clinical Trial to Evaluate Dose, Safety and Immunogenicity of a Recombinant Modified Vaccinia Virus Ankara Ebola Vaccine, VRC-EBOMVA079-00-VP (MVA-EbolaZ), Administered Alone or as a Boost to cAd3-Ebola Vaccines in Healthy Adults

INTRODUCTION

We invite you to take part in a research study at [site] the National Institutes of Health (NIH).

First, we want you to know that:

> Taking part in [site] NIH research is entirely voluntary.
>
> You may choose not to take part, or you may withdraw from the study at any time. In either case, you will not lose any benefits to which you are otherwise entitled. However, to receive care at the NIH, you must be taking part in a study or be under evaluation for study participation [change to site appropriate statement].
>
> You may receive no benefit from taking part. The research may give us knowledge that may help people in the future.

Second, some people have personal, religious or ethical beliefs that may limit the kinds of medical or research treatments they would want to receive (such as blood transfusions). If you have such beliefs, please discuss them with your [site] NIH doctors or research team before you agree to the study.

Now we will describe this research study. Before you decide to take part, please take as much time as you need to ask any questions and discuss this study with anyone at [site] NIH, or with family, friends or your personal physician or other health professional.

PURPOSE OF THE STUDY

This is a study of two experimental vaccines for the prevention of Ebola virus infection. “Experimental” means that the study vaccines have not been approved by the Food and Drug Administration (FDA). The FDA allows the use of an experimental vaccine in research only. It is not known if the vaccines work. The plan of this study is to enroll up to 160 people. There are 3 separate study sites. The main purpose of this study is to see if the experimental vaccines are safe and if they cause any side effects. We also want to study immune responses to the vaccines.

Ebola virus was discovered in 1976. It is named for a river in Africa that is near where the virus was discovered. Bats in some parts of Africa may carry the virus. The virus causes an infection known as Ebola virus disease (EVD). EVD is a rare disease. It starts with fever and muscle aches. More severe symptoms are diarrhea and vomiting, breathing problems, severe bleeding, kidney problems, and shock. The infection can also lead to death. EVD caused 340 deaths in Africa in 1976. When the Ebola virus has been spread in Africa, 50% to 90% of infected people have died. Ebola virus does not occur naturally in the United States. The 2014 EVD outbreak in

West Africa is the largest to date. There have been more people infected than in all other outbreaks combined. Also, EVD has occurred in some countries in Africa for the very first time.

You should not expect this experimental vaccine to protect you from Ebola virus infection. You must take steps to protect yourself f if you think you might be exposed through travel or work. If you have any symptoms that seem like EVD, tell the study team and get medical care as soon as possible. The study vaccines do not treat EVD. You should not enroll in this study if you have recently been exposed to Ebola virus.

You can be in this study because you understand the information given to you about the study and you are in general good health.

The Vaccine Research Center (VRC), NIH, sponsors the study. Your study visits will be at [insert Site name and city].

The study will last about 11 months if you get one study injection and 14 months if you get 2 study injections. While on the study, we will watch for possible effects from the vaccine.

We will tell you if we learn anything new during this study that might cause you to change your mind about staying in the study. At the end of the study, we will tell you when study results may be available and how to learn about them.

STUDY VACCINES

The technical names of the vaccines are: VRC-EBOMVA079-00-VP and VRC-EBOADC069-00VP. We will call them the “MVA-EbolaZ” and “cAd3-EBO” vaccines, or just the “Ebola vaccine.”

This is the first study to give the MVA-EbolaZ experimental vaccine to humans. The experimental cAd3-EBO vaccine is being studied now in clinical trials. Vaccines are given to teach the body to prevent or fight an infection. The study vaccines do not have live or killed Ebola virus in them. It is **impossible** for the study vaccines to give you an Ebola infection.

The NIH developed the experimental Ebola vaccines in this study.

The MVA-EbolaZ vaccine is made by using a virus called “modified vaccinia virus Ankara” (MVA), to deliver manufactured DNA to cells in your body. MVA is a weakened form of the virus used to make vaccines against smallpox. The cells in your body will use the vaccine to make small amounts of a protein from the Ebola Zaire type. Your body’s immune system may then respond to this protein. The MVA vaccine cannot multiply in a human body. You cannot get Ebola or MVA and you cannot give it to someone else when you get the study vaccine(s).

The cAd3-EBO vaccine is made by using a shell of a cold virus to deliver manufactured DNA to cells in your body. The cells in your body will use the vaccine to make small amounts of a protein from Ebola. Your body’s immune system may then respond to this protein. The cold virus used to make the vaccine is from a chimpanzee cold virus. This cold virus does not make humans sick. You cannot get Ebola or the cold virus and you cannot give them to someone else when you get the study vaccine. The cAd3-EBO vaccine has two parts. One part is directed against the Zaire type of the Ebola virus. The other part is directed against the Sudan type of the Ebola virus.

Part 1 of the VRC 208 study has 3 groups. People in Group 1 and Group 2 will get 1 injection of the MVA-EbolaZ vaccine. Group 1 will get a lower dose and Group 2 will get a higher dose of the vaccine. People in Group 3 will receive 2 injections, first cAd3-EBO (prime) and then MVA-EbolaZ (boost), 8 weeks later.

In Part 2 of the VRC 208 study, people who were in the VRC 207 study and got their first injection will get 1 booster injection of MVA-EbolaZ in the VRC 208 study.

Here is the study plan:

VRC 208				
**Part 1:**				
**Group**	**Subjects**	**Day 0**	**Week 8 Boost**	**Study site**
1	5	MVA-EbolaZ (1x10^7 PFU*)		VRC
2	5	MVA-EbolaZ (1x10^8 PFU*)		VRC
3	10	cAd3-EBO (2x10^{11} PU*)	MVA-EbolaZ (1x10^8 PFU*)	VRC
**Part 2:**				
**Groups**	**Subjects**	**Vaccine Given in VRC 207**	**VRC 208**	
4-7	up to 140	cAd3-EBOZ or cAd3-EBO	MVA-EbolaZ (1x10^8 PFU*)	Emory, UMD, VRC
**Total**	up to 160	*PFU and PU are measures of vaccine dose.		

Here at the [site name] we will be giving the [insert vaccine name] to participants.

STUDY PROCEDURES

If you agree to take part in the study you will get 1 or 2 study vaccine injections depending on the study group. You will know how many injections you will get.

We will use a needle and syringe to give you the vaccine into a muscle in your upper arm. This is called an intramuscular "IM" injection. There is no placebo in this study.

The study will be conducted in 2 parts. Part 1 will begin at the NIH Clinical Center (Bethesda, MD). The first 15 people to enroll into the study will be enrolled randomly (like flipping a coin) into either Group 1 or Group 3. You will not be able to choose the group, and you will find out which vaccine you are getting before the injection is given. The next 5 people will be enrolled in Group 2.

The study will continue with Part 2 and include up to 140 more participants. Participants who enroll in Part 2 of the study will be healthy adults who already received an investigational Ebola vaccine in the VRC 207 study.

Here at the [site name] we will be enrolling about [number] of participants. Participants here will: [insert either: "be enrolled in Groups 1, 2 and 3 of the study. You will find out what vaccine you are getting on the day you enroll before the injection is given." Or: "receive one dose of the MVA-EbolaZ vaccine"].

The vaccination visit will take about 3 to 5 hours. You will need to stay at the clinic for at least 1 hour after the vaccine is injected into your arm, and for at least 3 hours if you are in Group 1 or Group 2. Other visits are expected to take 2 hours or less.

Experimental vaccine studies follow a set schedule. This helps us answer the research questions. In the first 2 months after you enroll into the study, you will have at least 6 clinic visits and 1 telephone contact. Scheduling for your visits allows some flexibility, but it is important that you work with the staff to follow the schedule. You should try to not miss any visits.

One to 2 days after the study injection, you must call the clinic staff to report how you are feeling. If you do not call, we will contact you. For 7 days after the study injection you will be asked to check your temperature with a thermometer we give you and write it down. You will also be asked to look at the place on your arm where you got an injection and measure any redness or swelling with a ruler we give you. You will be asked to write down any symptoms you may have. If you have any side effects, you should tell a study physician or a nurse as soon as possible. You can reach the clinic staff by phone 24 hours per day. If you have symptoms, you might have to come into the clinic for an examination before your next scheduled visit. It is very important that you follow the instructions you get from the clinic staff.

At each visit, we will check you for any health changes or problems. We will ask you how you are feeling and if you have taken any medications. We will draw your blood at scheduled study visits and we may need urine samples. We will tell you right away if any of your test results show a health problem. We will use some blood samples to study your immune response to the vaccine. Results of immune response tests are not for checking on your health and we will not give you these results during the study.

We draw about 9 to 17 tubes of blood from you, depending on the visit. You might need to have extra clinic visits and laboratory tests if you have health changes that need to be checked.

MONITORING OF THE STUDY

A group of physicians and scientists at the NIH Clinical Center will monitor this study. This group will review the information from the study and will pay close attention to possible harmful reactions.

GENETIC TESTING

Some of the blood drawn from you as part of this study will be used for genetic tests. Some genetic tests are done in research studies to see if genetic differences in people cause different types of immune responses. Your blood sample used in these genetic tests will not have your name on it and the results will not be in your medical record. These tests are not used to check your health and we will not tell you the results.

A special genetic test, called HLA typing, may be ordered through the NIH Clinical Center medical laboratory. If this test is done at the NIH Clinical Center, your HLA type results will be in your medical record. These results are not used to check on your health and are not usually reviewed by a health care provider. Any genetic testing, including HLA testing is for research purposes only. Any genetic information collected or learned about you will be kept confidential.

Medical records, including HLA test results are kept securely. We will not give any genetic information that is in your medical record to anyone without your permission.

STORED SAMPLES

We will draw blood samples from you during your participation in this study. We will keep these samples for future research to learn more about viruses, vaccines, the immune system, and/or other medical conditions. Results from the research done with your stored samples will not be used for medical care. We will not give these results to you or to your health care provider and will not put them in your medical record.

Labeling of Stored Samples

We will label your stored samples by a code (like a number). Only the study team can link these numbers to you. Any identifying information about you will be kept confidential as much as the law allows.

Risks from Stored Samples

The greatest risk is the unplanned release of information from your medical records. The chance that this information will be given to an unauthorized person without your permission is very small. Possible problems with the unplanned release of information include discrimination when applying for insurance and employment. Similar problems may occur if you give information yourself or agree to have your medical records released.

Future Studies

In the future, other researchers at NIH or outside of NIH may wish to study your stored samples. When the study team shares your stored samples, they will be marked with a code. Your samples will not have any identifying information on them. Some information about you, like your gender, age, health history, or ethnicity may also be shared with other researchers. Any future research studies that use your samples will be reviewed by the Institutional Review Board (IRB). This is a special committee that watches medical research studies to protect the rights and welfare of human subjects.

Your stored samples will be used for research only and will not be sold. The research done with your samples may be used to make new products in the future. You will not receive payment for such products.

Making your Choice: You cannot take part in this study if you do not want us to collect or store your blood samples. If you agree to take part in this study, you must also agree to let us keep any of your samples for future research. If you decided to not take part in this study, you may still be eligible for other studies at NIH.

POSSIBLE STUDY RISKS

Possible **risks from the injection:** temporary stinging, pain, redness, soreness, itchiness, swelling or bruising at the injection site on your arm. There is a very small chance of infection.

Possible **risks of blood drawing:** pain, bleeding, bruising, feeling lightheaded, and fainting. Rarely, infection may occur at the site where the blood is taken.

Possible **risks from genetic testing:** unplanned release of information that could be used by insurers or employers; discovering a gene or HLA type that suggests risk of disease for you or your family; discovering undisclosed family relationships.

Possible **risks from any vaccine:** fever, chills, rash, aches and pains, nausea, headache, dizziness, and feeling tired and/or unwell. These types of reactions are usually greatest within the first 24 hours after vaccination and may last 1 to 3 days. Over-the-counter medicine, like acetaminophen (Tylenol), may be used to help relieve vaccination reactions.

Possible **risk of allergic reaction:** Rarely, a serious allergic reaction may occur shortly after any vaccination. This is called "anaphylaxis" and may be life-threatening. During the time you have to stay in the clinic after the vaccination, we will check your health. Treatment to control a reaction will be quickly given if an allergic reaction occurs.

Possible **risks of the MVA-EbolaZ experimental vaccine:** Before this study started, MVA-EbolaZ experimental vaccine was not given to humans. The risks are unknown. As previously described, this vaccine is based on a smallpox vaccine called MVA. Other types of smallpox vaccines (not MVA) were linked to a slightly greater risk of developing temporary inflammation in and around the heart. MVA has not caused this condition; however, we will do an electrocardiogram (ECG) at baseline in this study as a precaution. We will also check your health while you are on the study.

In studies with other MVA-based vaccines, the most common complaints after vaccination have been arm soreness, redness and swelling at the injection site, feeling tired and/or unwell, headache, muscle aches, nausea and chills.

As with any vaccine, there may be a risk of skin rash, hives, or other unknown side effects.

Possible **risks of the cAd3-EBO experimental vaccine:** In the VRC 207 study, cAd3-EBO experimental vaccine was found to be safe for additional studies in humans. As of January 2015, more than 100 people received an injection in VRC 207 study. Other studies with the vaccine are also ongoing. So far, the most common complaints after vaccination have been arm soreness, headache, muscle aches and feeling tired or unwell. Some people had fever within a day after vaccination. A few people had a temporary change in a laboratory test that required more tests. As with any vaccine, there may be a risk of skin rash, hives, or other unknown side effects.

Getting the experimental Ebola vaccines in this study may or may not affect how you respond to future Ebola vaccines. At this time, no vaccines are approved for use to protect against Ebola virus infection. We do not know if you will develop an immune response, like antibodies, after getting the vaccine(s). If you are exposed to a person with Ebola infection in the future, we do not know if these vaccines will make you less likely, more likely or have no effect on whether you would get Ebola infection. If you do develop an immune response, we also do not know how long it may last.

Unknown **safety risks:** There may be side effects from the study vaccine that we do not yet know about. Please tell the study staff about any side effect you think you are having. This is important for your safety.

Possible **risks from Pregnancy:** If you are pregnant, breast-feeding or want to become pregnant in the next 24 weeks, you cannot take part in this study. We do not know the possible effects of the study vaccine on the fetus or nursing infant. Women who are able to become pregnant must have a negative pregnancy test before getting the study injection. They must

use an effective form of birth control beginning at least 21 days before getting the study injection until 24 weeks after the last study injection. Effective methods of birth control include: male or female condoms, with or without a spermicide; diaphragm or cervical cap with spermicide; intrauterine device; all prescription methods (like contraceptive pills, injections, patches and others); or a male partner who has had a vasectomy. You must tell the clinic staff right away if you become pregnant during this study. You must also tell the clinic if you think that you **might** be pregnant during this study. If you become pregnant, you will be asked to continue with the planned study follow-up visits. We will contact you later to learn about the outcome of any pregnancy that starts in the first 24 weeks after study vaccination.

***Other* Risks:** We do not know if the study vaccine may change your response if you ever have an Ebola virus infection in the future.

We will tell you if we learn that the vaccine causes significant health problems or serious side effects. We will continue to update you during the trial as needed.

You may not donate blood at a blood bank while taking part in this research study. You may not donate blood for one year after your last experimental vaccine injection.

POSSIBLE BENEFITS

This study is not designed to benefit you. We do not know if the vaccine will work. The study is not designed to protect you from Ebola infection. You and others may benefit in the future from the information learned from this study.

COSTS TO YOU FOR YOUR PARTICIPATION

The purpose of all of your health checkes in this clinical trial is for research, not to provide health care. However, we will tell you right away if any of your test results show a health problem. There are no costs to you for taking part in this study.

We will not charge you or your insurance carrier for any health checks or services provided at [insert site name]. You (or your insurance carrier) will be responsible for the costs of all medical care that you get elsewhere.

You will get compensation consistent with [site name] policy to help you with transportation costs and other expenses that may occur because of your study participation. It is possible that you may have some expenses that are not covered by the compensation provided.

COMPENSATION TO YOU FOR YOUR PARTICIPATION

[Adapt to site plan]: You will be compensated [$] for each visit that does not include an injection but does include a blood draw and [$] for the injection visit. For visits that do not include an injection or a blood draw, you will be compensated [$]. Compensation for timely completion of all 7 days of an electronic diary will be [$] total. The approximate total compensation is between [$] and [$]. Actual compensation will depend upon the visits you complete. You will be paid during the study after each visit.

Compensation suggested by the study sites:

Site	Clinic visit	Visit including vaccination	Phone follow-up	Electronic diary card completion	Bonus for study completion	Total compensation
VRC*	$175	$275		$25		Between $1775 and $2950 (depending on the study group)
UMD	$50	$150	$25	$25	$200	Up to $800
Emory	$50	$150				Up to $550
*Higher compensation rates are provided by VRC due to difficulties with travel and additional time and efforts needed for security on the NIH campus.						

REASONS FOR REMOVING YOU FROM THE STUDY WITHOUT YOUR CONSENT

Your participation in the study may be stopped for several different reasons, including:

- You don't keep appointments or follow study procedures.
- The study sponsor or study doctor decides to stop or cancel the study.
- The regulatory boards or the FDA decide that the study should be stopped.
- You get a serious illness.
- You need to get treatment with a medication that affects your immune system (such as a steroid like prednisone).

If you agree to take part in this study, it is important for you to keep all your appointments. However, if you don't want to stay in the study, you can leave at any time. You will not lose any benefits that you would have received if you did not join the study.

ALTERNATIVES

This study is not designed to treat or prevent any disease. You may choose to not participate.

CONFLICT OF INTEREST

The NIH research staff is checked every year for conflicts of interest. You may ask the research team for more information.

The NIH and some members of the VRC scientific staff developed the experimental Ebola vaccines in this research study. The results of this study could play a role in if the FDA will approve the vaccine for sale at some time in the future. If approved, the future sale of the vaccine could lead to payments to NIH and some NIH scientists. By U.S. law, government scientists are required to receive such payments for their inventions.

You will not receive any money from the development or sale of the product.

This protocol may have investigators who are not NIH employees. Non-NIH investigators are expected to follow the principles of the Protocol Review Guide but are not required to report their personal financial holdings to the NIH.

OTHER PERTINENT INFORMATION

1. Confidentiality. When results of an NIH research study are reported in medical journals or at scientific meetings, the people who take part are not named and identified. In most cases, the NIH will not release any information about your research involvement without your written permission. However, if you sign a release of information form, for example, for an insurance company, the NIH will give the insurance company information from your medical record. This information might affect (either favorably or unfavorably) the willingness of the insurance company to sell you insurance.

The Federal Privacy Act protects the confidentiality of your NIH medical records. However, you should know that the Act allows release of some information from your medical record without your permission, for example, if it is required by the Food and Drug Administration (FDA), members of Congress, law enforcement officials, or authorized hospital accreditation organizations.

2. Policy Regarding Research-Related Injuries. The [study site] will provide short-term medical care for any injury resulting from your participation in research here. In general, no long-term medical care or financial compensation for research-related injuries will be provided by the National Institutes of Health, the [study site], or the Federal Government. However, you have the right to pursue legal remedy if you believe that your injury justifies such action.

3. Payments. The amount of payment to research volunteers is guided by [site] policies. Reimbursement of travel and subsistence will be offered consistent with guidelines.

4. Problems or Questions. If you have any problems or questions about this study or about any research-related injury, contact the Principal Investigator, [insert PI name; telephone] or the Study Coordinator, [insert name; telephone].

If you have any questions about your rights as a research subject, you may call [insert contact information].

5. Consent Document. Please keep a copy of this document in case you want to read it again.

COMPLETE APPROPRIATE ITEM(S) BELOW:

Adult Study Participant's Consent

I have read the explanation about this study and have been given the opportunity to discuss it and to ask questions. I hereby consent to take part in this study.

_______ Time

______________________________________ _________
Signature of Adult Participant/Legal Representative Date

____
Print Name

THIS CONSENT DOCUMENT HAS BEEN APPROVED FOR USE FROM XXXXXX THROUGH XXXXXX.

______________________ ________	______________________ ________
Signature of Investigator/ Person Obtaining Consent Date	Signature of Witness Date Date
______________________________	______________________________
Print Name	Print Name

APPENDIX 2. CONTACT INFORMATION

APPENDIX 3. SCHEDULE OF EVALUATIONS

Schedule 1: Groups 1 and 2													
**Visit**		01	[1]01R	02	02A	02B	02D	03	04	05	08	09	11
**Week of Study**		-8 to 0	-2 to 0	W 0	W1	W1	W1	W2	W4	W8	W16	W24	W48
**[1]Day of Study**		-56 to -1	-14 to 0	D 0	D 1	D 2	D 7	D 14	D 28	D 56	D 112	D 168	D 336
**Clinical Evaluations**	**Tube**	Screen											
VRC 500 Screening Consent		X											
VRC 208 AoU, Informed Consent			[1]X										
Physical exam and weight at screen; Vital signs, targeted exam at other visits.		X	X	X		X	X	X	X	X	X	X	X
Medical history for eligibility at screen; interim history other visits		X	X	X		X	X	X	X	X	X	X	X
[1] ECG		X											
[2] Study Vaccination				X									
Begin 7-Day Diary Card				X									
Telephone contact; clinic visit if indicated					X								
Counseling on pregnancy prevention				X					X	X	X		
CBC	EDTA	3		3		3		3	3	3		3	
[3] Pregnancy test: urine (or serum)		X		X					X			X	
Creatinine and ALT	GLT	4		4		4		4	4	4			
HIV		4											
[4] PT and aPTT	Blue	4		4		4		4	4				
[5] HLA type	EDTA										20		
**Research Immunology***													
Antibody assays and serum storage	SST	16		24			16	16	24	24	24	24	24
PBMC and plasma for storage	EDTA	40		60			60	40	80	60	60	80	80
Intracellular RNA	PAXgene			3		6	6						
**Daily Volume (mL)**		**71**		**98**		**17**	**82**	**67**	**115**	**91**	**1**	**107**	**104**
**Max. Cumulative Volume (mL)**		**71**		**169**		**186**	**268**	**335**	**450**	**541**	**645**	**752**	**856**

[1] VRC 500: Most screening evaluations must be no more than 56 days prior to enrollment. Study enrollment, conducted at Visit 01R, may be up to 2 weeks before Visit 02 or both visits may be on the same date. If clinical assessment on Day 0 suggests the subject is not well enough to be vaccinated, then the site may reschedule Visit 02. Day 0=day of vaccine injection. Day 0 evaluations prior to first injection are the baseline for assessing adverse events subsequently except that the baseline ECG is performed at screening. Day 0=day of vaccine injection. Day 0 evaluations prior to study injection are the baseline for assessing adverse events subsequently.

[2] Complete post vaccination blood pressure, pulse and injection site assessment after the study injection (target to complete this within the 15 to 60 minute interval post-vaccination). Subjects in Groups 1 and 2 must remain in the clinic for 3 hours after injection; subsequent subjects must remain in the clinic for at least 1 hour after injection.

[3] Negative pregnancy test results must be confirmed for women of reproductive potential prior to administering the vaccine injection.

[4] Use 4.5 mL Blue top citrate tube; 3 mL minimum needs to be collected for accuracy of results. Additional tests may be needed if results are not in range.

[5] HLA type blood sample is collected once at any timepoint in the study and is shown as a Visit 04 evaluation for convenience; however, if HLA type is already available in the medical record it does not need to be repeated. HLA type may also be obtained from a frozen sample.

Visit windows: 02A (+1 day); 02B (+1 day); 02D (±1 day); Visit 03 (±3 days); Visits 04 and 05 (±7 days); Visits 08, 09 and 11 (±14 days). Visit numbers 02C, 06, 07, and 10 are intentionally excluded from Schedule 1.

*The volume of research blood drawn may be greater than shown on the SOE; cumulative draw cannot exceed allowance of Med.Admin. Series 95-9 or about 15 tubes per visit.

Schedule 2: Groups 4, 5, 6, and 7												
**Visit**		[1]01R	02	02A	02B	02C	03	04	05	08	09	11
**Week of Study**		-2 to 0	W 0	W1	W1	W1	W2	W4	W8	W16	W24	W48
**[1]Day of Study**		-14 to 0	D 0	D 1	D 2	D 5	D 14	D 28	D 56	D 112	D 168	D 336
**Clinical Evaluations**	**Tube**											
VRC 208 AoU, Informed Consent		X										
Physical exam and weight at 01R; Vital signs, targeted exam at other visits.		X	X		X	X	X	X	X	X	X	X
Interim medical history		X	X		X	X	X	X	X	X	X	X
[1] ECG		X										
[2] Study Vaccination			X									
Begin 7-Day Diary Card			X									
Phone contact; clinic visit if indicated				X								
Counseling on pregnancy prevention			X					X	X	X		
CBC	EDTA		3		3		3	3	3		3	
[3] Pregnancy test: urine (or serum)			X					X			X	
Creatinine and ALT	GLT		4		4		4	4	4			
[4] PT and PTT	Blue		4		4		4	4				
[5] HLA type	EDTA											
**Research Immunology***												
Antibody assays and serum storage	SST		24			16	16	24	24	24	24	24
PBMC and plasma for storage	EDTA		100			80	40	100	50	60	80	80
Intracellular RNA	PAXgene		3		6	6						
**Daily Volume (mL)**			**138**		**17**	**102**	**67**	**135**	**81**	**84**	**107**	**104**
**Max. Cumulative Volume (mL)**			**138**		**155**	**257**	**324**	**459**	**540**	**624**	**731**	**835**

[1] Enrollment is completed at Visit 01R, which may be up to 2 weeks before the study injection. Day 0=day of vaccine injection. Day 0 evaluations prior to study injection are the baseline for assessing adverse events subsequently, except the baseline ECG is at Visit 01R. Visit 01R and Visit 02 may be the same date.
[2] Complete post vaccination blood pressure, pulse and injection site assessment after the study injection (target to complete this within the 15 to 60 minute interval post-vaccination). Subjects must remain in the clinic for at least 1 hour after injection.
[3] Negative pregnancy test results must be confirmed for women of reproductive potential within 24 hours prior to administering the vaccine injection.
[4] Use 4.5 mL Blue top citrate tube; 3 mL minimum needs to be collected for accuracy of results. Additional tests may be needed if results are not in range.
[5] HLA type will be collected from VRC 207 data. HLA type may also be obtained from a frozen sample.
Visit windows: 02A (+1 day); 02B (+1 day); 02C (+2 days); Visit 03 (±3 days); Visits 04 and 05 (±7 days); Visits 08, 09 and 11 (±14 days). Visit numbers 06, 07, and 10 are intentionally excluded from Schedule 2.
*The volume of research blood drawn may be greater than shown on the SOE; cumulative draw cannot exceed allowance of Med.Admin. Series 95-9 or about 15 tubes per visit. Previous blood draws in VRC 207 should be taken in consideration for cumulative blood draw volume; the volume of research blood draw should be adjusted if needed.

Schedule 3: Group 3 Prime									
**Visit**		01	[1]01R	02	02A	02B	02D	03	04
**Week of Study**		-8 to 0	-2 to 0	W 0	W1	W1	W1	W2	W4
**[1]Day of Study**		-56 to -1	-14 to 0	D 0	D 1	D 2	D 7	D 14	D 28
**Clinical Evaluations**	**Tube**								
VRC 500 Screening Consent		X							
VRC 208 AoU, Informed Consent			[1]X						
Physical exam and weight at screen; Vital signs, targeted exam at other visits.		X	X	X		X	X	X	X
Medical history for eligibility at screen; interim history other visits		X	X	X		X	X	X	X
[1]ECG		[1]X							
[2] Study Vaccination				**X**					
Begin 7-Day Diary Card				X					
Telephone contact; clinic visit if indicated					X				
Counseling on pregnancy prevention				X					X
CBC	EDTA	3		3		3		3	3
[3] Pregnancy test: urine (or serum)		X		X					X
Creatinine and ALT	GLT	4		4		4		4	4
HIV		4							
[4] PT and PTT	Blue	4		4		4		4	4
**Research Immunology***									
Antibody assays and serum storage	SST	16		24			16	16	24
PBMC and plasma for storage	EDTA	40		60			60	40	80
Intracellular RNA	PAXgene			3		6	6		
**Daily Volume (mL)**		**71**		**98**		**17**	**82**	**67**	**115**
**Max. Cumulative Volume (mL)**		**71**		**169**		**186**	**268**	**335**	**450**

[1] VRC 500: Most screening evaluations must be no more than 56 days prior to enrollment. Study enrollment conducted at Visit 01R may be up to 2 weeks before Visit 02 or both visits may be on the same date. If clinical assessment on Day 0 suggests the subject is not well enough to be vaccinated, then the site may reschedule visit 02. Day 0=day of vaccine injection. Day 0 evaluations prior to first injection are the baseline for assessing adverse events subsequently except that the baseline ECG is performed at screening.
[2] Complete post vaccination blood pressure, pulse and injection site assessment after the study injection (target to complete this within the 15 to 60 minute interval post-vaccination). Subjects must remain in the clinic for at least 1 hour after injection.
[3] Negative pregnancy test results must be confirmed for women of reproductive potential with 24 hours prior to administering the vaccine injection.
[4] Use 4.5 mL Blue top citrate tube; 3 mL minimum needs to be collected for accuracy of results. Additional tests may be needed if results are not in range.
Visit windows: 02A (+1 day); 02B (+1 day); 02D (±1 day); Visit 03 (±3 days); Visit 04 (±7 days). Timeline for Visits 02A-04 is calculated relatively to Visit 02.
*The volume of research blood drawn may be greater than shown on the SOE; cumulative draw cannot exceed allowance of Med.Admin. Series 95-9 or about 15 tubes per visit.

**Schedule 3 Continuation: Group 3 Boost**											
**Visit**		05	05A	05B	05C	06	07	08	09	10	12
**Week of Study**		W8	W8	W8	W8	W10	W12	W16	W24	W32	W56
**[1]Day of Study**		D56	D57	D58	D61	D70	D84	D112	D168	D224	D392
**Clinical Evaluations**	**Tube**										
Physical exam and weight at screen; Vital signs, targeted exam at other visits.		X		X	X	X	X	X	X	X	X
Medical history for eligibility at screen; interim history other visits		X		X	X	X	X	X	X	X	X
[1] Study Vaccination		**X**									
Begin 7-Day Diary Card		X									
Telephone contact; clinic visit if indicated			X								
Counseling on pregnancy prevention		X				X	X	X	X		
CBC	EDTA	3		3		3	3	3		3	
[2] Pregnancy test: urine (or serum)		X					X			X	
Creatinine and ALT	GLT	4		4		4	4	4			
[3] PT and PTT	Blue	4		4		4	4				
[4] HLA type	EDTA					20					
**Research Immunology**											
Antibody assays and serum storage	SST	24			16	16	24	24	24	24	24
PBMC and plasma for storage	EDTA	60			60	40	80	60	60	80	80
Intracellular RNA	PAXgene	3		6	6						
**Daily Volume (mL)**		**98**		**17**	**82**	**87**	**115**	**91**	**84**	**107**	**104**
**Max. Cumulative Volume (mL)**		**548**		**565**	**647**	**734**	**849**	**940**	**1024**	**1131**	**1235**

[1]Complete post vaccination blood pressure, pulse and injection site assessment after the study injection (target to complete this within the 15 to 60 minute interval post-vaccination). Subjects must remain in the clinic for at least 1 hour after injection.
[2] Negative pregnancy test results must be confirmed for women of reproductive potential prior to administering the vaccine injection.
[3] Use 4.5 mL Blue top citrate tube; 3 mL minimum needs to be collected for accuracy of results. Additional tests may be needed if results are not in range.
[4] HLA type blood sample is collected once at any timepoint in the study and is shown as a Visit 06 evaluation for convenience; however, if HLA type is already available in the medical record it does not need to be repeated. HLA type may also be obtained from a frozen sample.
Visit windows: Visit 05 (±14 days); 05A (+1 day); 05B (+1 day); 05C (+2 days); Visit 6 (±3 days); Visits 07 and 08 (±7 days), Visits 09, 10 and 12 (±14 days). Timeline for Visits 05A-12 is calculated relatively to Visit 05. Visit numbers 02C and 11 are intentionally excluded from Schedule 3.
*The volume of research blood drawn may be greater than shown on the SOE; cumulative draw cannot exceed allowance of Med.Admin. Series 95-9 or about 15 tubes per visit.

APPENDIX 4. TABLE FOR GRADING SEVERITY OF ADVERSE EVENTS

Assessment of Causality Relationship of an Adverse Event (AE) to Study Vaccine:

The relationship between an AE and the vaccine will be assessed by the investigator on the basis of his or her clinical judgment and the definitions below.

- **Definitely Related**. The AE and administration of study agent are related in time, and a direct association can be demonstrated.
- **Probably Related**. The AE and administration of study agent are reasonably related in time, and the AE is more likely explained by study agent than other causes.
- **Possibly Related**. The AE and administration of study agent are reasonably related in time, but the AE can be explained equally well by causes other than study agent.
- **Not Related**. There is not a reasonable possibility that the AE is related to the study agent.

For purposes of preparing data reports in which AE attributions are limited to "**Related**" or "**Not Related**", in this protocol, the "Definitely, Probably and Possibly" attributions will be mapped to the "Related" category. The definitions that apply when these two categories alone are used are as follows:

- **Related** – There is a reasonable possibility that the AE may be related to the study agent.
- **Not Related** – There is not a reasonable possibility that the AE is related to the study agent.

Grading the Severity of Adverse Events:
The FDA Guidance for Industry (September 2007): "Toxicity Grading Scale for Healthy Adult and Adolescent Volunteers Enrolled in Preventive Vaccine Clinical Trials" is the basis for the severity grading of adverse events in this protocol. Several modifications were made to the table as follows:

- "Emergency room visit" is not automatically considered a life-threatening event; these words have been removed from any "grade 4' definition where they appear in the table copied from the guidance document.
- Any laboratory value shown as a "graded" value in the table that is within the institutional normal range will not be severity graded or recorded as an adverse event.
- When a gap in grade-specific calculated laboratory values would have occurred, the higher grade was extended to include values greater than the lower grade.
- Severity grading for hemoglobin decrease on the basis of the magnitude of decrease from baseline is not applicable at the grade 1 level; only absolute hemoglobin will be used to define grade 1 decrease. Increases in hemoglobin are AEs only for values above the upper limit of normal and are graded by the systemic illness clinical criteria.
- Severity grading definition for Grade 4 local reaction to injectable product (Erythema/Redness and Induration/Swelling) included added text "requiring medical attention".
- 1 X ULN was removed from the definition for PT increase.
- Severity grading definition for hypotension includes added clarifications such that an asymptomatic low blood pressure reading is not an adverse event.

When not otherwise specified in the table, the following guidance will be used to assign a severity grade:

Grade 1 (Mild): No effect on activities of daily living
Grade 2 (Moderate): Some interference with activity not requiring medical intervention
Grade 3 (Severe): Prevents daily activity and requires medical intervention
Grade 4 (Life-threatening): Hospitalization; immediate medical intervention or therapy required to prevent death.
Grade 5 (Death): Death is assigned a Grade 5 severity.
Only the single adverse event that is assessed as the primary cause of death should be assigned "grade 5" severity.

Toxicity Grading Scale for Healthy Adult and Adolescent Volunteers Enrolled in Preventive Vaccine Clinical Trials
Modified from FDA Guidance - September 2007

A. Tables for Clinical Abnormalities

**Local Reaction to Injectable Product**	**Mild (Grade 1)**	**Moderate (Grade 2)**	**Severe (Grade 3)**	**Potentially Life Threatening (Grade 4)**
Pain	Does not interfere with activity	Repeated use of non-narcotic pain reliever > 24 hours or interferes with activity	Any use of narcotic pain reliever or prevents daily activity	Hospitalization
Tenderness	Mild discomfort to touch	Discomfort with movement	Significant discomfort at rest	Hospitalization
[1]Erythema/Redness	2.5 – 5 cm	5.1 – 10 cm	> 10 cm	Necrosis or exfoliative dermatitis requiring medical attention
[2] Induration/Swelling	2.5 – 5 cm and does not interfere with activity	5.1 – 10 cm or interferes with activity	> 10 cm or prevents daily activity	Necrosis requiring medical attention
**[3] Vital Signs**	**Mild (Grade 1)**	**Moderate (Grade 2)**	**Severe (Grade 3)**	**Potentially Life Threatening (Grade 4)**
[4] Fever (°C)<br>(°F)	38.0 – 38.4<br>100.4 – 101.1	38.5 – 38.9<br>101.2 – 102.0	39.0 – 40<br>102.1 – 104	> 40<br>> 104
Tachycardia - beats per minute	101 – 115	116 – 130	> 130	Hospitalization for arrhythmia
[5] Bradycardia - beats per Minute	50 – 54	45 – 49	< 45	Hospitalization for arrhythmia
Hypertension (systolic) - mm Hg	141 – 150	151 – 155	> 155	Hospitalization for malignant hypertension
Hypertension (diastolic) - mm Hg	91 – 95	96 – 100	> 100	Hospitalization for malignant hypertension
Hypotension (systolic) – mm Hg	85 – 89 and symptomatic	80 – 84 and symptomatic and requiring oral fluids	< 80 and symptomatic and requiring IV fluids	Hospitalization for hypotensive shock
Respiratory Rate – breaths per minute	17 – 20	21 – 25	> 25	Intubation

1. In addition to grading the measured local reaction at the greatest single diameter, the measurement should be recorded as a continuous variable.
2. Induration/Swelling should be evaluated and graded using the functional scale as well as the actual measurement.
3. Subject should be at rest for all vital sign measurements.
4. Oral temperature; no recent hot or cold beverages or smoking.
5. When resting heart rate is between 60 – 100 beats per minute. Use clinical judgment when characterizing Bradycardia among some healthy subject populations, for example, conditioned athletes.

Systemic (General)	Mild (Grade 1)	Moderate (Grade 2)	Severe (Grade 3)	Potentially Life Threatening (Grade 4)
Nausea/vomiting	No interference with activity or 1 – 2 episodes/24 hours	Some interference with activity or > 2 episodes/24 hours	Prevents daily activity, requires outpatient IV hydration	Hospitalization for hypotensive shock
Diarrhea	2 – 3 loose stools or < 400 gms/24 hours	4 – 5 stools or 400 – 800 gms/24 hours	6 or more watery stools or > 800gms/24 hours or requires outpatient IV hydration	Hospitalization
Headache	No interference with activity	Repeated use of non-narcotic pain reliever > 24 hours or some interference with activity	Significant; any use of narcotic pain reliever or prevents daily activity	Hospitalization
Fatigue	No interference with activity	Some interference with activity	Significant; prevents daily activity	Hospitalization
Myalgia	No interference with activity	Some interference with activity	Significant; prevents daily activity	Hospitalization

Systemic Illness	Mild (Grade 1)	Moderate (Grade 2)	Severe (Grade 3)	Potentially Life Threatening (Grade 4)
Illness or clinical adverse event (as defined according to applicable regulations)	No interference with activity	Some interference with activity not requiring medical intervention	Prevents daily activity and requires medical intervention	Hospitalization

B. Tables for Laboratory Abnormalities

**Serum ***	**Mild (Grade 1)**	**Moderate (Grade 2)**	**Severe (Grade 3)**	**Potentially Life Threatening (Grade 4)**
Sodium – Hyponatremia mEq/L	132 – 134	130 – 131	125 – 129	< 125
Sodium – Hypernatremia mEq/L	144 – 145	146 – 147	148 – 150	> 150
Potassium – Hyperkalemia mEq/L	5.1 – 5.2	5.3 – 5.4	5.5 – 5.6	> 5.6
Potassium – Hypokalemia mEq/L	3.5 – 3.6	3.3 – 3.4	3.1 – 3.2	< 3.1
Glucose – Hypoglycemia mg/dL	65 – 69	55 – 64	45 – 54	< 45
Glucose – Hyperglycemia<br>Fasting – mg/dL<br>Random – mg/dL	 100 – 110<br>110 – 125	 111 – 125<br>126 – 200	 >125<br>>200	Insulin requirements or hyperosmolar coma
Blood Urea Nitrogen BUN mg/dL	23 – 26	27 – 31	> 31	Requires dialysis
Creatinine – mg/dL	1.5 – 1.7	1.8 – 2.0	2.1 – 2.5	> 2.5 or requires dialysis
Calcium – hypocalcemia mg/dL	8.0 – 8.4	7.5 – 7.9	7.0 – 7.4	< 7.0
Calcium – hypercalcemia mg/dL	10.5 – 11.0	11.1 – 11.5	11.6 – 12.0	> 12.0
Magnesium – hypomagnesemia mg/dL	1.3 – 1.5	1.1 – 1.2	0.9 – 1.0	< 0.9
Phosphorous – hypophosphatemia mg/dL	2.3 – 2.5	2.0 – 2.2	1.6 – 1.9	< 1.6
CPK – mg/dL	1.25–1.5 x ULN**	>1.5 – 3.0 x ULN	>3.0 –10 x ULN	> 10 x ULN
Albumin – Hypoalbuminemia g/dL	2.8 – 3.1	2.5 – 2.7	< 2.5	--
Total Protein – Hypoproteinemia g/dL	5.5 – 6.0	5.0 – 5.4	< 5.0	--
Alkaline phosphate – increase by factor	1.1 – 2.0 x ULN	>2.0 – 3.0 x ULN	>3.0 – 10 x ULN	> 10 x ULN
Liver Function Tests –ALT, AST increase by factor	1.1 – 2.5 x ULN	>2.5 – 5.0 x ULN	>5.0 – 10 x ULN	> 10 x ULN
Bilirubin – when accompanied by any increase in Liver Function Test increase by factor	1.1 – 1.25 x ULN	>1.25 – 1.5 x ULN	>1.5 – 1.75 x ULN	> 1.75 x ULN
Bilirubin – when Liver Function Test is normal; increase by factor	1.1 – 1.5 x ULN	>1.5 – 2.0 x ULN	>2.0 – 3.0 x ULN	> 3.0 x ULN
Cholesterol	201 – 210	211 – 225	> 225	---
Pancreatic enzymes – amylase, lipase	1.1 – 1.5 x ULN	>1.5 – 2.0 x ULN	>2.0 – 5.0 x ULN	> 5.0 x ULN

* The laboratory values provided in the tables serve as guidelines and are dependent upon institutional normal parameters. Institutional normal reference ranges should be provided to demonstrate that they are appropriate.
**ULN" is the upper limit of the normal range.

**Hematology ***	**Mild (Grade 1)**	**Moderate (Grade 2)**	**Severe (Grade 3)**	**Potentially Life Threatening (Grade 4)**
Hemoglobin (Female) - gm/dL	11.0 – 12.0	9.5 – 10.9	8.0 – 9.4	< 8.0
Hemoglobin (Female) decrease from baseline value - gm/dL	not applicable	1.6 – 2.0	2.1 – 5.0	> 5.0
Hemoglobin (Male) - gm/dL	12.5 – 13.5	10.5 – 12.4	8.5 – 10.4	< 8.5
Hemoglobin (Male) decrease from baseline value – gm/dL	not applicable	1.6 – 2.0	2.1 – 5.0	> 5.0
WBC Increase - cell/mm^3	10,800 – 15,000	15,001 – 20,000	20,001 – 25, 000	> 25,000
WBC Decrease - cell/mm^3	2,500 – 3,500	1,500 – 2,499	1,000 – 1,499	< 1,000
Lymphocytes Decrease - cell/mm^3	750 – 1,000	500 – 749	250 – 499	< 250
Neutrophils Decrease - cell/mm^3	1,500 – 2,000	1,000 – 1,499	500 – 999	< 500
Eosinophils - cell/mm^3	650 – 1500	1501 - 5000	> 5000	Hypereosinophilic
Platelets Decreased - cell/mm^3	125,000 – 140,000	100,000 – 124,000	25,000 – 99,000	< 25,000
PT – increase by factor (prothrombin time)	1.1 x ULN**	>1.1 – 1.2 x ULN	>1.2 – 1.25 x ULN	> 1.25 ULN
PTT – increase by factor (partial thromboplastin time)	1.1 – 1.2 x ULN	>1.2 – 1.4 x ULN	>1.4 – 1.5 x ULN	> 1.5 x ULN
Fibrinogen increase - mg/dL	400 – 500	501 – 600	> 600	--
Fibrinogen decrease - mg/dL	150 – 200	125 – 149	100 – 124	< 100 or associated with gross bleeding or disseminated intravascular coagulation (DIC)

* The laboratory values provided in the tables serve as guidelines and are dependent upon institutional normal parameters. Institutional normal reference ranges should be provided to demonstrate that they are appropriate.
**ULN" is the upper limit of the normal range.

PROTOCOL RV422
PART I & PART II

A PHASE IB, OPEN-LABEL, CLINICAL TRIAL TO EVALUATE THE SAFETY, TOLERABILITY AND IMMUNOGENICITY OF THE INVESTIGATIONAL EBOLA VACCINES, VRC-EBOADC069-00-VP (cAd3-EBO) AND VRC-EBOADC076-00-VP (cAd3-EBOZ), AND VRC-EBOMVA079-00-VP (MVA-EbolaZ), IN HEALTHY ADULTS IN KAMPALA, UGANDA

Study Agent Provided by
Vaccine Research Center/NIAID/NIH, Bethesda, MD

Study Conducted By
U.S. Military HIV Research Program, Silver Spring, MD
Makerere University-Walter Reed Project, Makerere University, Kampala, Uganda

In Collaboration with
Vaccine Research Center, NIAID, NIH

Study Sponsored By
National Institute of Allergy and Infectious Diseases (NIAID)

RV 422 PART I submitted to BB IND Number: 16,111;
PART II submitted to BB IND Number: 16,343 - both held by VRC, NIAID, NIH

**Protocol Co-Chair:**	**Merlin Robb, M.D.**
**Protocol Co-Chair**	**Julie Ledgerwood, D.O**
**Principal Investigator:**	**Hannah Kibuuka, M.D.**
**Local Medical Research Monitor:**	**Moses Kamya**

RV 422 PART I & PART II/WRAIR #2170
Version 2.2 FINAL
26 October 2015

TABLE OF CONTENTS

TABLE OF CONTENTS....2
LIST OF TABLES....4
SCHEMA....5
LIST OF ABBREVIATIONS....8
1. INTRODUCTION....10
1.1. Background: Ebola Infection....10
1.2. Rationale for Study Agent....11
1.3. Previous Human Experience with VRC Filovirus Vaccines....12
1.4. Previous Human Experience with Chimpanzee Adenovirus Vector Serotype 3 (cAd3)....15
2. STUDY AGENT....16
2.1. Background....16
2.2. Description of Study Agents....16
2.3. Preclinical Studies Supporting the Safety of VRC-EBOADC069-00-VP....17
2.4. Nonclinical Immunogenicity and Protection Studies of cAd3 Constructs....18
2.5. Effects in Humans....19
3. STUDY OBJECTIVES....20
3.1. Primary Objectives....20
3.2. Secondary Objectives....20
3.3. Exploratory Objectives....20
4. ENDPOINTS....21
4.1. Primary Endpoints: Safety....21
4.2. Secondary....21
4.3. Exploratory....21
5. STUDY DESIGN AND POPULATION....22
5.1. Eligibility....22
5.1.1. Inclusion Criteria for Part I....22
5.1.2. Exclusion Criteria for Part I....23
5.1.3. Criteria for Participation in Part II....24
6. STUDY PROCEDURES....26
6.1. Schedule of Evaluations....26
6.1.1. Recruitment....26
6.1.2. Consent Procedures and Screening....26
6.1.3. Day 0 through Week 48....28
6.2. Administration of the Study Injection....28
6.2.1. 7-Day Solicited Reactogenicity....29
6.2.2. Management of Reactogenicity Adverse Events Following Study Vaccinations....29
6.2.3. Management of laboratory abnormalities after vaccination through Study Week 4:....29
6.3. Concomitant Medications....30
6.4. Study Discontinuation....30
6.4.1. Early Discontinuation or Withdrawal of Study Participants....30
6.4.2. Management of Volunteers Who Become Pregnant....31

7. STUDY TREATMENT 32
7.1. Regimens 32
7.2. Administration 33
7.2.1. Duration 33
7.3. Study Product Formulation, Preparation, and Storage 33
7.3.1. Formulation 33
7.3.2. Study Agent Labels 33
7.3.3. Preparation 34
7.3.4. Storage 35
7.4. Pharmacy: Product Supply, Distribution, and Accountability 35
7.4.1. Study Product Acquisition/Distribution 35
7.4.2. Disposal of Clinical Supplies 36
8. PHARMACOVIGILENCE, SAFETY, AND ADVERSE EXPERIENCE REPORTING 37
8.1. Definitions 37
8.2. Adverse Event Grading and Recording 37
8.3. Protocol Safety Review Team (PSRT) and PSRT Reviews 38
8.4. Safety Monitoring Committee (SMC) Reviews 38
8.5. Criteria for Study Pause or Termination 38
8.6. Reporting Requirements to the Local IRB and Regulatory Bodies 39
8.7. Reporting Serious and Unexpected Adverse Events 40
8.7.1. Study Recording Period for SAEs 40
8.7.2. Reporting Serious and Unexpected Adverse Events to the Local Regulatory Bodies & Institutional Review Boards 40
8.8. Reporting Serious and Unexpected Adverse Events to WRAIR HSPB and MRMC 40
8.9. Unanticipated Problems Reporting 41
8.10. Adverse Event Reporting to the IND Sponsor 41
8.10.1. IND Sponsor Reporting to the FDA 42
9. STATISTICAL CONSIDERATIONS 43
9.1. Analysis 44
9.1.1. Primary Endpoints 44
9.2. Interim Analyses of Immunogenicity 45
10. DATA COLLECTION 46
11. ETHICAL CONSIDERATIONS 47
11.1. Participation Of Children 47
11.2. Risks 47
11.2.1. Risks of the cAd3 Ebola Vaccines and Diluent 47
11.2.2. Risks of the MVA-EbolaZ Vaccine 48
11.2.3. Other Risks 48
11.3. Benefits 48
11.4. Informed Consent 49
11.5. Language 49
11.6. Compensation 49
11.7. Local Medical Research Monitor (LMRM) 49
11.8. Policy Regarding Research-Related Injuries 50
11.9. Participant Confidentiality 50
11.10. Institutional Review Board 50

11.11. Future Use and Storage of Blood Samples 50
11.12. Study Documentation And Storage 51
12. ADMINISTRATIVE AND LEGAL PROCEDURES 52
12.1. Protocol Deviation Reporting 52
12.2. Protocol Modifications 52
12.3. Continuing Reviews /Closeout Report 53
12.4. Volunteer Registry Database 54
12.5. Use Of Information And Publication 54
13. CONDUCT OF THE RESEARCH STUDY 55
13.1. Regulatory Audits 55
13.2. Sponsor Study Monitoring 55
14. PRINCIPAL INVESTIGATOR AGREEMENT 56
15. REFERENCES 58
APPENDIX 1. SCHEDULE OF EVALUATIONS 61
APPENDIX 2. CONSENT FORMS 64
Appendix 2a: MAIN CONSENT FORM 65
Appendix 2b: INFORMED CONSENT FOR GENETIC TESTING 80
Appendix 2c: WITHDRAWAL OF CONSENT FOR SAMPLE STORAGE 84
Appendix 2d: INFORMED CONSENT FOR FUTURE USE OF STORED SPECIMENS ...86
APPENDIX 3. ASSESSMENT OF UNDERSTANDING 90
APPENDIX 4. BRIEFING SLIDES 93
APPENDIX 5. GRADING SEVERITY OF ADVERSE EVENTS 100
APPENDIX 6. STUDY TEAM ROSTER 106
APPENDIX 7. ROLES AND RESPONSIBILITIES 109
APPENDIX 8. DIARY CARDS 111

LIST OF TABLES

Table 1: RV 422 PART I & PART II Study Design 6
Table 2: Summary of the VRC Studies for Evaluation of Ebola and Marburg Vaccines 12
Table 3. Ebola Products and Identifier Numbers 16
Table 4. Preclinical Proof-of-Concept Studies in Cynomolgus Macaques 19
Table 5. RV 422 PART I & PART II Study Design 32
Table 6. Exact 95% Confidence Intervals for the true rate of events for selected sample sizes and number of observed events 43

SCHEMA

Title

A Phase Ib, Open-Label, Clinical Trial to Evaluate the Safety, Tolerability and Immunogenicity of the Ebola Chimpanzee Adenovirus Vector Vaccines, VRC-EBOADCC069-00-VP(cAd3-EBO) and VRC-EBOADC076-00-VP (cAd3-EBOZ), in Healthy Adults in Kampala, Uganda.

Study Design

This is a- two-part open-label study evaluating safety and immunogenicity. Ninety healthy adults in the Kampala area ages 18-65 will be randomized. Study duration is 48 weeks.

Part I:

Group 1: Sixty Ebola vaccine naïve subjects will be randomized to receive a single injection of vaccine as follows:

a) 15 subjects will receive cAd3-EBOZ (Zaire) at $1x10^{10}$ PU
b) 15 subjects will receive cAd3-EBOZ (Zaire) at $1x10^{11}$ PU
c) 15 subjects will receive cAd3-EBO (Zaire and Sudan) at $2x10^{10}$ PU
d) 15 subjects will receive cAd3-EBO (Zaire and Sudan) at $2x10^{11}$ PU

Group 2: Up to 30 eligible subjects who previously participated in the RV 247 vaccine clinical trial and received VRC-EBODNA023-00-VP (Ebola DNA WT) or in combination with VRC-MARDNA025-00-VP (Marburg DNA) will be randomized to receive a single injection of vaccine as follows:

a) 15 subjects will receive cAd3-EBO (Zaire and Sudan) at $2x10^{10}$ PU
b) 15 subjects will receive cAd3-EBO (Zaire and Sudan) at $2x10^{11}$ PU

Part II:

All subjects who complete at least 36 weeks of follow-up following the initial injection will be offered an optional boost with MVA-EbolaZ at $1x10^{8}$ PFU IM.

Table 1: RV 422 PART I & PART II Study Design

	RV 422 PART I & PART II Study Schema			
**Group**	**Sub-Group**	**Subjects**	**Part I: Day 0**	**Part II: Week 36+**
1		60		
	1a	15	cAd3-EBOZ at $1x10^{10}$ PU IM	MVA-EbolaZ at $1x10^{8}$ PFU IM
	1b	15	cAd3-EBOZ at $1x10^{11}$ PU IM	MVA-EbolaZ at $1x10^{8}$ PFU IM
	1c	15	cAd3-EBO at $2x10^{10}$ PU IM	MVA-EbolaZ at $1x10^{8}$ PFU IM
	1d	15	cAd3-EBO at $2x10^{11}$ PU IM	MVA-EbolaZ at $1x10^{8}$ PFU IM
2		30*		
	2a	15	cAd3-EBO at $2x10^{10}$ PU IM	MVA-EbolaZ at $1x10^{8}$ PFU IM
	2b	15	cAd3-EBO at $2x10^{11}$ PU IM	MVA-EbolaZ at $1x10^{8}$ PFU IM
Total		90		

Groups 1 and 2 will be enrolled simultaneously
cAd3-EBO and cAd3-EBOZ are administered in 1 mL volume with needle and syringe. MVA-EbolaZ is administered in 0.3 mL volume with needle and syringe. If a subject enrolls, but is not vaccinated, another subject may be enrolled to achieve the target number of administered vaccinations.
If less than 30 subjects are enrolled into Group 2, the remaining enrollment slots may be allocated to additional randomization slots in Group 1.
In Part II, the interval of time to receive the boost will vary but will be at least 36 weeks.

Sample Size

Sample size is 90 participants

Participants

Healthy adults in the Kampala area aged 18-65 years will be randomized.

Study Duration

Part I: Subjects will be evaluated at 9 clinic visits for up to 48 weeks.

Part II: Subjects will be evaluated at 11 clinic visits for over 48 weeks after beginning Part II.

Intervention

VRC-EBOADC069-00-VP (cAd3-EBO) is composed of two recombinant cAd3 vectors in a 1:1 ratio that express Ebola WT GPs from Zaire and Sudan strains. It is formulated at 2 x 10^{11} PU/mL. VRC-EBOADC076-00-VP [cAd3-EBOZ] is composed one vector that expresses Ebola WT GP from the Zaire strain. It is formulated at 1 x 10^{11} PU/mL. VRC-EBOMVA079-00-VP (MVA-EbolaZ) is composed of the attenuated replication-defective orthopoxvirus vector, MVA that expresses Ebola Zaire WT GP. It is formulated at 3.2 x 10^{8} PFU/mL.

Study Objectives

Primary Objectives

- To evaluate the safety and tolerability of VRC-EBOADC076-00-VP when administered IM at doses of 1 x 10^{10} particle units (PU) and 1 x 10^{11} particle units (PU) to healthy adults 18-65;

- To evaluate the safety and tolerability of VRC-EBOADC069-00-VP when administered IM at doses of 2 x 10^{10} particle units (PU) and 2 x 10^{11} particle units (PU) to healthy adults 18-65;
- To evaluate the safety and tolerability of prime-boost regimens of VRC-EBOMVA079-00-VP when administered IM at a dose of 1 x 10^{8} PFU to healthy adults 18-65.

Secondary Objectives

- To evaluate the Ebola GP-specific antibody responses to each vaccine/dose combination at 4 weeks after vaccination as assessed by vaccine-antigen specific ELISA and neutralization assays.
- To evaluate the Ebola GP-specific T cell responses to each vaccine/dose combination at 4 weeks after vaccination as assessed by ICS.
- To evaluate the cAd3 neutralizing antibody titers at baseline, Week 4 and Week 24.
- To evaluate the priming effect of a prior Ebola DNA vaccine administration (VRC-EBODNA023-00-VP) in Group 2 participants.
- To evaluate the antibody response to the cAd3-EBO/EBOZ prime - MVA-EbolaZ boost regimen at 4 weeks after the boost as assessed by Ebola GP-specific ELISA and neutralization assays.
- To evaluate the Ebola GP-specific T cell responses to the cAd3-EBO/EBOZ prime - MVA-EbolaZ boost regimen at 4 weeks after the boost as assessed by ICS.

Exploratory Objectives

- To evaluate the immunogenicity of each vaccine/dose combination by various assay methods at some or all of the research sample collection time points indicated in the Schedule of Evaluations; genetic factors associated with immune response may also be evaluated.
- To evaluate vaccine-induced mRNA expression profiles through Study Week 1.
- To evaluate the time course and durability of cAd3 and MVA neutralizing antibody titers, mediators of inflammation following vaccination and time course and durability of immune response by a variety of exploratory assays using samples collected throughout the study.
- To evaluate the cAd3- and MVA-specific antibodies prior to vaccination and at 4 weeks post vaccination.

LIST OF ABBREVIATIONS

Abbreviation	Term
Ad5	Human adenovirus serotype 5
APA	anti-phospholipid antibody
aPPT	activated partial thromboplastin time
BDBV	Species Bundibugyo ebolavirus
cAd	recombinant chimpanzee adenovirus
cAd3	recombinant chimpanzee adenovirus serotype 3
cAd63	recombinant chimpanzee adenovirus serotype 63
cGMP	current Good Manufacturing Practices
DNA	deoxyribonucleic acid
DS	drug substance
DP	drug product
EBOV	Species Zaire ebolavirus
EHF	Ebola hemorrhagic fever
ELISA	enzyme-linked immunosorbent assay
ELISPOT	enzyme-linked immunospot
EVD	Ebola virus disease
FDA	Food and Drug Administration
GCP	Good Clinical Practices
GLP	Good Laboratory Practices
GP	Glycoprotein
GP (S)	glycoprotein from *Sudan ebolavirus*
GP (Z)	glycoprotein from *Zaire ebolavirus*
HA	Influenza hemagglutinin protein
HAE	hereditary angioedema
HCV	hepatitis C vaccine
HIV	human immunodeficiency virus
ICS	intracellular cytokine staining
ICTV	International Committee on the Taxonomy of Viruses
IgG	Immunoglobulin G
IgM	Immunoglobulin M
IM	Intramuscular
IND	investigational new drug application
NIAID	National Institute of Allergy and Infectious Diseases
NIH	National Institutes of Health
NSAID	nonsteroidal anti-inflammatory drug
PBMC	peripheral blood mononuclear cells
PFU	particle forming units
PU	particle units
rAd	recombinant human adenovirus
rAd5	recombinant human adenovirus serotype 5
RESTV	Species *Reston ebolavirus*
RNA	ribonucleic acid
SUDV	Species *Sudan ebolavirus*
TAFV	Species Taï *Forest ebolavirus*
VRC	Vaccine Research Center
Vp	virus particle

WBC	white blood cell
WHO	World Health Organization
WT	Wild type

1. INTRODUCTION

1.1. Background: Ebola Infection

In 2013, the International Committee on the Taxonomy of Viruses (ICTV) Filoviridae Study Group and other experts published an updated taxonomy for filoviruses. The genus Ebolavirus is one of three genera in the family Filoviridae, which along with the genera, Marburgvirus and Cuevavirus, are known to induce viral hemorrhagic fever. Five distinct species included in the genus Ebolavirus are Bundibugyo (BDBV), Reston (RESTV), Sudan (SUDV), Taï Forest (TAFV), and Zaire (EBOV) [1].

Ebolavirus is a large, negative-strand RNA virus composed of 7 genes encoding viral proteins, including a single glycoprotein (GP) [2-4]. The virus is responsible for causing Ebola virus disease (EVD), formerly known as Ebola hemorrhagic fever (EHF), in humans. In particular, BDBV, EBOV, and SUDV have been associated with large outbreaks of EVD in Africa and reported case fatality rates of up to 90%[5]. Transmission of Ebola virus to humans is not yet fully understood, but is likely due to incidental exposure to infected animals [6-8]. EVD spreads through human-to-human transmission, with infection resulting from direct contact with blood, secretions, organs or other bodily fluids of infected people, and indirect contact with environments contaminated by such fluids [5].

EVD has an incubation period of 2 to 21 days (7 days on average, depending on the strain) followed by a rapid onset of non-specific symptoms such as fever, extreme fatigue, gastrointestinal complaints, abdominal pain, anorexia, headache, myalgias and/or arthralgias. These initial symptoms last for about 2 to 7 days after which more severe symptoms related to hemorrhagic fever occur, including hemorrhagic rash, epistaxis, mucosal bleeding, hematuria, hemoptysis, hematemesis, melena, conjunctival hemorrhage, tachypnea, confusion, somnolence, and hearing loss. Laboratory findings include low white blood cell and platelet counts and elevated liver enzymes [5]. In general, the symptoms last for about 7 to14 days after which recovery may occur. Death can occur 6 to 16 days after the onset of symptoms [6, 9] . People are infectious as long as their blood and secretions contain the virus; the virus was isolated from semen 61 days after onset of illness in a man who was infected in a laboratory [5].

Immunoglobulin M (IgM) antibodies to the virus appear 2 to 9 days after infection whereas immunoglobulin G (IgG) antibodies appear approximately 17 to 25 days after infection, which coincides with the recovery phase. In survivors of EVD, both humoral and cellular immunity are detected, however, their relative contribution to protection is unknown[10].

While prior outbreaks of EVD have been localized to regions of Africa, there is a potential threat of spread to other countries given the frequency of international travel. The 2014 outbreak in West Africa was first recognized in March 2014 and, as of August 13, 2014 the known case rate exceeded the largest prior EVD outbreak with a combined total (laboratory-confirmed, probable and suspected) 2127 cases and 1145 deaths (case fatality rate = 54%) (WHO-Disease Outbreak News). The largest prior outbreak occurred in Uganda in 2000-2001 with 425 cases and 224 deaths (case-fatality rate=53%) [11].

Viruses in the Filoviridae family are also categorized as potential threats for use as biological weapons due to ease of dissemination and transmission, and high levels of mortality. Currently, no effective therapies or FDA-licensed vaccines exist for any member of Filoviridae family of viruses.

1.2. Rationale for Study Agent

The VRC, NIAID, NIH developed a recombinant chimpanzee adenovirus Type 3-vectored Ebolavirus vaccine (cAd3-EBO), VRC-EBOADC069-00-VP. The vaccine encodes wild type (WT) GP from Zaire [GP (Z)] and Sudan [GP (S)] species of Ebola virus. The VRC also developed a recombinant chimpanzee adenovirus Type 3-vectored Ebolavirus vaccine (cAd3-EBOZ), VRC-EBOADC076-00-VP to encode WT GP (Z) alone. The rationale for the development of these vaccines is based on previous human experience with other investigational Filovirus vaccines (see previous human experience with the cAd3 vaccine vector), and preclinical studies of vaccine candidates against Filoviruses.

For the MVA-EbolaZ vaccine, an attenuated poxvirus strain, modified vaccinia virus Ankara (MVA), was used as a vector. The MVA was derived from a smallpox vaccine strain that after more than 570 passages in primary chicken embryo fibroblast (CEF) cells lost about 12% of the genome and became defective for replication in most mammalian cells [12-14]. In human cells, MVA produces both early and late viral proteins, but the viral assembly and thus production of infectious progeny and cell-to-cell viral transmission are impaired [13, 15]. The first successful attempt to use MVA as a vector for expression of foreign proteins in human cells was reported in 1992 [15]. Since then, MVA vector is being used in investigational vaccines against infectious diseases and cancer. Many of these investigational vaccines have been tested in Phase 1 clinical trials. As a result of these studies, the safety of MVA vector is well documented [13, 14].

A vaccination strategy to achieve protective immunity in most recipients with a single vaccination would be desirable in an outbreak setting. Vaccination strategies that achieve durable protective immunity would be desirable for populations in areas of the world where outbreaks occur sporadically. Optimally, one approach would serve both needs, but a different approach may be needed for rapid immunity than is needed for durable immunity. The current Phase 1 study conducted at VRC in Bethesda, MD, USA, VRC 207, is primarily directed at assessing safety and immunogenicity for the cAd3-EBO vaccine that may provide rapid immunity with a single injection. Boosting the Ugandan participants who, through RV 247, received a DNA Ebola vaccine (VRC-EBODNA023-00-VP) provides important information regarding delayed boosting with the new cAd3 vaccine and may provide a novel approach to generating long term protective immunity to Ebola.

Human clinical trials with the cAd3-EBO vaccine began on September 2, 2014 in the VRC 207 study. As of November 12, 2014, there have been no serious adverse events or clinical evidence of coagulopathy. Of note, prolonged PTT is an expected adverse event for adenoviral vector vaccines and is described in the Investigator’s Brochure as an *in vitro* effect on the lab assay for PTT. Three of the first 20 subjects in the VRC 207 study had asymptomatic prolonged PTT laboratory results at 2 weeks after vaccination. Evaluation of these prolonged PTT adverse events (AEs) showed them to be consistent with the induction of an antiphospholipid antibody (APA) and not of a coagulopathy. Comparable cases were observed in other adenoviral vector vaccine studies and note that this typically occurs about 2 weeks after vaccination in a subset of vaccine recipients. The APA effect on PTT resolves over several weeks without any clinical side effects in the vaccine recipients. Therefore, AEs of prolonged PTT should be excluded from study pause criteria unless there is concerning clinical evidence of coagulopathy.

The $2x10^{11}$ PU dose of cAd3-EBO vaccine is associated with more reactogenicity than the $2x10^{10}$ PU dose and the pattern is similar to other adenoviral vector vaccines. The early data suggest that about 70% of subjects will have at least one systemic reactogenicity symptom. Based on data to date, the reactogenicity typically occurs on the day of or day after vaccination and may include headache,

malaise, myalgia, with a smaller percentage also experiencing fever or chills. When present, fever onset was within one day of vaccination and resolved within 24 hours of onset. A pattern of fever, occurring later than one day after vaccination or lasting longer than a day, may require evaluation for additional etiology.

1.3. Previous Human Experience with VRC Filovirus Vaccines

The VRC, NIAID, NIH developed six different investigational Ebola vaccines and one Marburg virus vaccine, which were evaluated in a series of Phase 1 clinical trials. The vaccine constructs shown to be safe and immunogenic in the human clinical trials are listed in Table 2.

Table 2: Summary of the VRC Studies for Evaluation of Ebola and Marburg Vaccines

Study Identifier (Clinicaltrials.gov number)	Study Design	Vaccine Product (s)	Dosage, route, x N administrations	Accrual Product/Placebo
VRC 204 [16] (NCT00072605)	Phase I, randomized, placebo-controlled, dose escalation	VRC-EBODNA012-00-VP (Ebola DNA, ΔTM GP)	2 mg IM x 3 doses<br>4 mg IM x 3 doses<br>8 mg IM x 3 doses	5/2<br>8/2<br>8/2
VRC 205 [17] (NCT00374309)	Phase I, randomized, placebo-controlled, dose escalation	VRC-EBOADC018-00-VP (Ebola-rAd5, PM GP)	$2x10^9$ vp† IM x 1 dose<br>$2x10^{10}$ vp IM x 1 dose	12/4<br>12/4
VRC 206 [18] (NCT00605514)	Phase I, open label	VRC-EBODNA023-00-VP (Ebola DNA, WT GP)<br><br>VRC-MARDNA025-00-VP (Marburg DNA, WT GP)	4 mg IM x 3 or 4 doses<br><br>4 mg IM x 3 or 4 doses	10/0<br><br>10/0
RV 247 [19] (NCT00997607)	Phase Ib, randomized, placebo-controlled	VRC-EBODNA023-00-VP<br><br>VRC-MARDNA025-00-VP<br><br>VRC-EBODNA023-00-VP and VRC-MARDNA025-00-	4 mg IM x 3 doses<br><br>4 mg IM x 3 doses<br><br>4 mg IM x 3 doses; both vaccines	30/6<br><br>30/6<br><br>30/6

		VP		
VRC 207 [23]<br>(NCT02231866),<br>in progress	Phase I/Ib,<br>open label	VRC-EBOADC069-00-VP (cAd3-EBO, WT GP)<br><br>VRC-EBOADC076-00-VP (cAd3-EBOZ, WT GP)	2x10^{10} PU^ IM x 1dose<br>2x10^{11} PU IM x 1 dose<br><br>1x10^{10} PU IM x 1 dose<br>1x10^{11} PU IM x 1 dose	**10**/0<br><br>**112**/0<br><br>**10**/0<br><br>**10**/0
VRC 208<br>(NCT02408913),<br>in progress	Phase 1/1b, open label, dose escalation	VRC-EBOMVA079-00-VP<br>(MVA-EbolaZ)<br><br>VRC-EBOMVA079-00-VP<br><br>VRC-EBOADC069-00-VP +<br><br>VRC-EBOMVA079-00-VP<br><br>[Received in VRC 207]<br>[VRC-EBOADC069-00-VP]<br>[VRC-EBOADC069-00-VP]<br>[VRC-EBOADC076-00-VP]<br>[VRC-EBOADC076-00-VP]<br>+<br><br>VRC-EBOMVA079-00-VP	1x10^{7} PFU* IM x 1 dose<br><br>1x10^{8} PFU IM x 1 dose<br><br>(prime)<br>2x10^{11} PU IM x 1 dose<br>+<br>(boost)<br>1x10^{8} PFU IM x 1 dose<br><br>(prime)<br>2x10^{10} PU x 1 dose<br>2x10^{11} PU x 1 dose<br>1x10^{10} PU x 1 dose<br>1x10^{11} PU x 1 dose<br>+<br>(boost)<br>1x10^{8} PFU x 1 dose	**5**/0<br><br>**5**/0<br><br>**13**/0<br><br>**ongoing**<br>**(up to 140)**/0
† viral particles<br>^ particle units<br>* plaque forming units				

Each clinical trial contributed to product development and a better understanding of human immune responses to investigational vaccines. The first investigational vaccine study was initiated in 2003. This was a 3-plasmid recombinant DNA vaccine, VRC-EBODNA012-00-VP, that encoded for nucleoprotein (NP) from the Zaire strain of Ebola and for transmembrane deleted (ΔTM) Ebola GP sequences from Zaire and Sudan strains. At that time, deletion of the TM region of the GP was included in the vaccine construct design to address theoretical concerns related to cellular toxicity that had been observed during in vitro experiments when plasmids expressing full length, WT GP proteins were transfected into cell culture [21].

The second investigational vaccine evaluated was a replication-defective, recombinant adenovirus serotype 5 vaccine, VRC-EBOADC018-00-VP (Ebola-rAd5). The constructs in this vaccine encoded GP genes with a point-mutation [aspartic acid substituted for glutamic acid at position 71 (E71D)], which had been found to be safe and immunogenic in animal studies without in vitro or in vivo toxicity. The design of this vaccine had been based on observation of complete protection in

macaques vaccinated with a rAd vaccine encoding for E71D GP (Z) combined with E71D GP (S) and then challenged with Ebola Zaire species [9]. The Ebola-rAd5 vaccine was immunogenic and induced humoral and T cell responses to the point mutant GP inserts with a single vaccination, but pre-existing immunity to Ad5 partially blunted antibody responses to the vaccine antigen [17].

Non-human primate (NHP) studies, ongoing in a similar timeframe to the conduct of these human clinical trials, showed that ΔTM GP and PM GP antigens were partially protective against Ebola virus infection, but WT GP constructs provide the highest level of protection [22]. Therefore, subsequently the WT GP antigen became the focus of Ebola vaccine research and development at VRC/NIAID. The third investigational Ebola vaccine, VRC-EBODNA023-00-VP (Ebola DNA WT), was a 2-plasmid recombinant DNA vaccine encoding for the wild type (WT) GP of the Zaire and Sudan strains of Ebola virus. At the same time, a DNA vaccine encoding for GP of the Angola strain of Marburg virus, VRC-MARDNA025-00-VP (Marburg DNA), was also evaluated. The plasmid DNA vaccines encoding for Ebola WT GP and Marburg WT GP evaluated in both the VRC 206 and RV 247 studies were safe and immunogenic but repetitive vaccination with 3 or 4 doses was needed to achieve high response rates to some of the antigens. Importantly, the evaluation of WT GP constructs has not been associated with coagulopathy or serious adverse events.

All of the investigational vaccines listed in Table 2 were assessed as safe and well tolerated in Phase 1 clinical trials. On the basis of this work, assessment of an adenoviral vector, for which there is little to no pre-existing immunity in human populations, to deliver encoded Ebola WT GP sequences would be a promising approach.

Human clinical trials with VRC-EBOADC069-00-VP (cAd3-EBO) and VRC-EBOADC076-00-VP (cAd3-EBOZ) vaccines began on September 2, 2014 in the VRC 207 study. The safety and immunogenicity assessments for the first 20 subjects who received cAd3-EBO in dosages up to $2x10^{11}$ PU in this study have been described in a preliminary report [23]. Greater than 200 subjects have received the cAd3-EBOZ vaccine in ongoing international studies.

There have been no serious adverse events or clinical evidence of coagulopathy. Of note, prolonged PTT is an expected adverse event for adenoviral vector vaccines and is described in the Investigator's Brochure as an *in vitro* effect on the lab assay for PTT. Nine (12.9%) of the first 70 subjects in the VRC 207 study had asymptomatic prolonged PTT laboratory results at 2 weeks after vaccination. Evaluation of these prolonged PTT adverse events (AEs) showed these to be consistent with the induction of an antiphospholipid antibody (APA) and not of a coagulopathy. We have observed comparable cases in other adenoviral vector vaccine studies and note that this typically occurs about 2 weeks after vaccination in a subset of vaccine recipients. The APA effect on the PTT test resolves over several weeks without any clinical side effects.

The $2x10^{11}$ PU dose of cAd3-EBO vaccine is associated with more reactogenicity than the $2x10^{10}$ PU dose and this pattern is similar to other adenoviral vector vaccines. The early data suggest that about 60% of subjects will have at least one systemic and/or local reactogenicity symptom. Based on data as of 12/1/2014, the systemic reactogenicity typically occurs within a day after vaccination and may include headache, malaise, myalgia, chills, and fever; about 30% of subjects in the higher dose group experienced mostly mild or moderate fever. When present, fever onset was within one day of vaccination and resolved within 24 hours of onset. A pattern of fever, occurring later than one day after vaccination or lasting longer than a day, may require evaluation for additional etiology [23].

Evaluation of cAd3-vectored Ebola vaccines is ongoing domestically in VRC 208 as well as in international studies being conducted by others. VRC 208, the first human clinical trial with VRC-EBOMVA079-00-VP (MVA-EbolaZ), began on April 27, 2015. As of July 6, 2015, MVA-EbolaZ has been given to 40 subjects, both as a single injection at doses of $1x10^7$ PFU IM (n=5) and $1x10^8$

PFU IM (n=5) and as a boost to subjects who previously received cAd3-EBO and cAd3-EBOZ (n=30, ongoing).

To date, cAd3-EBO, cAd3-EBOZ, and MVA-EbolaZ have been evaluated as well tolerated and safe for further evaluation in humans.

1.4. Previous Human Experience with Chimpanzee Adenovirus Vector Serotype 3 (cAd3)

Recombinant chimpanzee adenovirus serotype 3 (cAd3) is a subgroup C adenovirus with properties similar to those of human adenovirus serotype 5 (Ad5). Both cAd3 and cAd63 vectors were initially considered for development of a new Ebolavirus vaccine based on immunological properties and that both vectors were used in investigational products that advanced to Phase 1 human clinical trials. Both the cAd3 and cAd63 were shown to be safe and immunogenic in human studies evaluating candidate vaccines for hepatitis C virus (HCV) [24] and malaria [25], respectively. After initial consideration of these adenoviral vectors, the cAd3 vector was chosen for development into an investigational vaccine for human clinical trials.

Serological studies showed a low seroprevalence in human sera for antibodies to cAd3 [26] [and unpublished VRC data], and when present, antibody titers are low. Ad5 pre-existing immunity did not appear to cross-react with cAd3 in mice [27] [and unpublished VRC data]. The cAd3-based vaccines were capable of inducing an immune response comparable to human Ad5 vectored vaccine 27,28]. VRC investigators showed that cAd3 vectors had similar levels of potency as rAd5 using multiple antigens including influenza HA and HIV Env [unpublished VRC data].

In a Phase I study, Barnes et al. tested two rare serotype adenovirus vectors, cAd3 and human adenovirus serotype 6, expressing HCV antigens in healthy volunteers. A total of 41 subjects were immunized by intramuscular (IM) injection with dosages up to $7.5x10^{10}$ viral particles (vp). Several prime-boost schedules were evaluated with 4-6 subjects per treatment group. Overall, these vaccines were assessed as safe and well tolerated. Mostly mild, self-limited local and systemic reactogenicity was observed which was dose-dependent but did not differ significantly between priming and boosting. The vaccinations induced HCV-specific immunity with broad specificity sustained for at least a year after the boost with heterologous adenoviral vector [24].

In summary, use of the recombinant, non-replicating cAd3 vector for construction of the cAd3-EBO vaccine is based on properties to induce immune responses as readily as rAd5, but with little to no pre-existing immunity in human populations. The encoded WT GP antigens were chosen based on preclinical data and may provide protective immunity against Ebolavirus species.

2. STUDY AGENT

2.1. Background

Three products will be tested in this study. The monotypic VRC-EBOADC076-00-VP (cAd3-EBOZ) will be tested at two dose formulations of 1 x 10^{10} PU/mL and 1 x 10^{11} PU/mL. The recombinant chimpanzee adenovirus Type 3-vectored Ebola vaccine VRC-EBOADC069-00-VP (cAd3-EBO), consists of a 1:1 ratio of two drug substances: recombinant replication-deficient adenovirus chimpanzee serotype 3 (cAd3) vectors expressing WT GP (Z) and GP (S). This bivalent vaccine will also be tested at two doses of 2 x 10^{10} PU/mL and 2 x 10^{11} PU/mL. Recombinant live VRC-EBOMVA079-00-VP (MVA-EbolaZ) vaccine consists of the attenuated replication-defective orthopoxvirus, modified vaccinia virus Ankara (MVA) that expresses Ebola Zaire WT glycoprotein. The drug substances were manufactured by Advent S.r.l., Pomezia, Italy (a subsidiary of Glaxo Smith Kline) and the vaccine was manufactured at the VRC Pilot Plant (VPP), operated under contract by the Vaccine Clinical Materials Program, Leidos Biomedical Research, Inc., Frederick, MD. The vaccines under investigation in protocol RV 422 PART I & PART II are shown in Table 3.

Table 3. Ebola Products and Identifier Numbers

VRC Product Number	Antigen Description	Viral Seed Stock ID (Plasmid Number)	Master Virus Bank Lot Number	Drug Substance Lot Number
VRC-EBOADC069-00-VP (cAd3-EBO)	EBO Zaire (EBO Z)	12-RD00013-PR (VRC 9827, Z-6614)	C.0002	C.0005
	EBO Sudan (EBO S)	12-RD00007-PR (SG-6611)	C.0001	C.0007
VRC-EBOADC076-00-VP (cAd3-EBOZ)	EBO Zaire (EBO Z)	12-RD00013-PR (VRC 9827, Z-6614)	C.0002	C.0005
VRC-EBOMVA079-00-VP (MVA-EbolaZ)	P94GP Ebola Zaire	Okairos 1151	0011113 (MSV) 0010114 (WSV)	LN 04

2.2. Description of Study Agents

VRC-EBOADC076-00-VP (cAd3-EBOZ)

The vaccine VRC-EBOADC076-00-VP (cAd3-EBOZ) is a sterile, aqueous, buffered solution composed of cAd3 EBO Z drug substances filled into single dose vials at 1 x 10^{11} PU/mL. The VRC-EBOADC076-00-VP product label designates the long-term storage temperature as $\leq$-60°C. The product should be allowed to thaw at room temperature before use and should be used within 4 hours.

VRC-EBOADC076-00-VP, cAd3-EBOZ, was manufactured at the VPP and is supplied in a 3 mL glass vial. Fill volume is 0.7 mL to allow withdrawal of 1.0 mL for IM administration.

VRC-EBOADC069-00-VP (cAd3-EBO)

The vaccine VRC-EBOADC069-00-VP (cAd3-EBO) is a sterile, aqueous, buffered solution composed of 1:1 ratio of cAd3 EBO Z and cAd3 EBO S drug substances filled into single dose vials at 1 x 10^{11} PU/mL each (2 x 10^{11} PU/mL total). The VRC-EBOADC069-00-VP product label designates the long-term storage temperature as $\leq$-60°C. The product should be allowed to thaw at room temperature before use and should be used within 4 hours.

VRC-EBOADC069-00-VP, cAd3-EBO, was manufactured at the VPP and is supplied in a 3 mL glass vial with >1 mL fill volume.

Additional VRC-EBOADC069-00-VP (cAd3-EBO) composition and manufacturing information can be found in the Investigator Brochure.

VRC-EBOMVA079-00-VP (MVA-EbolaZ)

The vaccine VRC-EBOMVA079-00-VP (MVA-EbolaZ) is a sterile, aqueous, buffered solution composed of MVA-EbolaZ drug substance filled into single dose vials at 3.2 x 10^8 PFU/mL. Vials are aseptically filled to a volume of 0.7 mL. The different dose levels are achieved by administering the appropriate volume directly from the vial or dilution to the appropriate dosage.

Additional VRC-EBOMVA079-00-VP (MVA-EbolaZ) composition and manufacturing information can be found in the Investigator's Brochure.

VRC-DILADC65-00-VP Formulation Buffer

The formulation buffer, pH 7.4, is composed of 10 mM Tris, 10 mM Histidine, 5% Sucrose (w/v), 75 mM Sodium Chloride, 1 mM Magnesium Chloride, 0.02% Polysorbate 80 (PS-80) (w/v), 0.1 mM EDTA, and 0.5% Ethanol (v/v). Vials have a fill volume of >1 mL to allow withdrawal of 1.0 mL. The different dose levels are achieved by administering the appropriate volume directly from the vial or dilution to the appropriate dosage.

Vials of diluent for preparation of the 1x10^{10} PU dose of cAd3-EBOZ or the 2x10^{10} PU dose of cAd3-EBO, for IM administration, is comprised of the formulation buffer. VRC-DILADC0056-00-VP is a sterile, buffered, aqueous solution filled into glass vials at a fill volume of >1 mL to allow withdrawal of 1.0 mL. The diluent product label designates the long-term storage temperature -45°C to -10°C.

Additional diluent VRC-DILADC065-00-VP composition and manufacturing information can be found in the Investigator Brochure.

2.3. Preclinical Studies Supporting the Safety of VRC-EBOADC069-00-VP

The March 2014 Ebola outbreak in West Africa [11] prompted the VRC/NIAID to accelerate initiation of the first clinical study of VRC-EBOADC069-00-VP. The vaccine vector, cAd3, was used in an investigational HCV vaccine product, AdCh3-HCV (BB-IND 14818, Okairos, Inc.), for which both nonclinical and clinical evaluations were performed by others and a nonclinical toxicology study with VRC cAd3-HIV Vaccine (VRC-HIVADC064-00-VP) was performed by VRC/NIAID. In addition, VRC/NIAID evaluated in both a GLP toxicity study and two Phase 1 clinical trials, a plasmid DNA vaccine, VRC-EBODNA023-00-VP (BB-IND 13609, RCHSPB), which encodes for the same Ebola Zaire and Sudan WT glycoproteins used in the cAd3-EBO vaccine.

In April 2014, the VRC/NIAID proposed to the FDA that these prior nonclinical and clinical studies support evaluation of cAd3-EBO through a staged, dose escalation Phase I study design without the conduct of a GLP toxicity study. The FDA had previously reviewed product information and testing at a pre-IND meeting conducted in August 2013. In May 2014, the FDA concurred with the VRC proposal to proceed with evaluation of the cAd3-EBO vaccine in a Phase 1clinical trial and no preclinical GLP toxicity study was conducted. Prior relevant nonclinical and clinical studies are summarized in the Investigator's Brochure.

In August 2014 the IND to begin evaluation of cAd3-EBO was reviewed and assessed as safe to proceed by the FDA. On the basis of consultation with collaborating researchers the VRC/NIAID also sought and received approval to evaluate the cAd3 EBO GP Zaire drug substance alone as a vaccine under the same IND. This is designated VRC-EBOADC076-00-VP (cAd3-EBOZ).

2.4. Nonclinical Immunogenicity and Protection Studies of cAd3 Constructs

Several non-GLP studies were performed in non-human primates (NHP), Cynomolgus macaques, to select the cAd3-based EBO constructs for further development and to provide animal proof-of-concept data necessary to enter Phase I clinical studies. Research-grade materials, made with the same constructs as clinical material, of cAd3 EBO Z (containing GP from Ebolavirus Zaire), cAd3 EBO S (containing GP from Ebolavirus Sudan) and/or cAd3-EBO (composed of a 1:1 ratio of cAd3 EBO Z and cAd3 EBO S) [28] were used in all studies summarized below in Table 4.

Evaluation of the prime-boost regiment of MVA-vectored Ebola vaccine administered as a boost to the cAd3-vectored Ebola vaccine prime demonstrated that durable protection has been achieved against lethal EBOV challenge in Cynomolgus macaques. Animals that received a single IM dose of MVA-based vaccine had detectable viremia by day 6 after challenge and none survived infection [20].

Table 4. Preclinical Proof-of-Concept Studies in Cynomolgus Macaques

Study Purpose	Study Outcome
Demonstrate protection against lethal challenge with Ebolavirus Zaire in Cynomolgous macaques after single IM dose of cAd3 EBO Z	100% protection after a single IM dose of cAd3 EBO Z at 10^{10} and 10^{11} vp
Demonstrate protection against lethal challenge with Ebolavirus Zaire in Cynomolgous macaques after single IM dose of cAd3-EBO	100% protection after a single IM dose of cAd3-EBO at $2x10^{10}$ vp<br><br>50% protection after cAd3-EBO at $2x10^{9}$ vp
Demonstrate protection against lethal challenge with Ebolavirus Sudan in Cynomolgous macaques after single IM dose of cAd3-EBO	100% protection against Ebolavirus Sudan after a single IM dose of cAd3-EBO at $2x10^{10}$ vp
Demonstrate generation of humoral and cellular immune responses after single IM dose of cAd3-EBO at $2x10^{9}$ or $2x10^{10}$ vp.	Single IM dose of cAd3-EBO at $2x10^{9}$ vp or $2x10^{10}$ vp elicited antibody and antigen-specific CD4+ and CD8+ T cell responses. The lower cellular immune responses correlated with the lower level of protection (50%) observed after the $2x10^{9}$ vp dose.
Demonstrate durability of the immune responses after single IM dose of cAd3 EBO Z at 10^{11} vp or cAd3-EBO at $2x10^{10}$ vp administered 10 months before the lethal challenge with Ebolavirus Zaire.	Protective immune responses measured during the acute phase of infection declined over time. When challenged 10 months after vaccination, 2/4 macaques were protected after a single IM dose of cAd3 EBO Z at 10^{11}vp while 0/4 were protected after a single IM dose of cAd3-EBO at $2x10^{10}$vp.
Demonstrate protection against lethal challenge with Ebolavirus Zaire in Cynomolgous macaques after prime with single IM dose of cAd3-EBO and week 8 boost with single IM dose of MVA (expressing GPs from EBOV and SUDV).	100% protection after a prime with a single IM dose of cAd3-EBO at 10^{10} VP and week 8 boost with a single IM dose of MVA (expressing GPs from EBOV and SUDV) at 10^{8} PFU

2.5. Effects in Humans

The study agent, MVA-EbolaZ, is currently being tested in VRC 208 study.

The VRC 208 protocol entitled “A Phase I/Ib Open-Label Clinical Trial to Evaluate Dose, Safety and Immunogenicity of a Recombinant Modified Vaccinia Virus Ankara Ebola Vaccine, VRC-EBOMVA079-00-VP (MVA-EbolaZ), Administered Alone or as a Boost to cAd3-Ebola Vaccines in Healthy Adults” is conducted at the National Institutes of Health (NIH). VRC 208 is a two-part Phase I/Ib, open-label, dose-escalation study to examine safety, tolerability and immunogenicity of the Ebola modified vaccinia virus Ankara vaccine (MVA-EbolaZ) administered alone or as a boost to the cAd3-EBO or cAd3-EBOZ vaccines in healthy adults. The hypotheses are that the study vaccine, MVA-EbolaZ will be safe and will elicit immune responses to Ebola GP, and that the prime-boost regime will be safe and result in a polyfunctional response to Ebola GP that is of greater magnitude and duration that response to either of the vaccines alone. Relevant VRC 208 study data is included in Section 1.3.

3. STUDY OBJECTIVES

3.1. Primary Objectives

- To evaluate the safety and tolerability of VRC-EBOADC076-00-VP when administered IM at doses of 1 x 10^{10} particle units (PU) and 1 x 10^{11} particle units (PU) to healthy adults 18-65;
- To evaluate the safety and tolerability of VRC-EBOADC069-00-VP when administered IM at doses of 2 x 10^{10} particle units (PU) and 2 x 10^{11} particle units (PU) to healthy adults 18-65;
- To evaluate the safety and tolerability of prime-boost regimens of VRC-EBOMVA09-00-VP when administered IM at a dose of 1 x 10^{8} PFU to healthy adults 18-65.

3.2. Secondary Objectives

- To evaluate the Ebola GP-specific antibody responses to each vaccine/dose combination at 4 weeks after vaccination as assessed by vaccine-antigen specific ELISA and neutralization assays.

- To evaluate the Ebola GP-specific T cell responses to VRC-EBOADC069-00-VP at 4 weeks after vaccination as assessed by ICS.

- To evaluate the cAd3 neutralizing antibody titers at baseline, Week 4 and Week 24.

- To evaluate the priming effect of a prior Ebola DNA vaccine administration (VRC-EBODNA023-00-VP) in Group 2 participants.

- To evaluate the antibody response to the cAd3-EBO/EBOZ prime- MVA-EbolaZ boost regimen at 4 weeks after the boost as assessed by Ebola GP-specific ELISA and neutralization assays.

- To evaluate the Ebola GP-specific T cell responses to the cAd3-EBO/EBOZ prime-MVA-EbolaZ boost regimen at 4 weeks after the boost as assessed by ICS.

3.3. Exploratory Objectives

- To evaluate the immunogenicity of each vaccine/dose combination by various assay methods at some or all of the research sample collection time points indicated in the Schedule of Evaluations; genetic factors associated with immune response may also be evaluated.
- To evaluate vaccine-induced mRNA expression profiles through Study Week 1.

- To evaluate the cAd3- and MVA-specific antibodies prior to vaccination and at 4 weeks post vaccination.

To evaluate the time course and durability of cAd3 and MVA neutralizing antibody titers, mediators of inflammation following vaccination and time course and durability of immune response by a variety of exploratory assays using samples collected throughout the study.

4. ENDPOINTS

4.1. Primary Endpoints: Safety

Assessment of product safety will include clinical observation and monitoring of clinical chemistry and hematology parameters. Safety will be closely monitored after injection and evaluated through a minimum of 36 weeks after initial study injection 48 weeks after the boost injection. The following parameters will be assessed for all study groups:

- Occurrence of solicited local reactogenicity signs and symptoms for 7 days following each vaccination
- Occurrence of solicited systemic reactogenicity signs and symptoms for 7 days following each vaccination
- Change from baseline for safety laboratory measures
- Occurrence of adverse events of all severities through 4 weeks after each study injection
- Occurrence of serious adverse events and new chronic medical conditions through the last study visit

4.2. Secondary

The primary immunogenicity endpoints are ELISA and neutralization antigen-specific assays for antibody responses and intracellular cytokine staining (ICS) assay for T cell responses. The principal time point for antibody is week 4 after vaccination. The time course of T cell responses will be evaluated in this study to determine peak response.

4.3. Exploratory

ELISA, neutralization assay, and ICS performed with research samples collected at study time points shown in the Schedule of Evaluations (Appendix 1), as well as other immunogenicity assays throughout the study and evaluation of genetic factors associated with immune responses, may be completed as exploratory evaluations. Monoclonal antibodies may be isolated from cryopreserved peripheral blood mononuclear cells (PBMC) and evaluated to determine among other characteristics, epitope specificity, and functional capacity. Vaccine-induced mRNA expression profiles through 1 week after vaccination may also be performed as an exploratory evaluation.

5. STUDY DESIGN AND POPULATION

This is a two-part Phase Ib, open-label study to evaluate the safety, tolerability and immunogenicity of a recombinant vector for the Ebola virus. This study will be conducted at the MUWRP in Kampala, Uganda.

Part I enrollment will begin with the randomization of a total of 90 healthy adults aged 18 years to 65 years to receive a single vaccine injection of cAd3-EBO or cAd3-EBOZ. Randomizations within Group 1 will include at least 60 Ebola vaccine naïve subjects. Randomizations within Group 2 may include up to 30 eligible subjects who previously participated in the RV 247 vaccine trial and received VRC-EBODNA023-00-VP (Ebola DNA WT) or in combination with VRC-MARDNA025-00-VP (Marburg DNA). If less than 30 subjects are enrolled into Group 2, the remaining enrollment slots may be allocated to Group 1. The study randomization and vaccination schema is shown in Table 1.

Part II participation is optional and may begin after subjects complete at least 36 weeks of Part I study follow-up.

5.1. Eligibility

5.1.1. Inclusion Criteria for Part I

A volunteer must meet all of the following criteria:

1. 18 to 65 years old.
2. Available for clinical follow-up through Week 48 after enrollment.
3. Able to provide proof of identity to the satisfaction of the study clinician completing the enrollment process.
4. Must be willing to be taken home at enrollment visit and allow home visits if participant does not keep appointments
5. Must complete an Assessment of Understanding (AoU) prior to enrollment by answering 9 out of 10 questions at least once in 3 attempts.
6. Able to read (English or Luganda) and willing to complete the informed consent process.
7. Willing to donate blood for sample storage to be used for future research.
8. In good general health without clinically significant medical history.
9. Physical examination and laboratory results without clinically significant findings and a body mass index (BMI) ≤ 40 within the 56 days prior to enrollment.

Laboratory Criteria within 56 days prior to enrollment:

10. Hemoglobin ≥ 11.0 g/dL for women; ≥12.5 g/dL for men.
11. White blood cells (WBC) = 2,500-12,000 cells/mm^3.

12. WBC differential either within institutional normal range or accompanied by the Principal Investigator (PI) or designee approval.

13. Total lymphocyte count ≥ 800 cells/mm^3.

14. Platelets = 125,000 – 400,000/mm^3.

15. Alanine aminotransferase (ALT) ≤ 1.25 x upper limit of normal.

16. Serum creatinine ≤ 1 x upper limit of normal.

17. Partial thromboplastin time (PTT) within institutional normal range.

18. Prothrombin time (PT) within institutional normal range.

19. HIV-uninfected as evidenced by a negative FDA-approved HIV diagnostic test.

Female-Specific Criteria:

20. Negative β-HCG (human chorionic gonadotropin) pregnancy test (urine or serum) on day of enrollment if woman is presumed to be of reproductive potential.

21. Agrees to use an effective means of birth control from at least 21 days prior to enrollment through 24 weeks after study vaccination if presumed to be of reproductive potential.

5.1.2. Exclusion Criteria for Part I

A volunteer will be excluded if one or more of the following conditions apply:

Volunteer has received any of the following substances:

1. Investigational Ebola or Marburg vaccine (other than the Ebola DNA vaccine delivered in RV 247) in a prior clinical trial or prior receipt of a cAd3 adenoviral vectored investigational vaccine.

2. Chronic use of immunomodulators and systemic glucocorticoids in daily doses of glucocorticoid equivalence > 20 mg of prednisolone, for periods exceeding 10 days. Non-steroidal anti-inflammatory drugs [NSAIDS] are permitted. Participants that have used less than the stated glucocorticoid dose may still be excluded at the Investigator's discretion.

3. Blood products within 112 days (16 weeks) prior to enrollment.

4. Investigational research agents within 28 days (4 weeks) prior to enrollment.

5. Live attenuated vaccines within 28 days (4 weeks) prior to enrollment.

6. Subunit or killed vaccines within 14 days (2 weeks) prior to enrollment.

7. Current anti-tuberculosis prophylaxis or therapy.

Female-specific criteria:

8. Woman who is breast-feeding or planning to become pregnant during the first 24 weeks after study vaccine administration.

Volunteer has a history of any of the following clinically significant conditions:

9. Serious adverse reactions to vaccines such as anaphylaxis, urticaria (hives), respiratory difficulty, angioedema, or abdominal pain.

10. Clinically significant autoimmune disease or immunodeficiency.

11. Asthma that is not well controlled.

12. Diabetes mellitus (type I or II), with the exception of gestational diabetes.

13. Thyroid disease that is not well controlled.

14. A history of hereditary angioedema (HAE), acquired angioedema (AAE), or idiopathic forms of angioedema.

15. Idiopathic urticaria within the last 1 year.

16. Hypertension that is not well controlled.

17. Bleeding disorder diagnosed by a doctor (e.g. factor deficiency, coagulopathy, or platelet disorder requiring special precautions) or significant bruising or bleeding difficulties with IM injections or blood draws.

18. Malignancy that is active or history of a malignancy that is likely to recur during the period of the study.

19. Seizure in the past 3 years or treatment for seizure disorder in the past 3 years.

20. Asplenia or functional asplenia.

21. Psychiatric condition that precludes compliance with the protocol; past or present psychoses; or within five years prior to enrollment, history of a suicide plan or attempt.

22. Any medical, psychiatric, social condition, occupational reason or other responsibility that, in the judgment of the investigator, is a contraindication to protocol participation or impairs a volunteer’s ability to give informed consent.

5.1.3. Criteria for Participation in Part II

Subjects who have received the VRC-EBOADC069-00-VP (cAd3-EBO) or the VRC-EBOADC076-00-VP (cAd3-EBOZ) vaccine and have completed at least 36 weeks of follow-up, must be assessed as eligible in order to participate in Part II. Eligibility assessments are based on knowledge of subject health and medical history.

The following inclusion criteria apply:

1. The subject is willing to participate.
2. Must complete an Assessment of Understanding (AoU) prior to enrollment by answering 9 out of 10 questions at least once in 3 attempts.
3. By clinical judgment of the site PI or designee, the subject is in good general health without clinically significant medical history that precludes study participation.

Female-Specific Criteria:

4. Negative β-HCG (human chorionic gonadotropin) pregnancy test (urine or serum) on day of enrollment if woman is presumed to be of reproductive potential.

5. Agrees to use an effective means of birth control from at least 21 days prior to enrollment through 24 weeks after study vaccination if presumed to be of reproductive potential.

The following exclusion criteria apply:

1. Type 1 hypersensitivity reaction to aminoglycoside antibiotics.

Subjects who meet the above criteria will complete "Visit 07" on Schedule 1 as well as the additional procedures shown for "Visit 07" on Schedule 2, which includes an ECG. The subject may be excluded from receiving the MVA-EbolaZ vaccine for ECG with clinically significant abnormalities (examples may include: pathologic Q waves, significant ST-T wave changes, left ventricular hypertrophy, any non-sinus rhythm excluding isolated premature atrial contractions, right or left bundle branch block, advanced A-V heart block). ECG abnormalities determined by a cardiologist to be clinically insignificant as related to study participation do not preclude receipt of MVA-EbolaZ.

If a subject is unable to receive the MVA-EbolaZ vaccine, the subject's study participation will be complete.

6. STUDY PROCEDURES

6.1. Schedule of Evaluations

Evaluation of the safety of this vaccine will include laboratory studies, medical history, physical assessment by clinicians, and subject self-assessment recorded on a 7-day diary card. Potential adverse reactions will be further evaluated prior to continuing the immunization schedule. Research assays for immune responses will be performed at the MUWRP laboratory and VRC associated research laboratory facilities. The schedule of evaluation is located in Appendix 1. Total blood volume drawn from each subject will not exceed 450 mL in any 12-week period. Investigators or designee(s) will conduct all study procedures. As per GCP, all designees will be delegated study procedures as per designee education, training and experience.

6.1.1. Recruitment

Participants for Group I will be recruited through IRB-approved recruitment materials (to include flyers, posters, newspaper ads and radio scripts). Volunteers will be invited into information sessions during which general vaccine and Ebola information will be provided. Potential participants interested in the study will be subsequently invited and scheduled to a briefing session. The briefing sessions will be conducted at the MUWRP facility at regular intervals throughout the recruitment phase.

As earlier described, study Group II will be composed of up to 30 participants who participated in Group 1 of Protocol RV 247/ MUSPH# 059 “A Phase Ib Study to Evaluate the Safety and Immunogenicity of an Ebola DNA Plasmid Vaccine, VRC-EBODNA023-00-VP, and a Marburg DNA Plasmid Vaccine, VRC-MARDNA025-00-VP, in Healthy Adults in Kampala, Uganda” conducted 2009-2012; and received VRC-EBODNA023-00-VP (Ebola DNA WT) or in combination with VRC-MARDNA025-00-VP (Marburg DNA). These participants will be recruited by employing their mobile phone numbers and other contacts (to include home visits where necessary) in the RV 247 study records. The site will request of the IRB to permit contacting these clients based on contact information from their participation in RV 247. Participants will be informed about the study and scheduled for an information and briefing sessions at their convenience. All participants interested in this study will be subsequently administered informed consent as described in Section 6.1.2 of the protocol.

6.1.2. Consent Procedures and Screening

<u>Part I:</u>
Study volunteers will receive an information and briefing session about vaccines from the Principal Investigator (PI) or designee during which the study will be explained and participation requirements outlined. The content of the information and briefing sessions is provided as shown in Appendix 4.

The briefing session will be followed by an opportunity for questions from the volunteers. A study team member will then review the consent form in detail with potential volunteers and answer any questions. After review with the study team member, an Informed Consent Form (Appendix 2) will be signed by all volunteers prior to enrollment in the study and an Assessment of Understanding (AoU) (Appendix 3) will be completed. The AoU is a study tool that enables study staff to discern comprehension of study information in the informed consent form and to assess specific topics to re-visit. The informed consent and AoU will be a “one-on-one” review with the volunteer by a member of the study team. To pass the AoU, the volunteer must answer 90% or 9 out of 10 of the questions

correctly. If the volunteer is unable to do so, he or she will be given 3 opportunities to repeat the AoU. If after 3 attempts to pass the AoU the volunteer is unable to do so, the volunteer will become ineligible for study participation.

Volunteers who have passed the AoU and have given written informed consent will undergo a complete medical history, physical examination, and screening laboratory assessments to determine eligibility for trial participation. Volunteers will be instructed to avoid blood donations for 52 weeks after receiving the study vaccine.The following screening assessments will be completed after the informed consent process has been completed:

- Medical history
- Physical exam
- Vital signs and weight
- Lymph node assessment
- Pregnancy test for all females
- CBC with differential
- Creatinine
- ALT
- HIV
- Peripheral blood mononuclear cells (PBMC) and plasma for storage
- PT
- PTT

General eligibility for clinical trials will be dependent on results of laboratory tests and answers to the interview questions.

Counseling related to the potential risks of becoming pregnant during this trial will be provided. Pre-HIV test counseling and post-HIV test counseling will be provided during the screening process.

Study volunteers may be scheduled for a second Screening Visit to evaluate laboratory and test results if an abnormality or illness had been identified at the first screening. This approach will allow study staff to evaluate the recovery from any illness identified at the first screening as well as to repeat screening laboratory tests that were abnormal at the first screen due to transient illness. The PI or Investigator at the site may discover an illness or condition, which requires treatment for the volunteer. The site will provide basic care and treatment as per National guidelines and refer to a hospital or clinic, which can provide further evaluation and treatment. Where/when requisite, screened participants may be referred to Mulago National Referral Hospital or referred to a medical facility of their choice.

Eligible and willing volunteers will be scheduled for an appointment for enrollment and the initial vaccination visit within 56 days of having satisfied eligibility requirements.

Part II:
Assessment of eligibility for subjects who enroll into Part II of the study will be performed through evaluations conducted during participation on RV 422 PART I & PART II. Clinical judgment will be used for eligibility evaluation of these subjects, and each case will be approved by the site PI or designee. A baseline ECG will be performed to assess for eligibility to receive the MVA-EbolaZ study vaccine. If the subject receives the MVA-EbolaZ study vaccine, the subject will continue on study following Schedule of Evaluations 2. If the subject does not receive the MVA-EbolaZ study vaccine for any reason, study will conclude after completion of Visit 07.

6.1.3. Day 0 through Week 48

Day 0 is defined as the day of enrollment and study injection. Study eligibility criteria are reviewed on Day 0 as part of the enrollment process. However, if clinical assessment on Day 0 suggests significant changes may have occurred since the screening visit, then the physical examination, hematology tests, blood chemistries, and urinalysis done at screening must repeated for determination for eligibility. Pregnancy test results for women of childbearing potential must be obtained on each injection day prior to the study injection. Day 0 evaluations immediately prior to the first injection are the baseline for subsequent safety assessments.

Refer to the table in Appendix 1 for details on the Schedule of Evaluations and the window permitted for completion. After Day 0, deviations from the visit windows in completing study visits are discouraged and will be recorded as protocol deviations, but are permitted, at the discretion of the PI (or designee) in the interest of obtaining subject safety and immunogenicity evaluations following exposure to the investigational vaccines.

Study visit procedures and tests through Week 48 include the following as indicated in the Schedule of Evaluations (Appendix 1):

1. Signature of study participation informed consent form for RV 422 PART I & PART II.
2. Assessment of Understanding (prior to enrollment).
3. Clinical evaluations: vital signs; axillary lymph node examination (every visit); targeted physical examination on any visit if indicated by interim complaints or laboratory findings.
4. Interim medical history.
5. Pre- and Post HIV test counseling.
6. Counseling on avoidance of pregnancy.
7. Post-injection blood pressure, pulse and assessment of injection site at 30 to 60 minutes post-injection (target 30 to 45 minutes, when possible).
8. Diary Card (Appendix 8): Baseline on day of injection; 7-day diary card for self-assessment by subject following each injection.
9. Serum or urine pregnancy test, for females of reproductive potential.
10. CBC, differential, platelet counts.
11. Blood creatinine and ALT.
12. PT and PTT.
13. HLA Type.
14. Serum, PBMCs, and plasma for archiving.

Stored samples with consent for genetic testing may be used later to elucidate genetic factors associated with immune response to a vaccine and to further evaluate responses to the vaccine. Any cells, serum, or plasma not used will be stored for future exploratory virological and immunological assays. All future-use research will be reviewed and approved by a local IRB and the UNCST notified of those studies conducted abroad. Future use studies conducted within the country will be reviewed and approved by the local IRB and by the UNCST.

6.2. Administration of the Study Injection

cAd3-EBO and cAd3-EBOZ injections will be administered IM in a 1 mL volume by needle and syringe. MVA-EbolaZ injections will be administered IM in a 0.3 mL volume by needle and syringe. It is recommended, but not required, that the injection be administered into the non-dominant arm. When choosing an arm for the injection, clinicians should consider whether there is an arm injury, local skin problem or significant tattoo that precludes administering the injection or will interfere with evaluating the arm after injection.

Following the study injection, subjects will be observed for a minimum of 30 minutes. Vital signs (temperature, blood pressure, pulse, and respiratory rate) will be taken 30 to 60 minutes post-injection. The injection site will be inspected for evidence of local reaction. In keeping with good medical practice, acute medical care will be provided to subjects for any immediate allergic reactions or other injury resulting from participation in this research study.

6.2.1. 7-Day Solicited Reactogenicity

Temperature and solicited systemic symptoms will be recorded in the clinic prior to vaccination and at a minimum of 30 minutes post procedure and then daily by the participant for 7 days.

Subjects will be given a "Diary Card" to use as a memory aid, on which to record temperature, local and systemic symptoms and concomitant medications daily for 7 days after each injection. Subjects will be trained to complete the paper diary card. The written (paper) diary card may be used as a source document.

The solicited signs and symptoms on the diary card (among others) will include: unusually tired/feeling unwell, muscles aches (other than at injection site), headache, chills, nausea, and pain/tenderness at injection site. Subjects will also record the day's highest measured temperature and measurement of largest diameters for redness and swelling at the injection site. The completed diary cards are collected at the first visit following completion of the 7-day diary card (about 2 weeks after the injection).

Follow-up on subjects' well-being will be performed by clinic visit on Day 3 following each injection to assess their clinical status. Volunteers may call or come to the clinic at any time-point for evaluation, if they experience unusual, moderate or severe signs and symptoms. Diary cards will be reviewed with the clinician at any visits from day of vaccination through the first study visit following completion of the diary card.

Events following injection that may require clinical evaluation include rash, urticaria, fever of 38.5˚C (Grade 2) or higher lasting greater than 24 hours, or significant impairment in the activities of daily living (ADL). Fever of any severity or any condition which in the judgment of the clinician should be evaluated would require a clinical visit.

At every visit all participants will be asked about other adverse experiences, which will be recorded and entered the same day into the study database. The PI will assess the relationship of the study products to the events.

6.2.2. Management of Reactogenicity Adverse Events Following Study Vaccinations

Participants will be informed that a rash, urticaria, fever of 38.5˚C (Grade 2) or higher lasting greater than 24 hours, or significant impairment in the activities of daily living (ADL) should prompt a visit to the clinic. Fever less than 38.5˚C or lasting less than 24 hours may prompt a clinic visit at the discretion of the study clinicians. Any condition which in the judgment of the clinician should be evaluated clinically or by laboratory testing would require a clinical visit.

6.2.3. Management of laboratory abnormalities after vaccination through Study Week 4:

If any lab result is a Grade 3 or Grade 4, then repeat testing of the abnormal test will be performed within 48 hours. The specific timing of repeat testing for a Grade 2 laboratory test change will be determined by the study clinicians as medically appropriate.

Clinical evidence of coagulopathy, will prompt targeted physical exam including a blood smear review by hematologist for evidence of microangiopathic hemolysis. Other evaluations may be performed as would be indicated according to standard clinical practice for the site.

6.3. Concomitant Medications

Current concomitant medications are recorded in the study database at enrollment. Medications taken during the 7-day period following vaccination will be documented. Subsequently, concomitant medications will be updated in the study database if there is an occurrence of an adverse event that requires expedited reporting or development of a new chronic medical condition that requires ongoing medical management.

Clinicians should work with study subjects with regard to the timing of FDA or locally-approved vaccines. Receipt of a licensed vaccine during study participation will be recorded in the study database. Otherwise, a record of other concomitant medication changes throughout the study will not be recorded in the study database.

6.4. Study Discontinuation

6.4.1. Early Discontinuation or Withdrawal of Study Participants

A participant will be taken out of the study entirely under the following circumstances:

- Repeated failure to comply with protocol requirements.
- Decision by the study sponsor or the Protocol Chair to stop or cancel the study.
- Decision by Walter Reed Army Institute of Research (WRAIR) and the United States Army Medical Research and Materiel Command's (USAMRMC) Office of Research Protections (ORP), Human Research Protection Office (HRPO), NIAID, the U.S. FDA, the Office for Human Research Protection (OHRP), or local regulatory authorities and IRBs to stop or cancel the study.
- Participant requests withdrawal

Each subject has the right to withdraw from the study at any time for any reason without affecting the right to treatment by the investigator. The investigator should make an attempt to contact subjects who did not return for scheduled visits or follow-up. Although the subject is not obliged to give reason(s) for withdrawing prematurely, the investigator should make a reasonable effort to ascertain the reason(s) while fully respecting the subject's rights.

For those subjects who are unable to continue participation in the study, but who do not withdraw consent, an exit visit will be conducted (refer to SOE for list of procedures to be conducted). Any subject who withdraws consent will not have any further data collected after consent has been withdrawn. However, data that was collected prior to a subject's withdrawal of consent will be included in data analysis.

The investigator also has the right to withdraw a subject, e.g., because of worsening health status, intercurrent illness, or other event, which makes further follow-up impossible.

If a subject withdraws from the study, the next available participant will be placed in the vacated randomization slot. Replacements for participants who withdraw from the study will only be permitted while enrollment is still open.

6.4.2. Management of Volunteers Who Become Pregnant

Pregnant women are excluded from enrollment. The IND Sponsor requirement is to record in the study database the outcome of pregnancies that begin within 24 weeks after a vaccination. Reporting outcomes of pregnancies that begin later than this is not required by the IND Sponsor, nor is there an IND Sponsor requirement for annual evaluation requirement for children born to vaccine recipients.

However, the site is responsible for maintaining records that may be required by other regulatory authorities. In this regard, if a volunteer becomes pregnant during the course of the study, volunteers will continued to be followed for the remainder of the study period for safety and the study team will follow the course of the pregnancy until the pregnancy outcome is documented. During subsequent study visits, blood draws will be conducted for safety purposes only. No optional collections or procedures will be performed.

The site PI or designated associate investigator will be responsible for reporting any pregnancy. This information will be reviewed weekly in aggregate with other safety data and forward as necessary to IRBs, study sponsors, and regulatory agencies.

Pregnancy outcomes will be recorded via a standardized case report form. Information documented on this form will include date of last menstrual period, date pregnancy confirmed, history of complications during prior pregnancies (such as congenital abnormalities or spontaneous abortions), and the outcome of the pregnancy including date of termination or delivery, any complications of pregnancy, and the status of the child. A separate case report form will be completed for the delivered child to document date of delivery, gender, weight, presence of any congenital abnormalities, APGAR score, and any other complication of delivery.

All children born to vaccine recipients will be annually evaluated (as above) for a minimum of 5 years to meet requirements set by regulatory authorities in Uganda.

7. STUDY TREATMENT

Study treatment is defined as VRC-EBOADC076-00-VP (cAd3-EBOZ), VRC-EBOADC069-00-VP (cAd3-EBO), and VRC-EBOMVA079-00-VP (MVA-EbolaZ).

7.1. Regimens

Ninety healthy adults subjects in the Kampala area ages 18-65 will be randomized.

Part I:
Group 1: Sixty Ebola vaccine naïve subjects will be randomized to receive a single injection of vaccine as follows:

a) 15 subjects will receive cAd3-EBOZ at $1x10^{10}$ PU
b) 15 subjects will receive cAd3-EBOZ at $1x10^{11}$ PU
c) 15 subjects will receive cAd3-EBO at $2x10^{10}$ PU
d) 15 subjects will receive cAd3-EBO at $2x10^{11}$ PU

Group 2: Up to 30 eligible subjects who previously participated in the RV 247 vaccine clinical trial and received VRC-EBODNA023-00-VP (Ebola DNA WT) will be randomized to receive a single injection of vaccine as follows:

a) 15 subjects will receive cAd3-EBO at $2x10^{10}$ PU
b) 15 subjects will receive cAd3-EBO at $2x10^{11}$ PU

Part II:

All subjects who chose to participate in Part II and who meet eligibility requirements will receive a single injection of MVA-EbolaZ at 1 x 10^8 PFU.

Table 5. RV 422 PART I & PART II Study Design

	RV 422 PART I & PART II Study Schema			
**Group**	**Sub-Group**	**Subjects**	**Part I: Day 0**	**Part II: Week 36+**
1		60		
	1a	15	cAd3-EBOZ at $1x10^{10}$ PU IM	MVA-EbolaZ at $1x10^8$ PFU IM
	1b	15	cAd3-EBOZ at $1x10^{11}$ PU IM	MVA-EbolaZ at $1x10^8$ PFU IM
	1c	15	cAd3-EBO at $2x10^{10}$ PU IM	MVA-EbolaZ at $1x10^8$ PFU IM
	1d	15	cAd3-EBO at $2x10^{11}$ PU IM	MVA-EbolaZ at $1x10^8$ PFU IM
2		30*		
	2a	15	cAd3-EBO at $2x10^{10}$ PU IM	MVA-EbolaZ at $1x10^8$ PFU IM
	2b	15	cAd3-EBO at $2x10^{11}$ PU IM	MVA-EbolaZ at $1x10^8$ PFU IM
**Total**		**90**		

Groups 1 and 2 will be enrolled simultaneously
cAd3-EBO and cAd3-EBOZ are administered in 1 mL volume with needle and syringe. MVA-EbolaZ is administered in 0.3 mL volume with needle and syringe.
If a subject enrolls, but is not vaccinated, another subject may be enrolled to achieve the target number of administered vaccinations.
If less than 30 subjects are enrolled into Group 2, the remaining enrollment slots may be allocated to additional randomization slots in Group 1.
In Part II, the interval time to receive the boost may vary but will be at least 36 weeks.

7.2. Administration

VRC-EBOADC069-00-VP, VRC-EBOADC076-00-VP and VRC-EBOMVA079-00-VP will be administered intramuscularly by needle and syringe.

7.2.1. Duration

Part I: Participants will be followed for up to 48 weeks in Part I of the study.

Part II: Participants will be followed for 48 weeks after beginning Part II of the study.

7.3. Study Product Formulation, Preparation, and Storage

The study groups are shown in the Study Schema above. Refer to the Investigator's Brochure for further information about the study agents.

7.3.1. Formulation

The drug product, VRC-EBOADC076-00-VP (cAd3-EBOZ) was prepared at the VRC Pilot Plant (VPP) operated under contract by Vaccine Clinical Materials Program (VCMP), Leidos Biomedical Research Inc., Frederick, MD. The drug product is a sterile, aqueous, buffered solution composed of cAd3 EBO Z drug substance filled into single dose vials at 1 x 10^{11} PU/mL and is supplied in a 3 mL glass vial with 0.7 mL fill volume. Vials are aseptically filled under cGMP to a volume of 0.7 mL to allow withdrawal of 0.5 mL. Vials are intended for single use only and thus do not contain a preservative.

The drug product, VRC-EBOADC069-00-VP (cAd3-EBO), was prepared at the VRC Pilot Plant (VPP). It is formulated as a 1:1 ratio of cAd3 EBO Z and cAd3 EBO S drug substances. Final product meeting all test specifications will be released for use in the clinical study. The vaccine is manufactured at a $2x10^{11}$ PU/mL dose in formulation buffer. Vials are aseptically filled under cGMP to a volume of >1 mL to allow withdrawal of 1 mL. Vials are intended for single use only and thus do not contain a preservative.

The drug product, VRC-EBOMVA079-00-VP (MVA-EbolaZ), was prepared at the VRC Pilot Plant (VPP). The drug product is a sterile, clear to opaque to slightly brown homogeneous suspension compound of MVA-GP Ebola Z drug substance filled into single dose vials at 3.2 x 10^{8} PFU/mL. The buffer is 1.21 mg/mL Tris (hydroxymethyl)-amino methane and 8.18 mg/mL sodium chloride with trace amounts of gentamicin (antibiotic) and sucrose. Vials are aseptically filled to a volume of 0.7 mL.

The diluent, VRC-DILADC065-00-VP, was prepared by the VPP. It is comprised of the formulation buffer and will be used to dilute the cAd3-EBO vaccine to prepare the lower dosage to be administered in this study. The formulation buffer is pH 7.4 and consists of 10 mM Tris, 10 mM Histidine, 5% w/v sucrose, 75 mM NaCl, 1 mM MgCl2, 0.02% Polysorbate-80, 0.1 mM EDTA, and 0.5% (v/v) Ethanol. The diluent is a sterile, buffered, aqueous solution filled into glass vials at a fill volume > 1 mL to allow withdrawal of 1 mL.

7.3.2. Study Agent Labels

The labels for VRC-EBOADC076-00-VP (cAd3-EBOZ), VRC-EBOADC069-00-VP (cAd3-EBO vaccine), VRC-EBOMVA079-00-VP (MVA-EbolaZ) and VRC-DILADC065-00-VP, diluent, will have specific product information (e.g., part number, lot number, fill volume, storage temperature)

included on the product vial labels. The label will contain an Investigational Use Statement (“Caution: Limited by Federal Law to Investigational Use”), and manufacturer information.

7.3.3. Preparation

To prepare the vaccine for injection thaw the vial containing cAd3- or MVA- at ambient temperature. Material should be kept at ambient temperature during the preparation procedure until injection. Prepared injections should be kept out of direct sunlight lying horizontal for not longer than 4 hours from beginning the thaw to administration.

Preparation should be done in a temperature controlled preparation unit on a clean table with limited access using aseptic technique. Assure that only the required vials are present in the preparation unit during reconstitution, and medication labels are strictly segregated to avoid mix-ups.

All injections will be administered IM into the deltoid muscle by needle and syringe and must be administered within 4 hours after removing the vaccine vial from the freezer.

Preparation of VRC-EBOADC076-00-VP (cAd3 EBOZ) at 1 x 10^{10} PU Dose

Preparation of the 1 x 10^{10} PU dosage of cAd3-EBOZ requires one serial dilution. Remove one vial of cAd3-EBOZ vaccine and two vials of diluent from the freezer and allow them to equilibrate to room temperature. Using 1 mL sterile syringes, draw up 1 mL of diluent from one diluent vial and 0.35 mL from the other diluent vial and inject these volumes into a 10 mL sterile vial. Then, using a 1 mL sterile syringe draw up 0.15 mL of the 1 x 10^{11} PU/mL cAd3-EBOZ vaccine and inject this into the 10 mL vial with the diluent to achieve a total volume of 1.5 mL. Vortex the vial at half speed for 3-5 seconds. This vial now contains 1.5 x 10^{10} PU VRC-EBOADC076-00-VP in 1.5 mL and has a concentration of 1 x 10^{10} PU/mL. One 1 mL injection of this preparation will be administered for each dose. Discard remnant vaccine in vial in a biohazard container for incineration.

Preparation of VRC-EBOADC069-00-VP (cAd3-EBO) at 2 x 10^{10} PU dose.

Preparation of the 2 x 10^{10} PU dosage of cAd3-EBO requires one serial dilution. Remove one vial of cAd3-EBO vaccine and two vials of diluent from the freezer and allow them to equilibrate to room temperature. Using 1 mL sterile syringes, draw up 1 mL of diluent from one diluent vial and 0.35 mL from the other diluent vial and inject these volumes into a 10 mL sterile vial. Then, using a 1 mL sterile syringe draw up 0.15 mL of the 2 x 10^{11} PU/mL vaccine and inject this into the 10 mL vial with the diluent to achieve a total volume of 1.5 mL. Vortex the vial at half speed for 3-5 seconds. This vial now contains 3 x 10^{10} PU VRC-EBOADC069-00-VP in 1.5 mL and has a concentration of 2 x 10^{10} PU/mL. One 1 mL injection of this preparation will be administered for each dose. Discard remnant vaccine in vial in a biohazard container for incineration.

Preparation of VRC-EBOADC076-00-VP (cAd3 EBOZ) at 1 x 10^{11} PU dose.

No dilution is needed for preparation of VRC-EBOADC076-00-VP **(**cAd3-EBOZ) vaccine but two vials are needed for each dose as there is only 0.7 mL volume in the vial. Remove two vials of vaccine from the freezer and allow them to equilibrate to room temperature. Withdraw at least 0.5 mL from each vial One 1 mL injection of the preparation will be administered for each dose. Discard remnant vaccine in vials in a biohazard container for incineration.

Preparation of VRC-EBOADC069-00-VP (cAd3-EBO) at 2 x 10^{11} PU dose

No dilution is needed for preparation of VRC-EBOADC069-00-VP (cAd3-EBO) vaccine and only one vial is needed for each dose as there is >1 mL volume in the vial. Remove one vial of vaccine from the freezer and allow it to equilibrate to room temperature. One 1 mL injection of the preparation will be administered for each dose. Discard remnant vaccine in vials in a biohazard container for incineration.

Preparation of VRC-EBOMVA079-00-VP (MVA-EbolaZ) at 1 x 10^8 PFU dose

No dilution is needed for preparation of a 1x10^8 PFU dose of VRC-EBOMVA079-00-VP (MVA-EbolaZ). Remove a vial of vaccine from the freezer and allow it to equilibrate to room temperature. Withdraw at least 0.3 mL from the vial to prepare a syringe for administration of a 1x10^8 PFU dose in 0.3 mL volume. One 0.3 mL injection of the preparation will be administered IM into the deltoid muscle for each 1x10^8 PFU dose within 4 hours after removing the vaccine vials from the freezer. Discard remnant vaccine in vials in a biohazard container for incineration.

7.3.4. Storage

VRC-EBOADC076-00-VP (cAd3 EBOZ), VRC-EBOADC069-00-VP (cAd3-EBO) and VRC-EBOMVA079-00-VP (MVA-EbolaZ) will be stored at ≤-60°C and are intended for intramuscular administration.

VRC-DILADC065-00-VP (diluent) will have clinical storage in a qualified, continuously monitored, temperature-controlled freezer at -45°C to -10°C is expected.

If deviations in storage temperature occur from the normal allowance for the pharmacy freezer, the site pharmacist must report the storage temperature excursion promptly to the PI and the IND sponsor. The excursion must be evaluated and investigated and action must be taken to restore and maintain the desired temperature limits. Pending the outcome of the investigation, the IND sponsor will notify the pharmacist if continued clinical use of the product is acceptable. The site pharmacist must promptly report any storage temperature excursions outside of the normal allowance for the storage device to the IND sponsor’s authorized representative. The product must be quarantined at the appropriate temperature in a designated area. The IND Sponsor’s authorized representative will notify the site pharmacist if continued clinical use of the product is acceptable.

7.4. Pharmacy: Product Supply, Distribution, and Accountability

7.4.1. Study Product Acquisition/Distribution

The Study product (s) for this protocol are supplied by the Vaccine Research Center, NIH and should be obtained by following the instructions in the Pharmacy manual for further information. Study Product Accountability

The study pharmacist will be responsible for maintaining an accurate record of the codes, inventory, and an accountability record of vaccine supplies for this study. Electronic documentation as well as paper copies may be used.

The VRC will receive copies of pharmacy records at the end of the study.

7.4.2. Disposal of Clinical Supplies

The empty vials and the unused portion of the vial should be discarded in a biohazard containment bag and incinerated. Any unopened vials that remain will be discarded at the discretion of the sponsor in accordance with policies that apply to investigational agents. Partially used vials will not be administered to other subjects or used for *in vitro* experimental studies. They will be disposed of in accordance with institutional or pharmacy policy.

8. PHARMACOVIGILENCE, SAFETY, AND ADVERSE EXPERIENCE REPORTING

8.1. Definitions

An adverse event (AE) is any untoward medical occurrence in a patient or clinical investigation subject administered a pharmaceutical product and does not necessarily have a causal relationship with this product. An AE can therefore be any unfavorable and unintended sign (including an abnormal laboratory finding), symptom, or disease temporally associated with the use of a medicinal (investigational) product, whether or not related to the medicinal (investigational) product. (International Conference on Harmonization (ICH) E6) (Synonym: Adverse Experience).

A serious adverse event (SAE): An adverse event or suspected adverse reaction is considered "serious" if, in the view of either the investigator or sponsor, it results in any of the following outcomes: Death, a life-threatening adverse event, inpatient hospitalization or prolongation of existing hospitalization, a persistent or significant incapacity or substantial disruption of the ability to conduct normal life functions, or a congenital anomaly/birth defect. Important medical events that may not result in death, be life-threatening, or require hospitalization may be considered serious when, based upon appropriate medical judgment, they may jeopardize the patient or subject and may require medical or surgical intervention to prevent one of the outcomes listed in this definition. Examples of such medical events include allergic bronchospasm requiring intensive treatment in an emergency room or at home, blood dyscrasias or convulsions that do not result in inpatient hospitalization, or the development of drug dependency or drug abuse (21 CFR 312.32).

“Life-threatening” refers to an adverse event that at occurrence represents an immediate risk of death to the subject. An event that may cause death if it occurs in a more severe form is not considered life-threatening. Similarly, a hospital admission for an elective procedure is not considered an SAE.

All AEs will have their possible relationship to study vaccine assessed. The definitions and categories to use for attribution assessment can be found in Appendix V.

8.2. Adverse Event Grading and Recording

Recording of all AEs will occur during the period from study agent administration through 28 days after each study agent administration. At other intervals through completion of study participation, only SAEs and new chronic medical conditions that require ongoing medical management will be recorded as AEs in the study database. Because this is an Ebola vaccine study, in the unlikely circumstance of Ebola virus disease (EVD) diagnosis in a study subject at any time throughout the study, this will be recorded on an “EVD case report form”, rather than an AE form, without requiring an investigator attribution (“relatedness to study agent”) or severity grade.

The FDA Guidance for Industry (September 2007): “Toxicity Grading Scale for Healthy Adult and Adolescent Volunteers Enrolled in Preventive Vaccine Clinical Trials” was adapted for the severity grading of adverse events in this protocol. The FDA Guidance of Industry on toxicity grading scale is modified for criteria on absolute neutrophil counts and other parameters as described in Appendix 5, which also includes the definitions for severity grading parameters that do not have specific guidelines in the table. The clinical research team will ascertain accurate recording of AEs during the study. AE case report forms (CRFs) will be completed by the research staff on a daily basis as the data become available from the clinic or laboratory.

The clinical investigators will monitor and analyze study data including all AE and lab data as they become available and will make determinations regarding the severity of the adverse experiences and their relation to study product. To insure that all AEs are captured in a timely manner, CRFs will be entered in real-time and also subjected to analysis to identify AEs that may invoke study pause rules.

Although post injection reactogenicity (PIR)/Solicited AEs are documented separately from unsolicited AEs, they are reported if they meet SAE or study pause rule definitions as noted below. Therefore the PI or designee must review both PIR and AE CRFs to insure prompt and complete identification of all events that require expedited reporting as SAEs, study pause rules or other serious and unexpected events.

AEs will be followed by the clinical research team until resolved, stable or chronicity has been established and documented.

8.3. Protocol Safety Review Team (PSRT) and PSRT Reviews

The PSRT will review all AEs (including reportable AEs) on a regular and expedited basis as needed. In addition, the PSRT will review aggregate safety data reports on a weekly basis until 4 weeks after all subjects have completed the Schedule 1 vaccination, then the safety reviews will occur monthly. Reviews will again be weekly until all subjects participating in Schedule 2 complete the booster vaccination and then the safety reviews will occur monthly. This team includes the following: Principal Investigator or designee, Protocol Chair or designee, Protocol Clinical Coordinator, Protocol Consultants, Local Medical Monitor and RV 422 PART I & PART II VRC Representatives. Additional participants could include associate investigators, and senior clinical research nursing staff. A quorum is established with the Protocol Chair, site PI and RV 422 PART I & PART II VRC Representative or their designees.

8.4. Safety Monitoring Committee (SMC) Reviews

The SMC for this study will be comprised of an independent group of experts to review safety data during the clinical trial. The SMC will review and approve of the protocol prior to study initiation. The Protocol Chair (or designee) will submit written recommendations and cumulative safety data, grouped according to treatment group, to the SMC Executive Secretary after the first enrollee has completed 90 days of the study. Subsequently, the SMC will convene every six months to review the completeness of the study data collected, the adherence to the protocol, and the Protocol Chair’s review summaries. The SMC will also meet as needed to deliberate upon the initial interim safety data, the disposition of study pauses, and/or to provide other recommendations regarding the safe conduct of the study as requested by the Protocol Chair. The SMC Executive Secretary will provide the Protocol Chair with SMC recommendations, and the Protocol Chair and PI will inform IRBs and regulatory authorities as appropriate. The VRC will notify the FDA of the SMC recommendation as needed.

8.5. Criteria for Study Pause or Termination

The Principal Investigator will closely monitor and analyze study data as they become available and will make determinations regarding the presence and severity of adverse events. The administration of study injections and new enrollments will be paused and the IND Sponsor will be promptly notified according to the following criteria:

- **One** (or more) subject experiences a **SAE or grade 4** adverse event that is assessed as probably or definitely related to study agent, (including “verified”* abnormal laboratory values, local or systemic PIR, or other study treatment related AEs (if fever, it must persist

for ≥48 hours) or

- **Two** (or more) subjects experience the same **Grade 3** adverse event assessed as probably or definitely related to study agent. To include "verified"* abnormal laboratory values, fever for ≥48 hours, vomiting, erythema, induration or other clinical study treatment related AEs (Except subjective events).

*If no evidence of disease is present other than the abnormal laboratory value, the test must be repeated at least once in order to be considered "verified". The verification period will be a maximum of 48 hours after initial awareness of the abnormal laboratory value. When signs and symptoms are present, repeat test WILL NOT be needed.

These pause rules will not apply to expected adverse events of fever, neutropenia or prolonged PTT unless there are clinical signs and symptoms judged to be a Serious Adverse Event.

Plan for Review of Pauses and Resuming Rules:

The study injections and enrollments would resume only if review of the adverse events that caused the pause resulted in a recommendation to permit further study injections and study enrollments. The reviews to make this decision will occur as follows:

Pauses for SAEs: The IND Sponsor, with participation by the Principal Investigator, will consult with the FDA to conduct the review and make the decision to resume or close the study for any SAEs that meet the criteria for pausing the study.

Pauses for Grade 3 Events: The IND Sponsor, in consultation with the Principal Investigator, will conduct the review and make the decision to resume or close the study for the Grade 3 events that meet the criteria for pausing the study. As part of the pause review, the reviewers will also advise on whether the study needs to be paused again for any subsequent Grade 3 event of the same type. The FDA will be notified of Grade 3 pause reviews.

When indicated, safety data reports and changes in study status are submitted to the IRB.

8.6. Reporting Requirements to the Local IRB and Regulatory Bodies

The site PI will be responsible for providing all Safety Reports and reporting all SAEs, study pauses, social harms, UPIRTSOs, and major deviations to the local regulatory authority, such as a local IRB, and any country-specific regulatory agencies, in a timely manner according to the institution's and national guidelines.

8.7. Reporting Serious and Unexpected Adverse Events

8.7.1. Study Recording Period for SAEs

The protocol-defined expedited event reporting period for this protocol is 48 weeks after study agent administration until study completion or discontinuation of the subject from study participation for any reason. After the end of the protocol-defined EAE Reporting Period stated above, sites must report serious, unexpected, suspected adverse drug reactions (SUSARs) if the study site staff becomes aware of the event from a participant on a passive basis (i.e., from publicly available information).

8.7.2. Reporting Serious and Unexpected Adverse Events to the Local Regulatory Bodies & Institutional Review Boards

AE reporting requirements to the Ugandan National Council For Science and Technology (UNCST), the Makerere University School of Public Health Institutional Review Committee (MUSPH IRB) and National Drug Authority (NDA) for this protocol are as follows:

All SAEs regardless of relationship to the intervention and all unexpected events (whose nature, severity or frequency had not been identified in the Investigator’s Brochure or the protocol) of greater than moderate severity regardless of relationship to the intervention, to include SUSAR (Serious, Unexpected, Suspected Adverse Drug Reactions) must be reported to the UNCST, MUSPH IRB and the NDA as soon as possible and in any case no later than 7 calendar days of becoming aware of the event. Thereafter, a detailed report of the SAE should be submitted within 7 days.

All other reportable adverse events should be reported to the IRB as soon as possible and in any case no later than 14 calendar days. These include:

a. All events associated with protocol violations regardless of severity and relationship to the intervention;

b. When criteria for stopping or pausing a study is stipulated in the protocol are met;

c. Any event mandated by regulatory authorities;

d. Any event stipulated in the protocol as reportable to the regulatory bodies.

8.8. Reporting Serious and Unexpected Adverse Events to WRAIR HSPB and MRMC

The WRAIR provides DoD-required human subjects protection review of clinical trials involving WRAIR investigators and employees. It is the responsibility of the MHRP COO to fulfill the reporting requirements. Additionally, telephonic review must be followed by a written report within 24 hours to the Local Medical Research Monitor and forwarded to the WRAIR Human Subjects Protection Branch (HSPB).

All AEs that are serious and unexpected and related to the product are reported as described above and become IND Safety reports. Any serious and unexpected adverse events assessed as related to the study agent will be reported to the WRAIR HSPB immediately to the Director of HSPB (UWZ-C) (301-319-9940). WRAIR HSPB will report SAEs to USAMRMC ORP HRPO as per SOP UWZ-C-636.

For IND Safety Reports submitted to the FDA and received from VRC, the MHRP COO will complete the necessary reporting to the Walter Reed Army Institute of Research (WRAIR), including telephone contact and a written report generated within 24 hours of the initial safety report. WRAIR HSPB will forward to USAMRMC ORP HRPO.

The COO will also submit the necessary AE reporting documents and any study pauses to the WRAIR and the USAMRMC ORP HRPO.

8.9. Unanticipated Problems Reporting

All unanticipated problems related to the study and involving risk to subjects or others (UPIRTSOs) and all subject deaths should be promptly reported to the WRAIR HSPB (phone +1 301-319-9940, facsimile +1 301-319-9961) by the MHRP COO and to MUSPH, UNCST by the PI/ designee. A complete written report should follow the initial notification. The complete report will be sent to the Director, Human Subjects Protection HSPB, Walter Reed Army Institute of Research, 503 Robert Grant Ave., Silver Spring, MD 20910-7500, and the site PI will submit a complete written report to the MUSPH IRB and UNCST within 7 days of knowledge of the event.

The local medical monitor should also review the unanticipated events and provide an independent assessment for the IND sponsor, MUSPH IRB, UNCST and WRAIR HSPB (as a local medical research monitor’s report). WRAIR HSPB will report these summaries to USAMRMC ORP HRPO. Unanticipated problems related to the Investigational product will be promptly reported to the NDA.

8.10. Adverse Event Reporting to the IND Sponsor

Adverse events that meet SAE Reporting Requirements must be reported and submitted by the clinical site on an expedited basis to the IND sponsor, VRC/NIAID/NIH, according to sponsor guidelines as follows:

- results in death
- is life threatening
- results in persistent or significant disability/incapacity
- requires unplanned inpatient hospitalization or prolongation of existing hospitalization
- is a congenital anomaly/birth defect in the offspring of a study subject
- is an important medical event that may jeopardize the subject or may require intervention to prevent one of the other outcomes listed above

In addition, any event, regardless of severity, which in the judgment of an investigator represents a serious adverse event, may be reported on an expedited basis.

An investigator will communicate the initial SAE report within 24 hours of site awareness of occurrence to the IND sponsor.

A written report by investigator should be submitted to the IND Sponsor within 3 working days. In order for the IND Sponsor to comply with regulations mandating sponsor notification of specified SAEs to the FDA within 7 or 15 calendar days, the investigator must submit additional information as soon as it is available.

8.10.1. IND Sponsor Reporting to the FDA

The IND Sponsor is responsible for making the determination of which SAEs are suspected unexpected serious adverse reactions (SUSARs) that meet criteria for expedited reporting as defined in 21 CFR 312.32.

- *Suspected adverse reaction* means any adverse event for which there is a reasonable possibility that the drug caused the adverse event.
- *Unexpected Adverse Event* means an AE that is not listed in the Investigator's Brochure or is not listed at the specificity or severity that has been observed.

The IND Sponsor will also submit an IND Annual Report of the progress of the investigation to the FDA as defined in 21 CFR 312.33.

The IND Sponsor is responsible for providing copies of IND Safety Reports to the Clinical Site for submission to the IRB.

9. STATISTICAL CONSIDERATIONS

The primary objective of this trial is to assess the safety and tolerability of the described Ebola vaccines. A total of 90 subjects will be enrolled to receive one of two vaccines with 30 subjects receiving the cAd3-EBOZ and 60 subjects receiving cAd3-EBO. Enrollment to the latter is further stratified on Ebola vaccination history with Ebola vaccine naive subjects randomized 1:1:1:1 to Groups 1a, 1b, 1c and 1d and subjects with a prior Ebola vaccination randomized 1:1 between Groups 2a and 2b. As this is an open label study, clinic staff will be aware of treatment assignment, but will not be aware of future randomization assignments (except for unblinded staff performing the randomization).

To assess safety, the proportion of subjects experiencing a safety event can be described both by dose, vaccine and vaccination history as well as collapsed by various factors. Table 6 shows exact Clopper-Pearson 95% confidence intervals (CIs) for observed rates based on the number of subjects.

Table 6. Exact 95% Confidence Intervals for the true rate of events for selected sample sizes and number of observed events.

(all values are percents)

	Number of Observed Events		
**Sample Size**	**n=0**	**n=1**	**n=2**
15	[0.0, 21.8]	[0.2, 32.0]	[1.7, 40.5]
30	[0.0, 11.6]	[0.1, 17.2]	[0.8, 22.1]
60	[0.0, 6.0]	[0.0, 8.9]	[0.4, 11.5]

If no safety events are observed, then the upper limit of a 95% CI would be 21.8% for an individual group. Collapsing across prior vaccination history and doses would provide 60 subjects for cAd3-EBO and no safety events would correspond to an upper limit of 6.0%.

Pairwise group comparisons in event rates (or e.g. antibody response rates) between dose levels would have 81% power to detect a difference of 50% (n=15 in both groups, p1=0.2 and p2=0.7 using a two-sided 5% exact unconditional test, StatXact 9) while a collapsed comparison (e.g. comparing cAd3-EBO dose levels ignoring previous vaccination history) would have 81% power to detect a 35% difference (n1=n2=30, p1=0.2, and p2=0.55).

For continuous safety measures (and for continuous secondary immunogenicity endpoints), pairwise group comparisons using a two-sided 5% level t-test and assuming equal standard deviations in each group would have 83% power to detect a difference in means of 1.1 standard deviations for 15 subjects per group and 81% power to detect a difference in means of 0.75 standard deviations for 30 subjects per group.

Given the early phase nature of this trial, no adjustments for multiple comparisons are planned and significance will be assessed at the 0.05 level.

The design was modified via protocol amendment to incorporate an optional boost during the 24-48 weeks after initial vaccination. All subjects meeting the eligibility criteria for Part II will be offered the opportunity to receive the boost. As the boost is optional, the analysis is primarily intended to be descriptive although formal comparisons may occur as described below if sufficient subjects receive the boost.

9.1. Analysis

9.1.1. Primary Endpoints

All analyses will be described overall and by vaccine, dose, and previous Ebola vaccination history (yes/no). For continuous data, appropriate transformations (log, square root, or arcsin) will be applied as necessary.

Five primary endpoints related to safety are described in Section 4.1 Solicited reactogenicity signs and symptoms (local and systemic) will be described by frequency and severity. Proportion of subjects experiencing each event or any event will be described with an exact two-sided 95% CI. Adverse events (and serious adverse events) will be described similarly, (frequencies and proportions) and as well as by System Organ Class and Preferred Term. Continuous laboratory safety measures will be described as change from baseline at various timepoints using means and 95% CIs (or medians and interquartile ranges (IQRs)). Hypothesis testing will be performed if appropriate and the comparison timeframe will account for the optional boost (e.g. SAE rates may be compared during the first 24 weeks post-initial vaccination as subjects are allowed to receive the optional boost as early as 36 weeks). Key comparisons include differences between vaccines, dose levels, and a comparison using the dose by previous Ebola vaccination interaction. For pairwise group comparisons of proportions, exact unconditional tests will be used while for a multiple group comparison chi-square or Fisher’s Exact test will be performed. For continuous laboratory safety measures, t-tests and ANOVAs (or non-parametric equivalents of each) will be performed.

Subjects participating in the optional boost vaccination will be analyzed similarly. Time from initial injection will also be described as the timing of the boost is allowed to vary from 24-48 weeks. Further reasons for not receiving the optional boost (lost to follow-up, ineligible, refused, etc) will be described for each group and dose level. Proportion of subjects opting to receive the boost could be compared between groups and doses.

9.1.2 Secondary Endpoints

Immunogenicity endpoints comprise the key secondary endpoints (specifically ELISA and neutralization antigen-specific assays for antibody response and intracellular cytokine staining (ICS)). Data can be assessed as both proportions of responders (and 95% CIs) as well as continuous measures (means and 95% CIs or medians and IQRs).

The key time point is Week 4 post-initial vaccination. For continuous variables, comparisons between vaccines and dose levels within vaccine will be performed using either a t-test or the non-parametric equivalent if appropriate. Further, an ANOVA (or non-parametric equivalent) will compare groups defined by dose and previous Ebola vaccination. For binary antibody response,

pairwise group comparisons will be done using an exact unconditional test whereas multiple group comparisons will be performed using a chi-square or Fisher's exact test.

If sample sizes and/or event rates are sufficient, changes in antibody response over time will be assessed using repeated measures models to account for within subject correlation, adjusting for vaccine, dose and previous Ebola vaccination. Binary and continuous versions of immunogenicity endpoints will be considered.

For the boost vaccination, the key timepoint for assessment is 4 weeks post-boost. Similar methods will be applied although formal comparisons will be dependent upon sufficient subjects opting to receive the boost vaccination.

9.1.3 Exploratory Endpoints

Similar methods as those described above will be used for the exploratory endpoints.

9.2. Interim Analyses of Immunogenicity

Interim analyses of immunogenicity for each group may be performed during the study for the purpose of informing future product development decisions in a timely manner once there are at least 5 subjects in a group with the 4 weeks post vaccination follow-up completed. The analysis should not influence the conduct of the trial in terms of early termination or sample size adjustment, or affect later safety or immunogenicity endpoint assessments.

10. DATA COLLECTION

Clinical research data will be collected in a secure electronic data management system through a contract organization, EMMES (Rockville, MD). Data entered into the electronic data system shall be performed by authorized individuals. Corrections to the electronic data system shall be tracked electronically (password protected) with time, date, individual making the correction, and what was changed.

The investigator is responsible for assuring that the data collected is complete, accurate, and recorded in a timely manner. Source documentation (the point of initial recording of information) should support the data collected in the system, and must be signed and dated by the person recording and/or reviewing the data. Extracted data without patient identifiers will be sent to the Protocol Statistician for statistical analysis.

This data does not contain participant names or Social Security or other national identification number, but is referenced only by the study specific identification code.

Every attempt must be made to follow the protocol and to obtain and record all data requested for each subject at the specified times. However, ethical considerations or other events may result in the failure to obtain and record certain data, or to record data at the times specified. If this occurs, the events and, the reasons for the event must be clearly documented on the case report form for deviations and reported as described above.

Analysis files are created on a periodic basis and made available to the Protocol Chair, Principal Investigator (PI), and Associate Investigators (AIs) at the direction of the Protocol Chair. Other collaborators may be given access to these analysis files, or data gathered from them, at the direction of the Protocol Chair.

11. ETHICAL CONSIDERATIONS

11.1. Participation Of Children

Children are not eligible to participate in this clinical trial because it does not meet the guidelines for inclusion of children in research. These guidelines (45 CFR 46, Subpart D, 401-409) state the Department of Health and Human Services protections for children who participate in research. Generally, healthy children can be studied when the research is considered as "not greater than minimal risk." Children can be involved in research with greater than minimal risk only when it presents the prospect of direct benefit to the individual child or is likely to yield generalizable knowledge about the child's disorder or condition.

11.2. Risks

11.2.1. Risks of the cAd3 Ebola Vaccines and Diluent

Potential side effects resulting from intramuscular injection include stinging, arm discomfort, redness of the skin or mild bruising at vaccine injection sites. Study subjects can receive medications such as acetaminophen, NSAIDs, or antihistamines as required.

Subjects may exhibit general signs and symptoms associated with administration of a vaccine or placebo injection, including fever, chills, rash, aches and pains, nausea, headache, dizziness and fatigue. These side effects will be monitored, but are generally short term and do not require treatment. As with all vaccines, an allergic reaction is possible.

Chimpanzee adenoviral vector vaccines have been generally safe in healthy adults at dosages up to 10^{11} PU per injection in completed and ongoing clinical trials [24,25].

An Ebola-rAd5 vaccine, previously evaluated by the VRC, was associated with asymptomatic prolongations in the activated PTT (aPTT) in the 2 weeks following vaccination in two subjects, which after further investigation were assessed as due to the induction of a non-specific anti-phospholipid antibody (APA) and not due to coagulopathy. This effect is an artifact of the aPTT test as this test measure the clotting cascade and the assay requires the presence of phospholipid as a reagent. The presence of any APA can result in an elevated aPTT *in vitro*. In these 2 subjects, the aPTT returned to normal within 4 to 8 weeks as the APA waned, consistent with the half-life of immunoglobulin. Other laboratory tests, including coagulation factors, thrombin time and prothrombin time were normal and there were no clinical abnormalities associated with the in-vitro aPTT findings [17]. These APA were further evaluated and determined to be part of the acute phase response and were not consistent with anti-phospholipids that are known to be associated with disease. The basis for this in vitro phenomenon has been reported by others and observed following administration of other adenovirus vectored products[30-34]. The protocol includes a plan for assessing the basis for abnormal aPTT findings, if observed, following receipt of cAd3 vaccines.

There may be other unknown side effects.

11.2.2. Risks of the MVA-EbolaZ Vaccine

The first and the second generation smallpox vaccines have been associated with increased incidence of developing myoperiocarditis [36], however, no cases of myopericarditis have been reported with the highly attenuated modified vaccinia Ankara (MVA) based vaccines in healthy or immunocompromised volunteers [37-45]. In the VRC 208 study, subjects will be screened for cardiovascular risks using the non-laboratory method [46]. A baseline electrocardiogram (ECG) will be performed to serve as a baseline if further evaluations are required later.

Overall, MVA-vectored vaccines targeted to different pathogens have been evaluated as safe in Phase I/II studies in healthy and immunocompromised adults and infants [37-45].

The most often observed local reactogenicity include pain, redness and swelling at injection site. Some subjects may experience pruritus at the site of injection.

Subjects may exhibit general signs and symptoms associated with administration of a vaccine, including fatigue, headache, myalgia, nausea, chills and fever.

The side effects will be monitored, but these are generally short term and do not require treatment. As with all vaccines, an allergic reaction is possible.

Study subjects can receive medications such as acetaminophen, NSAIDs, or antihistamines as required.

There may be other unknown side effects.

11.2.3. Other Risks

Blood drawing may cause pain, bruising, fainting, and, rarely, infection at the site where the blood is taken.

Women of reproductive potential will be required to agree to use birth control for sexual intercourse beginning 21 days prior to enrollment and continue through 24 weeks after the study injection. Because this is a research study, women of reproductive potential will be asked to notify the site immediately upon learning of a pregnancy during this study and will be tested for pregnancy prior to administration of the study injection. The amount of blood drawn will be reduced. The subject will be contacted to ask about the outcome of a pregnancy that begins during the study.

It is possible that the standard medical tests performed as part of this research protocol will result in new diagnoses. Depending upon the medical findings and consequences of being provided with the new medical information about health status, the study subject may view this aspect of study participation as either a risk or a benefit. Any such information will be shared and discussed with the subject and, if requested by the subject, will be forwarded to the subject’s primary health care provider for further workup and management.

11.3. Benefits

Although study volunteers may benefit from clinical testing and physical examinations, study subjects will receive no direct benefit from participation.

In light of the current Ebola outbreak in 6 countries in West Africa that has killed more than 5,160 people (of an estimated 14,000 cases) and its potential for international spread, the general population in Uganda (and West Africa in particular), may benefit from information gained from the development of a vaccine against Ebola. Further, Uganda has itself experienced 3 outbreaks of Ebola between 2000 and 2012 with a case fatality rate ranging from 25% to 71%. While prior outbreaks of EVD have been localized to regions of Africa, there is a continued potential threat of spread to other countries given the frequency of international travel.

Others may benefit from knowledge gained in this study that may aid in the development of an Ebola virus vaccine.

11.4. Informed Consent

The study informed consent is provided in Appendix 2. It describes the investigational product to be used and all aspects involved in protocol participation. A properly executed written site specific informed consent based on the template provided here, in compliance with the Declaration of Helsinki, guidelines of the Council of International Organization of Medical Sciences (CIOMS), the Belmont Report, the U.S. Code of Federal Regulations 21 CFR 50, must be obtained from each subject prior to entering the subject into trial or prior to performing any unusual or non routine procedure that involves risk to the subject. The investigator must provide a copy of the approved informed consent to the subject and a signed copy must be maintained in the subject's record file. Before a subject's participation in the study, it is the investigator's responsibility to obtain this written informed consent from the subject, after adequate explanation of the aims, methods, anticipated benefits, and potential hazards of the study and before any protocol-specific procedures or study medications are administered.

11.5. Language

All written information and other material to be used by participants and investigative staff must use vocabulary and language that are clearly understood. Accordingly, the consent and all other written materials will be translated into Luganda in addition to English and will be submitted to the IRBs for review and approval.

11.6. Compensation

Participants will be compensated for time and inconvenience in accordance with the standards and legal obligations for compensation required by each study site. Any applicable guidelines by IRBs/ECs for compensation of research subjects will be sought and followed.

Suggested compensation is 50,000 Ugandan shillings for time and transportation costs associated with each scheduled visits, and 20,000 Ugandan shillings for each unscheduled visits to address safety concerns.

11.7. Local Medical Research Monitor (LMRM)

The role of the local medical research monitor are identified as follows:

The local medical research monitor (LMRM) will serve as an independent physician who can be approached for medical information by volunteers, act as their advocate and assess their medical care for events which occur during the course of the trial. He/she will also oversee the progress of the clinical trial and ensure that is it conducted, recorded, and reported in accordance with the protocol, standard operating procedures (SOPs), GCP, and the applicable regulatory requirements.

11.8. Policy Regarding Research-Related Injuries

The US DoD and NIAID/NIH are funding this protocol. As stated in the consent form, participants who experience illness or injury arising from participation in the study will receive medical care for such illness or injury with costs for such care provided by a limited set-aside fund and a clinical trials medical insurance policy that will be obtained by MHRP (HJF). While we anticipate the combination of the set-aside fund and the insurance policy is more than enough to pay for the research related injury medical care cost associated with this study, there is a limited to the amount of coverage available. If the limit is exceeded, the study subject may have to pay non-covered costs. Other than medical care, and other payments as stated in the consent form, there is no other compensation available from this research study.

11.9. Participant Confidentiality

The PI will maintain research records of participant's participation at the site for this study. All participants will receive study numbers that are known only to the investigators and clinic staff. Clinical tests will be identified by study number and the specimen bag will be identified by study number and/or bar code only. Clinical and research records may be reviewed by the representatives of NIH, USAMRMC, WRAIR, representatives of the USAMRMC ORP, representatives of the FDA, OHRP, the product, and other regulatory agencies as part of their responsibilities for insuring the protection of research participants.

Every effort will be made to keep the records as confidential as possible within the limits of the law. All data and medical information obtained about participants as individuals will be considered privileged and held in confidence. Research and clinical information relating to participants will be shared with other investigators and the scientific community through presentation or publication; however, participants will NOT be identified by name or national registration number. Electronic data will be stored at least as long as the IND remains open.

11.10. Institutional Review Board

A copy of the protocol, proposed informed consent form, other written participant information, and any proposed advertising material will be submitted to the appropriate ethical and scientific review committees in each country for review and approval.

In addition, the protocol will undergo review and approval by WRAIR, the MUSPH IRB, UNCST and the USAMRMC ORP at Medical Research and Materiel Command, U.S. Army (MRMC).

11.11. Future Use and Storage of Blood Samples

Each study participant will be asked to separately, and voluntarily consent to their blood samples to be stored for other research studies that may be done after this study is completed. Future

testing may involve genetic tests. As stated above, the sample will be labeled with the bar code of the subject ID that can be linked to their study information.

All samples for which consent has been obtained and for which additional material is available after study specified testing is complete will be stored for future testing at the site. Samples will be stored for 5 years.

However, WRAIR and the MUSPH IRB approval will be sought before any such samples are used for analysis not specified in the protocol or a protocol amendment approved by the IRB, or if the samples need to be stored for longer than 5 years. All samples belong to the site from which they were obtained and MHRP.

11.12. Study Documentation And Storage

The investigator will maintain a list of appropriately qualified persons to whom trial duties have been delegated

Source documents are original documents, data, and records from which the subject's data are obtained. These include but are not limited to hospital records, clinical and office charts, laboratory and pharmacy records, diaries, microfiches, radiographs, and correspondence.

The investigator and staff are responsible for ensuring maintenance of a comprehensive and centralized filing system of all study-related (essential) documentation, suitable for inspection at any time by representatives from the WRAIR, MRMC, NIAID/ VRC, FDA, and/or applicable regulatory authorities. Essential documents for all study subjects are to be maintained by the investigators in a secure storage facility. Elements include:

- Participant files containing completed informed consent forms, and supporting copies of source documentation (if kept)
- Study files containing the protocol will include all amendments and copies of correspondence between the study site, protocol team and the IRB
- All Essential Documents outlined in the ICH Good Clinical Practice Guideline.

In addition, all original source documentation must be maintained and be readily available.

All essential documentation should be retained by the institution for the same period of time required for medical record retention. The FDA requires study records to be retained for 2 years after marketing approval or refusal (21 CFR 312.62). According to the NIH FWA, records must be retained for a minimum of 3 years. No study document should be destroyed without prior written agreement between VRC, the Protocol Chair and the PI. Should the PI wish to assign the study records to another party or move them to another location, VRC must be notified in writing of the new responsible person and/or new location.

The VRC may request to receive a copy of all essential documents. FDA forms F1572, CVs and IRB approvals must be submitted to the VRC as IND Sponsor.

12. ADMINISTRATIVE AND LEGAL PROCEDURES

12.1. Protocol Deviation Reporting

A protocol deviation is defined as an isolated occurrence involving a procedure that did not follow the study protocol.

The timeline for reporting protocol deviations to the WRAIR Human Subjects Protection Branch and MUSPH IRB is determined by the categorization of the deviation: (1) emergent/significant or (2) non-emergent/minor. Protocol deviations arising of or leading to unanticipated problems should be reported in the appropriate timeframe according to the seriousness of the event as a significant deviation or a minor deviation. The unanticipated problem will be submitted as described in Section 8.9.

Emergent/significant deviations are departures from protocol that have a significant impact on the welfare or safety of a volunteer or on the integrity of the study data. Examples: providing the wrong lab result to a volunteer or failure to obtain a scheduled blood draw for multiple participants. Changes in protocol procedures may be initiated without prior IRB/ethical review committee (ERC) approval, only in cases where the change (s) is /are necessary to eliminate an immediate apparent hazard. Emergent/significant deviations should be reported promptly (within 48 hours) to the MUSPH IRB, UNCST, WRAIR HSPB and the IND sponsor, upon becoming aware of the event, by telephone or email. A written report is required to be submitted by the PI to the HSPB within 10 working days and to the MUSPH IRB and UNCST within 7 calendar days of knowledge of the significant deviation. Deviations will be reported by the WRAIR HSPB to USAMRMC ORP HRPO.

Non-emergent/minor deviations are routine departures that typically involve a volunteer's failure to comply with the protocol. Examples include missing scheduled visits and failing to complete a required questionnaire. Minor deviations will be reported to the sponsor, the MUSPH IRB, UNCST and the WRAIR Human Subjects Protection Branch in a summary report with the annual continuing review report.

A cumulative deviation report will be submitted to the IND Sponsor, MUSPH IRB and WRAIR HSPB with each protocol continuing review report or with the closeout report, whichever comes first.

12.2. Protocol Modifications

Amendments to the protocol will be made only after consultation and agreement between NIAID VRC, the protocol chair, and the principal investigators. All protocol modifications (including but not limited to changes in the principal investigator, inclusion/exclusion criteria, number of participants to be enrolled study sites, or procedures) must be submitted as a written amendment to the MUSPH IRB, NDA (as specified below), WRAIR HSPB and IND sponsor for approval before implementation of the changes. The WRAIR HSPB will submit protocol amendments and modifications to the USAMRMC ORP HRPO.

Protocol amendments will be submitted to the NDA for review and approval prior to implementation if the amendment has the potential to impact the safety of the participants (e.g. changes to dose, regimen etc.). If the amendment is unlikely to impact on participant safety (e.g. administrative changes, changes to end point assays, laboratory, statistical analysis), the amendment will be submitted and the change may be implemented 14 days after receipt of the amendment by NDA, if no notification to the contrary is received by the applicant within that period. The UNCST shall be notified of all protocol amendments with submission of local IRB approval for each amendment.

Modifications or updates to the Investigational Brochures (IBs) will also be submitted as protocol amendments to the NDA (as specified above) and IRB for review.

The Informed Consent Form must be revised to concur with any significant amendment that directly affects volunteers, and must also be reviewed and approved with the amendment. New volunteers enrolled in the study will be consented with the most recent approved consent form. Volunteers already enrolled in the study will be informed about the revision and, depending on the impact of the amendment, may be asked to re consent. This may be accomplished by repeating the consent process with the revised consent form with attention given to the changes, or it may be done using an addendum consent that states the revision or new information. The new document must be signed, placed in the study record, and a copy given to the volunteer.

Administrative changes to the protocol are corrections and/or clarifications that have no effect on the way the study is to be conducted. Such administrative changes will be submitted to the IRB for review and approval prior to implementation.

The VRC/NIAID, the PI, local IRB, NDA, UNCST and the FDA reserve the right to terminate the study. The investigator will notify the IRB in writing of the study's completion or early termination.

12.3. Continuing Reviews /Closeout Report

A continuing review report (CRR) will be submitted to all IRBs and NDA prior to the anniversary date determined at initial IRB review. If the continuing review is not approved by the MUSPH IRB, NDA and WRAIR by the anniversary date, all protocol activities must stop at the site until such time as the approval is obtained. A copy of the approved CRR and local IRB approval notifications will be submitted to the WRAIR HSPB/ MRMC ORP HRPO as soon as these documents become available. A copy of the approved closeout report and local IRB approval notifications thereof, will be submitted to the WRAIR HSPB/ MRMC ORP HRPO as soon as these documents become available.

Under current UNCST procedure, a protocol shall receive an approval for a period as may be applied for. Prior to expiration of that period, the Investigator will submit a request for extension of the approval with rationale. Copies of the annual CRR shall be submitted to the UNCST along with local IRB approval.

12.4. Volunteer Registry Database

It is the policy of the USAMRMC that Volunteer Registry Data Sheets are completed on all volunteers participating in greater than minimal risk research for entry into the Command's Volunteer Registry Database. Ordinarily this information would include the individual's unique identifier (e.g. SSN in the U.S.A.), study name and dates. But because many countries do not have a national identification system that would uniquely identify each person in the country, the Volunteer Registry Data Sheets will collect the following data on the volunteers:

- names (first and last name)
- date of birth, home district
- study name and study dates

The intent of the database is two-fold: first, to readily answer questions concerning an individual's participation in research sponsored by USAMRMC; and second, to ensure that the USAMRMC can exercise its obligation to ensure research volunteers are adequately warned (duty to warn) of risks and to provide new information as it becomes available. The information will be stored at USAMRMC for a minimum of 75 years. In countries other than the U.S., social security numbers do not exist and cannot be collected; all other data can. If available, country-specific identity numbers may be used.

12.5. Use Of Information And Publication

It is expected that data from this study will be reported in both scientific journals and international scientific meetings. Confidentiality of subjects will be maintained by the fact that no individual results will be reported or published, only group/aggregate results. All research data will be identified by the study number. The linkage between personal identifiers and study number will only be available in a confidential database at the respective sites. The local health authorities will be informed of all scientific outcomes of the study and general prevalence and incidence data however, confidentiality will be maintained, and participant identities will not be released. Only aggregate information will be released. All publications resulting from this study will be cleared through the collaborating partners to this study.

WRAIR recognizes the importance of communicating medical study data and therefore encourages their publication in reputable scientific journals and at seminars or conferences. Any results of medical investigations and/or publication/lecture/manuscripts based thereon, shall be exchanged and discussed by the investigator, the sponsor representative(s) and the U.S. Army Medical Research and Materiel Command 60 days prior to submission for publication or presentation.

Results from investigations shall not be made available to any third party by the investigating team outside the publication procedure as outlined previously. WRAIR will not quote from publications by investigators in its scientific information and/or promotional material without full acknowledgment of the source (i.e., author and reference). All publications written by WRAIR investigators must be reviewed and approved by WRAIR Office of Research Technology and Applications (ORTA).

13. CONDUCT OF THE RESEARCH STUDY

This research study will be conducted in accordance with GCP, ICH guidelines, DOD Directive 3216.2, the Declaration of Helsinki, the Belmont Report, the U.S. Code of Federal Regulations 21 CFR 312, 812, 50 and 56, and all applicable local laws and regulations.

13.1. Regulatory Audits

The knowledge of any pending compliance inspection/visit by the US FDA, OHRP, or other government agency concerning clinical investigation or research, the issuance of Inspection Reports, FDA Form 483, warning letters or actions taken by any regulatory agencies including legal or medical actions and any instances of serious or continuing noncompliance with the regulations or requirements will be reported immediately to the IND sponsor, WRAIR HSPB and MUSPH IRB. The WRAIR HSPB will report knowledge of any pending inspections/audits by regulatory agencies to the USAMRMC ORP HRPO.

13.2. Sponsor Study Monitoring

Site visits by the study monitor will include the following: study operations, the quality of data collected in the research records, the accuracy and timeliness of data entered in the database, and to determine that all process and regulatory requirements are met.

Site investigator will allow the study monitors, representatives of the VRC or designee, IRB, NDA, UNCST, MRMC, and the FDA to inspect study documents (e.g., consent forms, drug distribution forms, case report forms), and pertinent hospital or clinic records for confirmation of the study data.

Study data will be closed and final after data cleaning activities are completed and resolutions have been documented.

14. PRINCIPAL INVESTIGATOR AGREEMENT

1. I agree to follow this protocol version as approved by the IRBs/ERCs.
2. I will conduct the study in accordance with applicable IRB/ERC requirements, Federal regulations, and state and local laws to maintain the protection of the rights and welfare of study participants.
3. I certify that I, and the study staff, have received the requisite training to conduct this research protocol.
4. I will not modify the protocol without first obtaining an IRB/ERC approved amendment and new protocol version unless it is necessary to protect the health and welfare of study participants.
5. I have read and understand the information in the Investigators' Brochure (or Manufacturer's Brochure) regarding the risks and potential benefits. I agree to conduct the protocol in accordance with Good Clinical Practices (ICH-GCP), the applicable ethical principles, the Statement of Investigator (Form FDA 1572), and with local regulatory requirements. In accordance with the FDA Modernization Act, I will ensure the registration of the trial on the www.clinicaltrials.gov website.
6. In accordance with Command Policy 2008-35, I will ensure that the Commanding General receives a pre-brief (or Executive Summary) and approves the study prior to execution.
7. I will ensure that the data (and/or specimens) are maintained in accordance with the data (and/or specimen) disposition outlined in the protocol. Any modifications to this plan should first be reviewed and approved by the applicable IRBs/ERCs.
8. I will promptly report changes to the research or unanticipated problems to the WRAIR IRB immediately via the WRAIR Human Subjects Protection Branch at (301) 319-9940 (during duty hours) or to the usarmy.detrick.medcom-wrair.mbx.hspb@mail.mil and submit a written report within 10 working days of knowledge of the event.
9. I will prepare continuing review reports at an interval established by the IRB/ERC, and a study closure report when all research activities are completed.
10. I will immediately report to the WRAIR Human Subjects Protection Branch knowledge of any pending compliance inspection by any outside governmental agency.

11. I agree to maintain adequate and accurate records in accordance with IRB policies, Federal, state and local laws and regulations.

____

RV 422 PART I & PART II Protocol Co-Chair Printed Name/Signature Date (DD/MM/YYYY)

____

RV 422 PART I & PART II Protocol Co-Chair Printed Name/Signature Date (DD/MM/YYYY)

____

RV 422 PART I & PART II PI Printed Name/Signature Date (DD/MM/YYYY)

15. REFERENCES

1. Kuhn, J.H., et al., Virus nomenclature below the species level: a standardized nomenclature for natural variants of viruses assigned to the family Filoviridae. Arch Virol, 2013. **158**(1): p. 301-11.
2. Hart, M.K., *Vaccine research efforts for filoviruses.* Int J Parasitol, 2003. **33**(5-6): p. 583-95.
3. Sanchez, A., et al., Sequence analysis of the Ebola virus genome: organization, genetic elements, and comparison with the genome of Marburg virus. Virus Res, 1993. **29**(3): p. 215-40.
4. Sanchez, A., et al., Sequence analysis of the Marburg virus nucleoprotein gene: comparison to Ebola virus and other non-segmented negative-strand RNA viruses. J Gen Virol, 1992. **73 (Pt 2)**: p. 347-57.
5. WHO. *Ebola Virus Diseaase*. 2014 [cited 2014 August 12]; Available from: http://www.who.int/mediacentre/factsheets/fs103/en/.
6. Geisbert, T.W. and P.B. Jahrling, *Exotic emerging viral diseases: progress and challenges.* Nat Med, 2004. **10**(12 Suppl): p. S110-21.
7. Meslin, F.X., Global aspects of emerging and potential zoonoses: a WHO perspective. Emerg Infect Dis, 1997. **3**(2): p. 223-8.
8. Okware, S.I., et al., *An outbreak of Ebola in Uganda.* Trop Med Int Health, 2002. **7**(12): p. 1068-75.
9. Hensley, L.E., et al., Ebola and Marburg viruses: pathogenesis and development of countermeasures. Curr Mol Med, 2005. **5**(8): p. 761-72.
10. Sullivan, N., Z.Y. Yang, and G.J. Nabel, *Ebola virus pathogenesis: implications for vaccines and therapies.* J Virol, 2003. **77**(18): p. 9733-7.
11. Dixon, M.G., et al., *Ebola viral disease outbreak--West Africa, 2014.* MMWR Morb Mortal Wkly Rep, 2014. **63**(25): p. 548-51.
12. Verheust, C., et al., *Biosafety aspects of modified vaccinia virus Ankara (MVA)-based vectors used for gene therapy or vaccination.* Vaccine, 2012. **30**(16): p. 2623-32.
13. Gomez, C.E., et al., *Clinical applications of attenuated MVA poxvirus strain.* Expert Rev Vaccines, 2013. **12**(12): p. 1395-416.
14. Gilbert, S.C., *Clinical development of Modified Vaccinia virus Ankara vaccines.* Vaccine, 2013. **31**(39): p. 4241-6.
15. Sutter, G. and B. Moss, *Nonreplicating vaccinia vector efficiently expresses recombinant genes.* Proc Natl Acad Sci U S A, 1992. **89**(22): p. 10847-51.

12\. Martin, J.E., et al., A DNA vaccine for Ebola virus is safe and immunogenic in a phase I clinical trial. Clin Vaccine Immunol, 2006. **13**(11): p. 1267-77.

13\. Ledgerwood, J.E., et al., A replication defective recombinant Ad5 vaccine expressing Ebola virus GP is safe and immunogenic in healthy adults. Vaccine, 2010. **29**(2): p. 304-13.

14\. Ledgerwood, J.E., Enama, M.E., Costner, P., Hu, Z., Hendel, C.S., Sullivan, N., Koup, R., Graham, B. S., VRC 206: Phase I Clinical Trial of Ebola and Marburg DNA Vaccines in Healthy Adults, in National Foundation of Infectious Diseases, 15th Annual Conference. 2012: Baltimore, MD. .

19. Kibuuka, H., et al., *Safety and immunogenicity of Ebola virus and Marburg virus glycoprotein DNA vaccines assessed separately and concomitantly in healthy Ugandan adults: a phase 1b, randomised, double-blind, placebo-controlled clinical trial.* Lancet, 2014.
20. Stanley, D.A., et al., *Chimpanzee adenovirus vaccine generates acute and durable protective immunity against ebolavirus challenge.* Nat Med, 2014. **20**(10): p. 1126-9.
15. Yang, Z.Y., et al., Identification of the Ebola virus glycoprotein as the main viral determinant of vascular cell cytotoxicity and injury. Nat Med, 2000. **6**(8): p. 886-9.
16. Sullivan, N.J., et al., Immune protection of nonhuman primates against Ebola virus with single low-dose adenovirus vectors encoding modified GPs. PLoS Med, 2006. **3**(6): p. e177.
23. Ledgerwood, J.E., et al., *Chimpanzee Adenovirus Vector Ebola Vaccine - Preliminary Report.* N Engl J Med, http://www.nejm.org/doi/full/10.1056/NEJMoa1410863, 2014.
17. Barnes, E., et al., Novel adenovirus-based vaccines induce broad and sustained T cell responses to HCV in man. Sci Transl Med, 2012. **4**(115): p. 115ra1.
18. O'Hara, G.A., et al., Clinical assessment of a recombinant simian adenovirus ChAd63: a potent new vaccine vector. J Infect Dis, 2012. **205**(5): p. 772-81.
19. Colloca, S., et al., Vaccine vectors derived from a large collection of simian adenoviruses induce potent cellular immunity across multiple species. Sci Transl Med, 2012. **4**(115): p. 115ra2.
20. Peruzzi, D., et al., A novel chimpanzee serotype-based adenoviral vector as delivery tool for cancer vaccines. Vaccine, 2009. **27**(9): p. 1293-300.
21. Quinn, K.M., et al., Comparative analysis of the magnitude, quality, phenotype, and protective capacity of simian immunodeficiency virus gag-specific CD8+ T cells following human-, simian-, and chimpanzee-derived recombinant adenoviral vector immunization. J Immunol, 2013. **190**(6): p. 2720-35.
22. Betts, M.R., J.P. Casazza, and R.A. Koup, *Monitoring HIV-specific CD8+ T cell responses by intracellular cytokine production.* Immunol Lett, 2001. **79**(1-2): p. 117-25.
23. Cervera, R., et al., Antiphospholipid syndrome associated with infections: clinical and microbiological characteristics of 100 patients. Ann Rheum Dis, 2004. **63**(10): p. 1312-7.
24. Enama, M.E., et al., Induction of false-positive PTT elevations by investigational adenoviral vector vaccines, in 14th Annual Conference on Vaccine Research. 2011: Baltimore, MD.
25. Jaeger, U., et al., Transient lupus anticoagulant associated with hypoprothrombinemia and factor XII deficiency following adenovirus infection. Ann Hematol, 1993. **67**(2): p. 95-9.
26. Malaeb, B.S., et al., Elevated activated partial thromboplastin time during administration of first-generation adenoviral vectors for gene therapy for prostate cancer: identification of lupus anticoagulants. Urology, 2005. **66**(4): p. 830-4.
27. Shoenfeld, Y., M. Blank, and I. Krause, The relationship of antiphospholipid antibodies to infections--do they bind to infecting agents or may they even be induced by them? Clin Exp Rheumatol, 2000. **18**(4): p. 431-2.
28. Stanley, D.A., st al., Chimpanzee adenovirus vaccine generates acute and durable protective immunity against ebolavirus challenge. Nat Med, 2014.
36. *ACAM2000 Vaccines and Related Biological Products Advisory Committee (VRBPAC) Briefing Document* 2007 [cited 2014, December 8]; Available from: http://www.fda.gov/ohrms/dockets/ac/07/briefing/2007-4292b2-02.pdf.

37. Vollmar, J., et al., *Safety and immunogenicity of IMVAMUNE, a promising candidate as a third generation smallpox vaccine.* Vaccine, 2006. **24**(12): p. 2065-70.
38. von Krempelhuber, A., et al., *A randomized, double-blind, dose-finding Phase II study to evaluate immunogenicity and safety of the third generation smallpox vaccine candidate IMVAMUNE.* Vaccine, 2010. **28**(5): p. 1209-16.
39. Frey, S.E., et al., *Phase II randomized, double-blinded comparison of a single high dose (5x10(8) TCID50) of modified vaccinia Ankara compared to a standard dose (1x10(8) TCID50) in healthy vaccinia-naive individuals.* Vaccine, 2014. **32**(23): p. 2732-9.
40. Frey, S.E., et al., *Safety and immunogenicity of IMVAMUNE(R) smallpox vaccine using different strategies for a post event scenario.* Vaccine, 2013. **31**(29): p. 3025-33.
41. Peters, B.S., et al., *Studies of a prophylactic HIV-1 vaccine candidate based on modified vaccinia virus Ankara (MVA) with and without DNA priming: effects of dosage and route on safety and immunogenicity.* Vaccine, 2007. **25**(11): p. 2120-7.
42. Gorse, G.J., et al., *DNA and modified vaccinia virus Ankara vaccines encoding multiple cytotoxic and helper T-lymphocyte epitopes of human immunodeficiency virus type 1 (HIV-1) are safe but weakly immunogenic in HIV-1-uninfected, vaccinia virus-naive adults.* Clin Vaccine Immunol, 2012. **19**(5): p. 649-58.
43. Antrobus, R.D., et al., *A T cell-inducing influenza vaccine for the elderly: safety and immunogenicity of MVA-NP+M1 in adults aged over 50 years.* PLoS One, 2012. **7**(10): p. e48322.
44. Lillie, P.J., et al., *Preliminary assessment of the efficacy of a T-cell-based influenza vaccine, MVA-NP+M1, in humans.* Clin Infect Dis, 2012. **55**(1): p. 19-25.
45. Tameris, M.D., et al., *Safety and efficacy of MVA85A, a new tuberculosis vaccine, in infants previously vaccinated with BCG: a randomised, placebo-controlled phase 2b trial.* Lancet, 2013. **381**(9871): p. 1021-8.
46. Gaziano, T.A., et al., *Laboratory-based versus non-laboratory-based method for assessment of cardiovascular disease risk: the NHANES I Follow-up Study cohort.* Lancet, 2008. **371**(9616): p. 923-31.

APPENDIX 1. SCHEDULE OF EVALUATIONS

		RV422 PART I Schedule of Evaluations 1 – cAd3-EBO or cAd3-EBOZ vaccination								
**Visit**	01	02	02A	02B	02C	03	04	05	06	07
**Week of Study**	-8 to 0	W 0	W 1	W1	W2	W4	W 8	W 16	W 24	W 48
[1]**Day of Study**	-56 to 0	D 0	D 3	D 7	D14	D 28	D 56	D 112	D 168	D 336
Clinical Evaluations										
RV422 PART I AoU, Informed Consent	X									
Physical exam and weight at screen; Vital signs, targeted exam at other visits.	X	X	X	X	X	X	X	X	X	X
Medical history for eligibility at screen; interim history other visits	X	X	X	X	X	X	X	X	X	X
Study Vaccination[2]		X								
Begin/review 7-Day Diary Card		X			X					
Counseling on pregnancy prevention		X			X	X	X	X		
CBC	3	3	3		3	3			3	
Pregnancy test: urine (or serum)[3]	X	X				X			X	
Creatinine and ALT	4	4	4		4	4				
HIV	X									
PT and PTT[4]	4	4			4	4				
HLA Type[5]							10			
Research Immunology										
Antibody assays and serum storage	4	4	4	4	4	4	4	4	4	4
PBMC and plasma for storage	40	80		60	25	80	60	60	80	80
Intracellular RNA		3	6	6						
Daily Volume (mL)	55	98	17	70	40	95	74	64	87	84
Max. Cumulative Volume (mL)	55	152	168	237	276	370	443	506	592	675

Visit windows: 02A (+1 day); 02B (+2 days); 02C (+3 days); Visit 02D (±3 days); Visits 03 and 04 (±7 days); Visits 05, 06 and 07 (±14 days) when not opting for Schedule 2. **If** opting for Schedule 2, complete Visit 07 (±12 weeks) any time after Week 36; evaluations shown on both Schedule 1 & 2 are needed to proceed.

[1] Most screening evaluations must be no more than 56 days prior to Day 0 to be used for eligibility (pregnancy test from Day 0 must be used for eligibility). If clinical assessment on Day 0 suggests significant changes may have occurred since screening, then physical examination & laboratory studies done on Day 0 are used for eligibility. Day 0=day of enrollment and vaccine injection. Day 0 evaluations prior to first injection are the baseline for assessing adverse events subsequently.

[2] Complete post vaccination blood pressure, pulse and injection site assessment after the study injection (30 to 60 minutes post-vaccination).

[3] Negative pregnancy test results must be confirmed for women of reproductive potential prior to administering the vaccine injection.

[4] Additional tests may be needed if results are not in range.

[5] HLA type blood sample is collected once at any time point in the study shown at Visit 04 for convenience; however, if HLA type is already available in the medical record it does not need to be repeated. HLA type may also be obtained from a frozen sample.

RV422 PART II Schedule of Evaluations 2 - MVA-EbolaZ vaccine booster											
**Visit**	[1]07	08	08A	08B	08C	09	10	11	12	13	14
**Week of Study**	-28 to -1 days	W 0	W1	W1	W1	W2	W4	W8	W16	W24	W48
**[1]Day of Study**		D 0	D 1	D 2	D 5	D 14	D 28	D 56	D 112	D 168	D 336
**Clinical Evaluations**											
RV422 PART II Booster AoU, Informed Consent	X										
Vital signs, targeted exam		X		X	X	X	X	X	X	X	X
Interim medical history		X		X	X	X	X	X	X	X	X
[1] ECG	X										
[2] Study Vaccination		X									
Begin 7-Day Diary Card		X									
Phone contact; clinic visit if indicated			X								
Counseling on pregnancy prevention		X					X	X	X		
CBC		3		3		3	3	3		3	
[3] Pregnancy test: urine (or serum)		X					X			X	
Creatinine and ALT		4		4		4	4	4			
[4] PT and PTT		4		4		4	4				
**Research Immunology***											
Antibody assays and serum storage		16			16	16	24	24	24	24	24
PBMC and plasma for storage		40			80	40	80	50	60	80	80
Intracellular RNA		3		6	6						
**Daily Volume (mL)**		**59**		**17**	**102**	**67**	**115**	**81**	**84**	**107**	**104**
**Max. Cumulative Volume (mL)**		**59**		**76**	**178**	**245**	**360**	**441**	**525**	**632**	**736**

<u>Visit windows</u>: 08A (+1 day); 08B (+1 day); 08C (+2 days); Visit 09 (±3 days); Visits 10 and 11 (±7 days); Visits 12, 13, and 14 (±14 days).

[1] Evaluation for booster schedule participation is optional. For those who opt to participate, complete the evaluations shown on both schedules for "Visit 07," Visit 07 should be 1 to 28 days before Visit 08. On Schedule 2 Visit 08 is the day of booster vaccine injection and for purposes of timing subsequent visits is referred to as the Schedule 2 "Day 0." The Visit 08 evaluations prior to study injection are the baseline for assessing adverse events subsequently, except the baseline ECG is at Visit 07. Visit 07 and 08 should not be the same date.

[2] Complete post vaccination blood pressure, pulse and injection site assessment after the study injection (30 to 60 minute interval post-vaccination).

[3] Negative pregnancy test results must be confirmed for women of reproductive potential within 24 hours prior to administering the vaccine injection.

[4] Additional tests may be needed if results are not in range.

[5] Visits can occur outside the preferred window with approval by the Protocol Chair.

APPENDIX 2. CONSENT FORMS

Appendix 2a: MAIN CONSENT FORM

Makerere University Walter Reed Project
In Collaboration with the
U.S. Military HIV Research Program (MHRP)
And the
U.S. National Institutes of Health (NIH)

RV422 PART I & PART II
CONSENT TO BE A RESEARCH VOLUNTEER
MAIN CONSENT FORM

1. STUDY TITLE: "A Phase 1b, Open-Label, Clinical Trial to Evaluate the Safety, Tolerability and Immunogenicity of the Investigational Ebola Vaccines, VRC-EBOADC069-00-VP (cAd3-EBO) and VRC-EBOADC076-00-VP (cAd3-EBOZ), and VRC-EBOMVA079-00VP (MVA-EbolaZ), in Healthy Adults in Kampala, Uganda"

2. INTRODUCTION

You are being invited to participate in this study because you are a healthy person who may meet the criteria to join Part II of this study. Before deciding to participate in this study, please read this document thoroughly. In doing so, you will understand the purpose and details of this part of the study. If you have any further questions, please feel free to ask the study team (investigator doctors or nurses) at any time. The investigators will answer and make clarification pertaining to any matter.

Before you decide whether or not to take part in this study, we would like to explain the purpose of the research study, how it may help you or others, any risks associated with participation, and our expectations of you. This process is called informed consent. It is important that you know the following:

a. **Taking part is of your own free will (entirely voluntary).**

b. **If you decide not to participate you will not lose any of the benefits or rights you would normally have or be disadvantaged in any way.**

Please ask questions about anything you do not understand at any time. The clinic staff will talk with you about the information in this form. You can take as much time as you need to review this form and discuss your study participation with your family, friends, and community as you feel comfortable and appropriate, in order to decide whether or not you would like to participate. If you decide to participate in this study, you will review this document with a study staff and will be requested to sign this consent form. A copy of this informed consent will be provided to you.

You will also have the opportunity to consent for Future (currently unknown) use of your samples, and genetic testing. These will be explained to you, and you will sign a separate consent for each.

You have the right to withdraw from the study for any reason at any time; but please inform the study staff.

The technical names of the study vaccines are VRC-EBOADC069-00-VP, VRC-EBOADC076-00-VP and VRC-EBOMVA079-00-VP. We will refer to them as the "cAd3-EBO", "cAd3-EBOZ" or "MVA-EbolaZ" respectively or simply as the "Ebola vaccine." Vaccines are given to teach the body how to prevent or fight an infection. The study vaccines do not contain live or killed Ebola virus. It is **impossible** for the study vaccines to give you an infection.

This research study is funded by the U.S Army Department of Defense and NIAID/NIH.

3. PURPOSE AND BACKGROUND

This research study will evaluate experimental vaccines for the Ebola Virus. "Experimental" means that the study vaccines have not been approved by the US Food and Drug Administration (FDA) or the Uganda National Drug Authority (NDA). The FDA and the NDA have allowed their use in research studies only.

You cannot become infected with the Ebola virus from the vaccines.

It is not known if the vaccines work. The main purpose of this study is to see if the experimental vaccines are safe and if they cause any side effects. Another goal is to study blood samples in the lab to see if and how the immune system responds in people who receive these vaccines. Your immune system protects your body against infections.

The Ebola virus was discovered in 1976. It is named after a river in Africa close to where the virus was first discovered. Bats in certain parts of Africa may be a reservoir for the virus. The virus causes an infection known as Ebola virus disease (EVD). This disease starts with fever and muscle aches. More severe symptoms are breathing problems, severe bleeding, kidney problems, and shock. The infection may be mild, but it can also lead to death. The first two outbreaks of Ebola Hemorrhagic Fever in Africa in 1976 caused 340 deaths. In Africa, when there have been outbreaks of Ebola virus, 50% to 90% of infected people have died.

The Site Principal Investigator of this study is Dr. Hannah Kibuuka, at the Makerere University Walter Reed Project, Kampala, Uganda

This study will be conducted at the Makerere University Walter Reed Project. Kampala, Uganda.

4. STUDY VACCINES

Vaccines are substances used to create immune responses (resistance) to a disease in order to prevent infection. Immune responses are how your body recognizes and defends itself against bacteria, viruses, and substances that may be harmful to the body.

Many vaccines are made of proteins. Proteins are the basic chemicals that make up the structure of cells within our bodies and direct cell activities. An immune response happens when the body's cells come in contact with the vaccine proteins.

The experimental Ebola vaccines in this study were developed in a laboratory by the Vaccine Research Center (VRC) at the U.S. National Institutes of Health (NIH) located in Bethesda, Maryland USA. The cAd3-EBO and cAd3-EBOZ vaccines are made by using another virus called adenovirus (chimpanzee Adenovirus 3) to deliver manufactured DNA to cells in your body. The adenovirus used to make the vaccines is from a strain that infects chimpanzees. This strain of adenovirus does not cause human adenovirus infections and it is further changed to make sure it cannot reproduce in a human body. You cannot become infected with or infect someone else with either Ebola or adenovirus from receiving the study vaccines. The DNA in the vaccine allows cells in your body to make small amounts of a surface protein from Ebola. Your body will then make an immune response to this protein.

The MVA-EbolaZ vaccine is made by using a virus called "modified vaccinia virus Ankara" (MVA), to deliver manufactured DNA to cells in your body. MVA is a weakened form of the virus used to make vaccines against smallpox. The cells in your body will use vaccine to make small amounts of a protein from the Ebola Zaire type. Your body's immune system may then respond to this protein. The MVA vaccine cannot multiply in a human body. You cannot get Ebola or MVA and you cannot give it to someone else when you get the study vaccine.

5. STUDY PARTICIPATION

The study has two experiments – Part I and Part II. Only participants who received cAd3-EBO or cAd3-EBOZ vaccine and have completed at least 36 weeks of follow-up in Part I, will be eligible to participate in this study. If you agree to take part in the study you will have received one study injection in Part I, and you may receive one study injection in Part II. The vaccine injection will be given using a needle and syringe into an upper arm muscle. This is called an intramuscular "IM" injection. There is no placebo in this study.

A total of 90 volunteers were enrolled into Part I of this study. Part I was divided into 2 groups. Group 1 enrolled 60 participants. Group 2 was made up of 30 people who were previously enrolled in another Ebola vaccine study called RV 247. Part II will include all participants from Part I if you have been in the study for at least 36 weeks.

Vaccination Schedule for Part I and Part II:

	**RV 422 PART I & PART II Study Schema**			
**Group**	**Sub-Group**	**Subjects**	**Part I: At Day 0**	**Part II: After Week 36+**
**1**		**60**		
	1a	15	cAd3-EBOZ at $1x10^{10}$ PU IM	MVA-EbolaZ at $1x10^{8}$ PFU IM
	1b	15	cAd3-EBOZ at $1x10^{11}$ PU IM	MVA-EbolaZ at $1x10^{8}$ PFU IM
	1c	15	cAd3-EBO at $2x10^{10}$ PU IM	MVA-EbolaZ at $1x10^{8}$ PFU IM

	1d	15	cAd3-EBO at 2x10^{11} PU IM	MVA-EbolaZ at 1x10^{8} PFU IM
**2**		**30***		
	2a	15	cAd3-EBO at 2x10^{10} PU IM	MVA-EbolaZ at 1x10^{8} PFU IM
	2b	15	cAd3-EBO at 2x10^{11} PU IM	MVA-EbolaZ at 1x10^{8} PFU IM
**Total**		**90**		
Groups 1 and 2 were enrolled simultaneously<br>cAd3-EBO and cAd3-EBOZ were administered in 1 mL volume with needle and syringe. MVA-EbolaZ will be administered in a volume of 0.3 mL with needle and syringe.<br>In Part II, the interval of time to receive the boost will vary but will be at least 36 weeks.				

6. STUDY DURATION

Your participation in Part I took 48 weeks (almost 1 year) from the time of enrollment. Your participation in Part II will also take 48 weeks (almost 1 year). There will be 1 screening visit and 10 study visits (1 visit for vaccination and 9 visits for follow-up). However, these do not include additional appointments if you have any side effects and if the study team requests you to come to the clinic. The vaccination visit will take approximately 3-4 hours. Other appointments will take approximately 1-2 hours.

7. STUDY PROCEDURES

a) How do I join in this study?

You will have to sign this form accepting that you have read the form; all your questions have been answered satisfactorily, and agree to participate in this study.

You will qualify to take part in Part II of this study if you are a healthy, male and female volunteers who participated in Part I of this study, between ages 18 and 65 with BMI $\leq$ 40, available for a period of 48 weeks and agree to clinic staff visiting your home (as may be necessary). You must also be capable of reading English or Luganda, understand and complete this informed consent process, successfully complete an Assessment of Understanding (to assess your understanding of the information in this form), and be free of significant medical problems. This will include measuring your hemoglobin (amount of blood), your blood cells, your liver and kidney function. Females will also be required to not be pregnant and to use a birth control method for 21 days prior to vaccination and for at least 6 months after vaccination. You must also agree to have your photograph and/or fingerprint taken as proof of identity. Participants who are willing to provide consent for future use of their samples may participate in this study.

You **cannot** participate in this study if you received an investigational Ebola or Marburg vaccine other than the Ebola DNA in RV 247 or have received a different cAd3 investigational vaccine. You also cannot participate if you have a bleeding problem or disorder, history of a serious allergic reaction to any vaccines or are allergic to drugs like gentamycin, neomycin or streptomycin. You also cannot

participate if you have an autoimmune disease (where your immunity attacks other body cells) or if you are a recipient of any of the following:

- Drugs that may modify your immune system within 14 days prior to enrollment e.g. Prednisolone or dexamethasone
- Blood products within 112 days prior to enrollment.
- Any “live-attenuated” vaccine (e.g., polio) within 28 days prior to initial study vaccine administration in the present study.
- Investigational research agents or vaccine within 28 days prior to enrollment in the present study
- Drugs for treating or preventing Tuberculosis.

You also may not participate in the study if the Investigators think you may have a history of clinically significant condition(s) that may interfere with your full participation in the study or that may impair your ability to provide informed consent.

You will qualify to participate in Part II of this study if you completed at least 36 weeks of participation in Part I, and you are in general good health. You **cannot** participate in Part II if you have a reaction to antibiotics called aminoglycosides (e.g. Gentamycin, Streptomycin), or if your electrocardiogram (ECG) test result is significantly abnormal. The ECG is a painless procedure that involves placing several sticky pads attached to a machine, onto your chest. It reads the electrical activity of your heart and allows us to see if you may have a problem with your heart that will prevent you from participating in Part II of the study.

If you are unable to participate in Part II, your RV 422 Part I study participation will be complete.

It is important to remind you that to participate in this study you need to agree to home visits by the clinic staff. The clinic staff may visit your home if they are unable to reach you by phone, in order to remind you of your scheduled visit or for follow up.

b) Screening Process for Part II

After you have reviewed the study consent form and have agreed to participate, the study staff will ask you to complete an Assessment of Understanding (AOU). The AOU will help the study staff to determine how well you have understood the study and what is required for participation. You must complete 9 out of the 10 questions correctly at least once in 3 attempts. After the AOU, your medical history will be recorded and a thorough physical examination will be performed on you by a member of the study staff. You may also have your photograph and/or fingerprint re-taken as proof of identity (if necessary).

After 36 weeks in Part I, the Principal Investigator (PI) will determine if you are in good health to participate in Part II, and an ECG (described above) will be performed. In addition, you will have about 3 ½ tablespoons (54 mL) of blood taken to check for diseases such as HIV, and to check your general health. A urine pregnancy test will be required if you are female and able to become pregnant. You cannot participate in the study if you are presently pregnant. You will also be informed of potential risks of becoming pregnant during this trial.

The researchers will need to test your blood for HIV (human immunodeficiency virus), the virus that causes AIDS. The test will determine if you have antibodies against HIV. The body develops antibodies against infections. If you have antibodies to HIV, other tests will be done to determine if you really have HIV infection. Prior to this blood test, you will receive counseling about HIV, AIDS, and prevention of HIV. If you are HIV infected, you will receive additional information about HIV and will be referred to our HIV Clinic or the Infectious Disease Institute (IDI) at Mulago or any other HIV Clinic of your choice.

If the physician discovers an illness or condition that requires treatment, you will be referred to a hospital or clinic which can provide further evaluation or treatment.

You will return a few days later to review laboratory results including HIV status and review any questions or concerns you may have. If required (e.g. if you had a mild illness at the first visit), a few tests may be repeated to ascertain your health.

If you are found to be eligible for participation in this study, you will be scheduled for an appointment for the Part II vaccination visit within 28 days of the Part II screening date. It is important to remind you that as part of the qualification for this study you have agreed to possible home visits by the clinic staff, and to be identified by a document that contains your photograph.

c) Study Visits

Just like in Part I of the study, the clinic staff will observe you for at least 30 minutes after the injection. You will be asked to complete a diary card 6 hours after the vaccination (at home) and everyday for the next 7 days. This will require that you record your temperature and symptoms and look at the injection site each day. You will be provided with a thermometer to take your temperature and ruler to measure any injection-site skin changes. You will have to come to the clinic if you develop a rash or hives or a fever of 38.5˚C or higher that lasts more than 24 hours, or difficulty in your usual daily activities (such as going to work or taking care of yourself). You will be able to reach a study doctor at any time of day or night should you have any concerns.

If you have any symptoms, it may be necessary to come to the study clinic for an examination before your next scheduled visit. It is very important that you follow the instructions given to you by the clinic staff. You will also need to come to the clinic for any problem that the nurse or doctor thinks should be checked by exam, blood or other medical test.

At each visit, you will be checked for any health changes or problems since your last visit. You will be asked how you are feeling and what medications you may have taken. Blood will be drawn during clinic visits for testing of your health and your immune system.

In Part I, the amount of blood drawn varied from about 1 tablespoon (16 mL) to about 6 ½ tablespoons (97 mL), depending on the visit. You might also be asked to have laboratory tests between regular visits if needed to check your health. The total amount of blood drawn during the 48 weeks of study of Part I was just over 1 and ¼ *tumpeco mug* (675 mL).

In Part II, the amount of blood drawn will vary from about 1 tablespoon (17 mL) to about 7 ¾ tablespoons (115mL), depending on the visit. You might also be asked to have laboratory tests between

regular visits if needed to check your health. The total amount of blood drawn during the 48 weeks of participation will be about almost one and a half *tumpeco mugs* (736 mL).

No more than a total of about one *tumpeco mug* (450 mL) will be drawn over any 12-week period during the study. Female participants will also have to give a urine sample for pregnancy test at some visits. You will be informed promptly if any health concerns are identified by the tests. You should avoid donating blood for at least one year after your last vaccination.

The study team will inform you of the results from your lab tests and medical examination at the next study visit. In cases where a clinically significant abnormality is found, the investigators will notify you ASAP. If any clinically significant abnormality is detected, you will be referred for appropriate testing, treatment and care as may be required.

d) Sample collection during the study

i. **Blood and urine specimens:** The investigators will obtain blood to test for any possible side effects as well as evaluate the immune response to the vaccines. Urine collected at study visits, where no vaccine is administered, will be used for pregnancy tests for female volunteers.

ii. **HLA and Genetic tests:** Part of the blood samples for this study may be used to analyze for HLA ('Human Leukocyte Antigen'), type. HLA is a group of proteins present on the surface of all cells on the human body with an important role in the immune response to foreign organisms. For this study, an HLA test will be used to examine/ identify factors associated with response to a vaccine or other conditions. Determining HLA type is necessary to be able to perform certain research studies. We will not notify you with the results of this test unless there is a medical requirement to do so. The HLA test for this study is not a normal medical test and the test result will not be used for treatment purpose. You will be provided a separate form to consent or refuse genetic testing on your samples.

Before signing your name to store your specimen for future use, you will have an opportunity to review, ask, and discuss regarding information in the informed consent form. You can ask to limit the use of your specimens or your study data in the future, which may not be indicated in this document. After study closure, your remaining specimens will be stored for 5 years. If they are needed to be kept longer than that, the research team will inform the Institutional Review Board (Makerere University School of Public Health Research and Ethics Committee) and ask for approval to do so from them.

8. WHAT WILL HAPPEN TO MY SAMPLES AFTER THIS STUDY?

a) Sample Storage

During your participation in this study, blood samples will be collected from you as already explained. We will store residual samples in a secure central storage site (not in the clinic) in the USA or another country for future research to learn more about Ebola virus, vaccines, the immune system, and/or other medical conditions. Only residual samples from participants

who have provided consent for future use of their samples, will be stored at the end of this study. All other residual samples will be destroyed upon completion of tests for this study.

Please note that samples will only be transferred to another country after approval by the Uganda National Council of Science and Technology and your personal information will not be disclosed/ attached to these specimens (as described below).

b) **Future Studies**
You will have the opportunity to review, ask questions and provide consent (permission) for storage and use of your Part II blood samples for future unknown use, in the "Future Use Blood Sample Consent Form." **Although you may withdraw from the study at any time, the samples and data collected up to that time will be used in accordance with the protocol.**

c) **Specimen labeling**
Specimens will be stored and labeled using a numeric barcode without your name attached. Only the site-investigator team is able to connect those numeric codes and your name. Personal identification Information will be kept confidentially according to all applicable laws and regulations.

9. POSSIBLE RISKS OF THIS STUDY PARTICIPATION

This section describes the risks and restrictions that are known about the experimental vaccines. There may be additional risks related to the experimental vaccines that are currently unknown. These unknown risks could affect you or your fetus if you become pregnant. If the study investigators learn about new risks during this study, the study investigators will tell you.

Possible **risks from the injection:** temporary stinging, pain, redness, soreness, itchiness, swelling or bruising at the injection site on your arm. There is a very small chance of infection.

Possible **risks of blood drawing:** pain, bleeding, bruising, feeling lightheaded, fainting, or rarely, infection at the site where the blood is taken.

Possible **risks from genetic testing:** unintended release of information could be used by insurers or employers; discovering a gene or HLA type that suggests risk of disease for you or your family; discovering undisclosed family relationships.

Possible **risks from any vaccine:** fever, chills, rash, aches and pains, nausea, headache, dizziness, and fatigue. Some people have allergic reactions to vaccines. These types of reactions are usually greatest within the first 24 hours after vaccination and typically last 1 to 3 days. Over-the-counter medicine, such as paracetamol (panadol), will generally help relieve symptoms from vaccination and may be used.

Possible **risks of the experimental vaccines:**

Some risks may be unknown. So far, the most common complaints in the first few days after vaccination have been sore arm, headache, muscle aches and feeling tired. A few people had a fever within a day after vaccination. Over-the-counter medicine, such as panadol, may be used if you have

any symptoms from vaccination. A few people had a temporary change in a laboratory test that required additional tests.

As with any vaccine, there may be a risk of skin rash, hives, or other unknown side effects. There are currently no vaccines approved for use to protect against Ebola virus infection. Receipt of this experimental Ebola vaccine may affect your response to future vaccines against Ebola. It is unknown if you will develop an immune response, such as antibodies, after vaccination. It is unknown if your immune response would protect against infection, have no effect on protection, or increase your risk of infection. It is also unknown how long an immune response to the vaccine may last. You should continue to take all precautions against being exposed to body fluids of people who have Ebola virus infection.

***Unknown* safety risks:** There may be unknown side effects from the study vaccines - even serious or life threatening risks - that we do not yet know about. Please tell the study staff about any side effect you think you are having ASAP. This is important for your safety.

The first human clinical trial with VRC-EBOMVA079-00-VP (MVA-EbolaZ)- called VRC 208, began on April 27, 2015. As of July 6, 2015, MVA-EbolaZ has been given to 40 subjects, both as a single injection and as a boost to subjects who previously received cAd3-EBO and cAd3-EBOZ. To date, cAd3-EBO, cAd3-EBOZ, and MVA-EbolaZ have been evaluated as well tolerated and safe for further evaluation in humans.

***Possible* risks from Pregnancy:** If you are pregnant, breast-feeding or want to become pregnant during the next 24 weeks, you cannot participate. We do not know the possible effects of the study vaccine on the fetus or nursing infant. Therefore, women who are able to become pregnant must have a negative pregnancy test before the study vaccination and agree to practice adequate birth control beginning at least 21 days prior to receiving the study injection until 24 weeks after the injection. Adequate methods of birth control include: condoms, male or female, with or without a spermicide; diaphragm or cervical cap with spermicide; intrauterine device; all prescription methods (such as contraceptive pills, injections, patches and others); or a male partner who has previously undergone a vasectomy. You must notify the clinic staff immediately upon learning that you have become pregnant during this study. You must also notify the clinic if you suspect that you **might** be pregnant during this study. You will be asked to continue with the planned study follow-up visits and contacted later to learn about the outcome of any pregnancy that starts in the first 24 weeks after study vaccination.

***Other* Risks:** It is unknown if the study vaccine(s) may alter your response if you ever have an Ebola virus infection in the future.

You will be made aware of significant health effects of the vaccine(s) and serious side effects if they occur in other subjects, and will be updated during the trial as needed.

You may not donate blood at a blood bank while participating in this research study or for one year after the date of the experimental vaccine(s) injection.

10. WHAT IF THE RESEARCHERS LEARN NEW INFORMATION DURING THIS STUDY?

Results of this study or other scientific research may affect your willingness to continue to take part in

this study. During the course of the study, you will be informed of any significant new findings (either good or bad), such as changes in the risks or benefits resulting from participation in the research or new alternatives to participation that might cause you to change your mind about continuing in the study. If new information is provided to you, your consent to continue participating in this study will be re-obtained.

11. BENEFITS FROM THE STUDY PARTICIPATION

This study may be of no direct benefit to you because no one knows if the vaccines will work. However, you and others may benefit in the future from the information that will be learned from the study. The results of this study could play a role in whether the FDA will approve the vaccines for sale at some time in the future. You will not receive money or other compensation should this occur.

12. COMPENSATION FOR STUDY PARTICIPATION

Study volunteers will be compensated Ug shs 50,000 for time and inconvenience associated with each scheduled visits and Ug shs 20,000 for each unscheduled visits. Unscheduled visits will be compensated only if the Principal Investigator/ designee judge finds it necessary. Other than medical care that may be provided and other payment specifically stated in this form, there is no other compensation available for you taking part in this study.

13. PERSONAL INFORMATION CONFIDENTIALITY

The Principal Investigator at this clinic, Dr. Hannah Kibuuka, will maintain research records of your taking part in this study.

All study volunteers will receive an SID (Study Identification Digit). An SID is a unique number assigned to each participant, known only to the study team at the clinic and used to ensure the confidentiality of research information. All your study documents, samples and test results will not bear your name but will have your SID, the date, study number, group number and study visit number. Personal identifying information like your name and age collected at the time of enrollment will be stored in a lockable cabinet to which only designated study team members will have access. These steps will ensure confidentiality of your personal information and minimize the chances of it becoming known to others.

Clinical and research records may be reviewed by representatives of the U.S. Army Medical Research and Materiel Command (USAMRMC), U.S. Military HIV Research Program (USMHRP), The U.S. National Institutes of Allergy and Infection Diseases (NIAID), Walter Reed Army Institute of Research Institutional Review Board, Makerere University School of Public health IRB, the Uganda National Drug Authority, the Uganda National Council of Science and Technology, U.S. Food and Drug Administration, The U.S. Health and Human Services (HHS), Office for Human Research Protections (OHRP), the local Ministry of Health, the providers of the devices, and other regulatory agencies as part of their responsibilities for ensuring the protection of research volunteers. Representatives of all the above are bound by rules of confidentiality not to reveal your identity to others.

Complete confidentiality cannot be promised but every effort will be made to keep the records as confidential as possible within the limits of the law. All data and medical information obtained about you as an individual will be considered important and held in confidence.

Research and clinical information relating to you will be shared with other investigators and the scientific community through presentation or publication; however, you will not be identified by name or other personal information that could be used to identify you.

Additionally, it is the policy of the USAMRMC that data sheets are to be completed on all study volunteers participating in research for entry into this Command's Volunteer Registry Data Base. The information to be entered into this confidential database includes your name, study number, date of birth, contact information, address, study title and dates participating in study, any and adverse events related to the vaccine and details of which study products you received. The intent of the data base is two-fold: first, to readily answer questions concerning an individual's participation in research sponsored by USAMRMC; and second, to ensure that the USAMRMC can exercise its obligation to ensure research study volunteers are adequately warned (duty to warn) of risks and to provide new information as it becomes available. The information will be stored at USAMRMC for a minimum of 75 years. Please note that your name and study number will be stored separately from the USAMRMC volunteer registry database.

General clinical trial information will be kept at the database at the National Medical Library in the United States of America/the National Institutes of Health of the United States of America on http://www.clinicaltrials.gov. This website will not include information that can identify you. At most, the Web site will include a summary of the results.

14. SICKNESS OR INJURY AS A RESULT OF STUDY PARTICIPATION

If you get sick or injured due to the vaccination of this study, you will receive appropriate medical treatment and care as provided with two types of coverage by a limited fund and a clinical trials medical insurance policy that will be obtained by MHRP (HJF). While we anticipate the combination of the set-aside fund and the insurance policy is more than enough to pay for the costs associated with this study, there is a limit to the amount of coverage available.

The study team is responsible for the cost without using any personal health care package which belongs to the volunteer. MUWRP will pay costs up to the limit from set aside finds or through the insurance. However, you will not get any other compensation. You should discuss this thoroughly with the Principal Investigator or site clinicians before making a decision to participate in this study. If you have any questions about study-related sickness or injury, you can contact the following person:

Dr. Hannah Kibuuka
Makerere University Walter Reed Project
[redacted]

15. ENDING STUDY PARTICIPATION

You can choose not to participate or withdraw from the study at any time without any consequence to you. However, before withdrawing from participation, the study team will request to perform a medical examination, blood tests to evaluate your health status before your participation has come to an end.

If you would like to withdraw from this study, please contact the Principal Investigators or the study staff mentioned previously. You will not lose any legal rights, including the rights for medical treatment and others if you withdraw from this study.

Although you may be willing to participate in the study, the investigators may not give you the vaccinations if any of the following situations occur:

- Study is stopped.
- Study sponsors, the Institutional Review Board, or the US FDA request to terminate the study for unexpected reasons.
- You are unable to comply with the study requirement.
- You are not willing to have blood drawn although you are still willing to participate in other processes.
- You have a medical problem where continuing to be in the study would be harmful to you.
- Other incidents occurred and may be harmful to you if you continue being the study volunteer.

16. ALTERNATIVES

This study is not designed to treat any disease and no alternative currently exists. You may choose to not participate.

17. CONFLICT OF INTEREST STATEMENT

The NIH, including members of the VRC scientific staff, developed the experimental Ebola vaccine being used in this research study. The results of this study could play a role in whether the FDA will approve the vaccine for sale at some time in the future. If approved, the future sale of the vaccine could lead to payments to the NIH and to some of the NIH/VRC scientists. By US Law, government scientists are required to receive such payment for their inventions. Other participating investigators do not have a conflict of interest as a result of study participation. You will not receive money or other compensation should this occur. Please discuss with your study doctor any questions you may have about these issues. There is no conflict of interest with your doctors at this research site.

15.IF YOU NEED MORE INFORMATION OR HAVE ADDITIONAL QUESTIONS

If you have any question about this study or if you have any problems, you can contact the Principal Investigator Dr. Hannah Kibuuka at the research clinic on [redacted] or Dr. Salim Wakabi [redacted]

If you have any question and need to ask about your rights or you do not get appropriate treatment and care for sickness or injury which occur as a direct result of taking part in this study or the investigator does not treat you fairly in accordance with what is described in this consent form, you may make a complaint to the following bodies:

**For information on**	
Regulatory questions	Dr. Julius Ecuru, at Uganda National Council of Science and Technology, [redacted]
Human subject protection questions	Dr. Suzanne Kiwanuka at the Research and Ethics Committee, Makerere University School of Public Health, Mulago Hospital Complex [redacted]

Please keep a copy of this document in case you want to read it again.

15. STUDY VOLUNTEER STATEMENT

I have been asked to take part in RV 422 PART I & PART II "A Phase 1B, Open-Label, Clinical Trial to Evaluate the Safety, Tolerability and Immunogenicity of the Investigational Ebola Vaccines VRC-EBOADC069-00-VP (cAd3-EBO) and VRC-EBOADC076-00-VP (cAd3-EBOZ), and VRC-EBOMVA079-00-VP (MVA-EbolaZ), in Healthy Adults in Kampala, Uganda."

The principal investigator Dr. Hannah Kibuuka or her representative has explained the significance of the testing, the duration of the study, the testing that I will undergo, the methods to be used, and the risks and dangers of participation. I have been given a chance to ask questions about this research study. All questions were answered to my satisfaction. If I have other questions about this research, I can ask: Dr. Hannah Kibuuka or Dr. Salim Wakabi at the research clinic on [redacted]

I am signing below to indicate I wish to take part in this study, and my consent to follow the requirements of the study as much as possible. I will do my best to follow the recommendations of the study team, and I will report all problems occurring from this study to the study team. It has been explained to me that I can quit this study at any time, and I will not lose any benefits nor will I receive any penalty. If I decide to quit this study, I may be examined before leaving the study to ensure my good health. The medical care that I could receive as a result of sickness from being a part of this study have been explained to me and I have been offered a signed copy of this consent form.

I agree to participate in this study.

______________________________ __________ __________
SIGNATURE OF VOLUNTEER DATE TIME

____
PRINT NAME OF VOLUNTEER

______________________________ __________ __________
SIGNATURE OF PERSON ADMINISTERING CONSENT DATE TIME

____
PRINTED NAME OF PERSON ADMINISTERING CONSENT

Appendix 2b: INFORMED CONSENT FOR GENETIC TESTING

**Makerere University Walter Reed Project
in Collaboration with the
U.S. Military HIV Research Program (MHRP)
and the
U.S. National Institutes of Health (NIH)**

RV 422 PART I & PART II

INFORMED CONSENT FOR GENETIC TESTING

Title: "A Phase 1b, Open-Label, Clinical Trial to Evaluate the Safety, Tolerability and Immunogenicity of the Investigational Ebola Vaccines, VRC-EBOADC069-00-VP (cAd3-EBO) and VRC-EBOADC076-00-VP (cAd3-EBOZ), and VRC-EBOMVA079-00VP (MVA-EbolaZ), in Healthy Adults in Kampala, Uganda"

Uganda Site Principal Investigator
Hannah Kibuuka, M.B.Ch.B., M. MED., M.P.H.
Makerere University-Walter Reed Project
[redacted]

As we told you in the main informed consent form for this study and in the consent form for storage of samples for future use, we will do some testing of your blood. Some of the testing that we will do will be genetic testing. This consent form tells you everything we know now about genetic testing using your blood samples.

You can decide whether or not to let us use your blood for genetic tests. Your decision does not affect your participation in the study or any care you receive at this clinic. If you decide to allow us to use your blood samples for genetic tests, we will ask you to sign this form. You will get a copy to keep.

1. Your blood sample is a potential source of genetic information.

Researchers are able to measure how the immune system responds by looking at blood. We will try to understand why Ebola and other diseases affected by Ebola progress differently in some people and why some people are more likely to become infected than others. We know that sometimes genes, passed down from your parents, can be important to a person's immune response to Ebola. Because of this, we would like to do genetic testing on your blood samples. We will only perform genetic testing to learn more about how the immune system responds to Ebola and to other diseases affected by Ebola.

HLA and Genetic Testing: Some of the blood drawn from you, as part of this study will be used for a test called HLA type. HLA stands for 'Human leukocyte Antigen', a group of proteins present on the surface of all cells on the human body and help the body's immune system respond to foreign, harmful substances. For research, HLA testing is used to try to identify factors associated with response to a vaccine, progression of a disease or related conditions. Determining HLA type is necessary to be able to perform certain research studies.

We will **not** notify you of the results of any genetic test results unless it is known from current medical practices that medical care is needed and possible. The genetic research tests we plan to

conduct are not currently used in medical practice and the results of such tests are not used to make health care decisions.

2. Your samples used for genetic testing may be shipped to the USA or other regional laboratories.

In order to complete the genetic testing on your blood samples, they may be shipped and stored in the United States or other laboratories. There is no time limit on how long your samples will be stored.

3. Your privacy will be protected.

We will protect your privacy with any genetic testing of your blood samples, just like we do with all research information from you during the study. The blood samples will not be labeled with your name. Instead, they will have your study ID number only. If your samples are sent outside Uganda, the study ID number stays with them. Your genetic test results will only be connected to you by the study number, known only to the study team, and not by your name or other personal information.

4. There may be no benefit to you if you allow us to use your samples for genetic testing.

The researchers do not plan, in general, to contact you or your health care provider with results from the genetic testing using your blood. This is because the use of the samples is for research not for evaluation of your health. However, if the researchers decide that a test result would provide important information for your health, we will try to contact you. If you want this information, tell the clinic staff. Always let the study clinic staffs know if you change your address and/or phone number.

Your samples may contribute to a new invention or discovery. There is no plan for you to share in any money or other benefits resulting from this invention or discovery.

5. There are few risks related to genetic testing of your samples.

Risk of genetic tests and HLA testing: The greatest risk associated with genetic testing is to your privacy. Genetic test results can be used to provide information about how susceptible you are to certain diseases. Used inappropriately, this information could be discriminatory (for example, by insurance companies). HLA typing can also be used to determine who the true parent of a child is (if compared to the child's HLA type). However, the risk of this happening is extremely low, because your results will not be part of your medical records and will not be provided to the clinic.

The blood samples that you provide will only be used to provide study investigators information about your immune system. The results will be coded to protect your identity. Your HLA (and other genetic tests) can only be connected to you by the coded study number and not by your name or other personal information. Neither you nor your doctor will be given the results of the tests.

6. For more information:

If you have questions about the use of your samples for genetic testing, contact Dr. Hannah Kibuuka at Makerere University- Walter Reed Project, [redacted]

If you have a problem that you think may be related to the use of your samples for genetic testing, or if you want to withdraw your consent, contact Salim Wakabi at Makerere University- Walter Reed Project, [redacted]

If you have questions about your rights as a research participant, or problems or concerns about how you are being treated in this study, you may contact, Dr. Suzanne Kiwanuka of the Makerere University School Public Health Research and Ethics Committee, the IRB for this study, o[redacted]

Once you have read this form, and have had all your questions satisfactorily answered, please check whether or not you consent to have your blood samples used for genetic testing, and sign the consent form

☐I allow you to use my samples for genetic testing.

☐I do not allow you to do genetic testing on my samples.

__________________________________ __________ __________

SIGNATURE OF VOLUNTEER DATE TIME

____

PRINT NAME OF VOLUNTEER

__________________________________ __________ __________

SIGNATURE OF PERSON ADMINISTERING CONSENT DATE TIME

____

PRINT NAME OF PERSON ADMINISTERING CONSENT

Appendix 2c: WITHDRAWAL OF CONSENT FOR SAMPLE STORAGE

Makerere University Walter Reed Project
in Collaboration with the U.S. Military HIV Research Program
and the U.S. National Institutes of Health (NIH)

RV 422 PART I & PART II

WITHDRAWAL OF CONSENT FOR SAMPLE STORAGE

Title: "A Phase 1b, Open-Label, Clinical Trial to Evaluate the Safety, Tolerability and Immunogenicity of the Investigational Ebola Vaccines, VRC-EBOADC069-00-VP (cAd3-EBO) and VRC-EBOADC076-00-VP (cAd3-EBOZ), and VRC-EBOMVA079-00VP (MVA-EbolaZ), in Healthy Adults in Kampala, Uganda"

Principal Investigator: Dr. Hannah Kibuuka

Volunteer Statement of withdraw of consent to have samples stored for future testing:

I ____________________________________ withdraw my consent to have my samples stored for future use. I do not want to donate blood samples for storage and future use. However, I would still like to continue taking part in the main study. It has been explained to me that when I sign at the bottom of this form, my samples will be used for all the tests specified for this present study, but no blood will be stored for future use.

It has been explained to me that withdrawing my consent to have my samples stored for future use will not make any difference to the care I am receiving now or in the future, or to any benefits that I am entitled to.

I have been given a chance to ask all the questions that I have about withdrawing my consent to have my samples stored. All of my questions were answered to my satisfaction. I was offered a signed copy of this consent.

_____________________________________ ___________ __________
SIGNATURE OF VOLUNTEER DATE TIME

____
PRINTED NAME OF VOLUNTEER

____
SIGNATURE OF PERSON ADMINISTERING CONSENT DATE

____
PRINTED NAME OF PERSON ADMINISTERING CONSENT

Appendix 2d: INFORMED CONSENT FOR FUTURE USE OF STORED SPECIMENS

Makerere University Walter Reed Project
In Collaboration with the
U.S. Military HIV Research Program
and the
U.S. National Institutes of Health (NIH)

RV 422 PART I & PART II

INFORMED CONSENT FOR FUTURE USE OF STORED SPECIMENS

Title: "A Phase 1b, Open-Label, Clinical Trial to Evaluate the Safety, Tolerability and Immunogenicity of the Investigational Ebola Vaccines, VRC-EBOADC069-00-VP (cAd3-EBO) and VRC-EBOADC076-00-VP (cAd3-EBOZ), and VRC-EBOMVA079-00VP (MVA-EbolaZ), in Healthy Adults in Kampala, Uganda"

Principal Investigator: Dr. Hannah Kibuuka

During this study, you will be asked to provide blood. These blood samples will be stored for as long as possible and will be used according to your decision below. Some of the blood samples may be stored in Uganda or the United States (U.S.) for testing how your body fights Ebola infection and other future studies that we do not know about at this time. Some of the tests that may be conducted on your stored samples may not be developed as yet, so the investigators can not tell you all the tests that may be performed in the future.

There is a chance that the blood samples you are donating under this study may be used in other research studies and may have some commercial value. Your samples will not be sold or used directly to produce commercial products.

Should your donated sample(s) lead to the development of a commercial product, the study sponsor and inventor will own it and may take action to patent and license the product. Neither the sponsor nor the inventor intend to provide you with any compensation for your blood samples provided in this study, nor for any future value that the sample you have given may be found to have.

Use of stored samples for future studies will be subject to an IRB (Institutional Review Board) in the U.S. and Uganda as well as the Uganda National Council for Science and Technology (UNCST), for review and approval.

The blood samples will not be stored with any personal information, which will connect you to your blood sample. Your stored samples will be labeled by a code (such as a number) that only the study team can link to you. All personal information will be stored by the study investigator at the study site. Any identifying information about you will be kept confidential to the extent permitted by law.

You will not receive the results of future studies involving your stored blood samples.

1. Future research on your samples will be related to Ebola or vaccines.

Researchers are able to measure how the immune system responds by looking at blood samples. We will try to understand why Ebola disease progresses differently in some people. As new methods (or ways) of measuring the body's immune response to Ebola are made in the laboratory, we would like to test these methods on the samples we have already collected from you. We know that sometimes genes, passed down from your parents, can be important to a person's immune response to Ebola. Because of this, we may do genetic testing on your stored samples. We may use methods that have not been developed yet, so we cannot describe them to you now. We will only use your stored samples to learn more about how the immune system responds to Ebola and how vaccines can prevent Ebola infection.

2. Your samples used for future research may be shipped to the USA or to regional laboratories.

Your samples will be stored in a secure central storage site (not in the clinic) in the USA, or another country. The samples will not be labeled with your name but your study code/ PIN (Participant Identification Number). Some of your samples will be stored and used for research in Uganda and some will be shipped out of the country to laboratories chosen to carry out research tests. There is no time limit on how long your samples will be stored.

3. Your privacy will be protected.

We will protect your privacy with any future research testing of your samples, just like we do with all research information from you during the main study. The samples will not be labeled with your name. Instead, they will have your study code. After this study ends, when the samples are requested for future research, the study code stays with them, or in some cases, it is removed before the samples are sent to be used, if this information is not necessary for the study.

4. An Institutional Review Board/Independent Ethics Committee will review any future research on your samples.

An Institutional Review Board/Independent Ethics Committee, which is responsible for overseeing the safety, welfare and rights of research participants, must review and approve each research study that intends to use your samples in future studies.

5. There may be no benefit to you if you allow us to store your samples for future research.

The researchers do not plan, in general, to contact you or your health care provider with results from future studies using your samples. This is because the use of the samples is for research and not for evaluation of your health. However, if the researchers decide that a test result would provide important information for your health, we will try to contact you. If you want this information, tell the clinic staff. Always let the study clinic staff know if you change your address and/or phone number.

Your samples may contribute to a new invention or discovery. There is no plan for you to share any money or other benefits resulting from this invention or discovery.

6. There are few risks related to storing your samples.

When tests are done on the stored samples there is a small but possible risk to your privacy. It is possible that if others found out information about you that is learned from tests (such as information

about your genes) it could cause you problems with your family (e.g., having a family member learn about a disease that may be passed on in families or learning who is the true parent of a child) or problems getting a job or insurance. The risk of this happening is extremely low, because your results will not be a part of your medical record and will not be given to the clinic. Also, it is possible that your participant ID could be removed from the samples. If your participant ID number is removed from any samples, we will not be able to link that sample to you.

7. You can agree now to let us use your samples for future testing and still change your mind later.

If you agree now and decide later that you do not want us to use your samples for future research, please tell us. We will ask the storage facility to destroy any remaining samples that still have your participant ID on them so that they cannot be used for future research.

8. For more information:

If you have questions about the use of your samples for future research, contact Dr. Hannah Kibuuka [redacted]

If you have a problem that you think may be related to the use of your samples for future research, or if you want to withdraw your consent, contact Dr. Salim Wakabi [redacted]

If you have questions about your rights as a research participant, or problems or concerns about how you are being treated in this study, you may contact Dr. Suzanne Kiwanuka at the Research and Ethics Committee, Makerere University School of Public Health, Mulago Hospital Complex [redacted]

If you have read this form, and had all of your questions answered, please sign or place your thumbprint in the space provided below to declare that you consent to have your samples stored for future use.

☐ I allow you to store my samples for future testing which may include genetic testing.

☐ I **do not** allow you to store my samples for future testing.

Participant's name (print)	Participant's signature	Date	Time
Study staff conducting consent discussion (print)	Study staff signature	Date	Time

APPENDIX 3. ASSESSMENT OF UNDERSTANDING

**ASSESSMENT OF UNDERSTANDING – Part I**<br>**Protocol RV 422 PART I & PART II**	True	False
1. The main purpose of the study is to see if the experimental vaccines are safe.	■	☐
2. The vaccine in this study will definitely protect me against Ebola.	☐	■
3. I will be able to choose which vaccine I receive.	☐	■
4. Some participants will receive placebo instead of vaccine.	☐	■
5. I may leave the study at any time.	■	☐
6. Participation in the study will last for approximately 48 weeks (11 months).	■	☐
7. During participation in this study, samples of my blood will be collected and stored in a research laboratory and may be used for future research studies.	■	☐
8. Women participating in the study must use effective birth control 21 days prior to the first vaccine through 24 weeks after the first vaccination.	■	☐
9. There is a possibility that I can become infected with Ebola from the study vaccine.	☐	■
10. I will be asked to keep a detailed diary of possible side effects for seven days after vaccination.	■	☐

ASSESSMENT OF UNDERSTANDING – Part II<br>Protocol RV 422 PART I & PART II	True	False
1. I will be asked to keep a detailed diary of possible side effects for seven days after vaccination.	☐	☐
2. The vaccine in this study will definitely protect me against Ebola.	☐	☐
3. I can participate in Part II of the study if I received a different experimental Ebola DNA that was not RV 247 or RV 422 PART I.	☐	☐
4. In Part II of the study I will receive the MVA-EbolaZ vaccine.	☐	☐
5. The injection will be given using a needle and syringe in the upper arm muscle.	☐	☐
6. Participation in Part II of the study will last for approximately 48 weeks (11 months).	☐	☐
7. During participation in this study, samples of my blood will be collected and stored in a research laboratory and may be used for future research studies.	☐	☐
8. I may leave the study at any time.	☐	☐
9. I will receive a placebo in this study.	☐	☐
10. If I am unable to participate in Part II, my RV 422 PART I participation will be complete.	☐	☐

APPENDIX 4. BRIEFING SLIDES

Join Hands with Makerere University-Walter Reed Project Scientists to Develop an Ebola Vaccine.

A Presentation By:

Makerere University Walter Reed Project

PURPOSE OF THIS PRESENTATION

To provide you with detailed information about the Ebola vaccine study, the study visits, procedures, and risks and benefits associated with the study.

What is a Vaccine?

- A vaccine is a medical product used to try to create resistance within the body to disease and to prevent infection.

What Vaccines are Being Tested?

- The investigational Ebola vaccines in this study are called VRC-EBOADC069-00-VP and VRC-EBOADC076-00-VP are referred to as "cAd3-EBO" and "cAd3-EBOZ".
- These vaccines were developed by the US National Institute of Health (NIH) and the Vaccine Research Center (VRC).

The Objectives of this Research Are:

- To evaluate the safety of the Ebola vaccines
- To evaluate the immune response of Ebola vaccines.
  - Immune responses are how your body recognizes and defends itself against bacteria, viruses, and substances that appear harmful to the body.

Who Can Take Part in The Study?

- Men and Women ages 18-65
- Available for study visits for up to one year
- Able to read and provide informed consent
- In general good health
- Willing to undergo HIV testing and receive results

RV 422 Briefing Session, Ver 1.1 14 Nov14 7

How is Informed Consent Obtained?

- Taking part in the study is completely voluntary.
- Volunteers are given all necessary information about the vaccines and the study.
- Adequate time is given to ask questions.
- A volunteer signs the form to agree to take part in the study.

RV 422 Briefing Session, Ver 1.1 14 Nov14 8

You CANNOT take part in this study if:

- You are pregnant or breast feeding.
- You plan to get pregnant within 6 months after the vaccination.
  - Willing to receive family planning counseling
- You are not in good general health or have certain health conditions at the judgment of the clinician.

RV 422 Briefing Session, Ver 1.1 14 Nov14 9

The Study

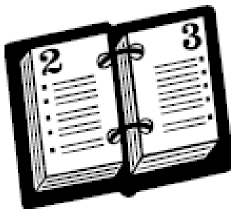

RV 422 Briefing Session, Ver 1.1 14 Nov14 10

The Study

- Phase Ib open-label clinical trial
- Sponsored by the US NIH/NIAID in collaboration with the MHRP
- 90 Participants from Kampala, Uganda

RV 422 Briefing Session, Ver 1.1 14 Nov14 11

The Study Cont.

	RV 422 Study Schema		
Group	Sub-Group	Subjects	Day 0
1		60	
	1a	15	cAd3-EBOZ at 1x10^{10} PU IM
	1b	15	cAd3-EBOZ at 1x10^{11} PU IM
	1c	15	cAd3-EBO at 2x10^{10} PU IM
	1d	15	cAd3-EBO at 2x10^{11} PU IM
2		30*	
	2a	15	cAd3-EBO at 2x10^{10} PU IM
	2b	15	cAd3-EBO at 2x10^{11} PU IM
Total		90	

Groups 1 and 2 will be enrolled simultaneously
Injections administered in 1 mL volume with needle and syringe.
* If less than 30 subjects are enrolled into Group 2, the remaining enrollment slots may be allocated to Group 1.

RV 422 Briefing Session, Ver 1.1 14 Nov14 12

If you agree to participate in the study

- You will review and voluntarily sign a consent form. Your understanding of the study will be determined by you completing and passing the Assessment of Understanding.
- You will have an initial medical history and physical exam with laboratory testing of blood samples to test your general health and include testing for HIV and test urine for pregnancy.

Study Procedures

- If you are healthy and do not have any condition which prevents you from taking part in the study, based upon the screening visit, you will be asked to return to the clinic for a review of your laboratory results and HIV results.
- If the results of your screening medical evaluation identify a problem which prevents you from participating in the study, you will be referred to a health care provider, if needed.

Study Procedures

- If you are eligible you will be scheduled for your vaccination
- The vaccination will be given into the upper arm
  - Group 1 and Group 2 will receive a 1mL dose of the experimental Ebola vaccine.

RV422 Protocol Schedule of Visits

Screening Visit I	Screening Visit II	Vaccination Visit	Follow-up Visits
Labs/H&P done 56 days or less prior to vaccination	Labs/H&P done 56 days or less prior to vaccination	Day 0	Day 3, 7 & 14, Visit 3, 4, 5, 6, 7
Information Session<br>A of U<br>Sign Consent<br>Paperwork<br>1)Media<br>2)Contact info<br>3)etc<br>Physical Exam<br>Blood	Lab results referral for f/u care<br>Blood Redraw?<br>Volunteer Question For team<br>Medical History Questions from team<br>Schedule vaccination appt.<br>Verification of how to contact	Blood<br>Injection (Vaccine)	Blood<br>Physical Exam<br>Medical History

Study Procedures

- Should you develop any symptoms that are of concern to you or to the study team, you will be asked to return to the clinic.

Study Procedures

- At every visit you will have blood drawn from a vein in your arm in a volume of about 1 to 6 ½ tablespoons (19 to 97 milliliters).
- Overall, the amount of blood that you will give is equivalent to donating blood to the blood bank twice in the same year.

Vaccination Days

- On each vaccination day, you will be required to remain at the clinic for 30-60 minutes after vaccination
- You will be taken home after the vaccination so that we know how to contact you if we are unable to reach you by phone.

RV 422 Briefing Session, Ver 1.1 14 Nov14 19

Post Vaccination

- You will return to the clinic 3 days after the vaccination. Blood will be collected for routine laboratory safety studies during this visit.
- You will be asked to complete a diary card at home for 6 hours after the vaccination and the next seven (7) days.

RV 422 Briefing Session, Ver 1.1 14 Nov14 20

Follow-Up Visits

- After vaccination, you will have 8 clinic follow-up visits.
- During each visit, the doctor will perform a brief medical history and physical exam.
- Blood will be collected for routine laboratory safety studies and to identify the body's responses to the vaccine.

RV 422 Briefing Session, Ver 1.1 14 Nov14 21

Your Rights as a Study Volunteer

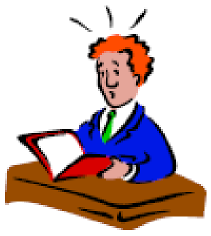

RV 422 Briefing Session, Ver 1.1 14 Nov14 22

CONFIDENTIALITY

- All volunteers will be assigned study numbers that are known only to the investigators.
- All research samples will be identified by study numbers.

RV 422 Briefing Session, Ver 1.1 14 Nov14 23

Your Vaccinations May be Terminated

- If you become infected with HIV.
- If your health conditions or other conditions occur that might be dangerous or harmful to you or your health.
- If you become pregnant.
- If you do not comply with study instructions.
- If the Sponsor or the IRB cancels the study.

RV 422 Briefing Session, Ver 1.1 14 Nov14 24

COMPENSATION

- For taking part in this study you will receive 50,000 Ugandan shillings after each scheduled visit to compensate you for your time, transport and inconveniences and 20,000 Ugandan shillings for visits to address safety concerns.

RV 422 Briefing Session, Ver 1.1 14 Nov14 25

MEDICAL CARE FOR INJURY OR ILLNESS

- Should you be injured as a direct result of taking part in this research project, you will be provided medical care, at no cost to you for that injury
- You will not receive any injury compensation, only medical care.
- You should discuss this issue thoroughly with the principal investigator before you enroll in this study

Dr. Hannah Kibuuka [redacted]

RV 422 Briefing Session, Ver 1.1 14 Nov14 26

RISKS, HAZARDS AND DISCOMFORTS

- You may get some side effects after vaccination. These side effects can also occur with other vaccines.
- Most side effects usually do not last very long and do not require treatment.
- You may have pain, redness, swelling and soreness in the area of the injection, similar to what may happen after any injection.
- You could also have an allergic reaction including hives, rash, fever, chills, difficulty breathing, abdominal pain, diarrhea, aches and pains (including joint pain and swelling), nausea, headache and fatigue.

RV 422 Briefing Session, Ver 1.1 14 Nov14 27

RISKS, HAZARDS, AND DISCOMFORTS

- Blood drawing may cause pain and bruising at the blood-drawing site, light-headedness or fainting and rarely, infection at the site where blood is drawn.
- Receiving the vaccines do NOT mean you are protected against Ebola and you SHOULD continue to follow all recommended precautions against Ebola.

RV 422 Briefing Session, Ver 1.1 14 Nov14 28

UNFORESEEABLE RISKS TO UNBORN CHILD

- To avoid becoming pregnant;
  - You should either abstain from sexual relations
  - Practice a reliable method of contraception
- Should you become pregnant while enrolled in this study, your vaccination will be discontinued.
  - You should understand that there is an unknown risk to an unborn baby.
  - You will be monitored only for safety to you and your infant.
- If you are capable of becoming pregnant, you will be given a pregnancy test prior to starting the study and prior to the injection with the vaccine.

RV 422 Briefing Session, Ver 1.1 14 Nov14 29

SAFEGUARDS

- You will be closely monitored by the study team.
- Blood studies will be conducted regularly throughout the study in order to detect any unforeseen or adverse reactions.
- You will be asked to return to the clinic for lab abnormalities or other concerns.

RV 422 Briefing Session, Ver 1.1 14 Nov14 30

BENEFITS

- The potential benefits to you for taking part in the study are unknown.
  - We cannot and do not guarantee that you will directly benefit if you take part in this study.
- However, your participation in this study will provide important information about the safety and usefulness of this and similar vaccines.

RV 422 Briefing Session, Ver 1.1 14 Nov14 31

QUESTIONS.........CONCERNS

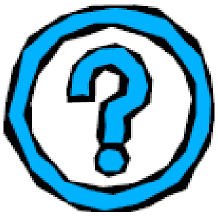

RV 422 Briefing Session, Ver 1.1 14 Nov14 32

A Phase 1B, Open-Label, Clinical Trial to Evaluate the Safety, Tolerability and Immunogenicity of the Ebola Chimpanzee Adenovirus Vector Vaccines, VRC-EBOADC069-00-VP (cAd3-EBO) and VRC-EBOADC076-00-VP (cAd3-EBOZ), in Healthy Adults in Kampala, Uganda

Study Location: MUWRP Clinic

Contact: Dr. Hannah Kibuuka

[redacted]

Website: www.muwrp.org

RV 422 Briefing Session, Ver 1.1 14 Nov14 33

APPENDIX 5. GRADING SEVERITY OF ADVERSE EVENTS

FDA Guidance for Industry (September 2007): "Toxicity Grading Scale for Healthy Adult and Adolescent Volunteers Enrolled in Preventive Vaccine Clinical Trials"

Modification to the FDA AE Grading Table
Individuals of African descent have lower neutrophil counts than individuals of other ethnicities. A study evaluating the reasons for ineligibility in phase I and II HIV vaccine trials in East Africa found that approximately one third of subjects were excluded because of neutropenia. The phase I and II studies used normal ranges from U.S. populations, not from African populations. If neutrophil ranges from East/South Africa populations were used instead, over one half of the subjects excluded because of hematologic abnormalities could have been included (ref.). Since this study will be conducted in part in East Africa, we will use the normal neutrophil range from East/South Africa (1.0 – 5.3 x 10^3 cells/ul, (ref.) for subjects in Uganda or other subjects of African descent. Grades for absolute neutrophil count in these subjects will be as follows:

	Grade 1	Grade 2	Grade 3	Grade 4
Absolute Neutrophil Count (cells/ul)	750 – 999	500 – 749	250 – 499	< 250

For other subjects, the usual FDA AE grading scale will be used as follows:

	Grade 1	Grade 2	Grade 3	Grade 4
Absolute Neutrophil Count (cells/ul)	–1,500 – 2,000	1,000-1,499	500 - 999	< 500

Assessment of Causality Relationship of an Adverse Event (AE) to Study Vaccine:

The relationship between an AE and the vaccine will be assessed by the investigator on the basis of his or her clinical judgment and the definitions below.

- **Definitely Related**. The AE and administration of study agent are related in time, and a direct association can be demonstrated.
- **Probably Related**. The AE and administration of study agent are reasonably related in time, and the AE is more likely explained by study agent than other causes.
- **Possibly Related**. The AE and administration of study agent are reasonably related in time, but the AE can be explained equally well by causes other than study agent.
- **Not Related**. There is not a reasonable possibility that the AE is related to the study agent.

For purposes of preparing data reports in which AE attributions are limited to "**Related**" or "**Not Related**", in this protocol, the "Definitely, Probably and Possibly" attributions will be mapped to the "Related" category. The definitions that apply when these two categories alone are used are as follows:

- **Related** – There is a reasonable possibility that the AE may be related to the study agent.
- **Not Related** – There is not a reasonable possibility that the AE is related to the study agent.

Grading the Severity of Adverse Events:
The FDA Guidance for Industry (September 2007): "Toxicity Grading Scale for Healthy Adult and Adolescent Volunteers Enrolled in Preventive Vaccine Clinical Trials" is the basis for the severity grading of adverse events in this protocol. Several modifications were made to the table as follows:

- “Emergency room visit” is not automatically considered a life-threatening event; these words have been removed from any “grade 4’ definition where they appear in the table copied from the guidance document.
- Any laboratory value shown as a “graded” value in the table that is within the institutional normal range will not be severity graded or recorded as an adverse event.
- Severity grading for hemoglobin decrease on the basis of the magnitude of decrease from baseline is not applicable at the grade 1 level; only absolute hemoglobin will be used to define grade 1 decrease. Increases in hemoglobin are AEs only for values above the upper limit of normal and are graded by the systemic illness clinical criteria.
- Severity grading definition for Grade 4 local reaction to injectable product (Erythema/Redness and Induration/Swelling) included added text “requiring medical attention”.
- 1 X ULN was removed from the definition for PT increase.
- Severity grading definition for hypotension includes added clarifications such that an asymptomatic low blood pressure reading is not an adverse event.
- Severity grading for neutropenia is provided for subjects of African descent based on normal ranges observed.

When not otherwise specified in the table, the following guidance will be used to assign a severity grade:

Grade 1 (Mild): No effect on activities of daily living
Grade 2 (Moderate): Some interference with activity not requiring medical intervention
Grade 3 (Severe): Prevents daily activity and requires medical intervention
Grade 4 (Life-threatening): Hospitalization; immediate medical intervention or therapy required to prevent death.
Grade 5 (Death): Death is assigned a Grade 5 severity.
Only the single adverse event that is assessed as the primary cause of death should be assigned “grade 5” severity.

Toxicity Grading Scale for Healthy Adult and Adolescent Volunteers Enrolled in Preventive Vaccine Clinical Trials

Modified from FDA Guidance - September 2007

A. Tables for Clinical Abnormalities

Local Reaction to Injectable Product	Mild (Grade 1)	Moderate (Grade 2)	Severe (Grade 3)	Potentially Life Threatening (Grade 4)
Pain	Does not interfere with activity	Repeated use of non-narcotic pain reliever > 24 hours or interferes with activity	Any use of narcotic pain reliever or prevents daily activity	Hospitalization
Tenderness	Mild discomfort to touch	Discomfort with movement	Significant discomfort at rest	Hospitalization
[1]Erythema/Redness	2.5 – 5 cm	5.1 – 10 cm	> 10 cm	Necrosis or exfoliative dermatitis requiring medical attention
[2] Induration/Swelling	2.5 – 5 cm and does not interfere with activity	5.1 – 10 cm or interferes with activity	> 10 cm or prevents daily activity	Necrosis requiring medical attention
**[3] Vital Signs**	**Mild (Grade 1)**	**Moderate (Grade 2)**	**Severe (Grade 3)**	**Potentially Life Threatening (Grade 4)**
[4] Fever (°C)<br>(°F)	38.0 – 38.4<br>100.4 – 101.1	38.5 – 38.9<br>101.2 – 102.0	39.0 – 40<br>102.1 – 104	> 40<br>> 104
Tachycardia - beats per minute	101 – 115	116 – 130	> 130	Hospitalization for arrhythmia
[5] Bradycardia - beats per Minute	50 – 54	45 – 49	< 45	Hospitalization for arrhythmia
Hypertension (systolic) - mm Hg	141 – 150	151 – 155	> 155	Hospitalization for malignant hypertension
Hypertension (diastolic) - mm Hg	91 – 95	96 – 100	> 100	Hospitalization for malignant hypertension
Hypotension (systolic) – mm Hg	85 – 89 and symptomatic	80 – 84 and symptomatic and requiring oral fluids	< 80 and symptomatic and requiring IV fluids	Hospitalization for hypotensive shock
Respiratory Rate – breaths per minute	17 – 20	21 – 25	> 25	Intubation

1. In addition to grading the measured local reaction at the greatest single diameter, the measurement should be recorded as a continuous variable.
2. Induration/Swelling should be evaluated and graded using the functional scale as well as the actual measurement.
3. Subject should be at rest for all vital sign measurements.
4. Oral temperature; no recent hot or cold beverages or smoking.
5. When resting heart rate is between 60 – 100 beats per minute. Use clinical judgment when characterizing Bradycardia among some healthy subject populations, for example, conditioned athletes.

Systemic (General)	Mild (Grade 1)	Moderate (Grade 2)	Severe (Grade 3)	Potentially Life Threatening (Grade 4)
Nausea/vomiting	No interference with activity or 1 – 2 episodes/24 hours	Some interference with activity or > 2 episodes/24 hours	Prevents daily activity, requires outpatient IV hydration	Hospitalization for hypotensive shock
Diarrhea	2 – 3 loose stools or < 400 gms/24 hours	4 – 5 stools or 400 – 800 gms/24 hours	6 or more watery stools or > 800gms/24 hours or requires outpatient IV hydration	Hospitalization
Headache	No interference with activity	Repeated use of non-narcotic pain reliever > 24 hours or some interference with activity	Significant; any use of narcotic pain reliever or prevents daily activity	Hospitalization
Fatigue	No interference with activity	Some interference with activity	Significant; prevents daily activity	Hospitalization
Myalgia	No interference with activity	Some interference with activity	Significant; prevents daily activity	Hospitalization

Systemic Illness	Mild (Grade 1)	Moderate (Grade 2)	Severe (Grade 3)	Potentially Life Threatening (Grade 4)
Illness or clinical adverse event (as defined according to applicable regulations)	No interference with activity	Some interference with activity not requiring medical intervention	Prevents daily activity and requires medical intervention	Hospitalization

B. Tables for Laboratory Abnormalities

Serum *	Mild (Grade 1)	Moderate (Grade 2)	Severe (Grade 3)	Potentially Life Threatening (Grade 4)
Sodium – Hyponatremia mEq/L	132 – 134	130 – 131	125 – 129	< 125
Sodium – Hypernatremia mEq/L	144 – 145	146 – 147	148 – 150	> 150
Potassium – Hyperkalemia mEq/L	5.1 – 5.2	5.3 – 5.4	5.5 – 5.6	> 5.6
Potassium – Hypokalemia mEq/L	3.5 – 3.6	3.3 – 3.4	3.1 – 3.2	< 3.1
Glucose – Hypoglycemia mg/dL	65 – 69	55 – 64	45 – 54	< 45
Glucose – Hyperglycemia<br>Fasting – mg/dL<br>Random – mg/dL	100 – 110<br>110 – 125	111 – 125<br>126 – 200	>125<br>>200	Insulin requirements or hyperosmolar coma
Blood Urea Nitrogen BUN mg/dL	23 – 26	27 – 31	> 31	Requires dialysis
Creatinine – mg/dL	1.5 – 1.7	1.8 – 2.0	2.1 – 2.5	> 2.5 or requires dialysis
Calcium – hypocalcemia mg/dL	8.0 – 8.4	7.5 – 7.9	7.0 – 7.4	< 7.0
Calcium – hypercalcemia mg/dL	10.5 – 11.0	11.1 – 11.5	11.6 – 12.0	> 12.0
Magnesium – hypomagnesemia mg/dL	1.3 – 1.5	1.1 – 1.2	0.9 – 1.0	< 0.9
Phosphorous – hypophosphatemia mg/dL	2.3 – 2.5	2.0 – 2.2	1.6 – 1.9	< 1.6
CPK – mg/dL	1.25–1.5 xULN**	1.6 – 3.0 x ULN	3.1 –10 x ULN	> 10 x ULN
Albumin – Hypoalbuminemia g/dL	2.8 – 3.1	2.5 – 2.7	< 2.5	--
Total Protein – Hypoproteinemia g/dL	5.5 – 6.0	5.0 – 5.4	< 5.0	--
Alkaline phosphate – increase by factor	1.1 – 2.0 x ULN	2.1 – 3.0 x ULN	3.1 – 10 x ULN	> 10 x ULN
Liver Function Tests –ALT, AST increase by factor	1.1 – 2.5 x ULN	2.6 – 5.0 x ULN	5.1 – 10 x ULN	> 10 x ULN
Bilirubin – when accompanied by any increase in Liver Function Test increase by factor	1.1 – 1.25 x ULN	1.26 – 1.5 x ULN	1.51 – 1.75 x ULN	> 1.75 x ULN
Bilirubin – when Liver Function Test is normal; increase by factor	1.1 – 1.5 x ULN	1.6 – 2.0 x ULN	2.0 – 3.0 x ULN	> 3.0 x ULN
Cholesterol	201 – 210	211 – 225	> 226	---
Pancreatic enzymes – amylase, lipase	1.1 – 1.5 x ULN	1.6 – 2.0 x ULN	2.1 – 5.0 x ULN	> 5.0 x ULN

* The laboratory values provided in the tables serve as guidelines and are dependent upon institutional normal parameters. Institutional normal reference ranges should be provided to demonstrate that they are appropriate.

**ULN" is the upper limit of the normal range.

Hematology *	Mild (Grade 1)	Moderate (Grade 2)	Severe (Grade 3)	Potentially Life Threatening (Grade 4)
Hemoglobin (Female) - gm/dL	11.0 – 12.0	9.5 – 10.9	8.0 – 9.4	< 8.0
Hemoglobin (Female) decrease from baseline	not applicable	1.6 – 2.0	2.1 – 5.0	> 5.0

value - gm/dL				
Hemoglobin (Male) - gm/dL	12.5 – 13.5	10.5 – 12.4	8.5 – 10.4	< 8.5
Hemoglobin (Male) decrease from baseline value – gm/dL	not applicable	1.6 – 2.0	2.1 – 5.0	> 5.0
WBC Increase - cell/mm^3	10,800 – 15,000	15,001 – 20,000	20,001 – 25, 000	> 25,000
WBC Decrease - cell/mm^3	2,500 – 3,500	1,500 – 2,499	1,000 – 1,499	< 1,000
Lymphocytes Decrease - cell/mm^3	750 – 1,000	500 – 749	250 – 499	< 250
Neutrophils Decrease – cell/mm^3-African descent	750-999	500-749	250-499	<250
Not of African descent	1,500 – 2,000	1,000 – 1,499	500 – 999	< 500
Eosinophils - cell/mm^3	650 – 1500	1501 - 5000	> 5000	Hypereosinophilic
Platelets Decreased - cell/mm^3	125,000 – 140,000	100,000 – 124,000	25,000 – 99,000	< 25,000
PT – increase by factor (prothrombin time)	1.10 x ULN**	1.11 – 1.20 x ULN	1.21 – 1.25 x ULN	> 1.25 ULN
PTT – increase by factor (partial thromboplastin time)	1.10 – 1.20 x ULN	1.21 – 1.4 x ULN	1.41 – 1.5 x ULN	> 1.5 x ULN
Fibrinogen increase - mg/dL	400 – 500	501 – 600	> 600	--
Fibrinogen decrease - mg/dL	150 – 200	125 – 149	100 – 124	< 100 or associated with gross bleeding or disseminated intravascular coagulation (DIC)

* The laboratory values provided in the tables serve as guidelines and are dependent upon institutional normal parameters. Institutional normal reference ranges should be provided to demonstrate that they are appropriate.
**ULN" is the upper limit of the normal range.

APPENDIX 6. STUDY TEAM ROSTER

APPENDIX 7. ROLES AND RESPONSIBILITIES

RV 422 PART I & PART II **Roles and Responsibilities**

Protocol Co-Chair(s)

- Responsible for study design and will serve as a liason between the site, vaccine developer (VRC/NIAID) and Sponsor (NIAID), and contribute support to overall project management and the analysis and reporting of study data.
- Provide support to the principal investigator with clinical trial guidance, protocol preparation, protocol review, and regulatory approvals.

Principal Investigator (PI):

- Conduct the study at the MUWRP clinic in Kampala.
- Responsible for local IRB submission and approval, study conduct, and reporting all unanticipated problems and adverse events to the protocol safety team and IRBs.
- Responsible for submitting protocol amendments and annual continuing review at intervals designated by the IRB of Record, and a Final Report in accordance with Title 21, Code of Federal Regulations Part 312.33.
- Take all necessary precautions to ensure that the study does not generate hazardous clinical waste, obtain the proper clearance for all publication and abstracts, and maintain a study regulatory file as instructed by the study IND sponsor (NIAID).

Clinical Trial Consultants

- Responsible for study design, protocol development, and serve as technical advisors and subject matter experts for study execution.

Clinical Site Investigator(s)

- Assist the PI with participant study visits, AE assessment and reporting, and will report findings and study status directly to study Principal Investigator.
- Will coordinate with the PI in the planning, design, and execution of the study.

Laboratory Investigator

- Laboratory Investigator supervises clinical (CAP certified) and research laboratory activities for the study.

Medical Monitor

- Medical Monitor monitors the conduct of the protocol per the approval plan and ensuring protection of human subjects. This may involve periodic review of medical records of enrolled subjects and the research files being maintained by the PI. The Medical Monitor reviews and keeps abreast of adverse events and protocol deviations that occur during the research (all adverse events, including deaths and serious or unexpected side effects, are reported to the Medical Monitor via the PI).

Study Pharmacist

- Responsible for administering, dispensing, and accountability of study drug; assessment and management of adverse events; and review of arising data queries.

IND Sponsor: NIH, NIAID

Vaccine Production and Developer: NIH, NIAID, Vaccine Research Center (VRC)

APPENDIX 8. DIARY CARDS

RV 422 PART I & PART II Participant Diary Card

Makerere University Walter Reed Project
Kampala, Uganda

Version 2.2
26 October 2015

Study ID

[redacted]

Symptoms Severity Scale

None= you have no symptoms

Mild= minimal symptoms; cause minimal or no interference with work, school, or self care activities.

Moderate=notable symptoms; required modification in activity; did not result in loss of work or cancellation of social activities

Severe = incapacitating symptoms; requiring bed rest and/or resulted in loss of work or cancellation of social activities.

For any Problems Call at

[redacted]

Injection Site Severity Scale

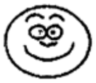

None= you have no symptoms

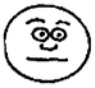

Mild= minimal symptoms; cause minimal or no interference with work, school, or self care activities.

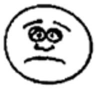

Moderate=notable symptoms; required modification in activity; did not result in loss of work or cancellation of social activities

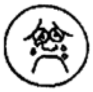

Severe = incapacitating symptoms; requiring bed rest and/or resulted in loss of work or cancellation of social activities.

Day of Vaccination

Date: ____/ ________/__________ (dd/mmm/yyyy)

6 Hours after Vaccination:

Temp: __________ Time Taken: ___________

If you have a fever and wish to take your temperature again, record your additional temperature:
Temp: ________ Time Taken: _______

Temp: ________ Time Taken: _______

Are you taking any medication? ☐ Yes ☐ No
Specify:

Please use the symptom scale at the bottom of the page

**Gen. Symptoms**	**None**	**Mild**	**Moderate**	**Severe**
Aching Joints				
Unusually Tired				
Muscle Aches (not at injection site)				
Headache				
Chills				
Nausea				

**Injection Site Symptoms**	**None**	**Mild**	**Moderate**	**Severe**
Local Pain				
Tenderness				
Swelling Measure in cm	**__ __. __X __. __ cm**		Please record in cm, the largest length and width measurements for the swelling ***(Example: 01.5 X 02.5 cm)***	
Redness Measure in cm	**__ __. __X __. __ cm**		Please record in cm, the largest length and width measurements for the redness ***(Example: 01.5 X 02.5 cm)***	

Symptoms Severity Scale

None= no symptoms
Mild=minimal symptoms; cause minimal or no interference with work, school, or self care activities.
Moderate=notable symptoms; required modification in activity or medication; did not result in loss of work or cancellation of social activities.
Severe = incapacitating symptoms; requiring bed rest and/or resulted in loss of work or cancellation of social activities.

For any problems call at [redacted]

Injection Site Severity Scale
None=no symptoms
Mild=minimal pain or tenderness; no limitation of use of arm
Moderate=notable pain or tenderness; some limitation of use of arm
Severe= extreme pain or tenderness; complete limitation of use of arm

Day 1 after Vaccination

Date: ____/ ________/__________ (dd/mmm/yyyy)

Temp: __________ Time Taken: ___________

If you have a fever and wish to take your temperature again, record your additional temperature:
Temp: ________ Time Taken: _______

Temp: ________ Time Taken: _______

Are you taking any medication? ☐ Yes ☐ No
Specify:

Please use the symptom scale at the bottom of the page

Gen. Symptoms	None	Mild	Moderate	Severe
Aching Joints				
Unusually Tired				
Muscle Aches (not at injection site)				
Headache				
Chills				
Nausea				

Injection Site Symptoms	None	Mild	Moderate	Severe
Local Pain				
Tenderness				
Swelling Measure in cm	**__ __. __X __. __ cm**		Please record in cm the largest length and width measurements for the swelling. ***(Example: 01.5 X 02.5 cm)***	
Redness Measure in cm	**__ __. __X __. __ cm**		Please record in cm the largest length and width measurements for the redness. ***(Example: 01.5 X 02.5 cm)***	

Symptoms Severity Scale

None= no symptoms
Mild=minimal symptoms; cause minimal or no interference with work, school, or self care activities.
Moderate=notable symptoms; required modification in activity or medication; did not result in loss of work or cancellation of social activities.
Severe = incapacitating symptoms; requiring bed rest and/or resulted in loss of work or cancellation of social activities.

For any problems call at [redacted]

Injection Site Severity Scale
None=no symptoms
Mild=minimal pain or tenderness; no limitation of use of arm
Moderate=notable pain or tenderness; some limitation of use of arm
Severe= extreme pain or tenderness; complete limitation of use of arm

Day 2 after Vaccination

Date: ____/ ________/__________ (dd/mmm/yyyy)

Temp: __________ Time Taken: ___________

If you have a fever and wish to take your temperature again, record your additional temperature:
Temp: ________ Time Taken: _______

Temp: ________ Time Taken: _______

Are you taking any medication? ☐ Yes ☐ No
Specify:

Please use the symptom scale at the bottom of the page

Gen. Symptoms	None	Mild	Moderate	Severe
Aching Joints				
Unusually Tired				
Muscle Aches (not at injection site)				
Headache				
Chills				
Nausea				

Injection Site Symptoms	None	Mild	Moderate	Severe
Local Pain				
Tenderness				
Swelling Measure in cm	**__ __. __X __. __ cm**		Please record in cm the largest length and width measurements for the swelling. ***(Example: 01.5 X 02.5 cm)***	
Redness Measure in cm	**__ __. __X __. __ cm**		Please record in cm the largest length and width measurements for the redness. ***(Example: 01.5 X 02.5 cm)***	

Symptoms Severity Scale

None= no symptoms
Mild=minimal symptoms; cause minimal or no interference with work, school, or self care activities.
Moderate=notable symptoms; required modification in activity or medication; did not result in loss of work or cancellation of social activities.
Severe = incapacitating symptoms; requiring bed rest and/or resulted in loss of work or cancellation of social activities.

For any problems call at [redacted]

Injection Site Severity Scale
None=no symptoms
Mild=minimal pain or tenderness; no limitation of use of arm
Moderate=notable pain or tenderness; some limitation of use of arm
Severe= extreme pain or tenderness; complete limitation of use of arm

Day 3 after Vaccination

Date: ____/ ________/__________ (dd/mmm/yyyy)

Temp: __________ Time Taken: ___________

If you have a fever and wish to take your temperature again, record your additional temperature:
Temp: ________ Time Taken: _______

Temp: ________ Time Taken: _______

Are you taking any medication? ☐ Yes ☐ No
Specify:

Please use the symptom scale at the bottom of the page

**Gen. Symptoms**	**None**	**Mild**	**Moderate**	**Severe**
Aching Joints				
Unusually Tired				
Muscle Aches (not at injection site)				
Headache				
Chills				
Nausea				

**Injection Site Symptoms**	**None**	**Mild**	**Moderate**	**Severe**
Local Pain				
Tenderness				
Swelling Measure in cm	**__ __. __X __. __ cm**		Please record in cm the largest length and width measurements for the swelling. ***(Example: 01.5 X 02.5 cm)***	
Redness Measure in cm	**__ __. __X __. __ cm**		Please record in cm the largest length and width measurements for the redness. ***(Example: 01.5 X 02.5 cm)***	

Symptoms Severity Scale

None= no symptoms
Mild=minimal symptoms; cause minimal or no interference with work, school, or self care activities.
Moderate=notable symptoms; required modification in activity or medication; did not result in loss of work or cancellation of social activities.
Severe = incapacitating symptoms; requiring bed rest and/or resulted in loss of work or cancellation of social activities.

For any problems call at [redacted]

Injection Site Severity Scale

None=no symptoms
Mild=minimal pain or tenderness; no limitation of use of arm
Moderate=notable pain or tenderness; some limitation of use of arm
Severe= extreme pain or tenderness; complete limitation of use of arm

Day 4 after Vaccination

Date: ____/ ________/__________ (dd/mmm/yyyy)

Temp: __________ Time Taken: ___________

If you have a fever and wish to take your temperature again, record your additional temperature:
Temp: ________ Time Taken: _______

Temp: ________ Time Taken: _______

Are you taking any medication? ☐ Yes ☐ No
Specify:

Please use the symptom scale at the bottom of the page

Gen. Symptoms	None	Mild	Moderate	Severe
Aching Joints				
Unusually Tired				
Muscle Aches (not at injection site)				
Headache				
Chills				
Nausea				

Injection Site Symptoms	None	Mild	Moderate	Severe
Local Pain				
Tenderness				
Swelling Measure in cm	**__ __. __X __. __ cm**		Please record in cm the largest length and width measurements for the swelling. ***(Example: 01.5 X 02.5 cm)***	
Redness Measure in cm	**__ __. __X __. __ cm**		Please record in cm the largest length and width measurements for the redness. ***(Example: 01.5 X 02.5 cm)***	

Symptoms Severity Scale

None= no symptoms
Mild=minimal symptoms; cause minimal or no interference with work, school, or self care activities.
Moderate=notable symptoms; required modification in activity or medication; did not result in loss of work or cancellation of social activities.
Severe = incapacitating symptoms; requiring bed rest and/or resulted in loss of work or cancellation of social activities.

For any problems call at [redacted]

Injection Site Severity Scale
None=no symptoms
Mild=minimal pain or tenderness; no limitation of use of arm
Moderate=notable pain or tenderness; some limitation of use of arm
Severe= extreme pain or tenderness; complete limitation of use of arm

Day 5 after Vaccination

Date: ____/ ________/__________ (dd/mmm/yyyy)

Temp: __________ Time Taken: ___________

If you have a fever and wish to take your temperature again, record your additional temperature:
Temp: ________ Time Taken: _______

Temp: ________ Time Taken: _______

Are you taking any medication? ☐ Yes ☐ No
Specify:

Please use the symptom scale at the bottom of the page

Gen. Symptoms	None	Mild	Moderate	Severe
Aching Joints				
Unusually Tired				
Muscle Aches (not at injection site)				
Headache				
Chills				
Nausea				

Injection Site Symptoms	None	Mild	Moderate	Severe
Local Pain				
Tenderness				
Swelling Measure in cm	**__ __. __X __. __ cm**		Please record in cm the largest length and width measurements for the swelling. ***(Example: 01.5 X 02.5 cm)***	
Redness Measure in cm	**__ __. __X __. __ cm**		Please record in cm the largest length and width measurements for the redness. ***(Example: 01.5 X 02.5 cm)***	

Symptoms Severity Scale

None= no symptoms
Mild=minimal symptoms; cause minimal or no interference with work, school, or self care activities.
Moderate=notable symptoms; required modification in activity or medication; did not result in loss of work or cancellation of social activities.
Severe = incapacitating symptoms; requiring bed rest and/or resulted in loss of work or cancellation of social activities.

For any problems call at [redacted]

Injection Site Severity Scale
None=no symptoms
Mild=minimal pain or tenderness; no limitation of use of arm
Moderate=notable pain or tenderness; some limitation of use of arm
Severe= extreme pain or tenderness; complete limitation of use of arm

Day 6 after Vaccination

Date: ____/ ________/__________ (dd/mmm/yyyy)

Temp: __________ Time Taken: ___________

If you have a fever and wish to take your temperature again, record your additional temperature:
Temp: ________ Time Taken: _______

Temp: ________ Time Taken: _______

Are you taking any medication? ☐ Yes ☐ No
Specify:

Please use the symptom scale at the bottom of the page

Gen. Symptoms	None	Mild	Moderate	Severe
Aching Joints				
Unusually Tired				
Muscle Aches (not at injection site)				
Headache				
Chills				
Nausea				

Injection Site Symptoms	None	Mild	Moderate	Severe
Local Pain				
Tenderness				
Swelling Measure in cm	**__ __. __X __. __ cm**		Please record in cm the largest length and width measurements for the swelling. ***(Example: 01.5 X 02.5 cm)***	
Redness Measure in cm	**__ __. __X __. __ cm**		Please record in cm the largest length and width measurements for the redness. ***(Example: 01.5 X 02.5 cm)***	

Symptoms Severity Scale

None= no symptoms
Mild=minimal symptoms; cause minimal or no interference with work, school, or self care activities.
Moderate=notable symptoms; required modification in activity or medication; did not result in loss of work or cancellation of social activities.
Severe = incapacitating symptoms; requiring bed rest and/or resulted in loss of work or cancellation of social activities.

For any problems call at [redacted]

Injection Site Severity Scale
None=no symptoms
Mild=minimal pain or tenderness; no limitation of use of arm
Moderate=notable pain or tenderness; some limitation of use of arm
Severe= extreme pain or tenderness; complete limitation of use of arm

Day 7 after Vaccination

Date: ____/ ________/__________ (dd/mmm/yyyy)

Temp: __________ Time Taken: ___________

If you have a fever and wish to take your temperature again, record your additional temperature:
Temp: ________ Time Taken: _______

Temp: ________ Time Taken: _______

Are you taking any medication? ☐ Yes ☐ No
Specify:

Please use the symptom scale at the bottom of the page

Gen. Symptoms	None	Mild	Moderate	Severe
Aching Joints				
Unusually Tired				
Muscle Aches (not at injection site)				
Headache				
Chills				
Nausea				

Injection Site Symptoms	None	Mild	Moderate	Severe
Local Pain				
Tenderness				
Swelling Measure in cm	**__ __. __X __. __ cm**		Please record in cm the largest length and width measurements for the swelling. ***(Example: 01.5 X 02.5 cm)***	
Redness Measure in cm	**__ __. __X __. __ cm**		Please record in cm the largest length and width measurements for the redness. ***(Example: 01.5 X 02.5 cm)***	

Symptoms Severity Scale

None= no symptoms
Mild=minimal symptoms; cause minimal or no interference with work, school, or self care activities.
Moderate=notable symptoms; required modification in activity or medication; did not result in loss of work or cancellation of social activities.
Severe = incapacitating symptoms; requiring bed rest and/or resulted in loss of work or cancellation of social activities.

For any problems call at [redacted]

Injection Site Severity Scale
None=no symptoms
Mild=minimal pain or tenderness; no limitation of use of arm
Moderate=notable pain or tenderness; some limitation of use of arm
Severe= extreme pain or tenderness; complete limitation of use of arm

If you have any of the General or Injection Site Symptoms that is still ongoing beyond day 7, please complete stop dates below.		
**General Symptoms**	**Date the Symptom Ended**	**Maximum severity beyond day 7** (Mild, moderate or severe)
**Aching Joints**		
**Unusually Tired**		
**Muscle Aches (not at injection site)**		
**Headache**		
**Chills**		
**Injection Site Symptoms**	**Date the Symptom Ended**	**Maximum severity beyond day 7** (Mild, moderate or severe)
**Local pain**		
**Tenderness**		
**Swelling**		
**Redness**		

Are you or have you taken any medication after day 7? If so, please record the type of medication the date you stopped taking the medication below.	
**Type of Medication**	**Date You Stopped Medication**
