# Supplementary material for: Heterologous cAd3-Ebola and MVA-EbolaZ vaccines are safe and immunogenic in US and Uganda phase 1/1b trials
Source: NPJ Vaccines. 2024 Mar 29;9:67. doi: 10.1038/s41541-024-00833-z (PMC10980745; doi:10.1038/s41541-024-00833-z)
Supplement: Supplementary file 2 — REPORTING SUMMARY [file 41541_2024_833_MOESM2_ESM.pdf]

nature portfolio

Corresponding author(s): Myra Happe

Last updated by author(s): Dec 27, 2023

Reporting Summary

Nature Portfolio wishes to improve the reproducibility of the work that we publish. This form provides structure for consistency and transparency in reporting. For further information on Nature Portfolio policies, see our Editorial Policies and the Editorial Policy Checklist.

Statistics

For all statistical analyses, confirm that the following items are present in the figure legend, table legend, main text, or Methods section.

n/a	Confirmed	
☐	☒	The exact sample size (*n*) for each experimental group/condition, given as a discrete number and unit of measurement
☐	☒	A statement on whether measurements were taken from distinct samples or whether the same sample was measured repeatedly
☐	☒	The statistical test(s) used AND whether they are one- or two-sided<br>*Only common tests should be described solely by name; describe more complex techniques in the Methods section.*
☐	☒	A description of all covariates tested
☒	☐	A description of any assumptions or corrections, such as tests of normality and adjustment for multiple comparisons
☐	☒	A full description of the statistical parameters including central tendency (e.g. means) or other basic estimates (e.g. regression coefficient) AND variation (e.g. standard deviation) or associated estimates of uncertainty (e.g. confidence intervals)
☐	☒	For null hypothesis testing, the test statistic (e.g. F, t, r) with confidence intervals, effect sizes, degrees of freedom and P value noted<br>*Give P values as exact values whenever suitable.*
☒	☐	For Bayesian analysis, information on the choice of priors and Markov chain Monte Carlo settings
☒	☐	For hierarchical and complex designs, identification of the appropriate level for tests and full reporting of outcomes
☐	☒	Estimates of effect sizes (e.g. Cohen's *d*, Pearson's *r*), indicating how they were calculated

Our web collection on statistics for biologists contains articles on many of the points above.

Software and code

Policy information about availability of computer code

Data collection	ELISA data were collected using PerkinElmer 2030 Manager and Excel.<br>Clinical data : AdvantageEDC (SM), Regulatory Tracking System (RTS) maintained and hosted by by the Clinical Program Support Center (CPSC) at the EMMES Corporation.
Data analysis	Statistical analyses performed in R software version 4.1.3, or in Prism version 10.

For manuscripts utilizing custom algorithms or software that are central to the research but not yet described in published literature, software must be made available to editors and reviewers. We strongly encourage code deposition in a community repository (e.g. GitHub). See the Nature Portfolio guidelines for submitting code & software for further information.

Data

Policy information about availability of data

All manuscripts must include a data availability statement. This statement should provide the following information, where applicable:

- Accession codes, unique identifiers, or web links for publicly available datasets
- A description of any restrictions on data availability
- For clinical datasets or third party data, please ensure that the statement adheres to our policy

The datasets used and/or analyzed during the current study available from the corresponding author on a reasonable request. The study protocols, statistical analysis plans, and informed consent forms are available in the Supplementary Appendix.

Research involving human participants, their data, or biological material

Policy information about studies with human participants or human data. See also policy information about sex, gender (identity/presentation), and sexual orientation and race, ethnicity and racism.

Reporting on sex and gender	Participants self-reported gender during enrollment and these results are reported in Supplementary Tables 2 and 3. No gender-based analysis was performed as this was not an objective of either of the clinical trial protocols. Such investigation is beyond the scope of these small phase 1 clinical trials and should be further investigated in the subsequent clinical investigation phases.
Reporting on race, ethnicity, or other socially relevant groupings	Participants self-reported on their race and ethnicity. Participants chose their race from options including Asian, Black or African American, White, and Multiracial and their ethnicity as either Non-Hispanic or Latino or Hispanic/Latino. For both race and ethnicity, it was permitted to decline self-reporting of these categories, in which case participants are reported as Unknown/Not reported.
Population characteristics	These clinical trials enrolled healthy adults with no exposure to Ebolavirus or Ebolavirus vaccines from the Washington, D.C. metropolitan area (ages 18-50 years, Trial US) or the Kampala, Uganda metropolitan area (ages 18-65 years, Trial UG). In addition, healthy adults with prior exposure to Ebolavirus vaccines were recruited from three sites in the United States: the Washington, D.C. metropolitan area, the Atlanta, Georgia metropolitan area and the Baltimore, Maryland metropolitan area (ages 18-66, Trial US) or the Kampala, Uganda metropolitan are (ages 18-65 years, Trial UG). Inclusion criteria included good general health as determined by laboratory tests, medical history, and physical exam. Exclusion criteria for the naïve participants included prior receipt of an investigational Marburg or Ebola vaccine. Participants with prior exposure to Ebola vaccine(s) had to have a minimum of 12 (Trial US) or 36 (Trial UG) weeks of follow-up since their last vaccine.<br>Between April 27 and November 20, 2015, 140 participants were enrolled into Trial US (Figure 1), including 70 (50%) males and 70 (50%) females with the mean age of 38 years (range: 20-66). Between February 16, 2015, and April 10, 2015, 90 participants were enrolled into Trial UG (Figure 1), including 75 (83%) males and 15 (17%) females, with the mean age of 30 years (range: 19-48).
Recruitment	Naive participants were recruited into Trial US or Trial UG via IRB-approved recruitment materials targeting the environs of the trial sites, including flyers, posters, newspaper ads and radio scripts. Ebola-experienced participants were recruited by contacting participants of previous clinical trials (NCT02231866, NCT00997607) via phone calls or other contact information on record with IRB approval.
Ethics oversight	Trial US was reviewed and approved by the NIAID Institutional Review Board (IRB). Trial UG was approved by the infectious diseases IRB of the Uniformed Services of the Health Sciences (Bethesda, MD, USA), the research and ethics committee of Makerere University School of Public Health (Kampala, Uganda), and the Uganda National Council of Science and Technology (Kampala, Uganda).

Note that full information on the approval of the study protocol must also be provided in the manuscript.

Field-specific reporting

Please select the one below that is the best fit for your research. If you are not sure, read the appropriate sections before making your selection.

☒ Life sciences ☐ Behavioural & social sciences ☐ Ecological, evolutionary & environmental sciences

For a reference copy of the document with all sections, see nature.com/documents/nr-reporting-summary-flat.pdf

Life sciences study design

All studies must disclose on these points even when the disclosure is negative.

Sample size	Primary sample size calculations for safety were expressed in terms of the ability to detect SAEs. For Trial US, within each group of 10 participants, the probability of observing at least one SAE is at least 90% if the true rate of at least one SAE is 0·206 and over 90% probability to observe no SAE if the true rate is no more than 0·01; for participants who received MVA-EbolaZ, there is over 90% chance to observe at least one SAE if the true rate is no less than 0·001 and over 90% chance of observing no SAE if the true rate is no more than 0·023 given the number of vaccinees is 100. For Trial UG, Clopper-Pearson 95% confidence intervals (CIs) for the true rate of at least one event for a sample size of 15 were determined; if one safety event occurred, then the upper limit of 95% CI would be 32·0 and the lower limit 0·2.
Data exclusions	No data were excluded from the analyses.
Replication	ELISA samples were run in triplicate.
Randomization	In Trial US, EVD vaccine-naïve participants were randomized 1:2 to receive 1x10^7 PFU dose of MVA-EbolaZ or 2x10^11 PU dose of cAd3-EBO (Figure 1). The randomization obtained via computer-generated random numbers were provided to the study pharmacist by the protocol statistician. The 1x10^8 PFU dose of MVA-EbolaZ was enrolled thereafter (non-randomized). In Trial UG, EVD vaccine-naïve study participants were randomized by unblinded staff 1:1:1:1 to receive a single injection of cAd3-EBOZ at a dose of 1x10^10 PU or 1x10^11 PU or cAd3-EBO at a dose of 2x10^10 PU or 2x10^11 PU.
Blinding	There was no blinding performed in Trial US or Trial UG.

Reporting for specific materials, systems and methods

We require information from authors about some types of materials, experimental systems and methods used in many studies. Here, indicate whether each material, system or method listed is relevant to your study. If you are not sure if a list item applies to your research, read the appropriate section before selecting a response.

Materials & experimental systems

n/a	Involved in the study
☐	☒ Antibodies
☐	☒ Eukaryotic cell lines
☒	☐ Palaeontology and archaeology
☒	☐ Animals and other organisms
☐	☒ Clinical data
☒	☐ Dual use research of concern
☒	☐ Plants

Methods

n/a	Involved in the study
☒	☐ ChIP-seq
☐	☒ Flow cytometry
☒	☐ MRI-based neuroimaging

Antibodies

Antibodies used	From BD Biosciences: anti-CD28-Cy5PE, anti-CD45RA-Cy7PE, anti-IFN-γ-APC, anti-IL-2-PE, anti-TNF-α-FITC, anti-CD8-Pacific Blue, and Aqua Blue LIVE/DEAD; From ReaMetrix: anti-CCR7-Ax680; From Beckman Coulter: anti-CD3-Cy7APC
Validation	All antibodies were commercially manufactured and validated by BD Biosciences, ReaMetrix, or Beckman Coulter.

Eukaryotic cell lines

Policy information about cell lines and Sex and Gender in Research

Cell line source(s)	ATCC Product number CCL-185, A549 cells were isolated from the lung tissue of a White, 58-year-old male with lung cancer.
Authentication	Since the A549 cell line was sourced from ATCC, independent authentication was not done. ATCC characterization report can be found here: https://www.atcc.org/-/media/product-assets/documents/characterization-data/cell-biology/34535.pdf?rev=32a165549b8e434ca0b3f772a36849e7
Mycoplasma contamination	A549 cell line was routinely tested for mycoplasma with Lonza MycoAert Mycoplasma detection kit and was found mycoplasma negative while in culture and in use for cAd3 neutralization assay.
Commonly misidentified lines (See ICLAC register)	None.

Clinical data

Policy information about clinical studies

All manuscripts should comply with the ICMJE guidelines for publication of clinical research and a completed CONSORT checklist must be included with all submissions.

Clinical trial registration	NCT02408913 and NCT02354404
Study protocol	The protocols for both clinical trials are available on ClinicalTrials.gov for the corresponding trial registration numbers as well as in the Supplementary Appendix for this manuscript.
Data collection	Between April 27 and November 20, 2015, 140 participants were enrolled into Trial US. Between February 16, 2015, and April 10, 2015, 90 participants were enrolled into Trial UG. Data were collected through October 2016 and April 2017 for Trial US and Trial UG respectively.
Outcomes	The primary objectives were to evaluate the safety and tolerability of cAd3-Ebola and MVA-EbolaZ vaccines when administered alone or as a prime-boost regimen. Safety monitoring included a 30-minute post-vaccination monitoring period and clinical and laboratory evaluations at protocol-specified follow-up visits. Participants reported solicited reactogenicity for the first week following each vaccination. Adverse events (AEs) were collected for the first 28 days after each vaccination, while serious adverse events (SAEs) and new chronic medical conditions were recorded throughout the trial. The secondary objectives were to investigate vaccine-induced antibody responses and T cell responses for each vaccine/dose combination, cAd3 neutralizing antibody titers, and the priming effect of a prior Ebola DNA vaccine exposure. Serum and peripheral blood mononuclear cell samples were collected at protocol-specified timepoints for immunogenicity analysis of vaccine-induced antibody and T cell responses. Immunogenicity assays included an enzyme-linked immunosorbent assay (ELISA), qualified intracellular cytokine staining (ICS), and an adenovirus serum neutralization assay.

Plants

Seed stocks

Report on the source of all seed stocks or other plant material used. If applicable, state the seed stock centre and catalogue number. If plant specimens were collected from the field, describe the collection location, date and sampling procedures.

Novel plant genotypes

Describe the methods by which all novel plant genotypes were produced. This includes those generated by transgenic approaches, gene editing, chemical/radiation-based mutagenesis and hybridization. For transgenic lines, describe the transformation method, the number of independent lines analyzed and the generation upon which experiments were performed. For gene-edited lines, describe the editor used, the endogenous sequence targeted for editing, the targeting guide RNA sequence (if applicable) and how the editor was applied.

Authentication

Describe any authentication procedures for each seed stock used or novel genotype generated. Describe any experiments used to assess the effect of a mutation and, where applicable, how potential secondary effects (e.g. second site T-DNA insertions, mosiacism, off-target gene editing) were examined.

Flow Cytometry

Plots

Confirm that:

☒ The axis labels state the marker and fluorochrome used (e.g. CD4-FITC).

☒ The axis scales are clearly visible. Include numbers along axes only for bottom left plot of group (a 'group' is an analysis of identical markers).

☒ All plots are contour plots with outliers or pseudocolor plots.

☒ A numerical value for number of cells or percentage (with statistics) is provided.

Methodology

Sample preparation	Peripheral blood mononuclear cells (PBMC) were isolated from participants' blood samples at baseline and at week 4 after vaccination using Ficoll-Hypaque density centrifugation, cryopreserved and stored at -150°C. PBMC were subsequently thawed, rested overnight, and stimulated for 6 h using a pool of peptides from Ebola GP or Sudan GP. Cells were stained for viability using a LIVE/DEAD Fixable Blue Dead Cell Stain Kit (Invitrogen), stained on the cell surface using antibodies against CD3, CD4, CD8, CD28, CD45RA, and CCR7, and intracellularly using antibodies against interferon gamma (IFN-γ), interleukin 2 (IL-2), and tumor necrosis factor (TNF; all from BD Biosciences, San Jose, CA, USA).
Instrument	BD FACSymphony A5 flow cytometer
Software	BD FACSDiva Software; FlowJo version 10.6.2 and FlowAI.
Cell population abundance	N/A
Gating strategy	The gating strategy is shown in Supplemental Figure 4 from Tapia, et al. 2016, The Lancet Infectious Diseases Volume 16 Issue 1 DOI: https://doi.org/10.1016/S1473-3099(15)00362-X.

☐ Tick this box to confirm that a figure exemplifying the gating strategy is provided in the Supplementary Information.
